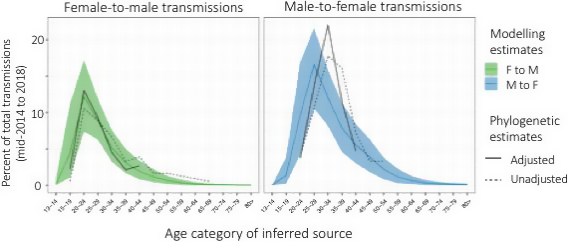# Oral abstracts of the 10th IAS Conference on HIV Science, 21‐24 July 2019, Mexico City, Mexico

**DOI:** 10.1002/jia2.25327

**Published:** 2019-07-24

## MOAB0101

### Virologic efficacy of raltegravir vs. efavirenz‐based antiretroviral treatment in HIV1‐infected adults with tuberculosis: W48 results of the ANRS 12300 Reflate TB2 trial

**N. De Castro^1^; O. Marcy^2^; C. Chazallon^2^; E. Messou^3^; S. Eholié^4^; N. Bhatt^5^; C. Khosa^5^; D. Laureillard^6^; G. Do Chau^7^; V.G. Veloso^8^; C. Delaugerre^1^; X. Anglaret^2^; J.‐M. Molina^1^; B. Grinsztejn^8^ and for the ANRS 12300 Reflate TB2 study group**

^1^APHP‐ Hopital Saint Louis, Paris, France, ^2^University of Bordeaux, Bordeaux Population Health Centre Inserm U1219, Bordeaux, France, ^3^CEPREF, Abidjan, Cote D'Ivoire, ^4^SMIT, Abidjan, Cote D'Ivoire, ^5^Instituto Nacional de Saúde, Maracuene, Mozambique, ^6^CHU de Nîmes, Nîmes, France, ^7^Pham Ngoc Thach Hospital, Ho Chi Minh City, Vietnam, ^8^Oswaldo Cruz Foundation ‐ FIOCRUZ, Rio de Janeiro, Brazil

**Background: **Double‐dose raltegravir is recommended in HIV1‐infected patients with tuberculosis. A previous phase 2 study showed similar efficacy of standard raltegravir 400 mg BID, raltegravir 800 mg BID, or efavirenz‐based regimens. We aimed to assess non‐inferiority of raltegravir 400 mg BID to efavirenz in HIV1‐infected patients with tuberculosis.

**Methods: **ANRS 12300 Reflate TB2 is an open‐label, phase 3, randomized trial conducted in Brazil, Côte d'Ivoire, France, Mozambique, and Vietnam. ART‐naïve HIV1‐infected patients aged ≥18 years on standard tuberculosis treatment for to  weeks were randomized (1:1) to receive raltegravir 400 mg BID or efavirenz 600 mg QD both with TDF/3TC 300 mg/300 mg QD. The primary endpoint was the proportion of patients with virologic success at week 48 defined as HIV‐RNA ≤50 cp/ml on allocated therapy using the FDA snapshot algorithm. The pre‐specified non‐inferiority margin was 12%.

**Results: **From September 2015 to January 2018, 230 patients were randomized in each trial arm: 201 (87%) and 203 (88%) completed follow‐up in the EFV and RAL arms, respectively. Median age was 35 (IQR: 2 to 3) years, 40% were female, median BMI 19.1 (IQR: 17. to 1.0) kg/m^2^, median CD4 102 (IQR: 38 to 239) cells/µL, median HIV‐RNA was 5.5 log (IQR: 5.0 to 5.8), 311 (68%) patients had pulmonary tuberculosis only, and 308 (68%) had bacteriologically‐confirmed tuberculosis. In the mITT population, virologic success was achieved: in 134/228 (59%) pts in the raltegravir arm and 135/227 (59%) pts in the efavirenz arm at W24 (end of TB treatment); in 139/228 (61%) patients in the raltegravir arm and 150/227 (66%) patients in the efavirenz arm at W48. At W48, the difference between the raltegravir and efavirenz arm was ‐5.1% (95% CI: ‐13.9‐ +3.7), thus not meeting criteria for non‐inferiority. Sixty‐two (27%) and 77 (33%) patients experienced grade 3 to 4 adverse events in the raltegravir and efavirenz arms, respectively (*p *= 0.1), including 11 (5%) and 13 (6%) IRIS (*p* = 0.7). Twelve (5%) patients in the raltegravir arm and 14 (6%) in the efavirenz arm died (Logrank *p* = 0.7).

**Conclusions: **The non‐inferiority of raltegravir 400 mg compared to efavirenz at week 48 was not demonstrated. Raltegravir remains a safe option in combination with tuberculosis treatment. Complementary analyses are necessary to identify determinants of virologic failures in both arms.

## MOAB0102

### Week 96 safety and efficacy of the novel HIV‐1 attachment inhibitor prodrug fostemsavir in heavily treatment‐experienced participants infected with multi‐drug resistant HIV‐1 (BRIGHTE study)

**M. Lataillade^1^; J. Lalezari^2^; J. Aberg^3^; J.‐M. Molina^4^; M. Kozal^5^; P. Cahn^6^; M. Thompson^7^; R. Diaz^8^; A. Castagna^9^; M. Gummel^10^; M. Gartland^11^; A. Pierce^11^; P. Ackerman^1^ and C. Llamoso^1^**

^1^ViiV Healthcare, Branford, United States, ^2^Quest Research, San Francisco, United States, ^3^Icahn School of Medicine at Mount Sinai, New York, United States, ^4^Hospital Saint‐Louis, Assistance Publique Hôpitaux de Paris, Paris, France, ^5^Yale University School of Medicine, New Haven, United States, ^6^Fundacion Huesped, Buenos Aires, Argentina, ^7^AIDS Research Consortium of Atlanta, Atlanta, United States, ^8^The Instituto Nacional de Infectologia Evandro Chagas, Fundação Oswaldo Cruz, Rio de Janeiro, Brazil, ^9^Clinic of Infectious Diseases, Vita‐Salute San Raffaele University, Milan, Italy, ^10^GlaxoSmithKline, Upper Providence, United States, ^11^ViiV Healthcare, Research Triangle Park, United States

**Background: **BRIGHTE is an ongoing Phase 3 study evaluating fostemsavir (FTR) in heavily treatment‐experienced (HTE) patients with multidrug resistant HIV‐1 who are unable to form a viable antiretroviral (ARV) regimen. FTR is a prodrug metabolized to temsavir (TMR), a first‐in‐class, investigational attachment inhibitor, which binds directly to HIV‐1 gp120 preventing initial attachment to CD4 receptors on T‐cells, and other host immune cells, thereby blocking infection.

**Methods: **Participants were assigned to the Randomized (RC) or Non‐randomized Cohort (Non‐RC) (Figure‐1). Results through Week 48 were presented previously. Week 96 results are presented here.

**Results: **Participants had a median baseline CD4 count of 80 cells/µL (100 RC;41 Non‐RC); 86% had AIDS. At Week 96, 60% of RC achieved virologic suppression (an increase of 6% from Week 48 despite continued attrition, Table‐1); mean increase in CD4 was 205 cells/µL. Of RC with baseline CD4< 200, 67% increased to CD4≥200; 56% from < 50 to ≥200 cells/µL.

Abstract MOAB0102‐Figure 1. Study Design.

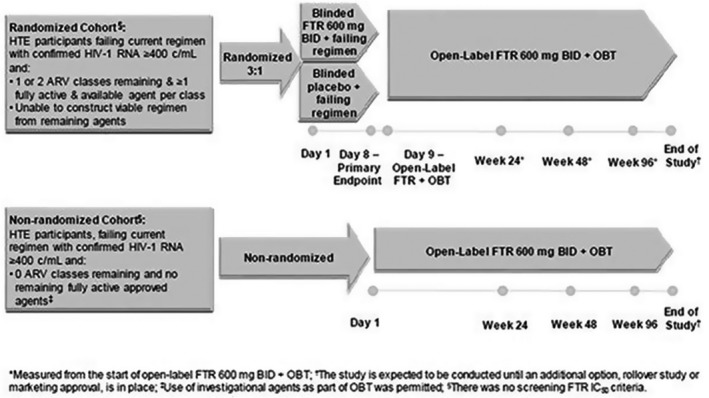

Abstract MOAB0102‐Table 1. Summary of Virologic Response (HIV‐1 RNA <40 c/mL) Over Time by Snapshot Analysis (Intent‐to‐Treat Exposed Population) and Observed Analysis

**Table:** 

	Randomized Cohort N=272		Non‐Randomized Cohort N=99	
	Snapshot n (%)	Observed n (%)	Snapshot n (%)	Observed n (%)
Week 24	144 (53)	141/246 (57)	37 (37)	37/89 (42)
Week 48	146 (54)	145/233 (62)	38 (38)	40/83 (48)
Week 96	163 (60)	170/214 (79)	37 (37)	39/66 (59)

Through Week 96, there were higher rates of severe AEs in the Non‐RC vs. RC: SAE (48%/34%), Grade 3 to 4 AEs (49%/29%), and deaths (16%/4%). Overall, 38% had an SAE; 3% were drug related. 7% discontinued due to AE. Most deaths were attributed to complications of advanced AIDS and acute infection.

**Conclusions: **Fostemsavir‐containing regimens remained generally well‐tolerated through Week 96 with no new safety signal and few AE‐related discontinuations. Virologic and immunologic response continued to improve in this difficult‐to‐treat population. BRIGHTE results support continued development of FTR as a potentially important treatment option for HTE patients with multi‐drug resistant HIV.

## MOAB0103

### Patient views on long acting HIV treatment: Cabotegravir + rilpivirine as maintenance therapy (ATLAS 48 week results)

**M. Murray^1^; A. Antela^2^; A. Mills^3^; V. Chounta^1^; J. Huang^4^; H. Jaeger^5^; M.‐A. Khuong‐Josses^6^; K. Hudson^7^; W. Spreen^7^; P. Williams^8^ and D.M. Margolis^7^**

^1^ViiV Healthcare, Brentford, United Kingdom, ^2^Complejo Hospitalario Universitario de Santiago, La Coruña, Spain, ^3^Southern California Men's Medical Group, West Hollywood, United States, ^4^GlaxoSmithKline, Mississauga, Canada, ^5^MUC Research GmbH and MVZ Karlsplatz, HIV Research and Clinical Care Centre, Munich, Germany, ^6^Hôpital Delafontaine, Saint‐Denis, France, ^7^ViiV Healthcare, Research Triangle Park, United States, ^8^Janssen Research & Development, Beerse, Belgium

**Background: **New modes of HIV treatment are needed to improve adherence and patient choice. ATLAS a phase 3, open‐label study enrolling virally suppressed participants demonstrated switching to monthly long‐acting (LA) formulations of Cabotegravir (CAB) + Rilpivirine (RPV) is non‐inferior to current ART (CAR) at Week 48. A planned secondary analysis of tolerability, health status, and acceptability of switching to a monthly LA regimen has been performed.

**Methods: **Participants who were virologically suppressed for >6 months on an oral regimen of 2 NRTIs + 1 INSTI, NNRTI, or PI were randomly assigned (1:1) to continue CAR or switch to the LA arm. The LA arm received oral CAB + RPV once daily for 4 weeks to assess tolerability prior to monthly CAB LA + RPV LA IM injections. Secondary objectives included treatment satisfaction (HIV‐Treatment Satisfaction Questionnaire), acceptability of treatment (general acceptance domain of ACCEPT), and health status (SF‐12). Tolerability and acceptability of injections (Perception of Injections (PIN)) was assessed in the LA arm only.

**Results: **616 participants were randomized and received treatment. The median age was 42 years with 5.4 years of previous treatment; 203 were women (33%). Participants in the LA group showed greater improvement from baseline in treatment satisfaction at Week 44 compared to CAR (mean +6.12 vs. +0.44; *p* < 0.001), along with greater acceptance of treatment at Week 48 (mean +13.7 vs. +3.0; *p *< 0.001). Overall, 94% and 66% of participants “were satisfied to continue their treatment” in the LA and CAR arms, respectively. There were no differences between LA and CAR arms in health status through Week 48. While 231 (75%) participants in the LA arm had injection site pain, 86% reported their pain as “totally” or “very” acceptable on the “Acceptability of ISRs” in the PIN at Week 48.

**Conclusions: **In addition to demonstrating CAB + RPV LA was non‐inferior to CAR, the LA arm reported higher levels of treatment satisfaction, greater willingness to continue therapy, and increased acceptance of treatment. These results indicate monthly CAB + RPV LA may be an important treatment option for virologically suppressed PLHIV who want an alternative to daily oral therapy.

## MOAB0104

### Virologic failure in ART‐naive HIV patients with high pre‐therapy viral load burden initiating on common core agents

**A.M. Mills^1^; K.L. Schulman^2^; J.S. Fusco^2^; M.B. Wohlfeiler^3^; J. Priest^4^; A. Oglesby^4^; L. Brunet^2^; P. Lackey^5^ and G.P. Fusco^2^**

^1^Men's Health Foundation, Los Angeles, United States, ^2^Epividian, Inc., Durham, United States, ^3^AIDS Healthcare Foundation, Department of Medicine, Los Angeles, United States, ^4^ViiV Healthcare, US Health Outcomes, Durham, United States, ^5^Atrium Healthcare, ID Consultants & Infusion Care Specialists, Charlotte, United States

**Background: **Patients initiating antiretroviral therapy (ART) with viral loads (VL) ≥100,000 copies/mL are less likely to achieve virologic success. We assessed the efficacy of dolutegravir (DTG), elvitegravir (EVG), raltegravir (RAL) and darunavir (DRV) on rates of virologic failure (VF).

**Methods: **ART‐naïve patients with VLs ≥100,000 copies/mL initiating DTG, EVG, RAL, or DRV between 12Aug2013 and 31July2017 were identified. VF was defined as (i)2 consecutive VLs ≥200 copies/mL after 36 weeks of ART, or (ii)1 VL ≥200 copies/mL with core agent discontinuation after 36 weeks, or (iii)2 consecutive VL ≥200 copies/mL after suppression (VL ≤50 copies/mL) before 36 weeks, or (iv)1 VL ≥200 copies/mL with discontinuation after suppression before 36 weeks. Analyses were conducted with Kaplan Meier methods and multivariate Cox modeling.

**Results: **There were 2038 ART‐naïve patients with high VL who initiated DTG (36%), EVG (46%), DRV (16%) or RAL (2%). Median follow‐up was 18.1 months (IQR: 12.4 to 28.9). EVG patients didn't differ from DTG at baseline. RAL patients were older and more likely to be female with low CD4 counts. DRV patients differed notably, especially on baseline characteristics associated with risk for treatment failure. (Table 1) VF was experienced by 9.2% DTG, 13.2% EVG, 18.4% RAL and 18.8% DRV initiators. Compared to DTG, the adjusted hazard ratio for VF was 1.46 (95% CI: 1.05, 2.03) for EVG, 2.24 (1.50, 3.34) for DRV, and 4.13 (1.85, 9.24) for RAL. (Figure 1)

**Conclusions: **ART‐naïve patients with high viral loads initiating on DTG were significantly less likely to experience VF compared to EVG, RAL and DRV initiators.

Abstract MOAB0104‐Table 1. Baseline patient characteristics by core agent

**Table:** 

	DTG n=736	EVG n=928	DTG vs. EVG *p*‐value	RAL n=48	DTG vs. RAL *p*‐value	DRV n=326	DTG vs. DRV *p*‐value
Median(IQR) Age(Yrs)	32.8 (25.7 to 43.2)	32.0 (25.9 to 43.7)	0.8093	40.3 (28.7 to 47.8)	**0.0206**	36.9 (28.7 to 45.4)	**0.0014**
Female (n,%)	90 (12.2%)	99 (10.7%)	0.6035	15 (31.3%)	**0.0015**	46 (14.1%)	0.6023
African American (n,%)	311 (42.3%)	418 (45.0%)	0.2550	21 (43.8%)	0.8391	164 (50.3%)	**0.0149**
Medicaid/Medicare/Ryan White (n,%)	441 (59.9%)	487 (52.5%)	**0.0105**	29 (60.4%)	0.4013	206 (63.2%)	0.2177
AIDS (n,%)	196 (26.6%)	239 (25.8%)	0.6862	15 (31.3%)	0.4844	131 (40.2%)	**<0.0001**
VL≥500K copies/mL (n,%)	147 (20.0%)	208 (22.4%)	0.2274	13 (27.1%)	0.2363	96 (29.4%)	**0.0007**
CD4 Count ≤200 (n,%)	294 (39.9%)	399 (43.0%)	0.2100	30 (62.5%)	**0.0021**	205 (62.9%)	**<0.0001**
Median (IQR) VACS	30 (20 to 53)	30 (20 to 53)	0.9143	46 (30 to 65)	**0.0018**	49 (30 to 65)	**<0.0001**
Hx of Syphilis (n,%)	208 (28.3%)	267 (28.8%)	0.8188	11 (22.9%)	0.4240	112 (34.4%)	**0.0459**

Abstract MOAB0104‐Figuer 1. VF following core agent initiation: unadj. Cumulative probability and adjusted hazard ratio.

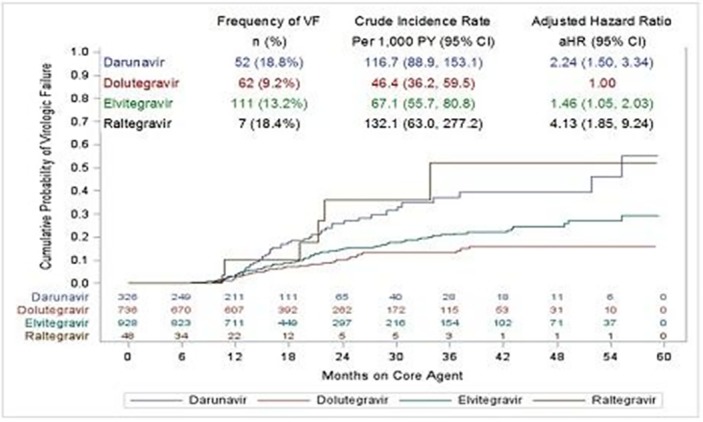

## MOAB0105

### Switching to a single‐tablet regimen bictegravir, emtricitabine, and tenofovir alafenamide (B/F/TAF) from dolutegravir (DTG) plus emtricitabine and either tenofovir alafenamide or tenofovir disoproxil fumarate (F/TAF or F/TDF)

**P.E. Sax^1^; J. Rockstroh^2^; A. Luetkemeyer^3^; Y. Yasdanpanah^4^; D. Ward^5^; B. Trottier^6^; A. Rieger^7^; H. Liu^8^; R. Acosta^8^; S.E. Collins^8^; D. Brainard^8^ and H. Martin^8^**

^1^Brigham and Women's Hospital, Harvard Medical School, Boston, United States, ^2^Bonn University Hospital, Venusburg, Germany, ^3^University of California San Francisco, San Francisco, United States, ^4^Hôpital Bichat Claude Bernard, Paris, France, ^5^Dupont Circle Physicians, Washington DC, United States, ^6^Clinique de Medecine Urbaine du Quartier Latin, Montreal, Canada, ^7^University of Vienna Medical School, Vienna, Austria, ^8^Gilead Sciences, Foster City, United States

**Background: **The single‐tablet regimen B/F/TAF is a guideline‐recommended treatment for HIV‐1. We evaluated whether people receiving dolutegravir (DTG) plus F/TAF or F/TDF can safely and effectively switch to B/F/TAF.

**Methods: **In this phase 3, double‐blinded study, virologically suppressed adults taking DTG plus either F/TAF or F/TDF were randomized (1:1) to switch to B/F/TAF or DTG+F/TAF, once daily with matching placebo. Documented or suspected prior resistance to NRTIs (i.e., M184V, K65R and thymidine analogue mutations [TAMs]), NNRTIs and/or PIs was permitted; INSTI‐resistance was exclusionary. Primary endpoint was the proportion with HIV‐1 RNA ≥50 c/mL at Week (W) 48 (FDA snapshot). Noninferiority was assessed through 95% confidence intervals (CI) using a margin of 4%. Secondary endpoints were the proportion with HIV‐1 RNA < 50 c/mL and change from baseline in CD4 counts at W48. Safety was assessed by adverse events [AEs] and laboratory results.

**Results: **565 participants were randomized/treated (B/F/TAF n=284, DTG+F/TAF n=281): 14% women, 23% Black, median age 51 years (range 20 to 79), 24% had resistance to NRTIs including 5% with K65R or ≥3TAMs, and 14% with M184V/I with or without other mutations. At W48, 0.4% on B/F/TAF and 1.1% on DTG+F/TAF had HIV‐1 RNA ≥50 c/mL demonstrating noninferiority. There was no treatment emergent resistance. No participant with NRTI‐resistance had HIV‐1 RNA ≥50 c/mL at W48. Overall, 93% on B/F/TAF and 91% on DTG+F/TAF had HIV‐1 RNA ≤50 c/mL. Change in CD4 was similar between groups (*p* = 0.23). The most common AEs were nasopharyngitis, diarrhoea, and upper respiratory tract infection. Six (2%) in each group discontinued study drug due to AEs.

**Conclusions: **At W48, switching to B/F/TAF was noninferior to DTG+F/TAF, with high rates of virologic suppression in both groups. The single‐tablet regimen B/F/TAF is an effective option for people virologically suppressed on DTG+F/TDF or F/TAF, with or without NRTI resistance mutations including M184V, K65R and TAMs.

Abstract MOAB0105‐Table

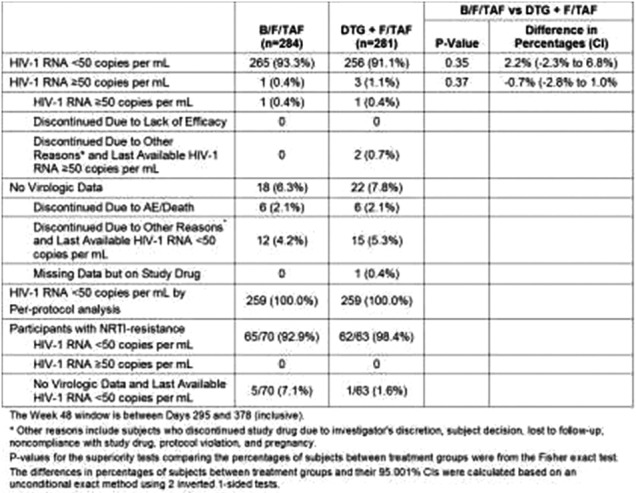

## MOAB0106

### Longer‐term (96‐week) efficacy and safety of switching to bictegravir/emtricitabine/tenofovir alafenamide (B/F/TAF) in women

**C. Kityo^1^; D. Hagins^2^; E. Koenig^3^; A. Avihingsanon^4^; P. Chetchotisakd^5^; K. Supparatpinyo^6^; N. Gankina^7^; V. Pokrovsky^8^; E. Voronin^9^; J.L. Stephens^10^; E. DeJesus^11^; H. Wang^12^; R. Acosta^12^; D. Brainard^12^; H. Martin^12^ and T.A. Makadzange^12^**

^1^Joint Clinical Research Centre, Kampala, Uganda, ^2^Chatham Care Center, Georgia Department of Public Health, Coastal Health District, Savannah, United States, ^3^Instituto Dominicano de Estudios Virologicos IDEV, Dr. Pineyro 211, Zona Universitaria, Santo Domingo, Dominican Republic, ^4^HIV Netherlands Australia Thailand Research Collaboration, The Thai Red Cross AIDS Research Centre, Bangkok, Thailand, ^5^Srinagarind Hospital, Khon Kaen, Thailand, ^6^Chiang Mai University, Chiang Mai, Thailand, ^7^Krasnoyarsk Territorial Center for Prevention and Control of AIDS and Infectious Diseases, Krasnoyarsk, Russian Federation, ^8^Center for Prevention and Control of AIDS, Moscow, Russian Federation, ^9^Federal State Institution ‘Republican Clinical Infectious Hospital’ of the Ministry of Health of the Russian Federation, Saint Petersburg, Russian Federation, ^10^Mercer University School of Medicine, Macon, United States, ^11^Orlando Immunology Center, Orlando, United States, ^12^Gilead Sciences, Foster City, United States

**Background: **Fixed‐dose combination B/F/TAF is recommended regimen for HIV‐1 treatment. We report week (W) 96 results from a phase 3 study evaluating switching to B/F/TAF in a globally distributed cohort of women. Primary outcome at W48 demonstrated noninferior virologic response, good tolerability, and no emergent resistance.

**Methods: **In the randomized phase of this multicentre, open‐label trial, women living with HIV who were virologically suppressed (HIV‐1 RNA < 50 copies/mL) on a baseline regimen (elvitegravir/cobicistat/F/TAF, E/C/F/tenofovir disoproxil fumarate [TDF], or atazanavir+ritonavir+F/TDF) were assigned (1:1) to switch to B/F/TAF (50/200/25 mg) or stay on baseline regimen (SBR) for 48W. At W48, women in the SBR arm switched to B/F/TAF; all participants received B/F/TAF through W96. Secondary efficacy endpoints included proportion with plasma HIV‐1 RNA ≥50 copies/mL (missing=excluded [M=E]) at W96 (for those on B/F/TAF throughout the study) and W48 (for those switched to B/F/TAF after W48). Adverse events (AEs) and laboratory test results were assessed through W96.

**Results: **470 women from the Dominican Republic, Russian Federation, Thailand, Uganda, and the US were treated in the randomized phase (234 B/F/TAF, 236 SBR); 231 continued on B/F/TAF and 228 in the SBR arm switched to B/F/TAF. At W96, virologic suppression (M=E) was maintained in 99.5% (95% CI 97.4%, 100.0%) of the women who received B/F/TAF throughout the study and in 98.5% (95% CI: 95.5%, 99.7%) of women who switched to B/F/TAF at W48. No individual who received B/F/TAF developed treatment‐emergent resistance. Over a median exposure of 76.6W, B/F/TAF was well tolerated, with low frequencies of grade 3 or 4 AEs (6.7%), treatment‐related AEs (5.8%), or serious AEs (5.2%). One participant who received B/F/TAF in the extension phase discontinued treatment due to drug‐related AEs (grade 2 elevated ALT, AST, and GGT). Grade 3 or 4 laboratory abnormalities occurred in 22.1%; most were menses‐associated urine RBCs.

**Conclusions: **B/F/TAF was safe and well tolerated through 96 weeks. Women who switched to B/F/TAF maintained high levels of virologic suppression without emergence of resistance. This analysis supports the efficacy and safety of B/F/TAF observed in other B/F/TAF studies and contributes important long‐term data on safety, tolerability, and efficacy in women living with HIV.

## MOAB0201

### Impact of rosuvastatin on atherosclerotic progression in people with HIV at moderate cardiovascular risk; A multinational, randomized, double blind, placebo‐controlled trial

**J. Trevillyan^12^; A. Dart^2^; M. Cavassini^3^; J. Fehr^4^; C. Staehlin^5^; L. Dewar^6^; A. Calmy^7^ and J. Hoy^6^**

^1^University of California, Los Angeles, Los Angeles, United States, ^2^Monash University, Melbourne, Australia, ^3^Lausanne University Hospital, Lausanne, Switzerland, ^4^University Hospital Zurich, Zurich, Switzerland, ^5^University of Zurich, Zurich, Switzerland, ^6^Alfred Health and Monash University, Melbourne, Australia, ^7^Hospital University Geneva, Geneva, Switzerland

**Background: **People with HIV are at increased risk for coronary artery disease. This study aimed to determine the effect of rosuvastatin on atherosclerotic progression in people with HIV at moderate cardiovascular risk.

**Methods: **Participants with well controlled HIV (suppressed viral load, ART for >6 months) who were at moderate cardiovascular risk (10 year Framingham risk score 10 to 15%) with no indication for statin therapy were recruited from a single centre in Australia and four centres in Switzerland. They were randomised 1:1 (stratified by site) to 20 mg of rosuvastatin or matched placebo. Participants on a protease inhibitor received dose reduced (10 mg) rosuvastatin.

All participants had assessment of carotid intima media thickness (cIMT) and fasting bloods at baseline, week 48 and 96. cIMT was measured at three sites, carotid bulb, common carotid artery (CCA) and internal carotid artery (ICA) bilaterally (the average of the combined sides presented here).

The primary endpoint was the change from baseline to week 96 in CCA cIMT.

**Results: **87 individuals were randomised (55: Australia ‐ 32: Switzerland). Predominantly male (85 [97%]) with a median age 54 years (range 42 to 67), 29 (33%) were current smokers.

There was no difference in baseline IMT between groups; carotid bulb 0.790 mm versus 0.81 mm, *p* = 0.43; CCA 0.690 mm versus 0.722 mm, *p* =  0.447; ICA 0.650 mm versus 0.647 mm, *p* = 0.9252 (rosuvastatin, placebo arms respectively). Despite significantly decreases in LDL cholesterol with rosuvastatin (mean change ‐1.06 mmol/L versus ‐0.06 mmol/L, *p *< 0.0001) there was no difference in progression of IMT from baseline to 96 weeks at any site [carotid bulb (*p* = 0.211), CCA (*p* = 0.876) or ICA (*p* = 0.950)] in those on rosuvastatin. At week 96 there was no difference in cIMT at any site between treatment arms (*p* = 0.993, *p* = 0.791, *p* = 0.462 respectively).

One participant developed type 2 diabetes and one cerebrovascular disease (both on rosuvastatin). Three participants had acute myocardial infarctions while on study (two on rosuvastatin, one on placebo). Two participants (one from each arm) had significant increases in creatinine kinase.

**Conclusions: **In this study of people with well controlled HIV at moderate cardiovascular risk who did not otherwise warrant statin therapy addition of rosuvastatin did not alter the progression of cIMT over 96 weeks.

## MOAB0202

### History of pulmonary opportunistic infection makes no attributable difference to long‐term pulmonary function of people living with HIV who smoke

**K.H. Tram^1^; J.A. O'Halloran^2^; R. Presti^2^ and J. Atkinson^3^**

^1^Washington University School of Medicine, St. Louis, United States, ^2^Washington University School of Medicine, Division of Infectious Diseases, St. Louis, United States, ^3^Washington University School of Medicine, Division of Pulmonary and Critical Care Medicine, St. Louis, United States

**Background: **Evidence suggests an accelerated rate of COPD and other lung diseases in people living with HIV (PLWH). We aimed to examine the long‐term sequelae of pulmonary opportunistic infections (OIs) in smokers living with HIV.

**Methods: **We recruited PLWH 30 years or older who had a 15 pack‐year history of smoking or were current smokers. Participants completed:

1. health questionnaire addressing demographics, smoking habits, and HIV status;
2. the St. George's Respiratory Questionnaire (SGRQ), scored from 0 to 100 (100 being maximum health impairment); and
3. pulmonary function tests (PFTs). COPD was diagnosed using GOLD criterion (FEV1/FVC < 0.7).

We used chi‐square, two‐sample t‐test, and logistic regression to compare PLWH with and without a history of pulmonary OIs. Data are presented as “mean [SD]”.

**Results: **Of the 153 PLWH, 37 (24%) had a history of pulmonary OIs, (25 (16%) pneumocystis pneumonia, 12 (7.8%) recurrent bacterial pneumonia, and 7 (4.6%) pulmonary tuberculosis). Compared to those without, those with previous OIs were older age (54.3 [7.5] vs. 49.2 [8.2], *p *= 0.001), had lower current CD4+ T cell counts (499 [290] vs. 658 [322], *p *= 0.009), and lower nadir CD4+ T cell counts (103 [131] vs. 213 [166], *p* < 0.001). HAART receipt, viral suppression rates, and smoking history were similar in both groups. There was no significant differences in total or component SGRQ scores. Of the 134 for whom PFTs were available, there was no difference in post‐bronchodilator FVC1 (3.7L vs. 4.0L, *p *= 0.155) or FEV1/FVC ratio (74.5% vs. 77.4%, *p *= 0.163) between the groups. Lower FEV1 (2.7L vs 3.1L, *p *= 0.024) was observed in the previous OIs group, and COPD rates were over double (9, 29% vs 14, 14%, *p *= 0.046). A borderline association with COPD was observed with a prior history of pulmonary OIs (unadjusted OR 2.6 [95% CI 0.997 ‐ 6.783] *p *= 0.051), however adjusted for age, the association attenuates (adjusted OR 1.9 [95% CI 0.680 ‐ 5.157] *p *= 0.225).

**Conclusions: **Our data suggests that a history of pulmonary OIs makes no attributable difference to long‐term pulmonary function in PLWH who smoke, and that other factors such as age and continued smoking may play a more important role in developing COPD.

## MOAB0203

### Validation of serological biomarkers for detection of non‐alcoholic fatty liver disease (NAFLD) and/or advanced liver fibrosis in people living with HIV

**C. Yanavich^12^; A. Pacheco^3^; S. Cardoso^1^; E. Nunes^1^; U. Chaves^1^; R. Santos^1^; M. Morata^1^; V.G. Veloso^1^; B. Grinsztejn^1^; H. Perazzo^1^ and GPC‐Hepatol**

^1^Fundação Oswaldo Cruz, Instituto Nacional de Infectologia Evandro Chagas, Rio de Janeiro, Brazil, ^2^University of California, Los Angeles, United States, ^3^Fundação Oswaldo Cruz, PROCC, Rio de Janeiro, Brazil

**Background: **Patients with HIV infection and non‐alcoholic fatty liver disease (NAFLD) are at increased risk for progression to advanced fibrosis. We aimed to validate the accuracy of serological biomarkers to detect NAFLD and advanced fibrosis in HIV mono‐infected patients.

**Methods: **From Jun‐2015 to Jan‐2018, HIV‐infected patients (n=547) were prospectively enrolled in the PROSPEC‐HIV study [NCT02542020]. At entry, a clinical evaluation, laboratory testing, and liver stiffness measurement (LSM) / Controlled Attenuation Parameter (CAP) using transient elastography (Fibroscan) were performed. Patients with viral hepatitis co‐infection (n=17), abusive alcohol intake [AUDIT>8 (n=54)] or unreliable Fibroscan (n=39) results were excluded. NAFLD was defined by CAP≥248 dB/m and advanced fibrosis by LSM≥8.7 kPa with M or ≥7.2 kPa with XL probes, respectively. Serological biomarkers for steatosis [Steato‐ELSA, Fatty Liver Index (FLI), Hepatic Steatosis Index (HSI), NAFLD Liver Fat Score (NAFLD‐LFS)] and fibrosis [FIB‐4, APRI and NAFLD Fibrosis Score (NFS)] were calculated. The area under the ROC curves (AUROC), sensitivity, specificity, positive predictive value (PPV), negative predictive value (NPV) and likelihood‐ratio (LR) were assessed.

**Results: **437 patients [57% female, median age=44 (IQR 35 to 52) years, BMI=26.1 (23.4 to 29.3) Kg/m^2^, ALT=30 (23 to 43)U/L, CD4=660 (427 to 901) cells/mm^3^] were included. The prevalence [95%CI] of NAFLD and advanced fibrosis were 38% [34 to 43] and 11% [8 to 14], respectively. The AUROCs [95%CI] for diagnosis of NAFLD were 0.854 [0.818 to 0.889], 0.840 [0.804 to 0.877], 0.805 [0.762 to 0.847] and 0.793 [0.750 to 0.836] for Steato‐ELSA; FLI; HSI and NAFLD‐LFS [*p* < 0.001], respectively. The AUROCs [95%CI] for diagnosis of advanced fibrosis were 0.736 [0.659 to 0.814], 0.700 [0.614 to 0.7851] and 0.795 [0.726 to 0.864] for FIB‐4, APRI and NFS [*p* = 0.077], respectively. The Table 1 shows sensitivities, specificities, PPV, NPV and LR.

**Conclusions: **Serological biomarkers accurately predicts steatosis; use in patients with fibrosis demonstrated high specificity and NPV. Integration of these tests should be encouraged as part of routine HIV management for the detection of NAFLD and to exclude advanced liver fibrosis.

**Table:** Abstract MOAB0203‐Table 1. Accuracy of serological biomarkers for NAFLD and advanced liver fibrosis in patients with HIV mono‐infection

	Sensitivity [95%CI]	Specificity [95%CI]	PPV	NPV	LR+	LR+
Biomarkers for diagnosis of NAFLD						
ELSA ≥ 0.386	81% [76 to 87]	74% [69 to 80]	66%	87%	3.19	0.25
FLI ≥ 60	75% [69 to 82]	76 % [70 to 81]	65%	83%	3.09	0.32
HSI ≥ 36	89% [84 to 93]	52% [46 to 58]	53%	88%	1.84	0.22
NAFLD‐LFS ≥ ‐0.640	80% [74 to 86]	63% [57 to 69]	57%	84%	2.17	0.31
Biomarkers for diagnosis of advanced fibrosis						
FIB‐4 ≥ 3.25	4% [0 to 10]	99% [98 to 100]	50%	90%	8.50	0.96
APRI ≥ 1.5	2% [0 to 6]	99% [98 to 100]	25%	90%	2.93	0.99
NFS ≥ 0.676	11% [2 to 20]	98% [97 to 99]	38%	90%	5.31	0.91

## MOAB0204

### Assessment of risk factors for hepatocellular carcinoma in HIV care and treatment programmes across 31 countries: A cross‐sectional survey

**C. Mugglin^1^; P. Coffie^2^; F. Dabis^3^; M. Kuniholm^4^; P. Easterbrook^5^; J. Ross^6^; A. Avihingsanon^7^; C. McGowan^8^; M. Yotebieng^9^; K. Anastos^10^; M. Urassa^11^; S. Duda^8^; M.‐A. Davies^12^; M. Egger^1^; G. Wandeler^1^ and IeDEA Collaboration**

^1^University of Bern, Institute of Social and Preventive Medicine, Bern, Switzerland, ^2^Programme PACCI, CHU Treichville Site de Recherches ANRS, Abidjan, Cote D'Ivoire, ^3^University of Bordeaux, Bordeaux School of Public Health ^ISPED^, Bordeaux, France, ^4^University at Albany, State University of New York, Department of Epidemiology and Biostatistics, Rensselaer, United States, ^5^Infectious Disease Institute, Kampala, Uganda, ^6^TREAT Asia/amfAR ‐ The Foundation for AIDS Research, Bangkok, Thailand, ^7^HIV‐NAT, Thai Red Cross AIDS Research Centre, Bangkok, Thailand, ^8^Vanderbilt University School of Medicine, Nashville, United States, ^9^Ohio State University, Columbus, United States, ^10^Albert Einstein College of Medicine, Department of Epidemiology and Population Health, New York, United States, ^11^The TAZAMA Project, National Institute for Medical Research, Mwanza, Tanzania, United Republic of, ^12^University of Cape Town, Cape Town, South Africa

**Background: **Liver cancer is the fourth leading cause of cancer death worldwide. According to the 2015 Global Burden of Disease Study, alcohol, hepatitis B virus (HBV) and hepatitis C virus (HCV) infections are the three main causes of hepatocellular carcinoma (HCC). We assessed diagnostic practices for these risk factors in >50 HIV clinics across the world.

**Methods: **Cross‐sectional web‐based survey among HIV care and treatment sites participating in the International epidemiology Databases to Evaluate AIDS (IeDEA); 55 HIV clinics from 31 countries in six different regions participated. Data were collected from December 2014 to September 2015.

**Results: **The majority of sites were from low‐income countries (36%) or lower‐middle‐income countries (31%), with 3 (5%) from high‐income countries. Thirty‐eight (69%) sites were tertiary‐care facilities and 44 (80%) were located in urban settings. Sites followed over 550,000 HIV‐positive individuals. Routine HBV testing ranged from 22 to 100% of sites across regions, and routine HCV testing from 0 to 80% (Figure). When any HBV testing was performed, 32 (58%) sites used a rapid HBV surface antigen test and 17 (31%) a laboratory‐based serological test. HBV viral load was performed in less than 25% of sites across regions. Of the 47 (85%) sites reporting any HCV antibody testing, none used rapid tests and HCV viral load was available in 5 (9%) clinics. Alcohol consumption was routinely assessed in 29 (53%) sites, with 12 (39%) using a structured assessment tool (e.g., AUDIT‐C). Five (9%) sites reported having an ongoing screening programme for HCC.

**Conclusions: **Although HBV and HCV testing were conducted in the majority of surveyed HIV clinics, only a minority performed it routinely, with large variation across regions, including across high burden countries. Confirmation of HBV and HCV replication and assessment of hazardous alcohol consumption, the most important modifiable HCC risk factors, were poorly implemented globally.

Abstract MOAB0204‐Figure 1. Screening practices and diagnostic tools for HBV, HCV and alcohol consumption in 55 sites across 6 IeDEA regions.

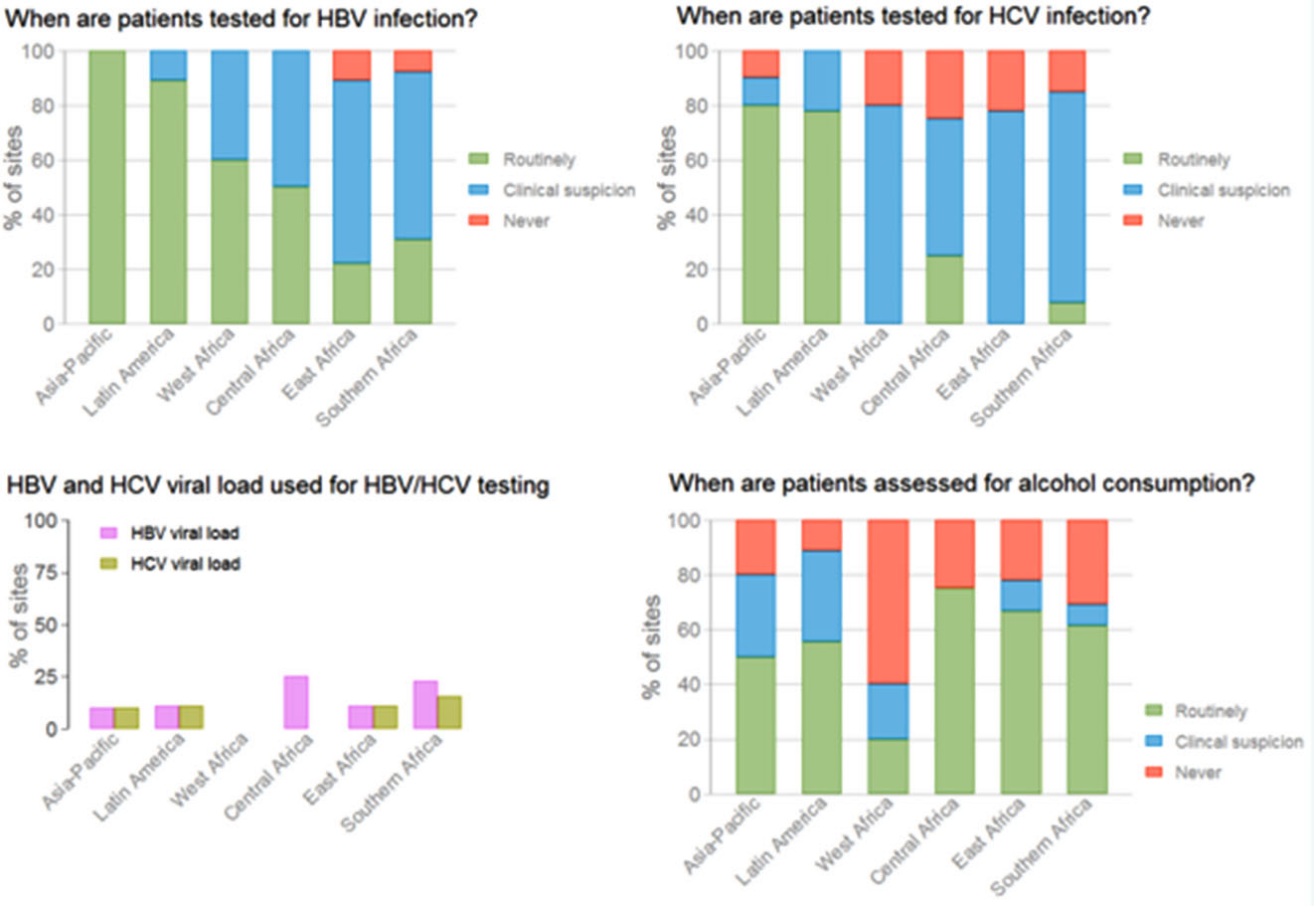

## MOAB0205

### Renal function trajectories after switching from TDF to TAF: A nationwide cohort study

**B. Surial^1^; B. Ledergerber^2^; M. Cavassini^3^; A. Calmy^4^; H. Günthard^2^; M. Stöckle^5^; E. Bernasconi^6^; P. Vernazza^7^; C. Fux^8^; H. Kovari^2^; H. Furrer^1^; A. Rauch^1^; G. Wandeler^1^ and the Swiss HIV Cohort Study**

^1^Bern University Hospital, Department of Infectious Diseases, Bern, Switzerland, ^2^Zurich University Hospital, Division of Infectious Diseases and Hospital Epidemiology, Zurich, Switzerland, ^3^Lausanne University Hospital, Division of Infectious Diseases, Lausanne, Switzerland, ^4^Geneva University Hospital, Division of Infectious Diseases, Geneva, Switzerland, ^5^Basel University Hospital, Division of Infectious Diseases, Basel, Switzerland, ^6^Regional Hospital of Lugano, Division of Infectious Diseases, Lugano, Switzerland, ^7^Cantonal Hospital of St Gallen, Division of Infectious Diseases, St Gallen, Switzerland, ^8^Cantonal Hospital of Aarau, Division of Infectious Diseases, Aarau, Switzerland

**Background: **Tenofovir alafenamide (TAF), characterized by its better renal safety profile than tenofovir disoproxil fumarate (TDF), became available in Switzerland in October 2016. We compared the impact of switching from TDF to TAF on estimated glomerular filtration rate (eGFR).

**Methods: **We included all participants of the Swiss HIV Cohort Study on TDF‐containing antiretroviral therapy with follow‐up after January 2016 who remained on TDF until the end of the observation period (October 2018), or switched to TAF. Baseline was defined as (1) switching date for patients on TAF, (2) October 1^st^ 2016 for patients remaining on TDF, or (3) registration date for patients remaining on TDF and registered after October 1^st^ 2016. We calculated eGFR with the CKD‐EPI formula, and used multivariable linear mixed‐effect models to explore the association between receiving TAF and eGFR over time.

**Results: **Of 3'430 individuals included, 2'499 (72.9%) were male and the median age was 49 years (IQR 42 to 56). At baseline, 1'823 individuals (53.1%) had an eGFR ≥90 ml/min, 1'433 (41.8%) a value of 60 to 89 ml/min, and 174 (5.1%) had an eGFR < 60 ml/min. The median follow‐up time was 15.3 months (IQR 13.4─16.6) for 1'575 individuals who remained on TDF and 11.4 months (8.8─13.6) for 1'855 who switched to TAF. Adjusted eGFR trajectories were similar in both groups if baseline eGFR was ≥90 ml/min (predicted difference in eGFR after 18 months: 0.3 ml/min, 95% confidence interval [CI] ‐1.5─2.0 ml/min) or 60 to 89 ml/min (predicted difference: 1.4 ml/min, 95% CI ‐0.4─3.2 ml/min). In contrast, difference in eGFR at 18 months was 9.6 ml/min (95% CI 5.1─14.0 ml/min) between individuals on TAF compared to those remaining on TDF if baseline eGFR was < 60 ml/min. (Figure 1).

Abstract MOAB0205‐Figure 1. Comparisons of predicted eGFR trajectories over time between TDF (dashed) and TAF (solid).

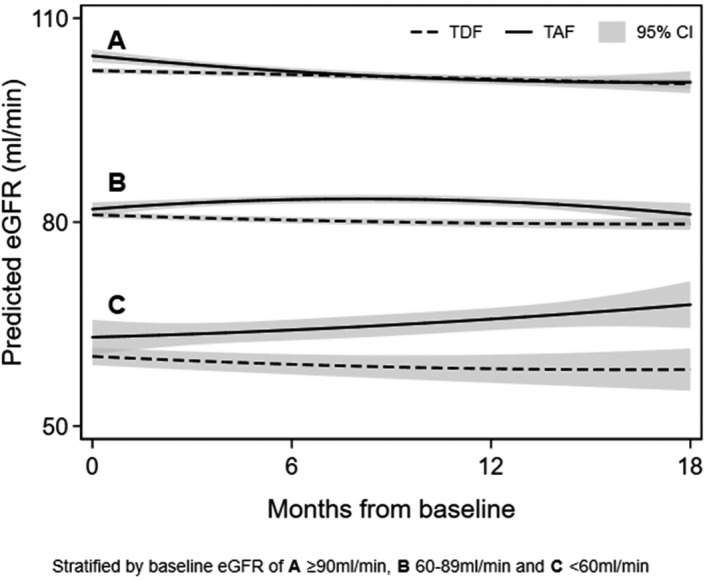

**Conclusions: **TAF was associated with an increase in eGFR over time compared to TDF in patients with moderate to severe impairment of renal function.

## MOAB0206

### Gastrocnemius muscle thickness as a predictor of sarcopenia in people living with HIV

**M. Houssein^1^; A.C. Inkaya^2^; C. Balcı^3^; M. Esme^3^; M.C. Sonmezer^2^; M. Halil^3^; S. Unal^2^ and Hacettepe HIV Metabolic Team**

^1^Hacettepe University School of Medicine, Ankara, Turkey, ^2^Hacettepe University School of Medicine, Infectious Diseases and Clinical Microbiology Department, Ankara, Turkey, ^3^Hacettepe University School of Medicine, Geriatric Department, Ankara, Turkey

**Background: **Sarcopenia is common in ageing people living with HIV (PLWH). Various difficult‐to‐perform methods can be utilized to determine sarcopenia. There is a demand for point‐of‐care sarcopenia diagnostics for ageing HIV population. This study aims to evaluate the role of muscle ultrasound imaging as a screening tool for sarcopenia in PLWH over 40.

**Methods: **PLWH registered in Hacettepe Cohort were invited to this cross‐sectional study. Local IRB reviewed and approved the study protocol. Patients were enrolled after providing informed consent. Inclusion criteria were ART over 6 months, age>40, viral load< 100copies/ml, no co‐infection. Demographic data collected from cohort database. Bioelectrical impedance analysis (fat mass index (FMI), fat free mass index (FFMI), and phase angle), hand grip, gait speed and muscle ultrasound imaging (gastrocnemius muscle thickness) were performed. Sarcopenia was defined as the presence of both low muscle mass (FFMI) and low muscle function (hand grip strength).

**Results: **A total of 95 PLWHA (77.9% male) were included. Mean age was 52.28±8.39 years. Median duration of ART was 60 (6 to 312) months. Median CD4 was 574 (39 to 1389) cells/ml. Mean BMI was 27.15±4.2 kg/m^2^. Median waist circumference and hip circumference were 96±10.6 cm and 103±7.8 cm, respectively. Median FFMI was 21 (16.4 to 27.6) kg/m² and median BFMI was 5.20 (0.60 to 20.6) kg/m². Median hand grip strength was 33.9 (14 to 52.8) kg, median gastrocnemius muscle thickness was 14.2 (8.9 to 20.7) mm, median phase angle was 7.4° (4.10 to 22.8). Sarcopenia was present in 12.6% of patients. FFMI score correlated with gastrocnemius thickness (r=0.560, *p* < 0.001) but not with phase angle (r=0.19, *p* = 0.059). Gastrocnemius muscle thickness also correlated with hand grip strength (r=0.52, *p* < 0.001). Receiver operating characteristic (ROC) curve analysis was performed. Gastrocnemius muscle thickness suggestive of sarcopenia was defined as 13.05 mm (sensitivity 84%, specificity 83%). Positive and negative predictive values were calculated as 42% and 97%, respectively.

**Conclusions: **Sarcopenia, which is suggestive of frail phenotype, is frequently present in PLWH. Muscle ultrasound imaging fulfils the criteria for point‐of‐care diagnostics. Our results suggest that gastrocnemius muscle thickness has a high negative predictive value in diagnosing sarcopenia, thus can be implemented as a screening tool for the detection of sarcopenia in PLWH over 40.

## MOAC0101

### Consumption of recreational drugs and their sexualized use in gay men, bisexual and other men‐who‐have‐sex‐with‐men from Latin America: Preliminary results of the Latin America MSM Internet Survey (LAMIS)

**P. Fernández Dávila^1^; J. Casabona^2^; U. Marcus^3^; M.A. Veras^4^; D. Barros^4^; V. Stuardo^5^; D. Ramírez^6^; K. Jonas^7^; C.F. Cáceres^8^; M. Reyes Díaz^8^; H. Barros^9^; P. Meireles^9^; A.J. Schmidt^10^ and Red Ibero‐Americana de Estudios en Hombres Gayotros HSH y Personas Trans**

^1^Centre d'Estudis Epidemiològics sobre les Infeccions de Transmissió Sexual i Sida de Catalunya, Badalona, Spain, ^2^Centre d'Estudis Epidemiològics sobre les Infeccions de Transmissió Sexual i Sida de Catalunya, Institut de Recerca Biomèdica Germans Trias i Pujol ^IGTP^, Badalona, Spain, ^3^Robert Koch Institute, Berlin, Germany, ^4^Facultad de Ciencias Médicas de la Santa Casa de Sao Paulo, Sao Paulo, Brazil, ^5^Universidad de Chile, Escuela de Salud Pública, Facultad de Medicina,, Santiago de Chile, Chile, ^6^Universidad de San Carlos de Guatemala, Ciudad de Guatemala, Guatemala, ^7^Maastricht University, Faculty of Psychology and Neuroscience, Maastrich, Netherlands, ^8^Universidad Peruana Cayetano Heredia, Centro de Investigación Interdisciplinaria en Sexualidad Sida y Sociedad, Lima, Peru, ^9^Universidade do Porto, Instituto De Saúde Pública, Porto, Portugal, ^10^Sigma Research, London School of Hygiene and Tropical Medicine, London, United Kingdom

**Background: **The increasing prevalence of sexualized drug use among gay, bisexual and other men‐who‐have‐sex‐with‐men (GBMSM) is causing concern globally, as it poses multiple risks for their psycho‐social‐sexual health. This study aimed to describe the sexualized use of recreational drugs, and to characterize users among Latin American GBMSM.

**Methods: **The Latin American MSM Internet Survey was an online questionnaire, available in three languages across 18 Latin American countries. From January‐May 2018, it collected information on sexual behaviour, drug use, psychosocial health, HIV/STI testing, self‐reported HIV/STI diagnosis, and preventive strategies. Participants were mainly recruited through gay apps/web‐pages. The Chi‐square test was used to compare proportions.

**Results: **Of the 64,655 participants, 45.8% had used drugs (excluding alcohol, tobacco, and sedatives) in the last 12 months (Brazil and Southern Cone countries had the highest proportions). Cannabis (29%), poppers (18%), erection‐enhancing medications (15%), cocaine (9.5%) and ecstasy (7.3%) were the most commonly used substances. Injection drug use was reported by 0.3%. Among those who had sex with a man in the last 12 months (n=60,985), 49% had sex under the influence of alcohol or other drugs, 9.9% never had sober sex, and 24% used some recreational drug before/during sex with their last non‐steady partner(s). Overall, 8690 men (13.6%) reported using drugs to enhance or prolong sex during the last 12 months and 6.6% used drugs in a group sex setting. The using drugs to enhance their sexual experience was significantly more likely among men who reported the following characteristics: living in a Southern Cone country, residence in a city of more than one million inhabitants, aged 25 to 40, born abroad, higher education, employment, gay identity, engaging in transactional sex, and diagnosed with HIV. Also among these men, 72% reported condomless anal sex with non‐steady partners in the last 12 months, 53% had been diagnosed with a previous STI; and, among those with no prior HIV‐diagnosis, 2.6% were taking PrEP.

**Conclusions: **The sexualized use of drugs among Latin‐American GBMSM is noticeable, particularly in big cities and Southern Cone countries. This pattern should be taken into account in public health programmes and harm‐reduction interventions included as part of the combined prevention approach.

## MOAC0102

### Polydrug use and HIV sexual risks in a sample of men who inject drugs on the U.S.‐Mexico border

**O. Beltran^1^; J. Lechuga^2^; G. Perez^3^; R. Ramos^3^ and A. Rios^4^**

^1^Programa Compañeros, A.C., Ciudad Juarez, Mexico, ^2^Lehigh University, Bethlehem, United States, ^3^Alliance of Border Collaboratives, El Paso, United States, ^4^Centros de Integración Juvenil, Ciudad Juarez, Mexico

**Background: **Polydrug use among Latino men who inject drugs on the U.S.‐Mexico border has been increasing over the last years yielding to negative health outcomes, (overdose), HIV high‐risk behaviours (e.g. condomless sex), and other STIs. We explored HIV risk behaviours and the patters of drug use by sexual behaviour (i.e. MSM vs non‐MSM) among Latino men who inject drugs.

**Methods: **The sample for this study included 160 men who inject drugs recruited through respondent driven sampling methodology. Data analysis was conducted using SPSS v.25. Independent sample t‐test was used to identify mean differences and chi‐squared to explore proportional differences on binomial substance use, STIs, and sexual risk behaviours.

**Results: **MSM reported higher odds of employment (OR=1.96, *p* = 0.007), but also higher use of inhalants (OR=1.96, *p* = 0.044), methamphetamine (OR=2.42, *p* = 0.005), and history STIs (OR=1.76, *p* = 0.015). Participants were asked to report the number of times that different risk behaviours occur in the past 30 days; significant differences were found on the number of new sex partners reported by MSM vs non‐MSM (Mean: 1.88 vs 0.54, *p* < 0.001), sex under the influence of a drug (Mean: 14.38 vs 6.83, *p* = 0.43), engage in anal sex (Mean: 4.47 vs 1.21, *p* < 0.001), and exchange sex for money (Mean: 3.32 vs 0.92, *p* < 0.001). However, the number of times MSM engaged in condomless sex was lower than non‐MSM (Mean: 4.53 vs 8.99, *p* = 0.005). Finally, more MSM reported experienced sexual abuse before they were 18 years old (OR=1.81, *p* = 0.016), and being gang raped (OR=7.04, *p*=0.007).

**Conclusions: **Although MSM who inject drugs reported lower cases of condomless sex compared to their non‐MSM counterpart, their sexual risk behaviours are higher, including sex under the influence of drugs which can decrease their willingness of use condom and be more susceptible to sexual violence. Risk reduction strategies including Pre‐Exposure Prophylaxis (PrEP) could be a method to effectively reduce their HIV risks.

## MOAC0103

### Still left behind: Using programmatic data to assess harm reduction service coverage and HIV treatment cascades for people who inject drugs in five South African cities

**A. Scheibe^123^; R. Matima^1^; A. Schneider^1^; R. Basson^1^; S. Ngcebetsha^1^; K. Padayachee^1^; Z. Von Homeyer^1^; C. Heathfield^1^; N. Medeiros^4^; A. Manion^5^; J. Hugo^2^; L. Kroukamp^2^; N. Gloeck^2^; U. Bhoora^2^; S. Dada^6^; K. Young^1^; M. Marks^3^; S. Shelly^12^; N. Harker‐Burnhams^6^ and H. Hausler^1^**

^1^TB HIV Care, Cape Town, South Africa, ^2^University of Pretoria, Pretoria, South Africa, ^3^Durban University of Technology, Durban, South Africa, ^4^OUT Wellbeing, Pretoria, South Africa, ^5^Anova Health Institute, Johannesburg, South Africa, ^6^South African Medical Research Council, Cape Town, South Africa

**Background: **A third (14% ‐ 58%) of people who inject drugs (PWID) in South Africa are living with HIV. For HIV epidemic control among PWID UNAIDS recommends 300 needles per PWID per year, 40% opioid substitution therapy (OST) coverage and reaching the 90‐90‐90 treatment targets. By 2018, PWID programming in the country included needle/syringe services (NSS), HIV testing and linkage to care, and opioid substitution therapy (OST). To estimate service coverage, we assessed needle distribution, OST coverage and HIV treatment cascades among PWID accessing harm reduction services in Cape Town, Durban, Johannesburg, Port Elizabeth and Pretoria.

**Methods: **We combined programmatic data from city services between January and June 2018. For this period, we:

1. consolidated counts of PWID accessing NSS;
2. calculated the average number of needles distributed per PWID accessing NSS,
3. divided the number of PWID on OST by the number of PWID accessing NSS as a proxy for OST coverage and
4. counted the numbers tested and treated to develop cascades.

**Results: **During this period, 7316 unique PWID accessed services (700 in Cape Town, 541 in Durban, 1365 in Johannesburg, 361 in Port Elizabeth and 4349 in Pretoria). Overall, 558,983 needles and syringes were distributed (ranging from 60,634 in Port Elizabeth to 225,709 in Pretoria); an average of 76 needles per PWID (ranging from 52 in Pretoria to 174 in Cape Town). 260 PWID were on OST at the end of the period, representing 4% coverage across cities (from 0% in Port Elizabeth to 8% in Cape Town). In total, 1773 (24%) PWID tested for HIV (ranging from 15% in Pretoria to 57% in Cape Town). HIV positivity was 23% (from 3% in Cape Town to 45% in Johannesburg), 20% of whom were initiated onto antiretroviral therapy (ranging from 6% in Durban to 65% in Port Elizabeth). Viral suppression data was unavailable.

**Conclusions: **To reach HIV epidemic control among PWID in these cities, needle distribution needs to double, OST coverage expand ten‐fold and access to HIV testing and treatment increase five‐fold. The viral suppression data gaps need to be filled.

## MOAC0104

### High levels of depression among Peruvian men who have sex with men: Implications for HIV prevention and treatment care

**J. Galea^12^; H. Sánchez^2^; S. León^23^ and B. Brown^24^**

^1^University of South Florida, Social Work, Tampa, United States, ^2^Epicentro Peru, Lima, Peru, ^3^Universidad Privada San Juan Bautista, Lima, Peru, ^4^University of California Riverside School of Medicine, Social Medicine and Population Health, Center for Healthy Communities, Riverside, United States

**Background: **Annual depression rates among men who have sex with men (MSM) from high income countries are three‐ to nine‐fold higher than the general population (12%‐36% vs. 4%, respectively); however, little is known about depression rates in MSM in low‐ and middle‐income countries (LMIC). Among HIV‐negative MSM, depression is associated with increased alcohol/drug use and decreased condom use during sexual intercourse while for HIV‐positive MSM, depression reduces accessing or adhering to medical care and is associated with worse long‐term survival. The present study assessed the prevalence of depression among MSM seeking HIV/STI services in Peru, a LMIC.

**Methods: **Between August 2017 and December 2018, MSM presenting for HIV/STI services at the community‐based organization Epicentro were offered depression screening using the Peruvian‐validated version of the Patient Health Questionnaire (PHQ‐9). The PHQ‐9, used globally, consists of 9 questions that measure the frequency of core depression symptoms on a scale ranging from “0” (not at all) to “3” (nearly every day). PHQ‐9 scores ≥ 5 are suggestive of depression with the highest score (27) corresponding to severe levels.

**Results: **A total of 185 MSM consented to depression screening, of whom 13% tested positive for HIV and 87% tested negative; 4 participants sought other STI services and declined HIV testing. Mean participant age was 28.77 years (range, 17 to 58). Alcohol and/or drug use during last sexual encounter was reported by 20% of men. Depression prevalence was: 58% none/no depression (PHQ‐9 = 0 to 4); 23% mild (PHQ‐9 = 5 to 9); 12% moderate (PHQ‐9 = 10 to 14); 5% moderately severe (PHQ‐9 = 15 to 19); and 2% severe (PHQ‐9 = 20 to 27). There was an association with depression (PHQ‐9 score ≥5) for both having a positive HIV test result and alcohol use though not statistically significant (*p*=0.39 and 0.09, respectively).

**Conclusions: **Depression was common among Peruvian MSM, with >40% scoring positive for the disorder. Most depression severity was mild‐ to moderate, which could be treated by brief, non‐pharmacological depression interventions. Though no significant association between depression and HIV or alcohol use was observed, both are known depression risk factors meriting future research in LMIC. Finally, future research must include more diverse populations, especially transgender women.

## MOAC0105

### Gender differences in syringe‐related policing behaviours and attitudes following a police education programme in Tijuana, Mexico: A longitudinal mixed methods analysis

**M.L. Mittal^12^; T. Rocha Jiménez^2^; I. Artamonova^2^; S.A. Strathdee^2^; M. Morales^3^; J. Cepeda^2^; P. Baker^2^; E. Clairgue Caizero^3^; A. Bañuelos Pérez^4^; J. Arredondo^2^; T. Patterson^2^ and L. Beletsky^25^**

^1^Universidad Xochicalco, Escuela de Medicina, Tijuana, Mexico, ^2^University of California, San Diego, Division of Infectious Diseases and Global Public Health, La Jolla, United States, ^3^Comisión de Salud Fronteriza México‐Estados Unidos, Tijuana, Mexico, ^4^Dirección de Planeación y Proyectos Estratégicos, Secretaría de Seguridad Pública Municipal, Tijuana, Mexico, ^5^Northeastern University, School of Law & Bouvé College of Health Sciences, Boston, United States

**Background: **Certain policing practices (i.e., syringe confiscation, syringe‐related arrests) continue to fuel HIV transmission among persons who inject drugs (PWID) and are barriers to HIV prevention worldwide. We aimed to explore gender differences in syringe‐related policing behaviours and attitudes towards PWID among law enforcement officers (LEOs) following a police education programme (PEP) in Tijuana, Mexico.

**Methods:** *Proyecto Escudo* was a PEP delivered by peer instructors and multimedia covering occupational health (i.e., needle‐stick injuries [NSIs]) and harm reduction topics. This mixed methods analysis drew from a random subsample of follow‐up assessments with LEOs reporting contact with syringes (2015 to 2016): pre‐PEP and 3‐month post‐PEP self‐administered surveys, and linked qualitative interviews. Longitudinal logistic regression with gender as a main predictor and robust variance estimation via GEE was used to assess associations between gender and syringe‐related policing behaviours and attitudes, which were further explored with qualitative narrative analysis.

**Results: **In our baseline subsample (n=766), 29.5% female LEOs (n=33) arrested someone for syringe possession versus 42.2% male LEOs (n=275; *p*=0.01), which significantly decreased 3‐months post‐PEP [subsample n=565; 18.7% female (n=14) versus 31.2% males (n=153); p=0.03]. Pre‐PEP, females were less likely than males to confiscate syringes (OR: 0.62; 95%CI: 0.41 to 0.93; *p*=0.02) and arrest someone for syringe possession (OR: 0.57; 95%CI: 0.37 to 0.88; *p*=0.01). Female LEOs were less likely to arrest someone for syringe possession 3‐months post‐PEP (OR: 0.51; 95%CI: 0.27 to 0.93; *p*=0.03), more likely to disagree with “laws that reduce penalties on drug users make my job more difficult” (OR: 1.76; 95%CI:1.08 to 2.87; *p*=0.02), and more likely to refer PWID to social/health programmes (OR: 1.89; 95%CI: 1.13 to 3.15; *p*=0.01). Qualitative analysis (n=20) revealed post‐NSI behaviour change and attitude changes towards PWID (“People do change…Many people do better their lives”). Female LEOs also reflected on how their own NSI experiences would have been different with tools learned during the PEP, “I didn't know, that's why I didn't do it [test for HIV].”

**Conclusions: **LEOs improved policing practices and attitudes following a PEP; however, these data suggest greater improvement among female LEOs, which persisted 3‐months post‐PEP. Female LEOs may serve as potential peer models to prevent NSIs and reduce HIV‐related harms among PWID.

## MOAD0101

### Policing as a structural determinant of HIV risk among people who inject drugs: A systematic literature review

**P. Baker^12^; L. Beletsky^13^; L. Avalos^1^; C. Venegas^1^; S.A. Strathdee^1^ and J. Cepeda^1^**

^1^University of California San Diego, Division of Infectious Disease and Global Public Health, San Diego, United States, ^2^San Diego State University, Graduate School of Public Health, San Diego, United States, ^3^Northeastern University, School of Law and Bouvé College of Health Sciences, Boston, United States

**Background: **The law and its enforcement are structural determinants of the HIV risk environment among people who inject drugs (PWID). Certain policing practices, such as syringe confiscation are consistently associated with increased HIV risk, but these relationships have not been systematically assessed. Our objective was to conduct a systematic literature review to provide a quantitative synthesis of policing practices acting as structural risk factors for HIV and its risk behaviours among PWID.

**Methods: **From September 2017 to November 2018, we conducted a systematic literature review (PROSPERO #CRD42018105967) screening MEDLINE, sociological databases and grey literature for quantitative studies conducted from 1981 to 2018 that included estimates of HIV infection or risky IDU behaviours and associations with policing practices that are adversely related to PWID health (syringe confiscation, beatings, arrest, etc). Abstracts were screened and those identified to contain elements of HIV risk and policing behaviours among PWID were selected for further review. We abstracted data on drug related harms and policing practices from eligible studies.

**Results: **Of 8201 abstracts screened, 175 full text articles were reviewed; 26 eligible articles presenting associations between policing and HIV risk behaviours among PWID were included. Eligible studies originated from nine countries (Russia, Mexico, USA, Canada, Ukraine, Thailand, Malaysia, China and India) across various per‐capita GDP income levels. HIV infection was significantly associated with syringe confiscation (Odds Ratio [OR]=2.04;95% Confidence Interval [CI]=1.00 to 4.21 and OR=2.38;CI=1.17 to 4.81) new syringe confiscation (OR=5.50;CI=1.80 to 16.6), not buying syringes for fear of police (OR=3.30;CI=1.40 to 7.60), not carrying syringes for fear of police (OR=2.20;CI=1.10 to 4.40), rushed injection due to police presence (OR=20.6;CI=10.00 to 42.70), pre‐loaded syringe confiscation (OR=3.50;CI=1.906.40), fear of arrest (OR=0.62;CI=0.42 to 0.93), forced to buy back syringe from police (OR=2.90;CI=1.50 to 5.40), arrested for planted drugs (OR=3.00;CI=1.30 to 6.80), beaten or tortured (OR=3.10;CI=1.50 to 6.50 and OR=1.35;CI=1.08 to 1.67).

**Conclusions: **Policing practices influencing HIV and drug‐related risk were pervasive among PWID populations with high HIV burden across diverse global settings. There is an urgent need for interventions to transform police encounters with PWID from a source of harm to a source of harm reduction.

## MOAD0102

### Witch‐hunt in Brazil: Bill 198/2015, criminalization of HIV transmission and pathologization of dissident sexualities

**L. Oliveira**

University of São Paulo, Anthropology Department of the Faculty of Philosophy, Languages and Human Sciences, São Paulo, Brazil

**Background: **Mass media and the National Congress has been working together on the construction of an imaginary of “risk”, especially from the “Clube do Carimbo”, a supposed practice of purposely transmitting the virus by seropositive homosexuals. That notion has been used to ground a law that would make the intentional transmission of HIV a heinous crime ‐ bill 198/2015. The objective of this research is to present a cartography of controversies involving parliamentarians, academics, activists, social movements, NGOs, international organizations and Ministry of Health.

**Methods: **The methodology is qualitative and is based on the analysis of public documents produced by social actors in the process related to bill 198/2015. A database was created with materials published in the media and in the media of the movement against AIDS, government programmes and international agencies; booklets and notes produced by the social movement; scientific articles. And the entire process of that bill in the National Congress, as well as its debate through public hearings or demonstrations of parliamentarians involved were monitored.

**Results: **The outcomes are:

1. the construction of the notion of risk promotes the persecution of LGBTI, sex workers, the black population and immigrants;
2. the construction of the “Clube do Carimbo” as a panic reifies the old project of pathologizing dissident sexualities and promotes an increase in stigma and discrimination against people living with HIV/Aids - PLWHA;
3. there is an individualization of the epidemic, focused on the responsibility of the PLWHA;
4. There is a national and international increase in punitive policies that criminalize PLWHA based on sexual behaviour.

**Conclusions: **The social construction of sexual behaviours seen as a threat, as well as the logic of individuality, drive the criminalization of HIV transmission and stigmatize and discriminate PLWHA and certain social groups. And the handling of the scientific evidence “undetectable equals untransmittable” has proved to be very useful and needs to be studied more closely, especially in countries with advances in prison devices.

## MOAD0103

### Utilizing individual level data to characterize the relationship between HIV infection and the legal context of sex work across 10 countries in sub Saharan Africa

**C. Lyons^1^; S. Baral^1^; S. Schwartz^1^; S. Murray^2^; K. Shannon^3^; D. Diouf^4^; T. Mothopeng^5^; S. Kouanda^6^; A. Simplice^7^; Z. Mnisi^8^; A. Kouame^9^; U. Tamoufe^10^; R.N. Phaswana‐Mafuya^11^; B. Cham^12^ and F. Drame^4^**

^1^Johns Hopkins Bloomberg School of Public Health, Department of Epidemiology, Baltimore, United States, ^2^Johns Hopkins Bloomberg School of Public Health, Department of Mental Health, Baltimore, United States, ^3^Centre for Gender & Sexual Health Equity/ University of British Columbia, Vancouver, Canada, ^4^Enda Sante, Dakar, Senegal, ^5^People's Matrix Association, Maseru, Lesotho, ^6^Institut de Recherche en Sciences de la Santé, Ouagadougou, Burkina Faso, ^7^ONG Arc‐en‐Ciel, Lome, Togo, ^8^Health Research Department, Strategic Information Division, Ministry of Health, Mbabane, Eswatini, ^9^Ministère de la Sante et de l'Hygiène Publique, Abidjan, Cote D'Ivoire, ^10^Metabiota, Yaounde, Cameroon, ^11^Human Sciences Research Council, Port Elizabeth, South Africa, ^12^Actionaid, Banjul, Gambia

**Background: **The legal and policy environment has been established as a key structural determinant of HIV risk for female sex workers and has become a focus for HIV response. Ecological studies of country level data have observed a relationship between HIV and legal status of sex work. The majority of studies have examined legal status of sex work and health outcomes through ecological studies and systematic reviews, highlighting the need for empiric data. In response, the aims of this study are to use pooled individual‐level data examine the relationship between HIV and legal environments.

**Methods: **Respondent driven sampling was used to recruit sex workers over the period of 2011 to 2018 across 10 countries: Burkina Faso, Cameroon, Côte d'Ivoire, Gambia, Guinea‐Bissau, Lesotho, Senegal, eSwatini, South Africa and Togo. Interviewer‐administered socio‐behavioural questionnaires and biological testing for HIV were conducted. Legal status of sex work for countries was defined and categorized based on the legal approach: Not specified; partially legalized; criminalized. Individual‐level data were pooled across countries. Multivariable logistic regression was used to measure the association between legal status and HIV.

**Results: **HIV prevalence among sex workers in contexts with partial legalization was 11.6%(219/1908), 19.6%(248/1266) within contexts where selling sex is not legally specified, and 40.4%(1605/3985) within criminalized settings. Legal status of sex work was associated with HIV(*p*‐value< 0.001). When compared to settings with partial legalization, criminalized status(aOR:7.6;95%CI:2.2, 26.6), and not legally specified(aOR:2.5;95%CI:1.1, 5.4) were associated with increased odds of HIV.

**Conclusions: **Consistently, the legal context of sex work was associated with prevalent individual HIV infection among sex workers. The magnitude of this relationship was highest among individuals in criminalized setting, followed by individuals in setting where the legal status of selling sex was not specified. These results highlight that laws contribute to individual level outcomes and decriminalization alongside supportive services should be established to effectively address the HIV epidemic.

Abstract MOAD0103‐Figure 1. HIV infection and country level legal status.

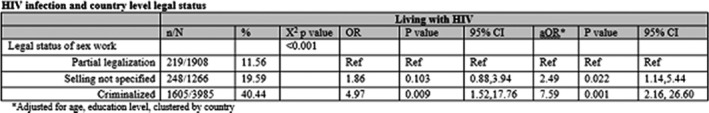

Abstract MOAD0103‐Figure 1. Index testing cascade in CIRKUITS project.

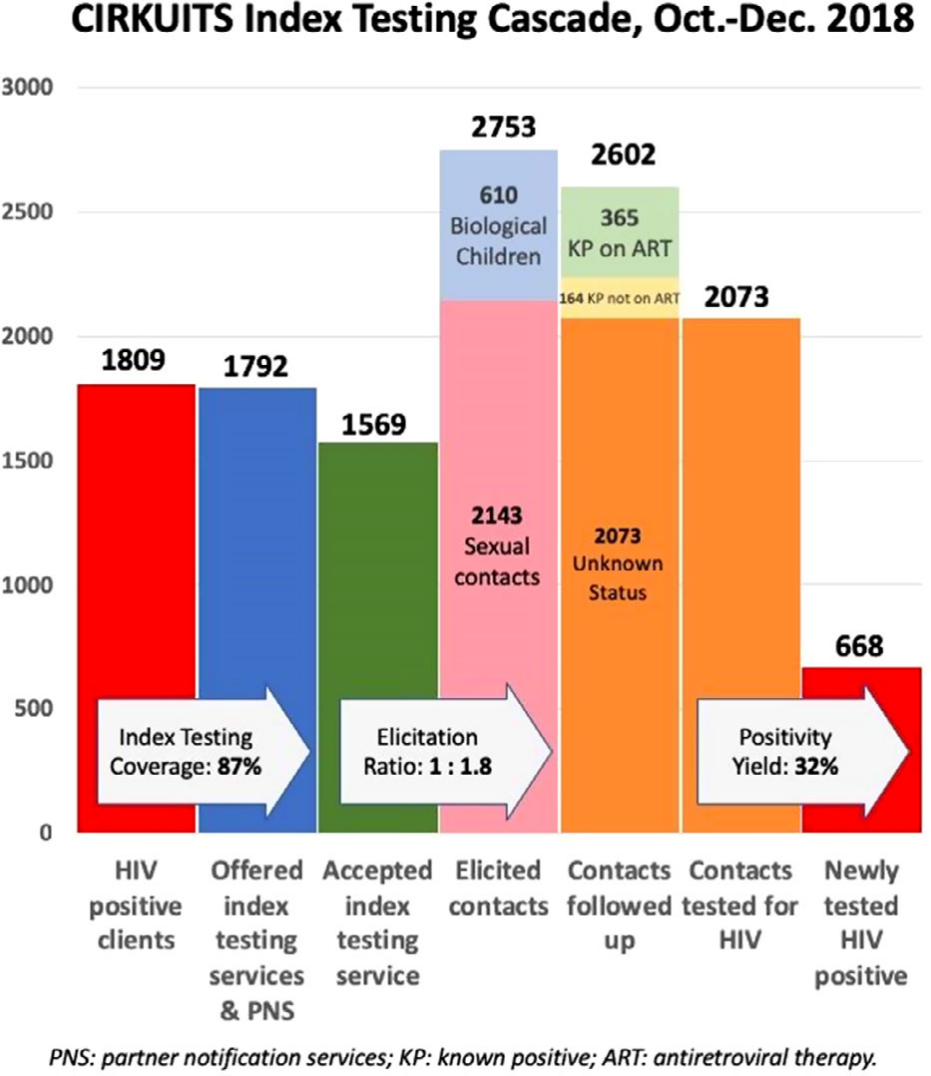

## MOAD0104

### A pathway to policy commitment for sustainability of a key population‐led health services model in Thailand

**P. Sakolsatayadorn^1^; S. Wattanayingcharoenchai^2^; S. Kanjana‐Wattana^3^; S. Tanprasertsuk^4^; P. Sirinirund^4^; S. Janyam^5^; D. Linjongrat^6^; P. Chanlearn^7^; R. Airawanwat^8^; S. Pengnonyang^8^; P. Na Nakorn^9^; S. Charoenying^10^; R. Vannakit^11^; N. Phanuphak^8^ and P. Phanuphak^8^**

^1^Office of Permanent Secretary, Ministry of Public Health, Bangkok, Thailand, ^2^Ministry of Public Health, Department of Disease Control, Bangkok, Thailand, ^3^National Health Security Office, Bangkok, Thailand, ^4^Country Coordinating Mechanism Secretariat Office, Bangkok, Thailand, ^5^Service Workers in Group Foundation, Bangkok, Thailand, ^6^Rainbow Sky Association of Thailand, Bangkok, Thailand, ^7^Mplus Foundation, Chiang Mai, Thailand, ^8^U.S. Agency for International Development ^USAID^ Community Partnership, Thai Red Cross AIDS Research Centre, Prevention, Bangkok, Thailand, ^9^Thai Red Cross AIDS Research Centre, Prevention, Bangkok, Thailand, ^10^FHI 360 and USAID LINKAGES Project, Bangkok, Thailand, ^11^Office of Public Health, USAID Regional Development Mission Asia, Bangkok, Thailand

**Background: **The key population‐led health services (KPLHS) model has been proven to be efficient, safe, and feasible in enhancing uptake of and retention in HIV testing, antiretroviral treatment, and pre‐exposure prophylaxis (PrEP) services among men who have sex with men, transgender women, and male and transgender sex workers in Thailand. Lay providers who are members of these key populations (KPs) delivered 42 percent of HIV testing, 35 percent of HIV diagnosis, and more than 50 percent of PrEP services among men who have sex with men and transgender women in the country in 2016. For KPLHS scale‐up and sustainability, policy commitment must be achieved to ensure legalization and domestic financing mechanisms.

**Methods: **A series of high‐level policy and advocacy dialogues were held with Ministry of Public Health stakeholders during 2017 and 2018. The Thai Red Cross AIDS Research Centre and community‐based organizations (CBOs), supported by LINKAGES Thailand, provided feasibility, impact, and health economic data on KPLHS to assist policy decisions. The USAID Community Partnership established a training and certification platform and facilitated regulatory reform to allow KP lay providers to deliver high‐quality HIV and sexually transmitted infection services.

**Results: **A Ministry of Public Health decree was revised to allow trained KP lay providers to perform HIV counselling, specimen collections for HIV and sexually transmitted infections, and rapid/point‐of‐care tests, as well as antiretroviral treatment and PrEP dispensing. KPLHS standards, training modules, and certification steps are in the process of being endorsed by the National AIDS Committee and the Ministry of Public Health. Domestic financing mechanisms are being piloted for the National Health Security Office to directly fund CBOs for KPLHS.

**Conclusions: **Concerted effort among key policy stakeholders, academia, and CBOs, together with strong leadership by the Ministry of Public Health, did efficiently advance regulatory reform to legalize KP lay providers and facilitate piloting to transition from international aid to domestic funding for sustainability of KPLHS.

## MOAD0105

### Barriers and facilitators to the successful transition of adolescents living with HIV from paediatric to adult care in low and middle‐income countries: A policy review

**T. Ritchwood^1^; C. Jones^2^ and T. Taggart^3^**

^1^Duke University, Durham, United States, ^2^Medical University of South Carolina, Charleston, United States, ^3^George Washington University, D.C., United States

**Background: **Adolescents living with HIV face the unique challenge undergoing healthcare transition, which occurs when they age out of paediatric HIV care and engage in a planned transfer to an adult care setting. This process often coincides with a developmental period during which many adolescents struggle with disease management and experience the onset of HIV‐related complications and interruptions in care, leading to high rates of HIV‐related morbidity and mortality. While there have been a number of published literature reviews focused on healthcare transition in North America and Europe, we lack reviews evaluating healthcare transition in low‐middle‐income countries. Therefore, this study systematically reviews the literature on transition‐related barriers and facilitators in low‐middle‐income countries. Additionally, we review relevant, country‐specific policies to determine whether they are responsive to the barriers and facilitators of successful transition.

**Methods: **We conducted a systematic literature review using the online databases to identify articles in peer‐reviewed journals that included text associated with HIV, adolescents, and healthcare transition. We identified country‐specific guidelines by searching the websites of each country's health ministry, international publications, and relevant online databases.

**Results: **Our review yielded 10 studies assessing barriers and facilitators of transition for adolescents residing in low‐middle‐income countries. Our review of country‐specific guidelines revealed that few countries have guidelines specific to transition for adolescents. We identified three factors critical to advancing the healthcare transition literature in low‐middle‐income countries: (1) more rigorous studies examining the effectiveness of transition programmes, (2) the development and implementation of targeted guidelines or policies that address barriers and facilitators of transition among adolescents, and (3) the development of transition‐oriented programmes that address the socio‐structural factors that affect transition, such as HIV‐related stigma and socioeconomic factors.

**Conclusions: **Our review has led to several recommendations to facilitate successful transition, including:

1. training for the adult treatment team prior to transition;
2. dedicated time to treat adolescent patients or employment of adherence clubs; and
3. comprehensive programmes that consider the developmental and cognitive needs of adolescents transitioning to adult care settings and children transitioning to adolescent care settings are also needed.

## MOAD0201

### Private sector HIV self‐testing in Kenya: Insights from a Mystery shopper study

**K. Little^1^; C. Odour^2^; H. Awsumb^1^ and H. Essendi^2^**

^1^Population Services International, HIV/TB, Washington, United States, ^2^Population Services Kenya, Nairobi, Kenya

**Background: **To understand the private sector's ability to reach adolescents and young people with high quality HIV self‐testing (HIVST) services, we conducted a mystery shopper study at pharmacies and private health facilities participating in an HIVST demonstration project in Nairobi and Mombasa.

**Methods: **Outlets were randomly selected for the study. Facility owners provided verbal consent, but were not informed about the date or time of visits. Mystery shoppers ages 18 to 30 visited facilities and attempted to purchase a quality‐assured HIVST kit using one of 14 pre‐defined mystery shopper scenarios. Scenarios included instructions for the shopper's age (range: 16 to 24 years), reason for testing, and type of kit to be purchased, and questions to ask the provider. After the visit, shoppers were interviewed about their experiences using a structured guide administered by a trained surveyor.

**Results: **In Sep. 2018, 28 mystery shoppers visited 14 private health facilities and 41 pharmacies. While the project set the consumer price for both HIVST kits at 500Ksh, 6 (11%) facilities sold them at higher prices. Observed prices ranged from 150Ksh‐900Ksh. Most facilities (25, 45%) stored kits in a storage room accessible only to employees, or under/behind the counter (10, 18%). Complete stock‐outs weren´t uncommon (7/55 facilities), and a further 5 facilities had just a single brand of quality‐assured HIVST in stock at the time of the visit. While 42/55 providers gave clients information to help them use the test kit, and 36 provided a step‐by‐step explanation on using the kit, slightly fewer (27, 49%) could answer specific test‐use questions satisfactorily. Misinformation, unfriendliness, lack of privacy, and provider preferences for one test kit over the other were also reported.

**Conclusions: **Despite a one‐time training and monthly medical detailing visits, HIVST service delivery quality was uneven. Kit prices ranged substantially, as did the ability/willingness of providers to answer questions or demonstrate the use of the kits. Because kits were not stocked on shelves, providers acted as an important mediator for consumers deciding on which test kit to purchase. Further supportive supervision efforts may be needed to ensure providers are able to support consumers in safely using and interpreting the results of HIVST kits.

## MOAD0202

### False negative HIV rapid test results among people living with HIV on antiretroviral therapy in Johannesburg, South Africa: Implications for HIV self‐testing roll out

**M. Majam^1,2^; J.M. Francis^1,2,3^; N. Rhagnath^1^; V. Msolomba^1^ and F. Venter^1,2^**

^1^University of the Witwatersrand, Wits Reproductive Health and HIV Institute ^Wits RHI^, Johannesburg, South Africa, ^2^University of the Witwatersrand, Faculty of Health Sciences, School of Clinical Medicine, Johannesburg, South Africa, ^3^Muhimbili University of Health and Allied Science, Epidemiology and Biostatistics, Dar es Salaam, Tanzania, United Republic of Tanzania

**Background: **The World Health Organization (WHO) self‐testing guidelines recommends that people living with HIV (PLHIV) on antiretroviral therapy (ART) refrain from performing self‐tests due to the risk of obtaining false‐negative results. We conducted a pilot study to assess the accuracy of one oral fluid and five blood based rapid diagnostic test (RDT) kits among PLHIV, of which four are designed for self‐testing.

**Methods: **This was a cross sectional study among PLHIV on ART participating in two randomized clinical trials within the Wits Reproductive Health and HIV Institute treatment optimization programme, in Johannesburg, South Africa. Participants were recruited using convenience sampling. All participants had been on ART for a minimum of 2 years. The research nurses performed the RDT serially, starting with the oral followed by the blood based. Two nurses blinded to the test kits read and interpreted the results. We assessed the agreement between the readers using the *kappa* statistic, computed the proportion of positive and negative test results by age, sex and duration on ART, and assessed the association using the Fisher's exact test.

**Results: **100 participants were recruited into the study; 67% of whom were females. Majority of the participants were ≤40 years (53%) and 41% had been on ART for ≥7 years. Overall, the two nurses had high agreement on the results reading with *kappa* ranging from 90 ‐ 100% (*p*< 0.001). Nine (9%) of the patients had false negative results on at least one of the RDTs with a total of 16 false results in the 600 tests performed. Four participants had multiple tests with false results. False negative results was not associated by duration on treatment.

**Conclusions: **False negative results have serious implications for HIV Self‐Testing Programmes. Retesting on ART could result in participants believing that they are ‘cured’, especially in instances where multiple tests appear negative. False negativity may not be associated with length on time on ART, however further investigation on time to ART initiation from infection may play a role in antibody production.

## MOAD0203

### Reaching the unreachable: early results from index testing in Zambia in the CIRKUITS project

**L.K. Mwango^1^; M. Mujansi^1^; J. Chipukuma^1^; B. Phiri^1^; H. Sakala^1^; N. Nyirongo^1^; S. Sivile^1^; M. Sinjani^1^; M.‐C. Lavoie^2^ and C.W. Claassen^2^**

^1^Maryland Global Initiatives Corporation, Lusaka, Zambia, ^2^University of Maryland School of Medicine, Center for International Health, Education, and Biosecurity ^CIHEB^, Lusaka, Zambia

**Background: **In Zambia, men, adolescent girls and young women (AGYW), and adolescent boys and young men (ABYM) are hard‐to‐reach priority populations (PPs) yet contribute significantly to gaps in achieving HIV epidemic control. Novel testing strategies are needed to identify HIV‐infected PP individuals in the community while maximizing positivity yield. We present data from the Community Impact to Reach Key and Underserved Individuals for Treatment and Support (CIRKUITS) project on index and social network testing. CIRKUITS is a PEPFAR‐funded project employing community approaches to accelerate HIV epidemic control among key and priority populations.

**Methods: **We analysed age and sex‐disaggregated programme data from Zambian Ministry of Health HIV testing services and index testing registers. We included data from October to December 2018 across 41 CIRKUITS‐supported facilities in Eastern, Western and Lusaka provinces. Since October 2018, CIRKUITS has trained, mentored, and deployed 124 community health workers (CHWs) and 21 community liaison officers to conduct index and social network testing, partner notification services, and PP hotspot mapping in all supported facilities.

**Results: **CIRKUITS CHWs tested 12,250 clients in the community, of whom 1809 (15%) were HIV‐positive. Among HIV‐positive clients, 1569 (87%) clients were indexed, with 2753 contacts elicited which included sexual partners and biological children (elicitation ratio: 1:1.8). Of the 2602 contacts followed up, 2073 contacts presented with unknown status and were tested for HIV; of these, 668 were newly diagnosed as HIV‐positive, representing 32% positivity yield. Of these, 230 (34%) were women older than 25 years, 211 (32%) were men older than 25 years, 89 (13%) were AGYW ages 10 to 24, 85 (13%) were ABYM ages 10 to 24, and 53 (8%) were children under the age of 10 years.

**Conclusions: **Index and social network testing are effective strategies to identify HIV‐infected persons in Zambia, especially priority populations that are hard to reach such as men, AGYW, and ABYM.

## MOAD0204

### “Once their wives are OK … they have no disease:”men infer HIV status from partner's results: qualitative insights into male HIV testing in rural Malawi

**A. Radunsky^1^; J. Weinstein^2^; E. Geoffroy^3^; R. Atun^1^; M.C.S. Fawsi^4^; M. McConnell^1^ and T. Barnighausen^1,5^**

^1^Harvard T.H. Chan School of Public Health, Global Health and Population, Boston, United States, ^2^Boston University, Boston, United States, ^3^Global AIDS Interfaith Alliance, San Francisco, United States, ^4^Harvard Medical School, Global Health and Social Medicine, Boston, United States, ^5^University of Heidelberg, Institute of Global Health, Heidelberg, Germany

**Background: **Even with available HIV testing and effective treatment, HIV testing rates for men in rural Malawi and across sub‐Saharan Africa remain low. We aim to add to the theoretical understanding for how and why men avoid HIV testing and how they make sense of this choice in HIV endemic settings. We use an ecological framework that considers institutional structures, social pressures and internal assessments of risk that strongly shape men's testing behaviours.

**Methods: **We conducted 30 semi‐structured in‐depth qualitative interviews with adult men ages 20 to 39, in Mulanje District of rural Malawi in 2014. Audio‐recordings were coded and analysed using a Modified Grounded Theory framework.

**Results: **Findings indicate that men rationalize their non‐testing, especially when they can use their spouses’ HIV negative test result as a proxy for their own status. Men identify testing as a source of anticipated stigma, social risk and potential family instability. Men resist testing even while encouraging their spouses to do so. Women are further encouraged to test in community and health service settings. Because of gendered social power dynamics, when women are encouraged to test they more often comply. If her test is negative, he can use that test result as a proxy for his own status. Her negative result reassures him that testing is unnecessary, and he can successfully rationalize going untested. Although unmotivated by their own health, men are more likely to test in response to an acute medical need.

**Conclusions: **Societal gender norms empower and motivate men to avoid testing, and institutional structures enable men to shift the social risk of an HIV positive result to women. Men highly value their social status, family structure and sexual access and HIV testing threatens to destabilize all of these. Men are sensitive to appeals to test that relate to helping their family, but if their wife is known to be HIV negative, this argument is less compelling. PMTCT related partner testing is an opportunity to draw men in, especially if men appreciate sex with their wife as a potential HIV risk to their unborn child.

## MOAD0205

### Community mobilization is associated with HIV testing, particularly among men and rural dwellers, in Zambia

**J.G. Rosen^1^; M.A. Carrasco^2^; B. Olapeju^3^ and E.K. Kumoji^3^**

^1^Population Council, Lusaka, Zambia, ^2^United States Agency for International Development, Office of HIV/AIDS, Washington, United States, ^3^Johns Hopkins Center for Communications Programs, Baltimore, United States

**Background: **Engaging men in HIV testing is a priority to reaching UNAIDS 90‐90‐90 goals in sub‐Saharan Africa, where men continue to access testing less than women. Using data from a population‐based assessment, this study aimed to measure the association between community mobilization and HIV testing in Zambia to determine if community mobilization could be a viable strategy to engage men in testing.

**Methods: **Data come from a nationally representative survey of individuals aged 15 to 59 in 14 districts across Zambia's 10 provinces. Two‐stage sampling proportional to population size was used to select households across residence types (urban/rural) in enumeration areas. The primary independent variable was operationalized using an 18‐item, 5‐point scale assessing four dimensions of community mobilization: community participation, social cohesion, collective efficacy, and leadership. Other independent covariates of interest included socio‐demographic factors and other HIV‐related factors, including sexual risk‐taking and HIV stigma. Bivariate and multivariable logistic regression was used to measure the association between HIV testing and community mobilization. Multivariable models were subsequently stratified by sex and residence. Analytic weights derived from 2010 Zambian census data and 2016 population projections adjusted for clustering and stratification.

**Results: **Among 3533 respondents, 83% (n=2921) reported previous HIV testing, with female (87% vs. 82%, *p*< 0.001) and married (93% vs. 68%, *p*< 0.001) respondents reporting HIV testing at significantly higher proportions than male and never‐married respondents, respectively. Adolescents (aged 15 to 24 years) reporting HIV testing at significantly lower proportions than respondents aged 25 to 34 years (73% vs. 94%, *p*< 0.001) and 35 to 44 years (73% vs. 95%, *p*< 0.001). In multivariable analysis, community mobilization (mean: 62.48, range: 30 to 90) was significantly associated with HIV testing (AOR=1.02, CI: 1.00 to 1.03). In sex‐ and residence‐stratified models, the odds of HIV testing were higher and significant among males (AOR=1.02, CI: 1.01 to 1.04) and rural residents (AOR=1.03, CI: 1.01 to 1.06), but not among females or urban residents, for each additional unit increase in community mobilization.

**Conclusions: **Community mobilization emerged as a significant factor associated with HIV testing among survey respondents, particularly among males and rural dwellers. These findings contribute to a growing toolkit of layered interventions for promoting HIV testing, particularly for hard‐to‐reach, vulnerable men and rural dwellers.

## MOAD0301

### PrEP knowledge, intention and uptake among MSM geosocial networking app users in Mexico

**I. Holloway^1^; A. Garner^2^; J. Lai^3^; A. Ritterbusch^3,4^; S. Giraldo^5^ and V. Guilamo‐Ramos^6^**

^1^University of California, Los Angeles, Department of Social Welfare, Los Angeles, United States, ^2^Hornet Network Limited, West Hollywood, United States, ^3^UCLA, Department of Social Welfare, Los Angeles, United States, ^4^Universidad de Los Andes, Bogota, Colombia, ^5^Universidad de Los Andes, Bogotá, Colombia, ^6^New York University, Silver School of Social Work, New York, United States

**Background: **Pre‐exposure prophylaxis (PrEP) is a biomedical prevention strategy with significant potential to reduce HIV incidence among MSM worldwide. In 2018, the ImPrEP Project began in Mexico, a preliminary step for the launch of comprehensive PrEP services in that country. However, little data exists about PrEP engagement among MSM in Mexico. We sought to understand correlates of PrEP knowledge, intention, and uptake among an online sample of MSM in Mexico in order to guide future PrEP intervention efforts.

**Methods: **Between November 2018 and January 2019 we recruited MSM from the social networking application, Hornet, using targeted advertising (N=2957). Participants completed a brief online survey, which assessed PrEP knowledge, intention to use PrEP in the next 6 months, and current PrEP use. Bivariate tests of association, followed by multivariable logistic regression analyses, were used to determine correlates of these three PrEP‐related outcomes, sociodemographic characteristics and behavioural health variables.

**Results: **HIV‐positive MSM (N=490; 16.6%) were excluded, leaving a total analytic sample of 2467. Over two‐thirds (70.0%) had previous knowledge about PrEP; 35.7% intended to use PrEP in the next 6 months; and 3.48% were current PrEP users. Of those currently using PrEP, the majority obtained the medication from their doctors (51.1%); a smaller percentage obtained the medication from a research study (25.0%), friends (7.9%), the internet (11.3%) or another source (4.5%). Mutivariable analyses demonstrated that PrEP use was associated with prior sexually transmitted infection testing (aOR: 6.67, CI: 4.12 to 10.77), and satisfaction with sex life (aOR: 1.37 , CI: 1.16 to 1.60 ). PrEP intention was associated with using the “know your status” function on Hornet (aOR: 2.17, CI: 1.76 to 2.68), and sex life satisfaction (aOR: 1.19, CI: 1.05 to 1.33).

**Conclusions: **PrEP use among Mexican MSM in our sample was low; however, knowledge of and intention to use PrEP were high, representing an important opportunity for HIV prevention. Geosocial networking apps and STI testing centres may be leveraged for PrEP information dissemination and PrEP access among MSM in Mexico. PrEP users in our sample were more satisfied with their sex lives than non‐PrEP users; highlighting this may be helpful to increasing PrEP uptake among Mexican MSM.

## MOAD0302

### Manifestations of stigma in the context of a national oral pre‐exposure prophylaxis (PrEP) scale‐up programme in Kenya

**D. Were^1^; K. Atkins^2^; A. Musau^1^; M. Plotkin^3^ and K. Curran^3^**

^1^Jhpiego Kenya, Nairobi, Kenya, ^2^Johns Hopkins Bloomberg School of Public Health, Baltimore, United States, ^3^Jhpiego, Affiliate of Johns Hopkins University, Baltimore, United States

**Background: **Since Kenya began scaling up oral PrEP in May 2017, uptake has been slower than expected among adolescent girls and young women (AGYW), and overall continuation rates consistently low. Stigma was documented as a substantial barrier during clinical trials, hence understanding how PrEP‐related stigma is experienced in routine service delivery is critical to improve PrEP outcomes. The Jilinde project, funded by the Bill & Melinda Gates Foundation, provides PrEP to female sex workers (FSW), men who have sex with men (MSM) and AGYW. This abstract describes how stigma is manifested and its impact on PrEP uptake and continuation.

**Methods: **Between October 2017 and November 2018, qualitative data were collected from 222 respondents via 22 focus group discussions (FGD) and 30 in‐depth interviews: 86 AGYW, 12 parents of AGYW, 10 male partners of AGYW, 36 MSM, 28 FSW, 29 health care providers, and 20 peer educators. All interviews and FGDs were audio‐recorded, transcribed and translated, then thematically analysed.

**Results: **Stigma negatively influenced PrEP uptake and continuation among AGYW, FSW and MSM, who self‐stigmatized and were stigmatized by others. Stigma was manifested through stereotypes, prejudice, and discrimination by peers, sexual partners, family, healthcare providers and the community. For MSM and FSW, PrEP‐related stigma was intertwined with identity stigma, while for AGYW it was manifested through stigma toward sexual behaviour. Some health providers equated giving PrEP to these populations to promoting immorality. All individuals disguised their PrEP use since it was associated with ‘recklessness’. PrEP users were labelled as promiscuous and subjected to similar stigma associated with being HIV‐positive. Consequently, PrEP use was hampered by: fear of violence and rejection by an intimate partner, family or community members; discrimination by providers; loss of ‘business’; reputational damage; and shame.

**Conclusions: **Stigma remains a critical barrier to PrEP use among priority communities in Kenya and was directed towards the product, clients’ behaviour and identity. While stigma was manifested differently for diverse populations, results were similar in terms of PrEP uptake and continuation. Health care providers and communities should be prioritized in stigma interventions to improve uptake and optimize the outcomes of PrEP.

## MOAD0303

### Results from a large Australian PrEP demonstration study: Discontinuation and subsequent HIV and other sexually transmitted infection risk

**K. Ryan^1,2^; J. Asselin^3^; C. Fairley^4,5^; L. Lal^13^; L. Nguyen^2^; M. Penn^6^; B. Price^1^; N. Roth^7^; S. Ruth^8^; B.K. Tee^9^; M. West^10^; J. Willcox^11^; M. Hellard^1,2^; J. Hoy^1^; M. Stoove^2^; E. Wright^1,2^ and PrEPX Study Group**

^1^Alfred Health and Monash University, Department of Infectious Diseases, Melbourne, Australia, ^2^Burnet Institute, Public Health Discipline, Melbourne, Australia, ^3^Burnet Institute, Melbourne, Australia, ^4^Monash University, Central Clinical School, Melbourne, Australia, ^5^Melbourrne Sexual Health Centre, Melbourne, Australia, ^6^Thorne Harbour Health, PRONTO!, Melbourne, Australia, ^7^Prahran Market Clinic, Melbourne, Australia, ^8^Thorne Harbour Health, Melbourne, Australia, ^9^The Centre Clinic, Melbourne, Australia, ^10^Victorian Government, Department of Health and Human Services, Melbourne, Australia, ^11^Northside Clinic, Melbourne, Australia

**Background: **The PrEPX demonstration study used existing health services to emulate the ‘real world’ provision of HIV PrEP prior to government subsidisation in Australia in April 2018. We describe PrEPX participants who discontinued receiving study drug prior to the study ending, examine factors associated with discontinuation and describe these participants’ ongoing HIV and sexually transmitted infection (STI) risk.

**Methods: **Study drug dispensing data from pharmacy logs, HIV/STI testing and behavioural survey data from four study clinics participating in the Australian Collaboration for Coordinated Enhanced Sentinel Surveillance (ACCESS) system were extracted for the duration of PrEPX (26 July 2016 to 30 April 2018). PrEPX participants were provided 90 pills per study drug dispensing event, and study discontinuation was classified as participants who were dispensed their last study drug before October 2017, seven months prior to study completion and missing at least two scheduled study prescriptions. Cox proportional hazards estimated covariates associated with discontinued study participation. HIV/STI diagnosis rates >100 days after last study drug dispensed are described and differences in HIV/STI positivity between study and post‐study periods were assessed using Chi squared analyses.

**Results: **This analysis included 2451 participants; 515 (21.0%) discontinued study participation with a median time from last study drug dispensed to study end of 367 days (IQR:272 to 499). PrEP naiveté (aHR1.67 95%CI: 1.11 to 2.48), age < 30 years (aHR1.65, 95%CI: 1.09 to 2.50), and reporting consistent condom use with casual partners (aHR1.52 95%CI: 1.01 to 2.30) at enrolment were associated with discontinuing study participation. Of these 515 participants, 130 (25.2%) accessed post‐study testing at ACCESS sites; four participants (3.3%) were diagnosed with HIV during the observation period. Mean time between last study drug dispensed and HIV diagnosis was 338 days (range 140 to 466 days). STI positivity was similar between pre and post‐study periods for chlamydia (8.5%, 8.3%, *p*=0.9), gonorrhoea (10.4%, 9.9%, *p*=0.9) and syphilis (0.5%, 1.3%, *p*=0.5).

**Conclusions: **Approximately 20% of participants in this analysis discontinued study participation. Four HIV diagnoses and similar STI positivity between study and post‐study periods suggest ongoing HIV and STI acquisition risk and unmet HIV prevention need. Greater understanding of barriers to PrEP retention and factors affecting accurate risk perception are needed to maximise the HIV prevention benefits of PrEP.

## MOAD0304

### Predictors of discontinuation in Brazil's free‐of‐charge PrEP programme

**I. Ornelas Pereira; A.R.P. Pascom; F. de Barros Perini; C. Habckost Dutra de Barros; G. Mosimann Júnior; A. Schwartz Benzaken and G.F. Mendes Pereira**

Ministry of Health of Brazil, Department of Surveillance, Prevention and Control of STIs, HIV/AIDS and Viral Hepatitis, Brasília, Brazil

**Background: **Retention in care is a major challenge in HIV pre‐exposure prophylaxis (PrEP) implementation programmes. PrEP has been offered free of charge in the Brazilian public health system since December 2017. We aimed to describe the profile of discontinued PrEP users as well as the rates and predictors of discontinuation before the first follow‐up visit in the Brazilian PrEP programme.

**Methods: **We used secondary data from the Ministry of Health of Brazil (MoH‐B), including individuals admitted in the national daily dosing PrEP programme, between January and December 2018. Discontinuity was defined as failing to attend the follow‐up visit, with a delay (in days) of more than 40% of the expected time difference between the first consultation and the scheduled follow‐up visit. Multivariable logistic regression model was used to assess the likelihood of PrEP discontinuation considering demographic and behavioural predictors.

Abstract MOAD0304‐Figure 1. Multivariable logistic regression model results for interrupting PrEP before first follow‐up visit.

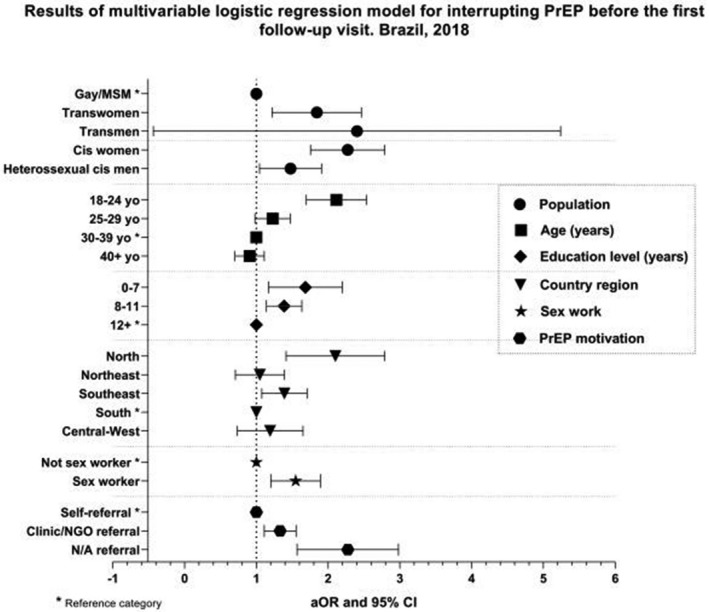

**Results: **Among the 8097 enrolled PrEP users, 821 (10%) did not attend their first follow‐up visit. Median age of discontinued users was 29 years old (IQR 24 to 36). Young users (18 to 24 years old) were 109% more likely to discontinue PrEP (aOR 2.087, 95%CI: 1.710 to 2.548) and sex workers were 52% more likely to do so (aOR 1.522, 95%CI: 1.215 to 1.905). Compared to MSM, the odds of discontinuing PrEP was 2.233 (95%CI: 1.776 to 2.807) among cis women, and 1.772 (95%CI: 1.258 to 2.497) among transwomen. Other factors positively associated with PrEP discontinuation were living in Brazil's North or Southeast region, compared to the South; lower education level; and clinic or NGO referral, rather than self‐referral.

**Conclusions: **Understanding characteristics of users who are most‐likely to discontinue PrEP is crucial to help health services to deliver strategies that are tailored to specific barriers to care. Enhancing education, motivation and social/psychological support during early PrEP visits may increase continuation in care and strengthen PrEP as public health policy.

## MOAD0305

### A competing risks model for the use of condom in the open‐label extension of the ANRS Ipergay study

**L. Sagaon Teyssier^1,2^; M. Mimi^1,2^; D. Rojas Castro^3,4^; N. Hall^5^; C. Capitant^6^; L. Meyer^6,7^; C. Chidiac^8^; C. Tremblay^9^; G. Pialoux^10^; C. Pintado^11^; M. Préau^12^; J.‐M. Molina^13^; B. Spire^1,2^ and ANRS IPERGAY Study Group**

^1^SESSTIM UMR 1252, Marseille, France, ^2^ORS PACA, Observatoire Régional de la Santé Provence‐Alpes‐Côte d'Azur, Marseille, France, ^3^AIDES, Paris, France, ^4^Coalition PLUS, Community‐based Research Laboratory, Paris, France, ^5^CHU de Nantes, Department of Infectious Diseases, Nantes, France, ^6^INSERM SC10 US19, Villejuif, France, ^7^Université Paris Sud, Paris Saclay, France, ^8^Hôpital de la Croix Rousse, INSERM U1052, Department of Infectious Diseases, Lyon, France, ^9^Research Center of the Centre Hospitalier de l'Université de Montréal, Montréal, Canada, ^10^Hôpital Tenon, Assistance Publique Hôpitaux de Paris, Maladies Infectieuses, Paris, France, ^11^Hospital Saint‐Louis, Assistance Publique Hôpitaux de Paris, Department of Infectious diseases, Paris, France, ^12^Groupe de Recherche en Psychologie Sociale ^EA 4163^, Université Lumière Lyon 2, Université de Lyon, Lyon, France, ^13^Hospital Saint‐Louis, Assistance Publique Hôpitaux de Paris, Department of Infectious Diseases, Paris, France

**Background: **During the open‐label extension (OLE) phase of the ANRS Ipergay trial, the overall use of condoms decreased when participants knew about the PrEP efficacy with TFDF/FTC vs. placebo. Still some participants resumed condom use during OLE. We aimed investigating the factors associated with using condoms back again combined or not with PrEP.

**Methods: **ANRS Ipergay OLE started in November 2014. Follow‐up between M0‐M18 included bimonthly HIV/STI testing and online questionnaires collecting sexual behaviour, PrEP (correct/sub‐optimal) and condom use at the most recent anal intercourse (MRAI). This analysis focused on participants using only PrEP at OLE enrolment. The outcome: time elapsed until condom resumption alone or combined with PrEP. Competing risks survival model estimated hazard‐ratios (HR). Individual characteristics, sexual behaviour and STI onset before condom resumption during OLE were specified as time‐dependent variables.

**Results: **Among 361 OLE participants, 146 (40.4%) used only PrEP for their MRAI at enrolment. Compared to other participants, those using only PrEP were less educated (63.3% vs 36.7%, *p*=0.03), and had higher HIV‐risk perception (scale 0 to 10; median[IQR]:4[2 to 7] versus 2[1 to 4], *p*< 0.001). No difference was found for age, number of sexual partners and number of sexual intercourses. 70% of these 146 participants were low‐level condom users during the double‐blind phase with: on average, 2/10 MRAI with condom (6/10 for the remaining 30%). Among these 146 participants, 8.2% resumed condoms instead of PrEP on average after 5.5 months (sd.±3.4); and 59.6% PrEP+condoms on average after 6 months (sd.±4.4). On average, condoms resumption lasted 7.6 months (sd.±6.1). Condom use probability increased with the number of sexual partners (previous 2 months) (HR:1.01, *p*=0.035); and the number of sexual intercourses (previous 4 weeks) (HR:1.05, *p*< 0.001). Sensations seeking increased condom resumption probability in combination with PrEP (HR:1.04, *p*=0.038). However, chemsex reduced condom resumption alone (HR:0.157, *p*=0.014) or combined with PrEP (HR:0.712, *p*=0.003). Finally, the onset of STIs during OLE increased condom resumption probability alone (HR:3.69, *p*< 0.001) and combined with PrEP (HR:1.42, *p*=0.013);

**Conclusions: **Condom resumption during the OLE phase of the ANRS Ipergay study was strongly associated to the onset of STIs. In contrast, chemsexers were less likely to resume condom while using PrEP.

## MOAD0401

### Are community‐led organizations the drivers for enhancing financial security among female sex workers? Lessons from a large‐scale HIV intervention in India

**S.K. Patel; S. Mukherjee; B. Mahapatra; M. Battala and N. Saggurti**

Population Council, HIV and AIDS Program, New Delhi, India

**Background: **Community‐led organizations have been essential part of HIV prevention programmes to address the socio‐economic and structural vulnerabilities among female sex workers (FSWs). The current study aims to examine whether strengthening of community organizations (COs) have been instrumental in reducing the financial vulnerability among FSWs in India.

**Methods: **This study used a panel data of 2085 FSWs selected from 30 COs across five states of India. Two rounds of data (baseline in 2015 and end line in 2017) were collected among FSWs. Data were collected both at CO and individual level. CO level data was used to assess the CO strength. Individual level data was used to measure financial security. The financial security measure was measured based on the composite score of having a savings account, investment in schemes, insurance products, had an alternative source of income, and had not taken any loan from informal sources. Descriptive statistics, frequency, bivariate and multilevel logistic regression techniques were used for the analysis.

**Results: **There was a significant improvement in CO strength from baseline to end‐line. High CO strength has led to improved financial security among FSWs (EL: 85% vs. BL: 51%, AOR: 2.5; 95% CI: 1.5 to 4.1) in end line from baseline. FSWs those associated with COs whom were formed more than 5 years ago, have higher financial security compared to others (EL: 86% vs. BL: 49%, AOR: 2.82). In addition, FSWs those belonged to larger outreach COs (covering >=1200 FSWs) have 2 times higher financial security compared to FSWs belonged to smaller outreach COs (covering < 1200 FSWs). Further, the improvement in financial security in the inter‐survey period led to increased or sustained individual empowerment (in terms of self‐confidence, self‐efficacy and individual agency) among FSWs.

**Conclusions: **The study concluded that organizational strengthening under the community mobilization interventions are key to address the structural issues and the decrease of financial vulnerability among FSWs. Further attention is needed to sustain the existing community advocacy and engagement systems to address the vulnerabilities faced by marginalized populations and build their empowerment.

## MOAD0402

### Organizational and individual‐level strategies associated with viral suppression in a sample of transgender women receiving care for HIV infection in the U.S

**G. Rebchook^1^; J. Keatley^1^; S. Shade^1^; A. Maiorana^1^; J. Xavier^2^ and SPNS Transgender Women of Color Study Group^2^**

^1^UCSF, San Francisco, United States, ^2^Independent Consultant, Silver Spring, United States

**Background: **With increasing evidence of high HIV infection rates among transgender women (TW) worldwide, it is critical to improve their engagement in HIV primary care to achieve viral suppression. However, TW encounter numerous difficulties accessing healthcare, including stigma, provider bias, and a higher priority of seeking transgender‐related care, where available.

**Methods: **The U.S. Department of Health and Human Services, through HRSA's Special Projects of National Significance, funded nine demonstration sites to implement innovative interventions to engage TW living with HIV into care. The University of California, San Francisco Evaluation Center conducted key informant interviews to characterize organization‐level strategies associated with successful interventions. Demonstration sites submitted information on individual‐level intervention activities (screening, referrals, and services) provided to intervention participants and medical chart data on linkage, treatment, retention, and viral suppression (< 200 copies/mL) before and after intervention participation. We employed generalized estimating equations to identify intervention activities significantly associated with change in viral suppression at 24 months.

**Results: **Sites enrolled 858 TW into nine interventions, with 79% participating in intervention activities. TW participated in a median of 280 minutes (IQR=45 to 630 minutes) of activities. Viral suppression increased from 23% at baseline to 35% at 24 months. Common organization‐level strategies included: transgender empowering environments and activities; TW in visible staff/mentoring roles; support for self‐care of staff; and incentives to attend intervention activities and/or health services. Individual‐level intervention activities associated with change in viral suppression at 24 months include: (1) screenings for: mental health diagnosis (aOR=4.15; 95% CI=1.23 to 13.95), substance abuse (aOR=0.41; 95% CI=0.26 to 0.65) or food insecurity (aOR=2.87; 95% CI=1.82 to 4.53); (2) referrals to: HIV primary care (aOR=2.22; 95% CI=1.17 to 4.20), mental health care (aOR=2.52; 95% CI=1.06 to 5.99) or food assistance (aOR=2.43; 95% CI=1.42 to 4.16); and (3) services: retention counselling (3.50; 95% CI=2.11 to 5.81) and employment (aOR=6.18; 95% CI=2.93 to 13.05).

**Conclusions: **Transgender‐affirming care settings with TW staff and integration of HIV primary care with mental health screening and referrals; food insecurity screenings and services; HIV care referrals; and retention counselling and employment services were associated with increased viral suppression. The continued development, adaptation and scale up of integrated care interventions for this key population will be necessary to meet 90‐90‐90 goals.

## MOAD0403

### Dreams of an AIDS‐free generation: Which supportive factors delay onset of adolescent sexual HIV‐risk behaviours in South Africa

**E. Toska^1,2^; L. Campeau^3^; L.D. Cluver^1,2^; F.M. Orkin^2^; M. Berezin^2^; L. Sherr^4^ and G. Bachman^5^**

^1^University of Cape Town, Cape Town, South Africa, ^2^University of Oxford, Social Policy and Intervention, Oxford, United Kingdom, ^3^Oxford Research South Africa, East London, South Africa, ^4^University College London, Research Department of Global Health, London, United Kingdom, ^5^PEPFAR, USAID, Orphans and Vulnerable Children, Washington, D.C., United States

**Background: **Early initiation of sexual risk‐taking during adolescence is strongly associated with HIV infection. However, little is known about which supportive factors may delay and prevent high‐risk sexual practices, especially in younger adolescents.

**Methods: **We pooled data from two longitudinal surveys in three South African provinces to identify adolescents who had not initiated high HIV‐risk behaviours (n=3662). Interviews were conducted by trained researchers following informed, voluntary consent from adolescents and their caregivers. Questionnaires were designed as teen‐friendly magazines to maximize participation. Initiation of HIV‐risk behaviours was defined as initiating one of four high‐risk sexual practices between baseline and 12‐month follow‐up (incident high‐risk sex). These practices included inconsistent condom use, transactional sex, age‐disparate relationship, or early sexual debut. Analyses used multivariate logistic regression to test whether consistent access to eight structural provisions was associated with incident high‐risk sex.

**Results: **Adolescents were young (baseline mean age 12.8 years), 57% female, and 42% lived in rural areas. Between baseline and follow‐up, 8.7% of adolescents reported incident HIV‐risk behaviours, which was strongly associated with incident pregnancy. Consistent access to four structural provisions was strongly associated with delayed incident high‐risk sex: parenting/caregiving support (OR=0.53 95%CI0.35 to 0.80 *p*=0.002), violence prevention (OR=0.54 95%CI0.37 to 0.79 *p*=0.002), school subsidies (OR=0.57 95%CI0.35 to 0.93 *p*=0.002), and HIV knowledge (OR=0.43, 95%CI0.21 to 0.89 *p*=0.023), alone and in combination. One‐year incident high‐risk sex was 1.1% with access to all four structural provisions compared to 12.4% without access to any provisions. Gender moderated the effect of school meals on incident high‐risk sex: boys who accessed school meals consistently reported the greatest reduction in incident high‐risk sex compared to boys who did not (Fig 1).

Abstract MOAD0403‐Figure 1. Effects of school meals on incident high‐risk sex by gender.

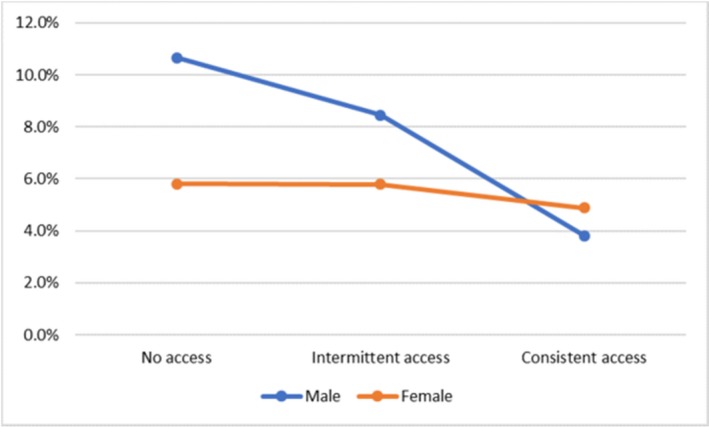

**Conclusions: **Consistent access to structural provisions in early adolescence is critical to delaying debut of HIV‐risk sexual practices for young adolescent boys and girls, whether they live in urban or rural areas.

## MOAD0404

### The *Sawa Sawa* Intervention: A quazi‐experimental trial to reduce community level stigma and improve HIV testing service uptake among men in Sofala Province, Mozambique

**A. Wirtz^1^; E. Mallalieu^2^; J. Chidassicua^3^; D. Pinho^3^; P. Devos^3^ and L. Van Lith^2^**

^1^Johns Hopkins School of Public Health, Epidemiology, Baltimore, United States, ^2^Johns Hopkins Center for Communications Programs, Baltimore, United States, ^3^Johns Hopkins Center for Communications Programs, Maputo, Mozambique

**Background: **In sub‐Saharan Africa, stigma may partially explain low uptake of HIV testing services (HTS) and delayed engagement in care among men compared to women. The *Sawa Sawa* intervention aimed to reduce community‐level stigma and improve uptake of HTS among men in Mozambique.

**Methods:** *Sawa Sawa* (*Sawa*=equality in Sena language) included Positive Prevention (7 sessions for PLHIV), Community Dialogues (6 sessions for all community members), district‐wide radio spots and call‐in programmes, and SMS‐based linkage system that connected people with health facility focal points. Sessions included HIV prevention/care information with anti‐stigma messaging. A quasi‐experimental design was created to estimate the effect of the intervention on 1) community‐level stigma and 2) HTS among adult men. All intervention activities ran continuously between March‐December 2017 in Dondo district; Nhamatanda served as the control district. A longitudinal population‐based survey (N=3000) was implemented pre‐ (November‐December 2016) and post‐intervention (February‐March 2018) in both districts (N=1500/district). Survey included validated stigma measures (Genberg 2008;a=0.79) and self‐reported HIV testing (last 12 months). Statistical analyses compared intervention to control sites and included: residualized change regression models to compare changes in the summed stigma score among all participants and multi‐level random effects model of men's survey data to compare changes in HTS. N=40 male Positive Prevention participants completed qualitative interviews.

**Results: **N=3017 and N=2447 completed the baseline and endline surveys (81% retention); by design, two‐thirds were men. Over 60% of intervention population participated in at least one intervention activity (Figure). Reductions in stigma were associated with the intervention (Beta:‐2.38; 95%CI:‐3.07,‐1.69; *p*< 0.001) and HTS among men increased with the intervention (aOR:1.32; 95%CI:1.01, 1.74; *p*=0.049). Qualitative interviews highlighted past experiences of stigma, observed changes in stigma during the intervention, and the mechanisms by which *Sawa Sawa* supported HIV care.

Abstract MOAD0404‐Figure 1. Coverage of Sawa Sawa intervention exposure in intervention communities.

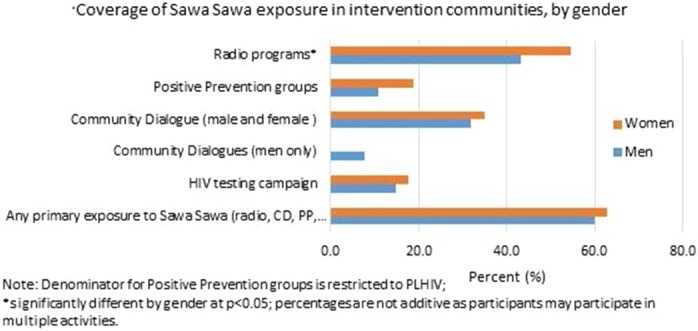

**Conclusions:** *Sawa Sawa* effectively reduced community‐level stigma and improved HIV testing among men and may support achievement of HIV care continuum targets.

## TUAA0101

### Two‐year post‐vaccination follow‐up from APPROACH: Phase 1/2a randomized study evaluating safety and immunogenicity of prophylactic HIV vaccine regimens combining Ad26.Mos.HIV and gp140 envelope protein

**F. Tomaka^1^; D.J. Stieh^2^; D.H. Barouch^3^; M.L. Robb^4,5^; N.L. Michael^4,5^; L. Lavreys^6^; S. Nijs^6^; K. Callewaert^6^; J. Hendriks^2^; Z. Euler^2^; M.G. Pau^2^; H. Schuitemaker^2^ and the APPROACH Study Team**

^1^Janssen Research and Development, Titusville, United States, ^2^Janssen Vaccines & Prevention B.V, Leiden, Netherlands, ^3^Beth Israel Deaconess, Medical Center, Harvard Medical Center, Boston, United States, ^4^Military HIV Research Program, Walter Reed Army Institute of Research, Silver Spring, United States, ^5^Henry M. Jackson Foundation for the Advancement of Military Medicine, Bethesda, United States, ^6^Janssen Pharmaceutica NV, Beerse, Belgium

**Background: **Despite increased availability of new options for HIV prevention, the epidemic remains insufficiently controlled, highlighting the need for a prophylactic vaccine. In APPROACH we evaluated seven combinations of viral vectors expressing mosaic HIV‐1 Env/Gag/Pol antigens and high‐dose (HD) or low‐dose (LD) aluminum phosphate adjuvanted clade C Env gp140 protein. All regimens were well tolerated and immunogenic. Two regimens were selected for longer‐term follow‐up.

**Methods: **In this unblinded follow‐up of APPROACH (phase 1/2a randomized, double‐blind, placebo‐controlled study) long‐term safety and immunogenicity were evaluated at Wk120 and Wk144 in healthy uninfected adults who had received Ad26.Mos.HIV (week [Wk]0 and Wk12) and Ad26.Mos.HIV, gp140HD or LD (Wk24 and Wk48).

**Results: **65 participants (18 to 49 years) vaccinated with Ad26.Mos.HIV, gp140HD (32) or LD (33) entered the long‐term follow‐up (from Thailand, Rwanda, Uganda, South Africa and USA).

No serious adverse events were reported during follow‐up. Immune responses were maintained in both groups with 100% responders to autologous Clade C ELISA at Wk120 and Wk144, in the HD group. Geometric mean titres were 3.5 and 3.3 Log10 at Wk120, and 3.4 and 3.2 Log10 at Wk144, in the HD and LD groups, respectively. Maintained responses were observed over the second year post last vaccination, remaining in the same range as those measured at Wk78 and Wk96. Comparison to a parallel NHP challenge study showed that Wk120 and Wk144 ELISA titers remained higher than NHP responses at Wk72 when they were protected against SHIV challenge.

Abstract TUAA0101‐Figure 1. Env ELISA IgG‐t gp140: Clade C (C97ZA.012).

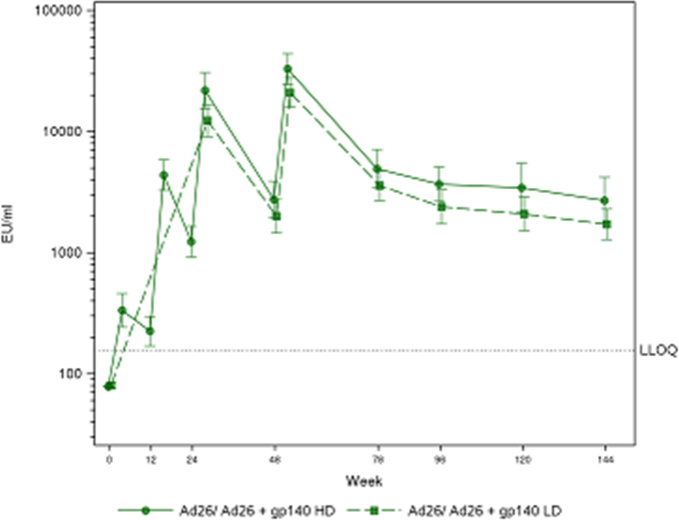

**Conclusions: **In this 2‐year post‐vaccination follow‐up of APPROACH, we observed durable humoral immune responses over 2 years with 100% response rate in the participants receiving the Ad26.Mos.HIV, gp140HD vaccine regimen. No safety issues were seen. Additional immunological analyses and follow‐up will continue (approximately 6 year post‐vaccination).

## TUAA0102

### Protein‐supplemented DNA/MVA vaccines: Preclinical immunogenicity and protection for transmitted/founder (B.63521Δ11mutC) and CD4‐induced gp120 (rhFLSC) proteins

**H.L. Robinson^1^; R. Basu^2^; J.A. Schwartz^3^; M.J. Hauser^2^; C.T. Demarco^4^; A.L. DeVico^3^; B.F. Haynes^4^ and H. Andersen^5^**

^1^GeoVax Inc., Atlanta, United States, ^2^GeoVax Inc, Atlanta, United States, ^3^Institute for Human Virology, Baltimore, United States, ^4^Duke Human Vaccine Institute, Durham, United States, ^5^BioQual, Rockville, United States

**Background: **In Phase 1 and 2a trials, a Clade B DNA/MVA HIV vaccine has elicited antibody (Ab) primarily directed to the gp41 subunit of Env. The only HIV vaccine to achieve some protection (RV144 trial in Thailand) induced antibodies exclusively against the gp120 subunit of Env and antibody to the V1V2 loop of gp120 correlated with reduced risk. Therefore, to enhance gp120 antibodies, including to the V1V2 loop of gp120, gp120 protein boosts are being added to the DNA/MVA vaccine.

**Methods: **72 rhesus macaques have been used in 3 trials testing B.63521Δ11mutC and rhFLSC protein boosts. Animals were primed IM with pGA2/JS7 DNA (3 mg) at weeks 0 and 8 and boosted IM with MVA/HIV62B (1 x 10^8^ TCID50) with or without IM or SC alum‐adjuvanted protein boosts (100 or 300 µg) at weeks 16, 24 and 40. A repeated SHIV‐162P3 challenge was delivered intra‐rectally at 3 or 6 months post the last immunization. Animals were monitored for responding T cells, Ab to gp120, the V1V2, V3 and inner domain (ID2) regions of gp120, gp41 and infection.

**Results: **Both gp120 proteins enhanced Ab responses to gp120 and V3 achieving hyperimmune sera of >1 mg per ml for gp120. However, the CD4‐induced rhFLSC protein was needed to enhance Ab to the V1V2 and ID2 regions of gp120. Ab to V1V2 peaked at estimated titers of 300 to 400 ug per ml after the 2^nd^ boost whereas peak levels of Ab to ID2 increased with the third boost. The protein boost enhanced CD4+ T cell responses to Env but not Gag. The vaccinations did not prevent infection but did increase control of post challenge viremia. The best control was achieved by the DNA prime and two boosts with MVA plus B.63521Δ11 plus rhFLSC. This protection correlated with the peak levels of binding Ab for gp120 (*p*=0.02, r= ‐0.5).

**Conclusions: **The gp120 protein boosts elicited high levels of Ab to gp120. Of the two tested gp120s, the CD4‐induced gp120 elicited the highest Ab to V1V2 and the ID2 regions of gp120. Despite high levels of elicited Ab, protection was limited to post challenge control of viremia.

## TUAA0103

### A vaccine targeting HIV maturation protects Cynomolgus monkeys against vaginal SIVmac251 acquisition

**M. Luo^1,2^; H. Li^1^; R. Omange^1^; B. Liang^12^; N. Toledo^1^; Y. Hai^1^; L. Liu^1^; D. Schalk^3^; T. Dacoba^4^; J. Campo^4^; S.‐Y. Lim^5^; L. Li^1^; M. Kashem^1^; Y. Wan^6^; E. Rakasz^3^; Q. Li^6^; N. Schultz‐Darken^6^; M. Alonso^4^; J. Wnitney^5^ and F. Plummer^1^**

^1^University of Manitoba, Winnipeg, Canada, ^2^National Microbiology Laboratory, Winnipeg, Canada, ^3^University of Wisconsin, Madison, United States, ^4^University of Santiago de Compostela, Santiao de Compostela, Spain, ^5^BIDMC, Harvard Medical School, Boston, United States, ^6^University of Nebraska‐Lincoln, Lincoln, United States

**Background: **HIV‐1 primarily infects a critical component of the human immune system, CD4+ T cells. It mutates rapidly, giving rise to extensive genetic diversity. These inherent characteristics underscore the greater challenges in developing a prophylactic HIV vaccine compared to those for other pathogens. Because activated CD4+ T cells are the primary targets of HIV‐1, virus infection induces inflammation and immune activation that enhances susceptibility to HIV‐1 infection by attracting target cells to the site of infection. These specific characteristics of HIV‐1 infection suggest that effective vaccines to HIV‐1 should 1) reduce the likelihood of the virus finding its target cells; 2) prevent the virus from entering target cells; 3) destroy virally infected cells; 4) cripple infectivity of the progeny viruses released from infected cells to abrogate infection; and 5) tackle the two important characteristics of HIV‐1 that pose a challenge to designing effective HIV‐1 vaccines, i.e. diversity and rapid mutation rate.

With funding from NIH's “Innovation for HIV Vaccine Discovery” programme and CIHR bridge fund, we conducted a more comprehensive evaluation of the efficacy of an HIV vaccine targeting HIV maturation (PCS vaccine) in female Mauritian Cynomolgus macaques. .

**Methods: **The PCS‐vaccine delivers 12 20‐amino acid peptides using two approaches, a modified recombinant vesicular stomatitis virus (rVSVpcs) and nanoformulations (NANOpcs). Female Mauritian Cynomolgus macaques (8/group) were immunized 5 times at weeks 0, 6, 16, 52 and 72 with control rVSV vector or PCS‐vaccine intramuscularly or intranasally. Six months after the last boost the macaques were intravaginally challenged with 250 TCID50 of pathogenic SIVmac251 every two weeks. Viral load was monitored 6, 10 and 14 days after each challenge.

**Results: **The PCS vaccine confers >80% risk reduction per SIVmac251 intravaginal challenge. The 75% of monkeys in the control group were infected after 4 challenges and it took only 2 challenges to infect 50% of control monkeys. It took 11 challenges to infect 50% of PCS vaccine vaccinated monkeys.

**Conclusions: **We showed for the first time that a candidate HIV vaccine targeting sequences surrounding the 12‐protease cleavage sites, other than full Gag and Env can provide significant protection against pathogenic SIVmac251 intravaginal challenges.

## TUAA0104

### A helical structure in the V2 domain of gp120 that localizes to the site of immune pressure in the RV144 vaccine trial is conserved in HIV and SIV envelopes

**S. Lertjuthaporn^1^; C. Cicala^2^; D. Van Ryk^2^; J. Yolitz^2^; D. Wei^2^; G. Gorini^3^; G. Franchini^3^; M. Roederer^3^; R. Mason^2^; K. Pattanapanyasat^1^; X.‐P. Kong^4^; K. Wibmer^5^; L. Morris^5^; A.S. Fauci^2^ and J. Arthos^2^**

^1^Mahidol University, Bangkok, Thailand, ^2^National Institute of Allergy and Infectious Diseases, Bethesda, United States, ^3^National Cancer Institute, Bethesda, United States, ^4^NYU Langone Medical Center, New York, United States, ^5^University of the Witwatersrand, Faculty of Health Sciences, Johannesburg, South Africa

**Background: **Integrin α4β7, which binds with high affinity to both HIV and SIV gp120, mediates trafficking of both naïve and memory CD4^+^T cells to gut tissues. A sieve analysis of the viral quasi‐species in RV144 vaccine recipients, who became infected with HIV identified two residues (K^169^, I^181^) in the V2 region of gp120 as sites of vaccine‐mediated immune pressure. These residues overlap the binding site of integrin α4β7.V2 ‐specific mAbs derived from an RV144 vaccinee, that map to this site, block binding of gp120 to α4β7. A prior study demonstrated that an α4β7mAb reduced the efficiency of SIV transmission in a macaque model of vaginal transmission. These findings prompted us to characterize the nature of both HIV and SIV antibodies that block V2‐α4β7 interactions.

**Methods: **SIV and HIV V2 mAbs derived from infection and vaccination were evaluated for their capacity to inhibit gp120‐ α4β7interactions using a cell‐based adhesion assay. High‐resolution structures of two of these antibodies were obtained by X‐ray crystallography. mAb‐gp120 affinities were measured by surface‐plasmon‐resonance (Biacore).

**Results: **Non‐neutralizing HIV and SIV V2 mAbs blocked gp120‐ α4β7adhesion. Unlike neutralizing mAbs that recognize a b‐sheet that is present in stabilized HIV SOSIP gp120/41 trimers, α4β7‐blocking mAbs recognize a helix structure that is present on unconstrained forms of V2. Remarkably, one of the SIV V2 blocking mAbs cross‐reacted with HIV gp120, which is unusual, but consistent with a conserved α4β7‐reactive helical structure in both HIV and SIV.

**Conclusions: **The capacity of non‐neutralizing V2 domain antibodies to correlate with reduced risk in the RV144 vaccine trial has generated interest in alternative mechanisms of protection from infection. We find that the region of V2, that was linked with protection presents a structure that is distinct from the structure targeted by neutralizing mAbs, is conserved in both HIV and SIV, and encompasses the α4β7 binding site. This conservation raises the possibility that V2 ‐derived immunogens that present this structure may elicit broadly cross‐reactive non‐neutralizing antibody responses that block the interaction between HIV and α4β7 and thus may play a role in vaccine‐induced protection against HIV acquisition.

## TUAA0105

### Lessons from the Research in Viral Eradication of Reservoirs (RIVER) study: Impact of a therapeutic vaccine targeting conserved HIV epitopes on T cell function in treated primary infection

**J. Kopycinski^1^; H. Yang^1^; E. Kim^1^; W. Stohr^2^; T. Hanke^1^; J. Frater^1^; S. Fidler^3^; L. Dorrell^1^ and RIVER Trial Investigators**

^1^Oxford University, Oxford, United Kingdom, ^2^UCL, London, United Kingdom, ^3^Imperial College, London, United Kingdom

**Background: **RIVER is the first randomised controlled trial to assess an HIV eradication ‘kick and kill’ strategy: 60 participants who commenced ART within 4 weeks of confirmed primary HIV infection (PHI) were assigned to one of two groups ≥24 weeks later: ART‐alone or ART plus therapeutic vaccines encoding conserved HIV sequences (‘ChAd63.HIVconsv prime’ followed by ‘MVA.HIVconsv boost’), together with vorinostat, a latency‐reversing agent over 4 weeks (ART+V+V). The lack of impact of ART+V+V on the viral reservoir, over ART‐alone was measured by the frequency of CD4+ T cells harbouring HIV DNA, has been reported previously. Here we describe the trajectories of HIV‐specific T cell responses in the two study arms.

**Methods: **HIVconsv‐specific T‐cell responses were quantified in cryopreserved peripheral blood mononuclear cells from all subjects by intracellular cytokine staining for IFNγ, TNFα, IL‐2, CD154 (an activation marker) and CD107a, which indicates lytic potential. CD8+ T cell killing was assessed in virus inhibition assays (VIA), using elimination of HIV‐superinfected Gag‐positive CD4+ T cells as a read‐out. Responses were measured at enrolment and randomisation to assess the effect of early ART and post‐randomisation (PR) weeks 9 and 12 to assess the effect of V+V.

**Results: **Polyfunctional HIVconsv‐specific IFNγ+CD154+ CD4+ T cell frequencies declined markedly between enrolment and randomisation (medians 0.02% and 0.009%; *p*=0.0001, Mann‐Whitney) but were significantly boosted after vaccination compared with ART‐alone (PR_12 median 0.11% vs. 0.006%; *p*< 0.0001). HIVconsv‐specific IFNγ+CD107a+ CD8+ T cell frequencies declined marginally between enrolment and randomisation (medians 0.08% and 0.07%; *p*=0.65) and were also increased after vaccination (PR_12 median 0.26% vs. 0.06%; *p*=0.0001). ART+V+V‐arm subjects also maintained CD8+ T cells with viral inhibitory capacity whereas these waned in ART‐only subjects (3.4‐fold difference in viral inhibitory activity at PR_12, *p* = 0.026).

**Conclusions: **Along with suppression of plasma viraemia, early ART significantly reduces HIV‐specific effector T cell frequencies and lytic capacity, likely by reducing antigenic drive. These responses were recovered to pre‐therapy levels by vaccination targeting conserved HIV sequences demonstrating the potential for recovery of immune function with prime boost vaccination with ART in early treated PHI. This approach may enhance future combination HIV eradication strategies.

## TUAA0201

### Preferential infection of α4β7+ CD4+ T cells in early acute HIV‐1 infection

**L.R. McKinnon^1,2^; A. Tokarev^3,4^; A. Pagliuzza^5^; A. Sivro^1,2^; E. Kroon^6^; N. Chomchey^6^; N. Phanuphak^6^; A. Schuetz^3,4,7^; M. Robb^3,4^; J. Ananworanich^3,4^; N. Chomont^5^; D. Bolton^3,4^ and on behalf of the RV254/SEARCH010 Study**

^1^University of Manitoba, Medical Microbiology and Infectious Diseases, Winnipeg, Canada, ^2^Centre for the AIDS Programme of Research in South Africa, Durban, South Africa, ^3^Henry M. Jackson Foundation for the Advancement of Military Medicine, Bethesda, United States, ^4^U.S. Military HIV Research Program, Walter Reed Army Institute of Research, Silver Spring, United States, ^5^Centre de Recherche du CHUM and Department of Microbiology, Infectiology and Immunology, Université de Montréal, Montreal, Canada, ^6^South East Asia Research Collaboration in HIV ^SEARCH^, The Thai Red Cross AIDS Research Centre, Bangkok, Thailand, ^7^Department of Retrovirology, Armed Forces Research Institute of Medical Sciences, Bangkok, Thailand

**Background: **CD4+ T cells show differential susceptibility to HIV‐1 infection, and better defining initial viral targets could have important implications for understanding pathogenesis and formation of viral reservoirs. We previously showed that CD4+ T cells expressing the gut homing integrin α4β7 were associated with HIV acquisition and disease progression, and were rapidly depleted from the gastrointestinal mucosa during acute HIV infection. In non‐human primates, preferential SIV detection in α4β7+ CD4+ T cells was observed in the first 10 days of infection, but not subsequently; these data have not been confirmed in humans.

**Methods: **PBMC were obtained from the RV254 acute HIV‐1 infection cohort in Bangkok, Thailand. CD4+ T cells were FACS sorted on the basis of integrin‐beta (clone: FIB504) surface expression into the following three subsets: CD45RA‐ α4β7‐high, CD45RA+ a4b7‐intermediate, and CD45RA‐ a4b7‐negative. Cells were analysed in triplicate for total and integrated HIV DNA by quantitative PCR. Correlation analyses were performed using non‐parametric statistical tests.

**Results: **Participants were all Thai men (median age 28) sampled during Fiebig stage II‐ III (n=6 each). All but one was infected by subtype CRF01_AE and identified a median of 14 days following HIV exposure, with a median plasma viral load (pVL) of 4.9*10^5^ RNA copies/ml. Total and integrated HIV DNA from all subsets correlated strongly with each other and with pVL. CD4+ T cells expressing high α4β7 levels were enriched in total and integrated HIV DNA compared to a4b7‐low cells (*p*=0.03 and *p*=0.003, respectively). For integrated HIV DNA, a4b7‐high CD4+ T cells harbored eight‐fold more DNA than a4b7‐negative cells on average, with increased a4b7‐high HIV DNA in 10/12 participants. This effect did not differ by Fiebig stage, nor did it correlate with HIV exposure timing, pVL, CD4 count, or age. a4b7‐intermediate (CD45RA+) cells contained 1 to 2 logs less HIV‐1 DNA than both a4b7‐negative or ‐high CD45RA‐negative subsets.

**Conclusions: **These data support a role for α4β7+ CD4+ T cells as preferential targets during very early HIV‐1 acute infection, which may contribute toward seeding of gut HIV reservoirs. Efforts to better understand the causes and consequences of this effect may help to inform future HIV cure efforts.

## TUAA0202

### Cellular proliferation maintains genetically intact and defective HIV‐1 over time

**B. Horsburgh^1^; B. Hiener^1^; J.‐S. Eden^1^; E. Lee^1^; T. Schlub^2^; S. von Stockenstrom^3^; L. Odevall^3^; J. Milush^4^; T. Liegler^4^; R. Hoh^4^; R. Fromentin^5^; N. Chomont^5^; S.G. Deeks^4^; F. Hecht^4^ and S. Palmer^1^**

^1^The Westmead Institute for Medical Research, Centre for Virus Research, Westmead, Australia, ^2^University of Sydney, Sydney School of Public Health, Sydney, Australia, ^3^Karolinska Institutet, Department of Microbiology, Tumor and Cell Biology, Stockholm, Sweden, ^4^University of California San Francisco, Department of Medicine, San Francisco, United States, ^5^Université de Montréal, Montreal, Canada

**Background: **An understanding of the mechanisms maintaining replication‐competent HIV will be needed to design eradication therapies. We examined the role of cellular proliferation in maintaining intact and defective proviruses within memory CD4+ T‐cell subsets from individuals on prolonged ART.

**Methods: **Naïve, central (CM), transitional (TM), effector (EM), HLA‐DR+ and HLA‐DR‐ memory CD4+ T‐cells were sorted from the peripheral blood of eight participants on long‐term ART. Additional sequences from four participants were obtained four years later. Full‐length individual proviral sequencing, which amplifies 92% of the genome, was used to characterise proviruses as intact or defective. Expansions of identical sequences (EIS) were classified as ≥2 identical sequences.

**Results: **At the early time‐point, 1041 sequences were obtained with 4% considered intact. The proportion of intact proviruses was different across cell subsets (*p*< 0.001), with the highest in EM and HLA‐DR+ cells. The proportion of intact and defective proviruses in an EIS was similar. When stratified by treatment duration, the proportion of all sequences in an EIS was higher in those on therapy for >14 years (n=6 participants). No intact expanded sequences were observed in participants on therapy for < 5 years (n=2 participants). Expanded intact sequences were predominantly found in EM and HLA‐DR+ cells, representing 24% and 17% of all intact sequences respectively. These intact expanded sequences were observed in two participants four years later. In two participants where no intact provirus was observed, large expansions of defective sequences predominated. In one participant these sequences expanded over four years, representing 41% (28/68) and 78% (167/215) of sequences at each time‐point. The expansion in the second participant was stable, with 46% (110/241) and 45% (91/202) of sequences belonging to this EIS at each time‐point.

**Conclusions: **Cellular proliferation contributes to the expansion of both intact and defective proviruses. Expansions of defective proviruses may dilute the number of intact proviruses and lead to difficulty in their identification. Genetically identical intact proviruses are enriched in HLA‐DR+ and EM cells ‐ cells with a higher proliferation potential ‐ and these proviruses are stable over time. This indicates that the latent HIV reservoir is maintained in these peripheral blood T‐cell subsets by proliferation.

## TUAA0203

### Adipose tissue contributes to viral persistence in ART‐treated SIV.sab infection in pigtailed macaques

**P. Sette^1^; E. Brocca‐Cofano^1^; R. Sivanandham^1^; E.P. Falwell^1^; B. Policicchio^1^; C.L. Xu^1^; T. He^1^; W.M. McFadden^1^; K.D. Raehtz^1^; T.L. Dunsmore^1^; G. Haret‐Richter^1^; A. Landay^2^; I. Pandrea^1^ and C. Apetrei^3^**

^1^Center for Vaccine Research, University of Pittsburgh, Pathology, Pittsburgh, United States, ^2^Rush University Medical Center, Department of Immunology and Microbiology, Chicago, United States, ^3^Center for Vaccine Research, University of Pittsburgh, Microbiology and Molecular Genetics, Pittsburgh, United States

**Background: **Antiretroviral therapy (ART) effectively suppresses viremia in HIV‐infected patients and SIV‐infected RMs. Yet, ART does not restore immune integrity and is not curative, with the virus persisting in a latent reservoir and rebounding upon ART cessation. An increasing body of evidences suggests that adipose tissue (AdT) is a key anatomical reservoir that contributes to both viral persistence and chronic immune activation/inflammation. We used our new model of highly pathogenic SIV.sab infection of PTMs, treated with a coformulated combination of Emtricitabine [FTC], tenofovir disoproxil fumarate [PMPA] and Dolutegravir [DTG] to address the role of AdT during viral infection.

**Methods: **Six SIVsab‐infected PTMs received the coformulated regimen for 14 months from 48 days post‐infection (dpi). Plasma viral loads (pVLs) were quantified by qRT‐PCR assay. Cells isolated from the AdT were immunophenotyped by flow‐cytometry and immunohistochemistry (IHC). DNAscope and cell‐associated DNA (CA‐DNA) were also performed to evaluate viral persistence in the AdT.

**Results: **ART resulted in a robust viral control between 16 and 164 days post‐treatment (dpt), with only rare blips occurring during the follow‐up. Large numbers of T cells were observed in both white and brown AdT and they were located both perivascularly or diffuse in the fat. The majority of the CD4+ T cells isolated from the AdT was of central memory phenotype and expressed low levels of Ki‐67 and HLA‐DR suggesting low levels of activation. DNAscope showed virus persistence in the blood vessels cells from AdT. CA‐DNA measurements in cells isolated from AdT and on snap‐frozen tissue fragments of abdominal skin, peritoneal fat and pericardial fat collected at the necropsy confirmed AdT as a virus reservoir. By IHC we showed that the cells infiltrating the AdT produce IL‐6 and have an increased expression of MXA‐1, indicating AdT as a source of residual INFL.

**Conclusions: **Our study shows that the AdT is a major anatomical site of virus persistence and immune activation/inflammation in a new model of ART‐treated SIV infection in Pigtail macaques. These findings confirm that AdT should be targeted by the HIV/SIV cure strategies.

### TUAA0204

#### Selective death induction of HIV‐1 infected myeloid reservoirs

**C. Gavegnano^1^; C. Shephard^2^; J. Holler^3^; S. Coggins^3^ and B. Kim^1^**

^1^Emory University School of Medicine, Pediatrics, Atlanta, United States, ^2^Emory University, Pediatrics, Atlanta, United States, ^3^Emory School of Medicine, Pediatrics, Atlanta, United States

**Background: **Existing antiretroviral therapy (ART) cannot efficiently eliminate HIV within the CNS. HIV persistence in myeloid sanctuaries represents a major barrier to eradication, and drives HIV associated neurocognitive dysfunction (HAND), which occurs in up to half of HIV‐infected individuals even with well‐controlled viremia. Safe, specific agents that selectively eliminate key cells harboring the myeloid reservoir are urgently needed. Our group has identified two safe, FDA approved agents rufinamide and bergenin (non‐HIV indication) that demonstrate selectivity for killing of only HIV‐infected macrophages.

**Methods:** *Cell isolation*: Primary human macrophages were isolated from healthy donors and differentiated with GM‐CSF. Memory T cells or activated CD4 T cells were isolated with magnetic beads (Miltenyi).

*HIV infections*: Macrophages were infected with HIV‐1bal (MOI 0.5) for 72 hr in the presence of 1 or 10 µM rufinamide or bergenin, HIV alone, or HIV+VPX (positive control). Effect on HIV infection was quantified (intracellular/extracellular p24, 2‐LTR circles). Effect on cell killing of HIV‐infected cultures+drug versus HIV infection alone, and HIV uninfected cells were quantified (FACS live/dead stain and MTT assay). Effect on dNTP pools and SAMHD1/pSAMHD1 were quantified.

**Results: **Rufinamide and bergenin do not kill uninfected macrophages. Both agents demonstrate selectivity for killing HIV‐infected macrophages, and increase cell killing in HIV‐infected cultures 3 to 4 fold versus HIV‐infected cultures without drug. Both agents significantly accelerate HIV replication in macrophages (intracellular and extracellular p24, and 2‐LTR circles) versus HIV infection alone. Both agents increase dATP levels in macrophages, but do not modulate SAMHD1/pSAMHD1 levels.

**Conclusions: **Rufinamide and bergenin demonstrate selectivity for killing only HIV infected macrophages, and increase cell killing in HIV‐infected cultures at least 4‐fold versus HIV infected cultures without drug. Agents accelerate HIV replication in macrophages, implying acceleration results in selective cell death of infected macrophages. Acceleration of replication is coupled with increase in dATP, but not SAMHD1/pSAMHD1; regulation of acceleration and cell death is conferred by increased dNTPs but not directly by SAMHD1. Bergenin and rufinamide demonstrate selectivity towards killing of only HIV‐infected macrophages, warranting further mechanistic studies to evaluate the use of these agents towards elimination of myeloid derived viral sanctuaries systemically and within the CNS.

### TUAB0101

#### Periconceptional antiretroviral exposure and central nervous system (CNS) and neural tube birth defects ‐ data from Antiretroviral Pregnancy Registry (APR)

**L.M. Mofenson^1^; V. Vannappagari^2^; A.E. Scheuerle^3^; B. Baugh^4^; K.P. Beckerman^5^; H. Betman^6^; N. Chakhtoura^7^; K. Dominguez^8^; A. Pikis^9^; N.S. Santanello^10^; W.R. Short^11^; C.T. Thorne^12^; H. Tilson^13^; V. Vinas^14^; H. Watts^15^ and J.D. Albano^16^**

^1^Elizabeth Glaser Pediatric AIDS Foundation, Washington, DC, United States, ^2^ViiV Healthcare, Research Triangle Park, United States, ^3^University of Texas Southwestern Medical Center, Dallas, United States, ^4^Janssen Scientific Affairs, LLC, NYC, United States, ^5^Mt Sinai Bronx Lebanon Hospital, Bronx, United States, ^6^AbbVie, Chicago, United States, ^7^National Institutes of Health, Washington, DC, United States, ^8^US Centers for Disease Control and Prevention, Atlanta, United States, ^9^Food and Drug Administration, Washington, DC, United States, ^10^Independent Pharmacoepidemiologist, Philadelphia, United States, ^11^University of Pennsylvania, Philadelphia, United States, ^12^University College of London, London, United Kingdom, ^13^UNC School of Public Health, Chapel Hill, United States, ^14^Mylan Laboratories, Morgantown, United States, ^15^Office of the Global AIDS Coordinator and Health Diplomacy, Washington, DC, United States, ^16^Syneos Health, Raleigh, United States

**Background: **Preliminary data from Tsepamo Botswana birth defects surveillance study identified potential neural tube defect (NTD) teratogenic signal in infants born to HIV‐infected women receiving dolutegravir (DTG)‐based antiretroviral therapy (ART) during the periconceptional period (before conception and into first trimester), compared with periconceptional non‐DTG ART or women without HIV (0.67%, 0.12%, and 0.09%, respectively) (Jul2018 IAS Conference). This analysis aims to 1) describe CNS defect cases reported to APR, a voluntary, international, prospective exposure registry and 2) determine any increased risk by ART drug class.

Abstract TUAB0101‐Table 1. Number of CNS and NTD defect cases with periconceptional exposure by drug class, antiretroviral pregnancy registry, January 1989 through 31 July 2018

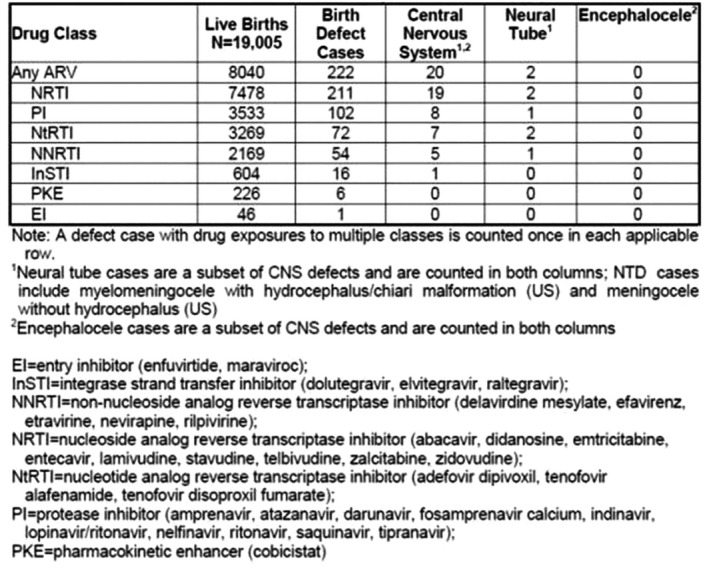

**Methods: **Data on prospectively enrolled pregnancies (Jan1989 through Jul2018) with birth outcome are summarized. Birth defects are reviewed by a dysmorphologist, coded by modified Metropolitan Atlanta Congenital Defects Programme criteria, classified by organ system and assigned exposure timing for each antiretroviral. CNS defects include NTD (myelomeningocele/spina bifida, anencephaly) and encephalocele which is reported separately from NTD.

**Results: **20,064 pregnancies resulted in 20,413 fetal outcomes including 19,005 live births. Reported pregnancies are from North America (75%), Europe (8%), Africa (7%), South America (6%) and Asia (4%). Of the 19,005 live births with any ART exposure, 8040 had periconceptional exposure, including 222 birth defect cases and 20 CNS defects (2 NTD and no encephalocele). See Table for drug class data.

**Conclusions: **Twenty CNS defects (2 NTD) were observed among 8040 birth outcomes with periconceptional ART exposure. Overall and drug class frequencies are consistent with observed low NTD prevalence (0.01%‐0.1%) in developed countries where food folic acid fortification and antenatal folic acid supplementation are prevalent, reducing overall NTD occurrence. However, the number of pregnancies enrolled in the APR with exposure to newer drug classes such as integrase inhibitors (InSTIs) are insufficient to rule out or confirm any potential association with NTD. Healthcare providers are encouraged to continue to report pregnancies with prospective antiretroviral exposures to the APR, especially those involving newer antiretrovirals.

### TUAB0102

#### Adverse pregnancy outcomes among HIV‐positive women in the era of universal antiretroviral therapy (ART) remain elevated compared with HIV‐negative women in Lesotho

**V.J. Tukei^1^; A. Tiam^2,3^; L. Greenberg^2^; H. Hoffman^4^; T. Ramatlapeng^5^; M. Nchephe^5^; M. Nchepe^5^; T. Motsoane^5^; F. Mohai^1^; A. Chabela^1^; M. Masitha^1^; L.M. Mofenson^2^; R. Steenstra^4^ and L. Guay^2,4^**

^1^Elizabeth Glaser Pediatric AIDS Foundation, Maseru, Lesotho, ^2^Elizabeth Glaser Pediatric AIDS Foundation, Washington, United States, ^3^Universiity of Bergen, Bergen, Norway, ^4^George Washington University School of Public Health, Washington, United States, ^5^Ministry of Health ‐ Lesotho, Maseru, Lesotho

**Background: **Prior to the ART era, adverse pregnancy outcomes were more common among HIV‐positive than HIV‐negative women. In the universal ART era, most HIV‐positive pregnant women are receiving ART, many prior to pregnancy. There are conflicting data whether adverse pregnancy outcomes among HIV‐positive women on ART in the current era of “test and treat” remain higher than among HIV‐negative women.

**Methods: **Pregnancy outcomes data were collected from an ongoing prospective study (started 2016) evaluating a multidisciplinary “Integrated Management Team to Improve Maternal‐Child Outcomes (IMPROVE)” intervention to improve maternal‐child health and HIV service uptake/retention. HIV‐positive and HIV‐negative pregnant women at 12 facilities in Lesotho were enrolled at their first antenatal visit (ANC) in a cluster randomized evaluation of the IMPROVE intervention versus standard of care, with prospective follow‐up through 12 to 24 months postpartum. We used combined data from both randomized groups on delivery outcomes. Chi‐square tests were used to test for statistical significance of differences between outcomes of HIV‐positive versus HIV‐negative women.

**Results: **1002 women were enrolled, with delivery data captured for 903 women to date (563 HIV‐positive and 340 HIV‐negative). Mean gestational age at enrollment was 20 weeks regardless of HIV status. Among HIV‐positive women, 74% knew their HIV status prior to their first ANC visit, with 92% already receiving ART (88% TDF + 3TC+EFV); among women first diagnosed with HIV during pregnancy, 95% started on ART (98% TDF + 3TC+EFV). HIV‐positive women were more likely than HIV‐negative women to experience a miscarriage or have a premature or low birthweight infant (mean 2.8 kg vs. 3.0 kg, respectively) (Table 1). Stillbirths and congenital anomalies were non‐significantly higher in HIV‐positive than HIV‐negative women (3.7% vs. 2.1% and 2.0% vs. 1.2%, respectively). Among HIV‐positive women, timing of ART initiation was not associated with any of the measured outcomes.

**Conclusions: **Despite nearly universal ART, with most women on ART before pregnancy, adverse birth outcomes remained elevated among HIV‐positive compared to HIV‐negative women.

**Table:** Abstract TUAB0102‐Table 1. Pregnancy Outcomes among a cohort of women in Lesotho

Adverse Birth Outcomes	HIV‐Negative (n = 340)	HIV‐Positive (n = 563)	*p* value
Miscarriage(<28 weeks gestation)	1/340 (0.3%)	15/563 (2.7%)	0.009
Stillbirth (>28 weeks gestation)	7/340 (2.1%)	21/563 (3.7%)	0.173
Prematurity (<37 weeks)	14/330 (4.2%)	45/541 (8.3%)	0.020
Low birth weight (<2500 g)	28/303 (9.2%)	75/487 (15.4%)	0.012
Congenital abnormality	4/332 (1.2%)	11/545 (2.0%)	0.470

### TUAB0103

#### Vaginal inflammation is associated with initiating antiretroviral therapy in pregnancy and with spontaneous preterm birth

**K.J. Rittenhouse^1^; H. Mwape^2^; K. De Paris^3^; J.T. Price^14^; J.A.E. Nelson^5^; E. Stringer^1^; M.E. Smithmyer^1^; B. Vwalika^4^ and J.S.A. Stringer^1^**

^1^University of North Carolina at Chapel Hill School of Medicine, Division of Global Womens’ Health, Chapel Hill, United States, ^2^UNC Global Projects Zambia, Lusaka, Zambia, ^3^University of North Carolina at Chapel Hill School of Medicine, Department of Microbiology and Immunology, Chapel Hill, United States, ^4^University of Zambia School of Medicine, Department of Obstetrics and Gynaecology, Lusaka, Zambia, ^5^University of North Carolina at Chapel Hill School of Medicine, Department of Microbiology, Chapel Hill, United States

**Background: **HIV infection and timing of ART initiation are associated with spontaneous preterm birth (sPTB) in some studies. Whether maternal immune mechanisms underpin this risk remains unclear. We evaluated plasma and vaginal inflammation by HIV serostatus (HIV+ vs. HIV‐) and preconceptional ART exposure (ART+ vs. ART‐) and assessed their association with sPTB.

**Methods: **Peripheral plasma and mid‐vaginal swab specimens were collected in the Zambian Preterm Birth Prevention Study cohort. Panels of 13 plasma and 14 vaginal fluid analytes were measured using a multiplex immunoassay from 241 paired baseline specimens (16 to 20 gestational weeks) of all HIV+ and sPTB women and a random subset of HIV‐ term birth women, using inverse‐probability weighting to account for sampling. We repeated paired panels in 56 HIV+ women with repeat specimens (24 to 34 weeks). We used confirmatory factor analyses of plasma and vaginal inflammation to score inflammation and compared by HIV and ART sub‐groups using linear regression. We assessed associations between 16 to 20 week pro‐inflammatory scores in plasma and vaginal fluid and the risk of sPTB < 37 weeks using univariate and multivariate logistic regression, excluding pregnancies with multiple gestation and short cervical length (strong sPTB predictors).

**Results: **At baseline, HIV+ART‐ women (coef 0.33; *p *=* *0.02) had greater vaginal inflammation than their HIV‐ counterparts, but no significant difference was observed between HIV+ sub‐groups. In repeat specimens, HIV+ART‐ women (most of whom started therapy between baseline and repeat) had greater vaginal inflammation than HIV+ART+ women (coef 0.57; *p *<* *0.01). In multivariate logistic regression, 1 to 2 prior preterm births (coef 1.4; *p *=* *0.02), 3 or more prior preterm births (coef 2.0; *p *=* *0.02), and vaginal inflammation (coef 1.04; *p *=* *0.03) positively correlated with sPTB. Plasma inflammation did not differ between HIV and/or ART sub‐groups and was not correlated with sPTB in univariate or multivariate analyses.

**Conclusions: **Vaginal, but not systemic, inflammation in the midtrimester is associated with sPTB. In apparent contrast to epidemiologic reports of higher PTB risk among women on preconceptional ART, women newly starting ART in our cohort had higher vaginal inflammation than those who were on it at baseline. Further studies are underway to confirm these data with a larger sample size of our highly relevant African cohort.

### TUAB0104

#### Characterizing viral load burden among HIV‐infected women at time of delivery: Findings from four tertiary obstetric units in Gauteng, South Africa

**F. Moyo^1,2,3^; A. Haeri Mazanderani^4,5^; T. Murray^4,6^; K.‐G. Technau^7^; S. Carmona^8^; T. Kufa^2,4^ and G.G. Sherman^4,6,7^**

^1^National Institute for Communicable Diseases, National Health Laboratory Service, Centre for HIV & STIs, Sandringham, Johannesburg, South Africa, ^2^Witwatersrand University, School of Public Health. Faculty of Health Sciences, Johannesburg, South Africa, ^3^Wits Health Consortium, Paediatric HIV Diagnostics, Johannnesburg, South Africa, ^4^National Institute for Communicable Diseases, National Health Laboratory Service, Centre for HIV & STIs, Johannesburg, South Africa, ^5^University of Pretoria, Department of Medical Virology, Pretoria, South Africa, ^6^Wits Health Consortium, Paediatric HIV Diagnostics, Johannesburg, South Africa, ^7^Witwatersrand University, Empilweni Services and Research Unit. Rahima Moosa Mother and Child Hospital. Department of Paediatrics and Child Health. Faculty of Health Sciences., Johannesburg, South Africa, ^8^Witwatersrand University, Department of Moelcular Medicine and Haematology. Faculty of Health Sciences., Johannesburg, South Africa

**Background: **In the South African public sector, antiretroviral therapy (ART) coverage amongst antenatal clients is estimated at > 95% and 90% of all women on ART are virologically suppressed. We describe maternal viral load (VL) burden at four tertiary obstetric units in Gauteng, South Africa.

**Methods: **Between June‐December 2018, routine point‐of‐care VL and early infant diagnosis (EID) PCR testing was introduced at four tertiary obstetric units in Gauteng‐ three in Johannesburg (subdistricts B, D, F) and one in Tshwane. Testing was restricted to 08 h00 to 16 h00 during weekdays. All HIV‐infected women and HIV‐exposed neonates were eligible for HIV VL and EID tests respectively around time of delivery using Cepheid Xpert^®^. Proportions of viraemic women at delivery were calculated. Percentage testing coverage of maternal VL and neonatal EID were calculated using live‐births to HIV‐positive women, obtained from routine records. Programmatic laboratory data were used to determine intra‐uterine (IU) transmission rates per unit.

**Results: **Overall, there were 5764 live‐births to HIV‐positive women of whom 1892 (32.8%) women had a valid VL result and 3188 (55.3%) neonates had a valid EID test result. Overall median VL at delivery was < 40 copies/mL (IQR: 0 to 483). The proportion of women with a VL ≥ 50, ≥400 and ≥ 1000 copies/mL was 37.1%, 25.4% and 22.7%, respectively (Table 1). Both a higher proportion of viraemic women and intrauterine infected neonates were observed for the Tshwane unit (*p *=* *0.001). Percentage positivity rates amongst study participants were comparable to overall intra‐uterine rates calculated from routine data suggesting generalisability.

**Conclusions: **Overall, >20% (n = 429) HIV‐infected women at time of delivery had a VL ≥ 1000 copies/mL suggesting higher VL burden compared to women on ART in general. Although testing coverage varied, % neonatal positivity approximated programmatic IU‐transmission rates. Scale‐up of VL monitoring and improving quality of antenatal care is required for elimination of mother‐to‐child transmission of HIV.

**Table:** Abstract TUAB0104‐Table 1. Maternal viral load and early infant diagnosis PCR testing at delivery: results from four tertiary obstetric units in Gauteng, South Africa

	Maternal PoC VL					Neonatal PoC EID		Programmatic
Site of Tertiary Obstetric Unit	Live births to HIV‐positive woman	Valid VL result N (%)	Median VL cps/ml (IQR)	VL ≥ 50 cps/ml N (%)	VL ≥ 400 cps/ml N (%)	VL ≥ 1000* cps/ml N (%)	Valid EID result N (%)	PCR positive N (%)	% IU transmission per Unit
Johannesburg B	1529	914 (59.8)	<40 (0 to 269)	318 (34.8)	218 (23.9)	194 (21.2)	987 (64.6)	16 (1.6)	1.7
Johannesburg D	2297	376 (16.4)	<40 (0 to 242)	131 (34.8)	85 (22.6)	81 (21.5)	955 (41.6)	13 (1.4)	1.7
Johannesburg F	1173	268 (22.8)	<40 (0 to 258)	92 (34.3)	62 (23.1)	52 (19.4)	663 (56.5)	8 (1.2)	1.3
Tshwane Metro	765	334 (43.7)	<40 (0 to 7650)	160 (47.9)	115 (34.4)	102 (30.5)	583 (76.2)	19 (3.3)	2.5
Total	5764	1892 (32.8)	<40 (0 to 483)	701 (37.1)	480 (25.4)	429 (22.7)	3188 (55.3)	56 (1.8)	1.7

N, number VL, viral load EID, early infant diagnosis cps/ml, copies per millilitre PCR, polymerase chain reaction IU, intra‐uterine PoC, point‐of‐care * Median VL = 26,000 cps/ml (IQR: 7490 to 87,900)

### TUAB0105

#### Dual epidemics: The impact of HIV and obesity on pregnancy outcomes among women in South Africa

**A. Bengtson^1^; T. Phillips^2^; S. le Roux^2^; E.J. Abrams^3^ and L. Myer^2^**

^1^Brown University School of Public Health, Department of Epidemiology, Providence, United States, ^2^University of Cape Town, Cape Town, South Africa, ^3^Columbia University, Department of Epidemiology and Pediatrics, New York, United States

**Background: **South Africa faces dual epidemics of HIV and obesity. However, little research has explored the impact of HIV and obesity on pregnancy outcomes.

**Methods: **We followed HIV‐uninfected and HIV‐infected pregnant women initiating antiretroviral therapy (ART) from first antenatal visit (ANC; baseline) through 12‐months postpartum. At baseline, gestational age (GA) was estimated using ultrasound. Maternal anthropometry was measured at baseline and 12‐months postpartum. Body mass index (BMI) was categorized as underweight (< 18.5 kg/m^2^), normal (18.5‐< 25.0), overweight (25.0‐< 30.0) and obese (>30.0). We used modified Poisson regression to estimate associations between BMI category and adverse pregnancy outcomes, and explored modification by HIV‐status.

**Results: **We included 884 women (HIV‐infected 53% and HIV‐uninfected 47%). At baseline, 39% of women were obese and 3% underweight. Overall, 11% of infants were preterm (< 37 weeks’ gestation, n = 96), 11% were low birthweight (LBW, < 2500 g, n = 100), 12% were small for GA (SGA, < 10^th^ percentile for GA, n = 102) and 11% were large for GA (LGA, >90^th^ percentile for GA, n = 95). In multivariable analyses, overall obesity was not associated with preterm birth (RR 0.89, 95% CI 0.53, 1.50) or LGA (RR 1.39, 95% CI 0.86, 2.25). Among HIV‐uninfected women, obesity was associated with LGA (RR 2.15, 95% CI 1.15, 4.02), while being underweight increased the risk of LBW (RR 3.55, 95% CI 0.76, 16.60) and SGA (RR 1.86 95%, CI 0.49, 6.99), although estimates were imprecise. Among HIV‐infected women, normal BMI women had the highest risk of LBW (19%) and SGA (15%) and few infants (6%) were LGA (Figure).

Abstract TUAB0105‐Figure 1. Adverse pregnancy outcomes by body mass index (BMI) category for 884 pregnant women.

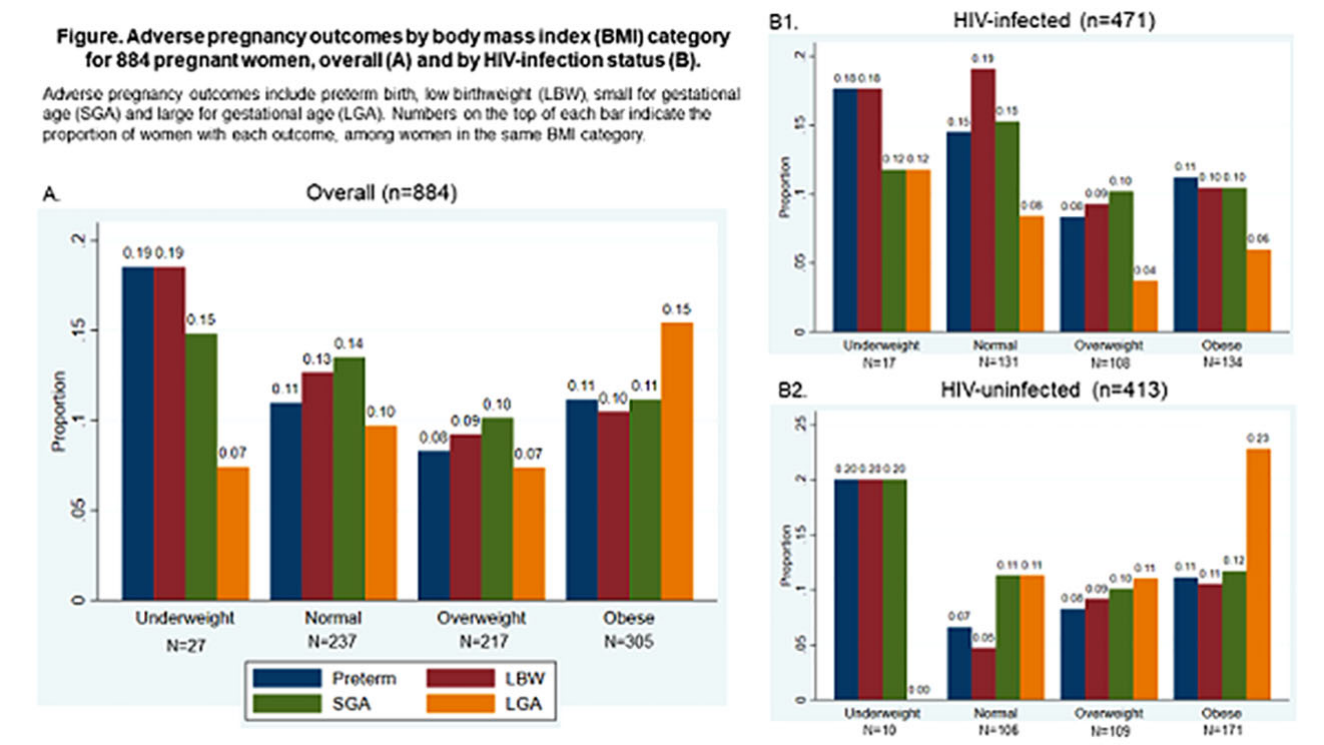

**Conclusions: **In our population, associations between BMI and adverse pregnancy outcomes differed by HIV‐status. Among HIV‐uninfected women, being obese or underweight may have increased the likelihood of several adverse pregnancy outcomes. Conversely, HIV‐infected women at normal BMI were at higher risk of adverse outcomes, including LBW and SGA.

### TUAC0101

#### Transgender people's experiences in HIV testing, sexual partner based violence and mental health in Malawi

**E. Umar^1^; R. Chalera^2^; G. Trapence^2^; C. Bandawe^3^ and V. Jumbe^4^**

^1^University of Malawi, College of Medicine, Health Systems and Policy, Blantyre, Malawi, ^2^Centre for the Development of People, Lilongwe, Malawi, ^3^University of Malawi, Mental Health, Blantyre, Malawi, ^4^University of Malawi, Health Systems and Policy, Blantyre, Malawi

**Background: **This study set out to establish the experiences of transgender people in relation to health seeking in Malawi. It focused on experiences with the health system in relation to HIV testing; partner sexual based violence; and mental health.

**Methods: **The study was conducted in August 2018 in four administrative districts of Malawi. These were cities in the three regions and in addition, a rural district in the southern region. This was a cross sectional study utilizing mixed methods. The study population was people who identify themselves as transgender in Malawi. Data collection was conducted using a structured questionnaire that included a depression scale as well as Focus Group discussion (FGD) guide. Descriptive data analysis was conducted using SPSS v.22. Thematic content analysis using NVIVO was used for FGDs.

**Results: **A total of 117 transgender people, comprising of self‐reported transwomen (55.5%), transmen (41.0%) and genderqueer / non‐binary (3.4%) participated in the study. Demographically, the sample was relatively young (mean age = 23.3, SD 4.0); and mostly out of school (70.7%). More than 80% were or had gone to high school. Regarding HIV testing, majority (91.5%) reported ever testing for HIV. It was established that majority had accessed HIV testing in nongovernmental transgender friendly drop in centres. However, their experiences with the health system was not good, with 42%, reporting ever feeling afraid to seek health care, 24% being ever denied health care by a health care provider because they were transgender. Almost a third (30.7%) reported being abused by their sexual partner. In terms of mental health, 64.8% reported features of common mental disorder while 23% had considered committing suicide in the past 12 months.

**Conclusions: **The study reveals that transgender people experience various difficulties that make them avoid seeking health care including HIV/AIDS related services; they experience partner sexual based violence and considerably high rates of mental health problems. In order to enhance HIV prevention and treatment, there is need for the creation of transgender safe spaces in Malawi.

### TUAC0102

#### Acceptability of HIV and syphilis domiciliary testing among transgender women in Buenos Aires, Argentina

**V. Zalazar^1^; C. Frola^1,2^; A. Gun^1^; N. Cardozo^1,3,4^; M. Duarte^1,3,4^; S. Fabian^1,5^; P. Radusky^1,6^; I. Aristegui^1,7^; P. Cahn^1^ and O. Sued^1^**

^1^Fundacion Huesped, Research Department, Buenos Aires, Argentina, ^2^Hospital Juan A. Fernández, Infectious Diseases Unit, Buenos Aires, Argentina, ^3^Asociación de Travestis, Transexuales y Transgéneros de Argentina ^A.T.T.T.A.^, Buenos Aires, Argentina, ^4^REDLACTRANS, Buenos Aires, Argentina, ^5^Asociación Civil Gondolin, Buenos Aires, Argentina, ^6^Universidad de Buenos Aires, Buenos Aires, Argentina, ^7^Universidad de Palermo, Buenos Aires, Argentina

**Background: **HIV and syphilis, although easily diagnosed by simple laboratory low‐cost methods, remain important health problems for transgender women (TGW), who face numerous barriers to access to the health system. This study aimed at assessing the acceptability of a domiciliary‐provider‐initiated HIV and syphilis testing strategy.

**Methods: **From 5/2018 to 12/2018, a multidisciplinary team tested TGWs in rooming‐houses and other venues. Inclusion criteria were: self‐identified as TGW; ≥14 years old; previous HIV‐negative > 3 months or unknown status; non‐history of syphilis or previous episode with > 6 months after treatment. Acceptability survey included 5 items with a 5 point‐Likert scale, a satisfaction question, and comments. SD BIOLINE HIV/Syphilis Duo rapid tests (provided by Abbott) was used (detects antibodies to HIV‐1 including subtype‐O, HIV‐2 and Treponema pallidum). HIV+ was confirmed by VL and positive treponemal tests were complemented with quantitative VDRL to identify active syphilis or past infection. All confirmed cases were referred for treatment initiation and follow‐up.

**Results: **A total of 68 TGW were tested with a median age of 26 (IQR: 25.7 to 29.8). Most of them were sex workers (77.9%). HIV prevalence was 4.4% and 50% had syphilis antibodies (26.5% indicating incident syphilis and 23.5% showing adequate response to previous treatment). Almost all (98.5%) considered the domiciliary rapid test as a very good/good strategy. Participants strongly agree that they: prefer simultaneous HIV and syphilis diagnosis test (60.3%), prefer to receive results the same day (85.3%), think this rapid test is safe and reliable (77.9%) and would be willing to repeat it in the future (95.6%). All participants strongly agreed that they would recommend this test to another TGW. The main comment was to include other STIs in rapid test (i.e., HBV, HCV, etc.).

**Conclusions: **TGW have a high prevalence of syphilis and HIV. Research activities constitute a fundamental input to inform evidence‐based policies on the feasibility and acceptability of new strategies for the diagnosis of STIs that contributes to the development of appropriate and effective interventions to promote access to health services. Our pilot study showed that domiciliary rapid testing of STIs is a feasible, acceptable and a successful approach for this hard‐to reach population.

### TUAC0103

#### Evaluation of syndemics in transgender women using pre‐exposure prophylaxis (PrEP) for HIV prevention: Preliminary findings

**M. Ramos; E. Jalil; F. Lessa; C. Castro; C. Jalil; E. Carvalheira; L. Kamel; R.I. Moreira; V. Pacheco; V.G. Veloso; B. Grinsztejn and R. B. De Boni**

FIOCRUZ, LAPCLIN DST/AIDSLAPCLIN DST/AIDS, Instituto Nacional de Infectologia Evandro Chagas, Rio de Janeiro, Brazil

**Background: **Despite advances in HIV prevention, transgender women (transwomen) remain at increased risk for HIV infection. As seen in other key populations, syndemics, defined as synergistic psychosocial comorbidities, may exacerbate this risk. There are few studies describing Syndemics for transwomen in low‐and‐middle income countries (LMIC). We aimed to assess the prevalence of syndemics and high‐risk behaviour among transwomen in Rio de Janeiro, Brazil.

**Methods: **This is a cross‐sectional analysis of data collected at the screening visit of PrEParadas, a pre‐exposure prophylaxis (PrEP) demonstration trial designed for transwomen. Using standardized and validated questionnaires, participants were screened for a history of substance abuse, binge drinking, depression, sexual compulsive behaviour and intimate partner violence. We considered the presence of 2 + aforementioned conditions as syndemics. We compared sexual behaviour, substance abuse, history of child abuse and victimization among transwomen with and without evidence of syndemics using Chi‐square tests.

**Results: **Of 165 transwomen surveyed, 135 had valid results and were included in the present analysis. The median age was 30 years (interquartile range 24 to 26) 25.4% were black, 28.4% completed an elementary school education, and 44% were currently unemployed. The prevalence of syndemics was 46.6%. Transwomen presenting syndemics had statistically significant higher prevalences of tobacco abuse, alcohol abuse, cocaine abuse, sexual and violence victimization, child abuse and suicidal risk, as compared to non‐syndemic transwomen (Table 1).There were no statistical differences between groups regarding transactional sex (overall prevalence 73.5%), unprotected anal sex (82.6%), and history of sexually transmitted infections (24.2%).

**Conclusions: **The prevalence of syndemics was high in Brazilian transwomen. Improvements in targeted interventions for mental health and social vulnerabilities are necessary in this population, Syndemics must be considered when designing PrEP engagement and adherence strategies for transwomen in order to improve and reduce the prevalence of HIV infection.

Abstract TUAC0103‐Table 1

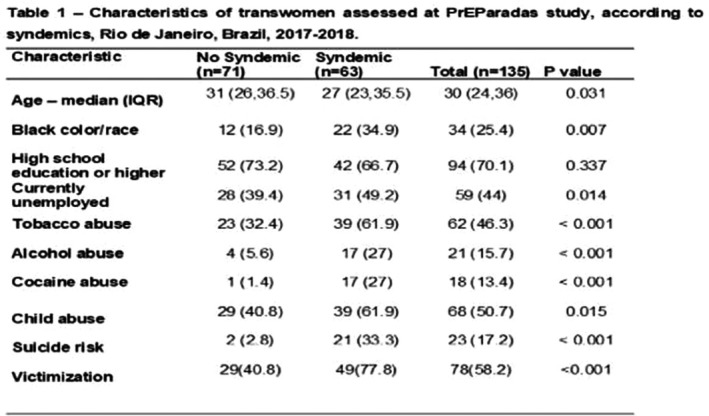

### TUAC0104

#### Regular consumption of alcohol and risk of HIV infection in transgender women in Fortaleza, Northeast Brazil

**S. Brignol^1^; I. Dourado^2^; A. Brito^3^; C.A. Davoli^4^; F. Marques de Oliveira Filho^4^; M.D.S. Cavalcanti^5^ and F.I. Bastos^6^**

^1^Universidade Federal Fluminense, department of biostatistics and epidemiology, Niterói, Brazil, ^2^Universidade Federal da Bahia, Instituto de Saúde coletiva, Salvador, Brazil, ^3^FioCRUZ‐Pernambuco, Recife, Brazil, ^4^Universidade Federal Fluminense, Instituto de Saúde coletiva, Niterói, Brazil, ^5^Fortaleza Secretary of Health, Fortaleza, Brazil, ^6^FioCruz‐RJ, ICICT, Rio de Janeiro, Brazil

**Background: **According to the Brazilian National Health Survey, the prevalence of regular alcohol consumption (RAC) is 24%. The World Health Organization warns: this level of consumption significantly impacts morbidity and mortality, increases the risk factor of HIV infection, and compromises adherence to antiretroviral treatment. Among transgender women (TW), alcohol consumption is much higher (50%‐70%) compared to the general population. In addition, TW are 13 times more likely to contract HIV compared to women in the general population. We aimed to analyse factors associated to regular consumption of alcohol among TW in Fortaleza a large capital city (2,609,716 inhabitants) in Northeast Brazil.

**Methods: **A cross‐sectional research study “Divas Research” recruited 348 TW by Respondent Driven Sampling (RDS) between November 2016‐March 2017. Participants were interviewed and the association of RAC (Daily/Weekly) during the last 3 months with HIV was analysed. The estimates were weighted using RDSII estimator and we analysed data by STATA 12 complex survey procedure. Descriptive statistics was used to describe the general characteristics and logistic regression to estimate odds ratios and 95% confidence interval.

**Results: **Among 348 TW the proportion of regular consumption of alcohol in the last three months was 44.9% (95% CI: 39.3 to 50.5); 20.5% (95% CI: 13.6 to 27.4) tested positive for HIV. The profile of women that had RAC: 55% are more 25 years old, black (15.6%) or brown (69%), depressive symptoms (92%), low income (49%), without formal employment (85%) and with low schooling (97%).The odds of HIV infection was 2.1 times higher among TGW who consumed alcohol regularly (OR = 2.1(95% CI: 1.04 to 4.2) and after adjusting have risco for RAC: smoking habit (OR = 3.0 (1.7 to 5.3)) and use of crack/cocaine (OR = 4.4 (2.1 to 9.5)).

**Conclusions: **The RAC was twice higher compared to rates of Brazilian general population. Social and programmatic vulnerability, in addition to the difficulties of accessing health services, in Brazil, increases TW exposure to violence, high alcohol consumption and other drugs. We confirmed that such consumption increases the risk of HIV infection, results suggest a high need for continuous attention to the health of transsexuals who are exposed to factor to vulnerability social. The consumption prevention of licit and illicit drugs is need.

### TUAC0105

#### Factors associated with current ART use among transwomen participating in TransAmigas study, São Paulo, Brazil

**L.F. Maschião^1^; K. Bassichetto^1^; G.S.R. Sagesse^1^; H. Gilmore^2^; M.A. Veras^1^; J. Sevelius^2^; S. Lippman^2^ and Núcleo de Pesquisa em Direitos Humanos e Saúde LGBT+NUDHES**

^1^Faculdade de Ciencias Médicas da Santa Casa de São Paulo, Saúde Coletiva, São Paulo, Brazil, ^2^UCSF, San Francisco, United States

**Background: **Transwomen face a high prevalence of HIV infection globally, affected by a syndemic of poor economic, social and mental health indicators. In Brazil, this disparity persists despite universal healthcare. In the context of 90‐90‐90 treatment targets, transwomen may experience different barriers to retention in care and antiretroviral therapy (ART) use than other groups, yet few studies of high statistical power exist to provide insight into this question. We assess factors associated with current ART use among trans women enrolled in an ongoing peer‐navigation (PN) intervention in São Paulo, Brazil.

**Methods: **TransAmigas is a 9‐month randomized pilot intervention study. Between May‐December 2018, 113 HIV positive, 18 + transwomen were randomized to control (n = 38) or peer navigation intervention (n = 75). Interviewer‐administered questionnaires were conducted at enrollment. Bivariate and multivariate Poisson regression models were employed to analyse current ART use association with sociodemographic and mental health indicators at baseline.

**Results: **The majority (84.1%) of participants saw a doctor for HIV care at least once in the previous 12 months, while 73.5% were currently in care. 79.7% had taken ART at some point, but only 63.7% were currently on treatment. In the multivariate model, Being 35 years or older (adjusted Prevalence Ratio (aPR) 2.27; CI95% 1.29 to 3.99), as well as having 12 or more years of education (aPR 1.37; CI95% 1.05 to 1.78), were associated with current ART use. K10 mental distress scale score, ethnicity, income, housing stability, legal name rectification and employment status were not significantly associated with current ART use.

**Conclusions: **Using 90‐90‐90 as a benchmark, current ART use was considerably low in our sample, which may have hindered elucidation of meaningful predictors other than older age and higher education, which have been documented in other groups. Further research, including studies with larger samples, is needed to examine the unique barriers facing this vulnerable population.

**Table:** Abstract TUAC0105‐Table 1. Multivariate correlates of current ART use among HIV+ transwomen in São Paulo, Brazil, 2018

		Current ART Use			
	No (%)	Yes (%)	aPR	*p*	CI95%
Age					
18 to 24 (ref)	15 (65.22)	8 (34.78)	1	‐	‐
25 to 34	18 (36.00)	32 (64.00)	1.74	0.065	0.97 to 3.11
35 or more	8 (20.00)	32 (80.00)	2.20	0.004	1.29 to 3.99
Education years					
Less than 12 (ref)	32 (43.84)	11 (56.16)	1	‐	‐
12 or more	9 (22.50)	31 (77.50)	1.37	0.020	1.05 to 1.78

### TUAC0201

#### Continuing low HIV incidence in the expanded pre‐exposure prophylaxis (PrEP) implementation in communities ‐ New South Wales study (EPIC‐NSW)

**A. Grulich^1^; F. Jin^1^; S. Vaccher^1^; B. Bavinton^1^; T. Vickers^1^; J. Amin^2^; C. Selvey^3^; K. Chant^3^; I. Zablotska‐Manos^4^; D. Baker^5^; M. Bloch^6^; A. Carr^7^; D. Smith^8^; R. Guy^1^ and the EPIC‐NSW study group**

^1^University of New South Wales ‐ Kirby Institute, Sydney, Australia, ^2^Macquarie University, Faculty of Medicine and Health Sciences, Sydney, Australia, ^3^NSW Ministry of Health, Sydney, Australia, ^4^Western Sydney Sexual Health Centre and Sydney Medical School‐Westmead, Sydney, Australia, ^5^East Sydney Doctors, Sydney, Australia, ^6^Holdsworth House Medical Practice, Sydney, Australia, ^7^St Vincent's Hospital, Sydney, Australia, ^8^The Albion Centre, Sydney, Australia

**Background: **Randomized trials of oral PrEP in gay and bisexual men (GBM) have reported efficacy of close to 90%, with HIV infections occurring only in non‐adherent participants. In the first 3700 EPIC‐NSW participants followed for one year, incidence was 0.51/1000 person‐years (PY) compared to an expected incidence of over 20/1000PY. Concern has been raised about long‐term PrEP efficacy. We report HIV incidence in the expanded study cohort with extended follow‐up

**Methods: **EPIC‐NSW is a population implementation study of daily oral PrEP based in 31 clinics across NSW, Australia. HIV diagnoses were reported 1) as serious adverse events, 2) through electronic medical record systems, and 3) in consenting participants (80%), by linkage to the state HIV register. Participants contributed PY from date of enrolment to date of HIV diagnosis or to 31/12/2018 in those who remained HIV‐negative. PY incidence rates and 95% confidence intervals (CIs) were calculated. Hazard ratios (HRs) were estimated using Cox regression

**Results: **9708 participants (98.5% GBM) were enrolled between March 2016 and April 2018. Over 17,747 PY of follow‐up, there were 16 HIV diagnoses, with evidence of PrEP non‐adherence in all cases. HIV incidence was 0.90/1000PY (95%CI 0.55 to 1.47). HIV incidence was higher in the younger (*p *=* *0.007), reaching 2.4/1000PY in those aged < 25. Incidence was higher in those who at baseline had either a rectal sexually transmitted infection (STI) or recent methamphetamine use, and incidence was highest in those who had both (8.93/1000PY, HR 48.9 95%CI 10.2 to 236). Incidence was not related to country of birth (*p *=* *0.100) or residing in a Sydney suburb with > 10% of males identifying as gay (*p *=* *0.615).

**Conclusions: **Over a mean approaching 2 years of follow‐up per person, PrEP remained highly effective, with incidence remaining below 1/1000PY overall. However, annual HIV incidence was about 1% in those who at baseline used methamphetamine and had a rectal STI.

**Table:** Abstract TUAC0201‐Table 1. Baseline factors predictive of HIV infection in the EPIC‐NSW study

Predictor	Level	Incidence per 1000 py	Hazard ratio	95% CI	*p*
Age	18 to 24	2.37	1	‐	0.007
	25 to 34	1.02	0.43	0.14 to 1.36	
	35 to 44	0.21	0.09	0.01 to 0.78	
	>=45	0.26	0.11	0.01 to 0.94	
Rectal sexually transmissible infection	No	0.29	1	‐	<0.001
	Yes	4.19	14.17	4.57 to 43.9	
Methamphetamine use	No	0.54	1	‐	0.002
	Yes	2.54	4.70	1.75 to 12.7	

### TUAC0202

#### Incidence of HIV‐infection with daily or on‐demand PrEP with TDF/FTC in Paris area. Update from the ANRS Prevenir Study

**J.‐M. Molina^1^; J. Ghosn^2^; M. Algarte‐Génin^3^; D. Rojas Castro^4^; L. Béniguel^3^; G. Pialoux^5^; C. Delaugerre^1^; J.‐P. Viard^6^; C. Katlama^7^; C. Segouin^8^; C. Pintado^1^; P.‐M. Girard^9^; J. Lourenco^10^; M. Ohayon^11^; S. Le Mestre^12^; B. Spire^13^; V. Doré^12^; L. Assoumou^7^; D. Costagliola^3^ and Prevenir ANRS Study Group**

^1^University Paris Diderot, Sorbonne Paris Cité, Saint‐Louis Hospital, Assistance Publique Hôpitaux de Paris, Paris, France, ^2^University Paris Diderot, Sorbonne Paris Cité, Bichat Hospital, Assistance Publique Hôpitaux de Paris, Paris, France, ^3^Institut Pierre Louis d'Epidémiologie et de Santé‐Publique, INSERM, Sorbonne Université, Paris, France, ^4^Coalition PLUS, Pantin, France, ^5^Sorbonne Université, Tenon Hospital, Assistance Publique Hôpitaux de Paris, Paris, France, ^6^Paris Descartes University, Sorbonne‐Paris‐Cité, Hotel‐Dieu Hospital, Assistance Publique Hôpitaux de Paris, Paris, France, ^7^Institut Pierre Louis d'Epidémiologie et de Santé Publique, INSERM, Sorbonne Université, Pitiè‐Salpétrière Hospital, Assistance Publique Hôpitaux de Paris, Paris, France, ^8^Lariboisière Hospital, Assistance Publique Hôpitaux de Paris, Paris, France, ^9^Institut Pierre Louis d'Epidémiologie et de Santé Publique, INSERM, Sorbonne Université, Saint‐Antoine Hospital, Assistance Publique Hôpitaux de Paris, Paris, France, ^10^IHU Imagine, Centre d'infectiologie Necker‐Pasteur, Necker‐Enfants Malades Hospital, Assistance Publique Hôpitaux de Paris, Paris, France, ^11^Centre de santé Sexuelle Le 190, Paris, France, ^12^ANRS, France Recherche Nord & Sud Sida‐HIV Hépatites, Agence Autonome de l'INSERM, Paris, France, ^13^Aix Marseille Univ, INSERM, IRD, SESSTIM, Sciences Economiques and Sociales de la Santé and Traitement de l'Information Médicale, ORS PACA, Observatoire Régional de la Santé Provence‐Alpes‐Côte d'Azur, Marseille, France

**Background: **On‐demand PrEP with TDF/FTC has been recommended as an alternative to daily PrEP for MSM by EACS and IAS‐USA guidelines, but has not been endorsed yet by WHO due to limited real‐world experience.

**Methods: **The ANRS Prevenir study is an ongoing prospective cohort study enrolling individuals at high risk for HIV infection on PrEP in Paris area. Both daily and on‐demand PrEP were offered to MSM. At baseline, month 1 and every 3 months thereafter, subjects were tested for HIV using a 4^th^ generation combined ELISA assay and other STIs and creatinine plasma levels were monitored. At each visit participants provided information regarding sexual behaviour, dosing regimen and adherence using computer assisted self‐interviews. HIV and HCV incidence were assessed as well as safety and study retention.

**Results: **From May 3rd 2017 to October 31^st^ 2018, 2143 subjects were enrolled across 22 sites in the Paris region, 56% already being PrEP user (median duration 10 months). Median age was 36 years (IQR: 30 to 44), 98.7% were MSM. At enrolment, PrEP was used daily in 46.7% and on demand in 53.3% of participants. Median number of partners in the last 3 months was 15 (IQR: 7 to 25) in the daily group and 10 (5 to 15) in the on‐demand group (*p *<* *0.001). Median number of condomless sex events in the prior 4 weeks was 2 (0 to 8) and 2 (0 to 4), respectively, (*p *=* *0.04). Current follow‐up lasted 744 and 830 person‐years (PY) in the daily and on‐demand groups, respectively. HIV‐1 incidence was 0 (95% CI: 0 to 0.5) and 0 (95% CI: 0 to 0.4) per 100 PY in the daily and on‐demand groups, respectively; and HCV incidence was 0.67 and 0.60 per 100 PY, respectively (*p *=* *0.865). Ninety‐two subjects discontinued the study during follow‐up (4.3%) and 49 (2.2%) discontinued PrEP, but only one for drug‐related adverse events (nausea/headache/dizziness).

**Conclusions: **In this ongoing PrEP cohort in Paris area, enrolling mainly MSM at high risk of HIV‐acquisition, no breakthrough HIV‐infection was reported so far in participants choosing either daily or on‐demand PrEP, supporting continuing use of both dosing regimens in this population.

### TUAC0203

#### High adherence and sustained impact on HIV‐1 incidence: Final results of an open‐label extension trial of the dapivirine vaginal ring

**J.M. Baeten^1^; T. Palanee‐Phillips^2^; N. Mgodi^3^; G. Ramjee^4^; B. Gati^5^; F. Mhlanga^3^; P. Hunidzarira^3^; L. Mansoor^6^; S. Siva^4^; V. Govender^4^; B. Makanani^7^; L. Naidoo^4^; N. Singh^4^; G. Nair^8^; L. Chinula^9^; A. Mayo^10^; D. Szydlo^11^; L. Soto‐Torres^12^; A. Nel^13^; Z. Rosenberg^14^; S. Hillier^15^; E. Brown^11^ and MTN‐025/HOPE Study Team**

^1^University of Washington, Global Health, Seattle, United States, ^2^Wits Reproductive Health & HIV Institute ^Wits RHI^, Johannesburg, South Africa, ^3^University of Zimbabwe, Harare, Zimbabwe, ^4^South African Medical Research Council, Durban, South Africa, ^5^Makerere University ‐ Johns Hopkins University Research Collaboration, Kampala, Uganda, ^6^CAPRISA, Durban, South Africa, ^7^Queen Elizabeth Central Hospital, Blantyre, Malawi, ^8^Desmond Tutu HIV Centre, Cape Town, South Africa, ^9^UNC Project Malawi, Lilongwe, Malawi, ^10^FHI360, Durham, United States, ^11^Fred Hutch, Seattle, United States, ^12^NIH, Bethesda, United States, ^13^International Partnership for Microbicides, Paarl, South Africa, ^14^International Partnership for Microbicides, Silver Spring, United States, ^15^University of Pittsburgh/Magee‐Womens Research Institute, Pittsburgh, United States

**Background: **Phase III clinical trials (MTN‐020/ASPIRE & IPM 027/The Ring Study) showed that a monthly vaginal ring containing 25 mg dapivirine was well‐tolerated and reduced HIV‐1 incidence by approximately 30% compared to placebo. This abstract presents the final results of MTN‐025/HOPE, one of the two phase IIIb open‐label extension trials of the dapivirine vaginal ring.

**Methods: **HOPE initiated in July 2016 and concluded in August 2018. HIV‐1 uninfected women who had participated in ASPIRE were offered 12 months of access to the dapivirine vaginal ring at 14 sites in Malawi, South Africa, Uganda, and Zimbabwe. Used rings were returned at each study visit (monthly for 3 months, then quarterly) and were tested for residual levels of dapivirine. HIV‐1 serologic testing was done at each visit and archived, frozen plasma samples, collected quarterly, were tested for HIV‐1 RNA to more precisely define incident infection, and infections occurring after enrollment and through the Month 12 visit were considered to have occurred on study. HIV‐1 incidence was compared to that expected by weighted bootstrap sampling of the placebo arm of ASPIRE, matched on trial site, age, and presence of a curable sexually transmitted infection at trial entry; a limitation is lack of a contemporaneous placebo group in this open‐label trial.

**Results: **A total of 1456 women enrolled into HOPE. The median age was 31 years. At baseline, 1342 (92%) accepted the dapivirine vaginal ring; ring acceptance remained high: 90%, 89%, 87%, 83%, and 79% at Months 1, 2, 3, 6, and 9. 86% of returned rings had residual dapivirine levels consistent with some use during the prior month (>0.9 mg released). A total of 35 HIV‐1 infections were observed (incidence 2.7 per 100 person‐years, 95%CI 1.9 to 3.8). Expected HIV‐1 incidence was 4.4 per 100 person‐years (95% CI 3.2 to 5.8) in the absence of access to the dapivirine vaginal ring, and an incidence of ≤ 2.7 would be expected to occur in fewer than 33 in 10,000 samplings (0.33%).

**Conclusions: **Final results from this open‐label extension trial of the dapivirine ring indicate high uptake and lower than anticipated HIV‐1 incidence in this high‐risk population.

### TUAC0204

#### What is the effect of layered prevention interventions on HIV risk among adolescent girls in Zambia?

**S. Mathur^1^; N. Pilgrim^1^; C.J. Heck^2^; S.K. Patel^3^ and M. Musheke^4^**

^1^Population Council, Washington, DC, United States, ^2^Population Council, New York, United States, ^3^Population Council, New Delhi, India, ^4^Population Council, Lusaka, Zambia

**Background: **HIV prevention efforts are increasingly addressing social/structural factors associated with adolescent girls’ HIV vulnerability. However, there is limited evidence whether interventions that go beyond the health sector can decrease HIV risk among adolescent girls in high‐incidence settings. We delineate the layered effects of social protection, education, and economic interventions on HIV‐risk among urban adolescent girls in Zambia.

**Methods: **Surveys—conducted March‐May 2018—captured knowledge, attitudes, practices, programme experiences, and HIV service uptake of 15‐ to 19‐year‐old women (n = 487) enrolled in the DREAMS programme in Lusaka and Ndola. We focus on 4 layers of programme exposure: (1) Participated in some safe space/social asset building interventions (SSI), (2) Completed all SSIs and received a certificate (SSC), (3) Completed all SSIs and received educational support (SSC+Ed), and (4) Completed all SSIs and received educational support and cash transfer (SSC+Ed+CT). Poisson regressions assess association between programme exposure and HIV‐risk outcomes (HIV knowledge, consistent condom use, transactional sex, and intimate partner sexual violence).

**Results: **Among respondents, 30% received only some SSIs, 32% completed all SSIs (SSC), 17% received SSC+Ed, and 21% received SSC+Ed+CT. There were no differences in HIV‐risk outcomes between SSI and SSC groups, except that the SSC group was more likely to engage in transactional sex [IRR:1.05 (0.74 to 1.47)]. Compared to SSI only, respondents who received the SSC+Ed were significantly more likely to have comprehensive knowledge about HIV (Incidence‐Rate Ratio [IRR:1.09, [1.02 to 1.15]) and report consistent condom use (IRR:4.80 [3.35 to 6.87]) and less likely to experience sexual violence (IRR:0.31, [0.15 to 0.65]). Similar significant findings were found for respondents receiving SSC+Ed+CT. Respondents who received SSC+Ed+CT were significantly less likely to engage in transactional sex (IRR: 0.59, [0.43 to 0.80]), compared to the SSI group.

**Conclusions: **We provide empirical evidence of the value of going beyond the health sector for HIV prevention efforts. Safe space interventions alone did not seem to influence HIV‐risk and findings around transactional sex warrant further investigation. Layering educational and economic interventions on top of safe spaces/social asset‐building activities reduced HIV‐risk among urban adolescent girls in Zambia.

**Table:** Abstract TUAC0204‐Table 1. IRRs between programme exposure and key HIV‐risk factors

	Programme uptake %	HIV knowledge IRR (95% CI	Consistent condom use IRR (95% CI)	Transactional sex IRR (95% CI)	Sexual violence IRR (95% CI)
Participated in some Safe Space interventions only (SSI)	30%	ref	ref	ref	ref
Completed all Safe Space interventions only (SSC)	32%	1.05 (0.97 to 1.13)	1.45 (0.58 to 3.59)	1.14 (1.03 to 1.27)	1.05 (0.74 to 1.47)
Completed all safe space interventions & received education support (SSC+Ed)	17%	1.09 (1.02 to 1.15)	4.80 (3.35 to 6.87)	(low power)	0.31 (0.15 to 0.65)
Completed all safe space interventions & received education support & cash transfer (SSC+Ed+CT)	21%	1.15 (1.09 to 1.20)	4.66 (4.35 to 5.00)	0.59 (0.43 to 0.80)	0.62 (0.48 to 0.80)

### TUAC0301

#### PrEP adherence and effect of drug level feedback among young African women in HPTN 082

**C.L. Celum^1^; N. Mgodi^2^; L.‐G. Bekker^3,4^; S. Hosek^5^; D. Donnell^6^; P.L. Anderson^7^; B.J. Dye^8^; S. Pathak^6^; Y. Agyei^9^; J.M. Fogel^9^; M.A. Marzinke^9^; K. Makgamathe^10^; S. Kassim^3,4^; S. Mukaka^2^; H. Noble^6^; A. Adeyeye^11^; S. Delany‐Moretlwe^10^ and on behalf of the HPTN 082 Study Team**

^1^University of Washington, Department of Global Health, Department of Medicine, Department of Epidemiology, Seattle, United States, ^2^University of Zimbabwe, College of Health Sciences Clinical Trials Unit, Harare, Zimbabwe, ^3^The Desmond Tutu HIV Centre, Cape Town, South Africa, ^4^University of Cape Town, Cape Town, South Africa, ^5^Department of Psychiatry, Stroger Hospital of Cook County, Chicago, United States, ^6^SCHARP, Fred Hutchinson Cancer Research Center, Seattle, United States, ^7^University of Colorado, Department of Pharmaceutical Sciences, Aurora, United States, ^8^FHI 360, HIV Prevention Trials Network, Durham, United States, ^9^Johns Hopkins University School of Medicine, Department of Pathology, Baltimore, United States, ^10^Wits RHI, University of Witwatersrand, Johannesburg, South Africa, ^11^National Institutes of Allergy and Infectious Diseases, National Institutes of Health, Division of AIDS, Rockville, United States

**Background: **PrEP adherence was low in efficacy trials among African adolescent girls and young women (AGYW). Adherence among African AGYW after PrEP is known to be efficacious and with additional adherence support are unknown.

**Methods: **HPTN 082 enrolled sexually‐active AGYW ages 16 to 25 in Cape Town and Johannesburg, South Africa and Harare, Zimbabwe. AGYW were randomized to standard adherence support (counselling, 2‐way SMS, and adherence clubs) or standard support *plus* drug level feedback at 2 and 3 months (M) with follow‐up at M 6, 9 and 12. Adherence was assessed by tenofovir‐diphosphate (TFV‐DP) in dried blood spots (DBS) measuring use in the prior month, and plasma tenofovir (TFV) measuring use in the prior week; high adherence is defined as TFV‐DP > 700 fmol/punch and plasma TFV > 40 ng/ml, given association with HIV protection in trials.

**Results: **427 AGYW started PrEP; median age was 21 and median VOICE risk score was 7 (score ≥ 5 associated with > 6% HIV incidence in prior cohorts). 212 AGYW were randomized to standard and 215 to drug level feedback; 74 (17%) discontinued PrEP by M12, most commonly due to pregnancy (n = 13) and participant preference (n = 19). At M3, 85% took PrEP (detectable TFV‐DP; 66% detectable TFV by plasma), and 25% had high adherence by DBS and 48% by plasma. There were no differences by arm in proportions with detectable TFV‐DP or high adherence by DBS at M3 and M6 or plasma TFV at M6 and 12 (all *p *>* *0.3). Adherence decreased significantly from M3‐M12 when visits decreased to quarterly (*p *<* *0.0001). Four acquired HIV, all of whom had undetectable plasma tenofovir at seroconversion.

**Conclusions: **Most African AGYW were taking PrEP in the first 3 months, and a substantial minority had high adherence by DBS and plasma, which was > 2‐fold higher than in PrEP efficacy trials. Adherence declined significantly from M3‐12, similar to other PrEP studies in youth. PrEP adherence did not increase with addition of drug level feedback. The four (1%) who acquired HIV were not taking PrEP. This combination prevention package that included PrEP achieved high protection. Research is needed to determine effective adherence support to sustain PrEP use among African AGYW.

### TUAC0302

#### PrEP uptake and early adherence among at HIV risk transgender women from Rio de Janeiro, Brazil: Results from the *PrEParadas* study

**E. Jalil^1^; T. Torres^1^; R.I. Moreira^1^; C. Castro^1^; L. Monteiro^1^; L. Monteiro^1^; A.C. Garcia^1^; D. Santos^1^; I. Leite^1^; M. Pedrosa^1^; V. Cattani^1^; L. Kamel^1^; C. Jalil^1^; E. Carvalheira^1^; P.M. Luz^1^; M. Gomes^1^; B. Hoagland^1^; R. Estrela^1^; P.L. Anderson^2^; A.Y. Liu^3^; S. Wagner^1^; V.G. Veloso^1^; B. Grinsztejn^1^ and PrEParadas Study Team**

^1^Fiocruz, National Institute of Infectious Diseases, Rio de Janeiro, Brazil, ^2^Skaggs School of Pharmacy and Pharmaceutical Sciences, University of Colorado Anschutz Medical Campus, Aurora, CO, United States, ^3^Bridge HIV, San Francisco Department of Public Health, San Francisco, CA, United States

**Background: **Pre‐exposure prophylaxis (PrEP) has proven efficacious for HIV prevention among transgender women (transwomen) with good adherence, but its implementation is challenging, especially in low‐and‐middle‐income countries (LMIC). We aimed to describe PrEP uptake and early adherence in *PrEParadas* study.

**Methods:** *PrEParadas* is a Brazilian trans‐specific PrEP demonstration project. Inclusion criteria were: assigned as male at birth, self‐identification as transwomen, aged 18 + years, HIV‐negative and high‐risk sexual behaviour. Procedures included HIV viral load to evaluate acute viral infection (AVI). We calculated medication possession ratio (MPR) at the week 4 post‐enrollment visit, which has been shown as a good measure for adherence. Predictors of low adherence (defined as MPR < 1) were evaluated using logistic regression model. At week 4, dried blood spots were also collected; TDF levels will be presented.

Abstract TUAC0302‐Figure 1. Flowchart of PrEParadas Study.

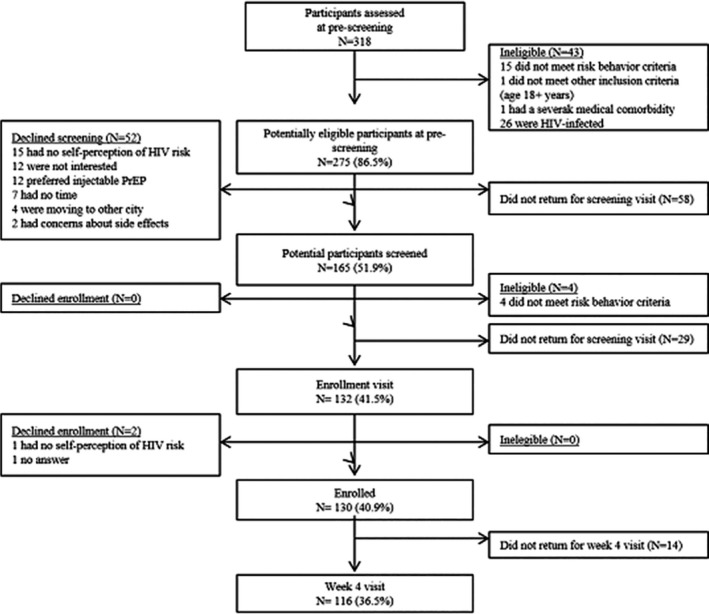

**Results: **We assessed 318 transwomen for participation; 271 were potentially eligible and 130 were offered PrEP between August 2017 December 2018 (PrEP uptake: 48.0%). Among those enrolled (N = 130), eligibility was determined by: condomless anal sex in the last 6 months (63.1%), sexually transmitted infection diagnosis in the last 12 months (24.6%), HIV‐positive partner in the last 30 days (3.9%), and transactional sex in the last 6 months (40.0%). Median age was 30 years (IQR:23 to 37). Twenty‐three (17.7%) had < 8 years of schooling. Out of 130, 89.2% returned at week 4, with no indication of AVI; only 22 transwomen had low adherence. Adjusted odds ratio indicated that transwomen with limited schooling (OR = 3.9, 95%CI:0.8 to 18.4) and those not living in own or rented housing (OR = 2.3, 95%CI:0.8 to 6.4) were more likely to have lower adherence.

**Conclusions: **Although a hard‐to‐engage group, transwomen had good uptake and adequate adherence levels in a LMIC study. More vulnerable transwomen had the worst adherence levels and deserve tailored strategies for PrEP delivery.

### TUAC0303

#### Factors associated with stopping HIV pre‐exposure prophylaxis (PrEP) for three months or more in the EPIC‐NSW trial

**B. Bavinton^1^; S. Vaccher^1^; G. Prestage^1^; M. Holt^2^; R. Guy^1^; J. Amin^3^; C. Selvey^4^; I. Zablotska‐Manos^5^; F. Jin^1^; A. Carr^6^; D. Templeton^7^; A. Grulich^1^ and The Expanded PrEP Implementation in Communities New South Wales (EPIC‐NSW) research group**

^1^Kirby Institute, University of New South Wales, Sydney, Australia, ^2^Centre for Social Research in Health, University of New South Wales, Sydney, Australia, ^3^Macquarie University, Sydney, Australia, ^4^NSW Ministry of Health, Sydney, Australia, ^5^Sydney Medical School‐Westmead, University of Sydney, Sydney, Australia, ^6^St Vincent's Hospital, Sydney, Australia, ^7^RPA Sexual Health, Sydney, Australia

**Background: **While pre‐exposure prophylaxis (PrEP) is highly effective at preventing HIV, seroconversions have been reported in individuals who cease or take a break from PrEP. We examined factors associated with stopping PrEP for ≥ 3 months among high‐risk men enrolled in the implementation trial of daily PrEP, EPIC‐NSW.

**Methods: **Between March 2016 and April 2018, 9708 individuals were enrolled. At baseline, then quarterly, participants were invited to complete an optional online behavioural survey. Analysis was restricted to participants who had completed ≥ 1 survey ≥ 18 months after enrolment. Participants indicated in the surveys whether they had taken any PrEP in the previous 3‐month period. Factors associated with stopping PrEP for ≥ 3 months were identified using generalised estimating equations controlling for repeated measurements.

**Results: **Analyses included 1682 participants; 96% were gay men. 220 participants reported stopping PrEP for ≥ 3 months (mean times per participant = 2.0, SD = 1.2; total = 327). Among these, median days to first time stopping PrEP was 494 (IQR = 122 to 591). 100 (45.5%) of these participants had at least one period of restarting PrEP after stopping for ≥ 3 months (median days stopping = 101). Stopping PrEP for ≥ 3 months was significantly associated with younger age, lower partner numbers and less condomless anal intercourse (CLAI) in the previous week (table). There was no difference in likelihood of stopping PrEP by year of enrolment. In periods where PrEP was stopped, 8% of those who stopped PrEP had > 20 partners, 30% and 15% reported party‐drug and methamphetamine use, and 52% reported any CLAI in the last week (12% reported any CLAI with unknown‐HIV‐status partners).

**Conclusions: **Participants who stopped PrEP for ≥ 3 months had lower HIV risk than those who continued use. However, over half of those who stopped reported recent HIV risk when not on PrEP, and recreational drug use was common. Greater understanding of why individuals with high HIV risk stop PrEP is needed. Individuals with varying levels of HIV risk over time may need interventions to encourage re‐uptake at the appropriate time or be encouraged to use on‐demand PrEP.

**Table:** Abstract TUAC0303‐Table 1. Associations with stopping PrEP for at least 3 months, among those in EPIC‐NSW for at least 18 months

Item	Crude Odds Ratio (95% CI)	*p*‐value	Adjusted Odds Ratio (95% CI)	*p*‐value
Reported > 20 partners in last 3 months (vs. ≤20)	0.46 (0.29 to 0.72)	0.001	0.50 (0.29 to 0.84)	0.008
Reported CLAI in the last week (vs. no CLAI)	0.58 (0.47 to 0.73)	<0.001	0.60 (0.46 to 0.79)	<0.001
Age (per year of age)	0.98 (0.96 to 0.99)	0.009	0.98 (0.96 to 1.00)	0.024

### TUAC0304

#### PrEP re‐initiation after interruption by adolescent girls and young women in Kenya and South Africa

**V. Omollo^1^; J. Odoyo^1^; D. Travil^2^; E. Rousseau^3^; L. Kidoguchi^4^; L.‐G. Bekker^3^; S. Delany‐Moretlwe^2^; J. Morton^4^; J. Haberer^5^; G. O'Malley^4^; G. Barnabee^4^; A. van der Straten^6^; R. Hefron^4^; R. Johnson^4^; C.L. Celum^4^; E.A. Bukusi^1^ and J.M. Baeten^4^**

^1^Kenya Medical Research Institute ^KEMRI^, Kisumu, Kenya, ^2^Wits Reproductive Health & HIV Institute ^Wits RHI^, Faculty of Health Sciences, University of the Witwatersrand, Johannesburg, South Africa, Johanesburg, South Africa, ^3^Desmond Tutu HIV Foundation, University of Cape Town, Cape Town, South Africa, ^4^University of Washington, Seattle, United States, ^5^Massachusetts General Hospital, Boston, United States, ^6^Women's Global Health Imperative, RTI International, San Francisco, United States

**Background: **African adolescent girls and young women (AGYW) are at high risk of HIV. PrEP is highly effective in reducing HIV acquisition, but interruptions are common. We explored PrEP re‐initiation in a PrEP implementation project among African AGYW.

**Methods: **POWER is a PrEP implementation science project among AGYW ages 16 to 25 in Kisumu, Kenya, Johannesburg and Cape Town, South Africa. Women are offered PrEP and have visits at month 1 and then quarterly. Patterns of PrEP use were measured using pharmacy records; PrEP interruption was defined as PrEP not dispensed at a visit or a gap of > 14 days without PrEP due to a missed visit. Reasons for interruptions were documented in chart notes. This analysis characterizes PrEP interruptions and re‐initiation within 6 months among AGYW who initiated PrEP at enrollment.

**Results: **Between June 2017‐November 2018, 1367 AGYW (median age 20) were enrolled. Most, (83%) were single, 28% reported never using condoms with their current partner(s), and 36% knew the HIV status of their partner(s). Most (92% 1254/1367) accepted PrEP. Of 970 women with 6 months of follow‐up after PrEP initiation, 917/970 (95%) had a PrEP interruption; most (874/917, 95%) were due to late or missed visits. Of 644 women who could have had 6 months of follow‐up after an interruption, 25% (160/644) re‐initiated PrEP: 154 after a missed/late visit, 5 after declining a refill, and 1 after a clinical hold. PrEP re‐initiations occurred within a month of interruption in 59% (median 38 days, interquartile range 26 to 57), and a higher proportion (87%) restarted within a month among those whose interruption occurred after the first 2 months of PrEP. Women reported travel and relationship dissolution as reasons for interruptions. Importantly, women sometimes did not view these periods as interruptions (e.g. intentional delays of PrEP initiation after the first prescription, attending follow‐up visits late, or periods of PrEP non‐use due to sexual abstinence).

**Conclusions: **PrEP uptake was high among African AGYW. PrEP interruptions were common, often intentional, and one‐quarter re‐initiated PrEP, typically in 1 to 2 months. In evaluating PrEP programmes, interruptions, reasons for discontinuation, and re‐initiation patterns should be monitored to assess the delivery and impact of PrEP.

### TUAD0101

#### How do HIV cure trial researchers respond to an embedded social science study?

**H. Peay^1^; S. Isaacson^2^; N. Ormsby^3^; A. Corneli^4^; R.J. Cadigan^3^ and G. Henderson^3^**

^1^1RTI International, Research Triangle Park, United States, ^2^The University of North Carolina at Chapel Hill, Gillings School of Global Public Health, Chapel Hill, United States, ^3^The University of North Carolina at Chapel Hill, Social Medicine, Chapel Hill, United States, ^4^Duke University School of Medicine, Population Health Sciences and General Internal Medicine, Durham, United States

**Background: **There are increasing calls to integrate social/behavioural studies into HIV clinical research, especially research with complex ethical and social implications such as ‘cure’ trials. Yet limited data exist on how clinician‐investigators respond to the findings of social/behavioural studies embedded in their trials.

**Methods: **As part of our longitudinal social/behavioural Decision Making Study (DMS) embedded within four HIV ‘cure’ trials in the Thai SEARCH cohort, we periodically communicate findings on the experience of trial participants. At two timepoints we conducted anonymous surveys of SEARCH clinicians to examine how DMS findings 1) concord with clinician experience, and 2) influence clinicians’ communication with participants. Findings were presented as statements; e.g., “Many trial participants feel the SEARCH team will protect them from any harms in the trial.” Survey 2 added questions about DSM study impact on participants and clinicians.

**Results: **At timepoint 1, all 18 clinicians responded; at timepoint 2, 15 of 18 responded. Concordance between each timepoint 1 DMS finding and clinician experience ranged from 33%‐67% agreement. Concordance trended higher for timepoint 2 findings. Clinicians generally rated each finding's concordance with personal experience higher than the finding's potential impact on their communication with participants; the exception was the burden of optional procedures, where respondents reported similar communication impact (M = 2.7, scale 1 to 4) and concordance (M = 2.5). Most (10 of 15) agreed DMS interviews made participants more satisfied; few (4) agreed interviews made participants worry about trial participation. While almost all clinicians (14) agreed the DMS findings made them more satisfied with how SEARCH conducts trials, 6 agreed it made them worry about trial conduct. Most (12) agreed DMS findings left them wondering what to do differently in their communication with participants.

**Conclusions: **Results suggest variation in clinician awareness of ethical challenges identified by the DMS. Most clinicians believed DMS participation did not increase worry among trial participants. DMS findings led many clinicians to wonder whether/how to communicate differently with participants, supporting the need for practical interventions to address ethical challenges. Additional research is needed to understand the influence of social/behavioural findings on clinician behaviour and enhance the integration of social/behavioural studies into clinical research.

### TUAD0102

#### Bringing good participatory practice into action: Considerations and challenges from a sponsor perspective

**H. Muller^1^; J. De Decker^1^; K. Buleza^2^; S.P. Chai^3^; A. Poretti^4^; L. Van Vijnckt^5^; T. Matthews^6^; V. Oriol Mathieu^7^; M. van Alst^8^; S. Leggewie^9^; C.A. Comeaux^7^; R. De Greef^5^; A. Zemsi^10^; M. McBride^11^; A. Colfer^11^; D. Goedhart^7^; E. van den Broecke^1^; L. Lavreys^1^; C. McShane^11^; C. Kambili^12^ and F. Tomaka^11^**

^1^Janssen Pharmaceutica NV, Beerse, Belgium, ^2^Janssen Research and Development, Philadelphia, United States, ^3^Janssen Pharmaceuticals, Selangor, Malaysia, ^4^Janssen Clinical Insights and Experience, Titusville, United States, ^5^Janssen Clinical Insights and Experience, Beerse, Belgium, ^6^Johnson & Johnson Global Public Health, Punta Pacifica, Panama, ^7^Janssen Vaccines & Prevention B.V, Leiden, Netherlands, ^8^Janssen Biologics BV, Leiden, Netherlands, ^9^Johnson & Johnson Design, New York, United States, ^10^The Bamenda Center of Health Promotion and Research, EDCTP Alumni, Bamenda, Cameroon, ^11^Janssen Research and Development, Titusville, United States, ^12^Johnson & Johnson Global Public Health, South Raritan, United States

**Background: **Good Participatory Practice (GPP) guidelines developed by UNAIDS and AVAC advise trial funders, sponsors and clinical trial implementers how to engage with stakeholders in the design and conduct of clinical HIV‐prevention trials. Anticipated outcomes of GPP implementation are a more engaged community, better trial acceptance/inclusion and increased participant retention. To our best knowledge, since 2016, Janssen (in its role as a sponsor) is the first pharmaceutical company to design an operational framework to implement GPP in sponsored trials.

**Methods: **A working group developed a ‘GPP Considerations Document’ providing high‐level guidance to trial teams on the ‘WHAT’ per GPP topic areas outlining action plans, roles and responsibilities. Complementing this, a ‘GPP‐Toolbox’ guided implementers on the ‘HOW’. We applied GPP to three ongoing trials.

**Results: **From a pharmaceutical‐sponsor's perspective, meaningful adoption of GPP in global programmes is challenging. It is a new way of working that implies internal/external stakeholder mapping and awareness, followed by change management, training, guidance and follow‐up. Implementation in Janssen's trials required employee training on the guidelines. The ‘Introduction to GPP’ training module is accessible to employees and GPP awareness has been enhanced in teams working on infectious diseases and vaccine clinical trials. Several people obtained AVAC‐certification.

Initial steps included enhanced stakeholder engagement. Unlike the usual approach, Janssen engaged more directly with study participants via letters informing them of study status, and sharing our appreciation for their efforts. Study results were shared more frequently and earlier with investigators and participants through talking points provided to sites. We designed participant engagement tools and long‐term follow‐up according to each country's/site's needs. HIV testing and counselling was provided to manage Vaccine‐Induced Seroreactivity/Positivity.

**Conclusions: **Through meaningful planning and implementation of GPP within the HIV‐prevention field, Janssen is contributing to a deepened awareness of the value of GPP; not only to hold trial conduct to a higher ethical standard, but to truly understand how best to serve communities through the research process and ultimate delivery of new prevention methods. More extensive GPP implementation is underway in Janssen's HIV vaccine programme. These practices could benefit other clinical research outside of HIV‐prevention, thus elevating and broadening standards of engagement.

### TUAD0103

#### Getting from good participatory practice to good trial outcomes for everyone: How stakeholders believe GPP works (or not)

**K. MacQueen and N. Eley**

FHI 360, Global Health Research, Durham, United States

**Background: **Many claims are made about the benefits of using Good Participatory Practice (GPP) in biomedical trials but no systematic outcome evaluations exist. Indeed, we do not know the extent to which diverse stakeholders share common expectations about how GPP works. We used online surveys to develop a conceptual map of stakeholder beliefs about GPP.

**Methods: **We used existing contacts, publicly available research networks, and social media to recruit biomedical research stakeholders at community (n = 111), national (n = 41) and global (n = 41) levels to participate in a series of online surveys November 2017 to January 2019. Survey 1 used Maximum Difference Scaling to identify high priority GPP goals derived from the literature. Survey 2 asked participants to describe practices they believed could be used to achieve these goals; these were consolidated into broader strategies. Survey 3 asked participants to describe good and bad outcomes they believed could result from using the strategies. Surveys 4 & 5 assessed levels of agreement with the emerging model.

**Results: **Survey panelists identified as 65% female, 34% male, and 1% transgender. There was strong agreement that the highest priority GPP goals were community‐focused and emphasized protecting, engaging and empowering communities where biomedical prevention research takes place. Five strategies to achieve these goals were identified. Accountability, Context Mapping, and Continuous Community Engagement were believed to reinforce engagement & communications; knowledge, learning & understanding; awareness & transparency; and trust. The remaining two strategies, Maximizing Benefit and Minimizing Risk & Burden were believed to reinforce researchers valuing community expertise, being knowledgeable about participants, and being transparent which led to increased participant trust. Unintended bad outcomes were believed possible if local partners became insular or burdens on researchers too costly. Specific and potentially measurable outcomes were identified with relevance for researchers, participants, and communities.

**Conclusions: **The conceptual map provides a foundation for developing a mixed method systematic evaluation strategy to better understand and improve GPP in HIV research. Beliefs about relationships between strategies and good/bad outcomes can be translated into hypotheses, measures, and indicators as well as context‐rich comparative analysis of cases.

### TUAD0104

#### The game‐changing nature of early and ongoing community engagement in HIV prevention efficacy trials: The AMP studies’ experience (HVTN 703/HPTN 081 & HVTN 704/HPTN 085)

**M. Andrasik^1^; G. Broder^1^; J. Lucas^2^; J. Davis^2^; R. White^2^; N. Luthuli^3^; K. Baepanye^3^; L. Oseso^1^; S. Wallace^1^; N. Ennis^1^; C. Shipman^1^; S. Karuna^1^; P. Andrew^2^; S. Edupuganti^4^; N. Mgodi^5^; J. Andriesen^1^; M. Cohen^2^; L. Corey^1^ and AMP Protocol Teams**

^1^Fred Hutch, HIV Vaccine Trials Network, Seattle, United States, ^2^FHI 360, Science Facilitation Department, Durham, United States, ^3^Fred Hutch, HIV Vaccine Trials Network, Johannesburg, South Africa, ^4^Emory University, Hope Clinic of Emory Vaccine Center, Atlanta, United States, ^5^Seke South Clinic, Department of Health Services, Harare, Zimbabwe

**Background: **Engagement of community stakeholders is essential to the facilitation of community awareness, understanding, and support for research from conceptualization through retention. Recognizing the stigma surrounding HIV and misconceptions about research, ongoing community engagement is especially important for studies of novel biomedical HIV prevention products.

In 2015, the HIV Vaccine Trials Network (HVTN) partnered with the HIV Prevention Trials Network (HPTN) to launch the AMP trials among heterosexual women in sub‐Saharan Africa (HVTN 703/HPTN 081) and cisgender men and transgender persons who have sex with men in North and South America and Switzerland (HVTN 704/HPTN 085). These are the first efficacy trials of a broadly neutralizing antibody (bnAb) for HIV prevention. Recruitment began in April (HVTN 704/HPTN 085) and May (HVTN 703/HPTN 081) of 2016. Recruitment and retention were considered major potential barriers for these ongoing trials, which require 2 years of monthly visits and 10 intravenous infusions per participant.

**Methods: **Community engagement training and implementation began 6 months prior to opening. A series of stakeholder engagement consultations were held to facilitate information exchange and encourage dialogue with in‐country ethics, advocacy, spiritual, healthcare and research representatives. A Community‐Based Participatory Research approach was utilized to develop and disseminate a set of four educational videos explaining the studies, bnAb science, and trial participation. Print materials and a website were created for each geographic region to educate and direct potential participants to local study sites. Retention workshops and ongoing communication with the studies’ Community Working Groups maintain attention on retention.

**Results: **Full study enrollment exceeded projected rates. Recruitment was efficient, with a screening‐to‐enrollment ratio of roughly 2.8:1 in the Americas and Switzerland and 2.4:1 in Africa at selected timepoints. As of January 16, 2019, retention in the AMP studies is 96% in HVTN 703/HPTN 081, 95% in HVTN 704/PHTN 085; early termination is 8% overall. There was tremendous community enthusiasm regarding the overall bnAb concept for HIV prevention.

**Conclusions: **Community engagement is critical for ethical trial conduct and must be employed in its entirety (education, recruitment and retention). Early and consistent integration throughout the clinical trial process contributes to improved screening, substantial recruitment, and strong retention.

### TUAD0106

#### Engaging youth as long‐acting HIV prevention product co‐researchers in the iPrevent study in Cape Town, South Africa

**M. Hartmann^1^; A. Minnis^1^; E. Krogstad^1^; S. Ndwayana^2^; S. Sindelo^2^; M. Atujuna^2^; L.‐G. Bekker^2^ and E.T. Montgomery^2^**

^1^RTI International, San Francisco, United States, ^2^The University of Cape Town, The Desmond Tutu HIV Centre, Cape Town, South Africa

**Background: **South African youth are one of the highest risk groups, globally, for HIV acquisition, thus identifying prevention methods that they will consistently use is an urgent priority. iPrevent sought to understand preferences for long‐acting (LA) PrEP among youth 18 to 24 in Cape Town recognizing this group as critical “end‐users” of future LA methods. Undertaking multiple strategies, the study established an advisory board of young heterosexual men, women and men‐who‐have‐sex‐with‐men to contribute as product co‐researchers throughout the study process, to ensure effective engagement of the population under study.

**Methods: **The iPrevent study implemented a large (n = 809) community‐based discrete choice experiment (DCE) survey assessing attributes of LA‐PrEP with young women and men, including MSM. Through a series of four youth advisory board meetings, a WhatsApp group and 20 cognitive interviews, iPrevent's participatory approach to engaging youth as co‐researchers included giving them opportunities as film‐makers, as designers of the visual component of the DCE, and as interpreters of the results.

**Results: **Convening youth as co‐researchers had several impacts on iPrevent's approach and outputs. Youth input informed the use of local actors in the study's educational video creating a “real‐world” community setting that situated the dialogue and content in a meaningful and accessible way. Their participation in cognitive interviews led to the successful development of language and images to explain scientific concepts in terms that would resonate (e.g., chili peppers to express pain associated with product insertion). Lastly, their insight reviewing results during an interpretation meeting, led to clarifications around misinterpretations of risk perception and confirmed youth's desires for future LA products that fit within their goals around fun, family, and their future. The findings from this end‐user interpretation meeting were shared with developers of HIV prevention products at an international conference.

**Conclusions: **The engagement of youth through creative, interactive activities to inform film‐making, research tool design, and results interpretation directly contributed to adaptations of the study design and to research implementation and understanding of results. This was important for connecting with young end‐users and translating the DCE findings to LA product developers in a way that reflected the context of youth's lives.

### TUAD0201

#### Modelling combination interventions including increased school attendance to prevent HIV among girls of school age in South Africa (HPTN 068)

**M. Stoner^1^; J. Ahern^2^; K. Kahn^3,4^; R. Twine^3^; F.X. Gomez‐Olive^3,4^; S. Lippman^3,5^ and A. Pettifor^1,3,6^**

^1^University of North Carolina at Chapel Hill, Carolina Population Center, Chapel Hill, United States, ^2^University of California, School of Public Health, Division of Epidemiology, Berkeley, United States, ^3^University of the Witwatersrand, Johannesburg, South Africa, MRC/Wits Rural Public Health and Health Transitions Research Unit ^Agincourt^, School of Public Health, Faculty of Health Sciences, Johannesburg, South Africa, ^4^INDEPTH Network, Accra, Ghana, ^5^University of California, Department of Medicine, San Francisco, United States, ^6^University of North Carolina at Chapel Hill, Department of Epidemiology, Chapel Hill, United States

**Background: **Combination prevention interventions may be an effective way to prevent HIV in adolescent girls and young women. However, combinations require enormous resources to implement and evaluate. Modelling strategies are a cost‐effective way to determine what might work before implementation. We modelled HIV prevention interventions including 1) staying in school or attending 80% or more school days; 2) intervention 1 plus eliminating depression; 3) intervention 1 plus reducing depression by 50%; 4) intervention 2 plus eliminating physical intimate partner violence (IPV) in the last 12 months; and 5) intervention 3 plus reducing IPV by 50%.

**Methods: **We used data from the main trial period of the HIV Prevention Trials Network (HPTN) 068 study from 2011 to 2015 when girls were of school age. Our study includes 2328 HIV negative adolescent girls and young women aged 13 to 20 years in rural South Africa. We used the parametric G‐formula where we simulated a population of 10,000 girls from observed data in which confounding from observed covariates was removed. Confounders were age, intervention assignment, socioeconomic status, alcohol use, anxiety, and orphanhood. We then manipulated variables in this population and determined how these changes corresponded with changes in the cumulative incidence of HIV over the study period. Interaction terms were included between all exposures. Confidence intervals were calculated using the standard deviation of results from 200 bootstrap samples with replacement.

**Results: **The observed risk of HIV over 3.5 years was 4.5%. School attendance, depression and IPV were each independently associated with incident HIV infection. Compared to the observed risk of HIV (4.5%), HIV risk was 4% for intervention 1 (Risk Ratio (RR) 0.90; 95% Confidence interval (CI): 0.84, 0.96; 3.5% for intervention 2 (RR 0.77; 95 % CI: 0.70, 0.84); 3.9% for intervention 3 (RR 0.87; 95% CI: 0.80, 0.94); 3.4% for intervention 4 (RR 0.75; 95% CI: 0.69, 0.82); and 3.6% for intervention 5 (RR 0.80; 95% CI: 0.75, 0.86).

**Conclusions: **Combination prevention interventions that include increasing school attendance, reducing depression and reducing IPV may be an effective way to reduce risk of HIV in adolescent girls and young women in South Africa.

### TUAD0202

#### Improving uptake of prevention of mother‐to‐child HIV transmission services in Benue State, Nigeria through a church congregation‐based approach

**M. Montandon^1^; T. Efuntoye^2^; J. Oko^3^; C. Onyenuobi^2^; C. Onwuchekwa^3^; R.W. Shiraishi^1^; J. Gwamna^2^; A. Abutu^1^; A. Schwitters^2^ and M. Swaminathan^2^**

^1^US Centers for Disease Control and Prevention, Atlanta, United States, ^2^CDC‐Nigeria, Abuja, Nigeria, ^3^Catholic Caritas Foundation of Nigeria, Abuja, Nigeria

**Background: **Nigeria has low antiretroviral therapy (ART) coverage among HIV‐positive pregnant women, partially due to low uptake of facility‐based antenatal care. In a previous cluster randomized trial in Nigeria, a church congregation‐based intervention (also called Baby Shower events) demonstrated improvement in HIV testing and linkage of pregnant women to ART. In this project, we assess outcomes for HIV testing and ART linkage using the congregational approach to prevention of mother‐to‐child HIV transmission (PMTCT) in a non‐research setting.

**Methods: **Seventy‐seven congregations in Benue State conducted 630 Baby Shower events from July 2016‐December 2017. Baby Shower events included a prayer ceremony, group education, music, gifting of a delivery pack, and HIV, Hepatitis B, and sickle cell testing with subsequent ART linkage support for HIV‐positive participants. De‐identified data were collected on participant demographics, pregnancy and HIV testing history, test results and linkage to ART. Frequencies and proportions were summarized for participant characteristics, HIV testing uptake and yield, partner testing results, and ART linkage.

**Results: **Over the implementation period, 9510 pregnant women and 5650 male partners participated in the Baby Shower events (56.4% male participation). The median age of participants was 24 years (IQR 20, 28) for females and 30 years (IQR 25, 37) for males. Nearly half of female participants (45%) were not enrolled in antenatal care for the current pregnancy, and 22% and 23% of female and male participants respectively reported that they had never been tested for HIV. Overall, 9498 (99.9%) female and 5639 (99.8%) male participants had their HIV status ascertained, with 7% of female and 4% of male participants testing HIV‐positive, 2.8% of females and 2.2% of males receiving a new HIV‐positive diagnosis, and 5.5% of couples receiving discordant HIV results. The majority of HIV‐positive pregnant women (83.5%, 581/696) were confirmed linked to antiretroviral therapy.

**Conclusions: **In this setting of low PMTCT/ANC uptake and strong church influence, the congregational approach facilitated identification of HIV‐positive pregnant women and male partners, many of whom were not engaged with facility‐based care. Future implementation should incorporate enhanced support for ART linkage and treatment retention in order to maximize the impact of this intervention on vertical HIV transmission.

### TUAD0203

#### Does early infant male circumcision increase mothers’ attendance for postnatal care services? Findings from Iringa and Njombe Regions, Tanzania

**M. Machaku**

Jhpiego Tanzania, MERL, Dar es Salaam, Tanzania, United Republic of Tanzania

**Background: **The WHO and UNICEF recommend early infant male circumcision (EIMC) as a strategy to maintain male circumcision coverage in areas with high HIV prevalence. he USAID‐funded AIDSFree Project and the Tanzania Ministry of Health Community Development Gender Elderly and Children scaled up EIMC services integrated into existing reproductive health services.

The home delivery rate of infants are 2%, 3% and 12% in Iringa, Njombe and Tabora, respectively. Parents who give birth at home may not be educated on the benefits of EIMC which generally occurs in facilities. Therefore, through project volunteers, parents are educated on the benefits (and risks) of EIMC. We explored the uptake of EIMC services on women who delivered at home, who were educated in the community, and whose infants accessed postnatal care within 60 days post‐delivery.

**Methods: **We conducted chat review of the client‐level EIMC database from October 2016 to September 2018 from 38 EIMC facilities and 38 non‐EIMC facilities was conducted to determine the effect of EIMC programme on increasing number of PNC visits out of home deliveries. We used two‐population z‐test to compare the increase of PNC services between VMM facilities and non‐VMMC facilities.

**Results: **Out of 8748 Infants attending PNC, (3.4%) 298 were delivered at home and came for PNC through EIMC and out of 61,931 Infants attending non‐EIMC (1%) 713 were delivered at home. Therefore, Infants delivered at home from EIMC communities were 3.4 times more likely to come for PNC visits compared to Infants delivered at home from non‐EIMC communities (*p *<* *0.0001, z = 18.97). Infants born at home that came for EIMC received the missed PNC services.

**Conclusions: **The opportunity provided by EIMC services enabled 298 women who delivered at home to access postnatal care services that may have been missed. Postnatal care services are vital to mitigate risks of maternal and neonatal mortality in Tanzania. Therefore, advocating and scaling up EIMC services and ensuring them are linked to reproductive health services can be a catalyst to attract and subsequently offer, postnatal care services.

### TUAD0204

#### Condom and oral PrEP use among female sex workers: Findings from a study in South Africa

**P. Shamu^1^; D. Pillay^1^; S. Jenkins^2^; M. Murire^1^; K. Stankevitz^3^; M. Lanham^3^; K. Ridgeway^3^ and S. Mullick^1^**

^1^Wits Reproductive Health & HIV Institute ^Wits RHI^, Faculty of Health Sciences, University of the Witwatersrand, Johannesburg, South Africa, ^2^Clinton Health Access Initiative ^CHAI^, Pretoria, South Africa, ^3^FHI 360, Durham, United States

**Background: **Simultaneous use of oral PrEP and condoms may be a challenging behavioural aspect of PrEP. However, little data exists on the simultaneous use of PrEP and condoms in real world settings. This abstract aims to contribute to this knowledge gap.

**Methods: **We administered a cross‐sectional survey to female sex workers (FSW) aged 18 and above at nine facilities offering PrEP, followed by in‐depth interviews (IDIs). Condom use at last sex was assessed for current, past and never users of PrEP in different sexual relationships (main or casual partner, client). Condom use at last sex is a proxy for condom use over time. We summarized data using descriptive statistics.

**Results: **We enrolled 156 self‐identified FSW (57 current, 43 past, 56 never users). In surveys, over 80% said that they used a condom the last time they had sex with a client; these proportions were similar among current (87%), past (86%), and never (86%) users. Among those with main (n = 85) or casual (n = 64) partners, condom use was higher with casual partners overall, and was higher for never (77% casual/54% main) and current users (70% casual/38% main) compared to past users (53% casual/24% main). Condom use was lowest with main partners, and in IDIs some FSW described that in steady relationships it was challenging to use condoms. Most current users felt it was easy to use PrEP and condoms simultaneously with main partners (90%) and clients (95%). However, in IDIs many noted that clients removed condoms and offered more money to “trick” or “tempt” participants into having sex without them, which could explain why, when asked which method worked better for them (condoms, PrEP or both), 70% of current users preferred both methods. However only 19% of past users preferred both, and 72% preferred condoms alone.

**Conclusions: **Current users seem to be able to use condoms and PrEP simultaneously. However, low condom usage with main partners is worrisome as this may potentially see a rise in sexually transmitted infections and unwanted pregnancies for those women not on contraceptives. Therefore, simultaneous use of PrEP and condoms should be encouraged.

### TUAD0205

#### Improving prevention choice while we wait for an HIV vaccine: Prioritizing resources for key population‐specific prevention research and implementation

**F. Riaz^1^; K. Segal^1^; K. Fisher^1^; M. Warren^2^ and L. Fitch^3^**

^1^AVAC, Policy and Data Analytics, New York, United States, ^2^AVAC, New York, United States, ^3^AVAC, Product Introduction and Access, New York, United States

**Background: **Since 2004, the Resource Tracking for HIV Prevention Research and Development (R&D) Working Group (AVAC, IAVI & UNAIDS) has tracked resource trends in R&D for new HIV prevention options. A 2018 UNAIDS report found that 47% of new HIV infections globally, or 850,000 in 2017, were among key populations and their sexual partners. A licensed HIV vaccine effective in all populations—even if current candidates in trials prove effective—is at least five years away.

**Methods: **For 2017, the Resource Tracking Working Group tracked R&D investment in HIV prevention, and AVAC tracked oral PrEP implementation through the Prevention Market Manager Global PrEP Tracker. Data were collected on annual global disbursements for product development, clinical trials and trial preparation, community education, and policy advocacy to estimate annual non‐vaccine investment in HIV prevention R&D directed specifically toward key populations. Funding was categorized based upon grant descriptions of work pertaining to men who have sex with men (MSM), sex workers, transgender people, and people who inject drugs (PWID).

**Results: **In 2017, of $228 million invested globally in microbicides, pre‐exposure prophylaxis (PrEP), and treatment as prevention (TasP) R&D, $14.6 million, or 6%, was devoted to research expressly directed toward key populations. This represents 2.3% of funding for research on microbicides, 17% for PrEP, and 4% for TasP. Among key populations, out of a global HIV non‐vaccine prevention research portfolio of $281 million, $3.5 million was allocated to prevention research for transgender people, $1.8 million for PWID, $0.6 million for male sex workers, $8.7 million for MSM, of which $2.5 million was allocated for Black or Latino MSM.

**Conclusions: **The level of investment in key population‐specific prevention research suggests the field is not sufficiently resourcing the necessary intersection of biological risk, preferences, and social determinants that affect uptake and effectiveness of new prevention options. Research in key populations is critical to inform development of prevention interventions with positive public health impact, effectively engage civil society in product development, and implement human‐centered design research, yet it remains an underfunded and underutilized tool.

### TUAD0301

#### Do changes in development assistance for health crowd out domestic investment for health and what are the implications for HIV/AIDS outcomes

**B. Patenaude**

Johns Hopkins University Bloomberg School of Public Health, International Health, Baltimore, United States

**Background: **Much of the previous research on the relationship between development assistance for health and domestic health spending utilizes standard panel data regression approaches or relatively weak instrumental variables. These methods have made handling simultaneity and omitted variable bias difficult. In this study I utilize causal time series and panel techniques as well as a new data source to examine the interplay between development assistance and domestic health spending, both public and private. I then translate these dynamics into impacts on health outcomes including HIV prevalence, tuberculosis incidence, and mortality.

**Methods: **I merge data from the Institute for Health Metrics and Evaluation's (IHME) Development Assistance for Health dataset and Health Expenditure dataset with economic, demographic, and health outcomes data from the World Bank Databank and World Health Organization's Global Health Observatory from 2000 to 2015. The final dataset consists of 237,656 individual donor transactions from 30 source countries through 42 bilateral, multilateral and private channels, to 174 distinct recipients. To address the shortcomings of previous research, I employ a fixed‐effects Arellano‐Bond system generalized methods of moments model to assess the impact of changes in the level of development assistance for health on both domestic public and domestic private health spending. I also adapt the multi‐level systems approach to determine the impact of these dynamics in development assistance on total mortality, HIV prevalence, and TB incidence.

**Results: **The results demonstrate that a 1% increase in Development Assistance for Health results in a 0.011% (SE: 0.002) increase in private health expenditure, a 0.009% (SE: 0.001) decrease in public health expenditure and a 0.004% (SE: 0.001) decrease in out‐of‐pocket expenditure. Additionally, a 1% increase in development assistance for health also has an impact on decreasing overall mortality by 0.002% (SE: 0.0005), decreasing tuberculosis incidence by 0.8% (SE 0.24), and decreasing HIV prevalence by 0.011% (SE: 001).

**Conclusions: **Between 2000 and 2015 development assistance for health has mildly crowded out domestic public health investment. However, development assistance has also crowded out out‐of‐pocket expenditure and crowded‐in domestic private health investment. These dynamics in development assistance have translated to reducing the incidence of TB, prevalence of HIV/AIDS, and mortality.

### TUAD0302

#### From “nice‐to‐have” to “necessary”: Increases in domestic financing and perceived value of key population‐lead HIV services by the Thai government as international donor funding transitions

**R. Vannakit^1^; J. Thammatach‐aree^2^; K. Rungtanatada^2^; D. Linjongrat^3^; S. Janyam^4^; P. Chanlearn^5^; R. Reankhomfu^6^; T. Nakpor^7^; S. Mills^8^; S. Charoenying^8^; K. Benjamaneepairoj^8^; P. Na Nakorn^9^; M. Sanguankwamdee^10^; N. Phanuphak^9^ and P. Phanuphak^9^**

^1^United States Agency for International Development, Regional Development Mission, Asia, Bangkok, Thailand, ^2^National Health Security Organization, Thai Ministry of Public Health, Bangkok, Thailand, ^3^Rainbow Sky Association of Thailand, Bangkok, Thailand, ^4^SWING, Bangkok, Thailand, ^5^Mplus Foundation, Chiang Mai, Thailand, ^6^Caremat, Chiang Mai, Thailand, ^7^Sisters, Pattaya, Thailand, ^8^FHI 360 and U.S. Agency for International Development LINKAGES Project, Bangkok, Thailand, ^9^Thai Red Cross AIDS Research Center, Bangkok, Thailand, ^10^United States Agency for International Development Regional Development Mission, Asia, Bangkok, Thailand

**Background: **The US Presidents Emergency Plan for AIDS Relief (PEPFAR) and The Global Fund for AIDS, Tuberculosis, and Malaria (GFATM) have been the predominant financers of key population‐led HIV services (KP‐LHS) in Thailand (and many other countries) for over 10 years. Data from multiple sites in Thailand suggest that KP‐LHS focusing on men who have sex with men (MSM) and transgender women (TGW) have contributed substantially to national 90‐90‐90 goals. Given the upper‐middle‐income development status of Thailand, the funding for these interventions needs to transition from international donor support to domestic financing.

**Methods: **The National Health Security Organization (NHSO) of the Thailand Ministry of Health provides funding to hospitals through reimbursement schemes. In 2016, the Ministry of Public Health and NHSO developed reimbursement schemes to pay CSOs for HIV‐related outreach to key population groups. However, these planned schemes resulted in little actual reimbursements because of lack of understanding by CSOs of NHSO system requirements and a lack of perceived value by numerous stakeholders of the role of KP‐LHS in HIV epidemic control in Thailand. To counter these barriers, leaders from local KP‐LHS organizations and other partners including the Thai Red Cross AIDS Research Centre engaged in advocacy efforts with central and regional NHSO offices, hospitals, and provincial health offices. NHSO and CSOs also had numerous joint sessions to clarify NHSO submission requirements for reimbursements.

**Results: **Advocacy efforts led to increased investments by the NHSO in local CSOs who provide these services. As shown in Table 1, five large local CSOs ‐ SWING, Rainbow Sky Association of Thailand, MPLUS, Caremat, and SISTERS ‐ all received increased investments by the NHSO from 2016 to 2019. Overall domestic funding investments in these institutions increased from US$ 167,000 in 2016 to a planned and contracted US$ 1.44 million in 2019.

**Conclusions: **With these domestic financing investments, Thailand joins only a few middle‐development countries who are substantially supporting critical KP‐LHS that can lead to epidemic control among MSM and TG. These investments resulted from hard won advocacy efforts on the part of key population leaders and their allies with a ready‐to‐listen‐and‐act Ministry of Public Health.

### TUAD0303

#### Study comparative NASA and budget tracking: Enabling and empowering the local budget and resource allocation in Bandung district

**S. Dewi^1,2^; B. Prabowo^3^; Y. Mulyana^4^; O. Danial^5^; B. Rahmat^6^; R. Prawira Nagara^7^; I. Yudha^2^; A. Siregar^2^; R. Verita^8^; R. Hadiana^8^ and S. Darsiti^9^**

^1^Bandung District AIDS Commission, Bandung, Indonesia, ^2^Universitas Padjadjaran, Economy, Bandung, Indonesia, ^3^Bandung District AIDS Commission, Secretary, Bandung, Indonesia, ^4^Vice Mayor Bandung City, Government, Bandung, Indonesia, ^5^Mayor Bandung City, Government, Bandung, Indonesia, ^6^Bandung District AIDS Commission, Health, Bandung, Indonesia, ^7^Universitas Padjadjaran, Health, Bandung, Indonesia, ^8^Goverment, Health, Bandung, Indonesia, ^9^Goverment, Sosial welfare, Bandung, Indonesia

**Background: **Compared to another city, Bandung has the highest HIV and AIDS cases in West Java Province, with the trend of the new case is increasing continuously. Currently, many external donors emerge their programme, but sustainability and ownership of the programme remain questionable. Empowering the budgeting programme from local fund resources was needed as an effort to ensure the prevention and control programme. This study aims to track the previous and current budget expenditure and generate future costing for preparation of local fund.

**Methods: **We analysed donor and local government financing using evidence from National AIDS Spending (NASA) and district reports on domestic HIV spending. A literature search conducted for peer‐reviewed and grey literature on HIV related costing and financing published between 2007 and 2017. We estimated the costs of five service delivery models:prevention and health promotion, mitigation diagnosis and treatment, management and administration, and enabling conducive environment.

**Results: **Prevention and mitigation programme acts as the most significant component of the overall funding allocation (61.73% and 24.20%) with average value needed is USD 313,340 (SD = 62,791) and USD 122,844 (SD = 9517). Current management and administration allocation are appropriate (13.10%) with average value of USD 66,493 (SD = 18,684). Diagnostic and treatment are among the lowest allocation (0.39%) with average value of USD 5889 (SD = 1601) due to the ARV drug procurement are regulated by central government. Budget allocation for enabling conducive environment is among the lowest (0.59%) with average value of USD 2985 (SD = 1488).

**Conclusions: **Financial investment for prevention is the largest allocated for the available fund with a big gap to others service delivery. The local government need more fund allocation in diagnostic and enabling conducive environment to ensure sustainability and ownership of HIV programme and allow empowering of budget and resource allocation in Bandung District.

### TUAD0304

#### Exploring the unit cost variation of Mexico's HIV treatment programme: A facility‐level longitudinal analysis

**D. Cerecero; C. Pineda‐Antunez; F. Macías and S. Bautista‐Arredondo**

Instituto Nacional de Salud Publica, Cuernavaca, Mexico

**Background: **The efforts for scaling up HIV prevention and treatment programmes have increased in the last decade as well as the need for accurate cost information in a timely fashion. In moments of critical decision‐making, decision makers need to understand how programme costs vary at different implementation settings and facility characteristics. The aim of this work is to explore the variation of HIV treatment costs in Mexico using data on costs and outputs for the period 2010 to 2015.

**Methods: **We obtained data from administrative records to measure costs for three main inputs: personnel, ARV drugs, and laboratory tests ‐ CD4 and viral load. We estimated the total and unit cost ‐ defined as the facility‐level average annual cost per patient ‐ of providing HIV treatment services for a sample of 74 facilities in the period 2010 to 2015. We conducted OLS regression modelsto estimate theeffect of the determinants ofunit cost variation across sites.

**Results: **The average unit cost for HIV care and treatment in the period 2010 to 2015 was USD $4012 and on average 63,725 patients were served in the study period. We observed an increase of the unit cost from USD $3950 in 2010 to USD $4285 in 2015. The average cost composition in the study period was 60% ARV drugs, 27% personnel, and 13% laboratory tests. Our regression model showed non‐linearities with respect to the number of patients served in the study period. Also, rural facilities and those located in the south region of Mexico showed significant determinants of lower unit costs. We also found that patient characteristics such as age, and clinical outputs such as the number of visits and viral suppression were predictors of HIV treatment costs.

**Conclusions: **We found an increase in the cost of HIV treatment services in the study period, as well as an increase in the number of patients served. We also found evidence consistent with economies of scale. This is the first study conducted in Mexico using longitudinal data on cost for HIV treatment services. Our results can help policy‐makers to more efficiently allocate resources to scale up HIV treatment programmes in Mexico.

### WEAA0101

#### Decreased ex vivo T cell proliferation and increased immune exhaustion in early treated and long‐term suppressed HIV‐infected pre‐adolescents from the CHER trial: Implications for cure strategies

**S. Naidoo^1^; T. Maponga^1^; K. Veldsman^1^; B. Laughton^2,3^; M. Cotton^4^; R. Glashoff and HIV Reservoir Study Group**

^1^Stellenbosch University, Division of Medical Virology, Cape Town, South Africa, ^2^Stellenbosch University, FAMCRU, Cape Town, South Africa, ^3^Tygerberg Children's Hospital, Cape Town, South Africa, ^4^Stellenbosch University, Medical Microbiology and Immunology, Cape Town, South Africa

**Background: **Suppressive long‐term ART is accompanied by several immune aberrations that are not normalized even with early therapy. The extent to which continuing immune dysfunction exists in HIV infected, long‐term treated South African pre‐adolescents, requires delineation. Understanding the role dysfunctional immune mechanisms play in HIV persistence is required to develop additional novel therapeutic targets for alleviation of immune‐mediated damage.

**Methods: **Samples originated from the Children‐with‐HIV‐Early‐Antiretroviral (CHER) trial. ART was initiated at < 1 year with sustained viral suppression after 8 years. The frequency of CD4 + and CD8 + T‐cells expressing cellular immune markers of activation (CD38, CD69, HLA‐DR), proliferation (Ki67) and exhaustion (PD‐1, Tim‐3, TIGIT, LAG‐3) was measured by flow cytometry. An extensive panel of T‐cell related plasma cytokines was evaluated using Luminex^®^Multiplex assays. Age‐matched controls were measured for the same biomarkers. A subset of HIV+ participants was tested for HIV‐1 DNA using qPCR targeting *integrase*. Statistical analysis employed a nonparametric ANOVA‐Kruskal‐Wallis.

**Results: **162 samples (88 HIV+, 74 HIV‐controls) were quantified for plasma biomarker levels and 64 participants (29 HIV+, 35 HIV‐controls) were analysed for cellular markers. Median viral load and CD4‐percentage at ART initiation was 738,500.5copies/ml and 36.9%. At 8 years, there were no differences between the CD4‐percentage of the HIV+ and control group (*p *=* *0.261). The HIV+ group showed lower levels of IL‐3(*p *<* *0.0005), RANTES(*p *=* *0.0078), GCSF(*p *<* *0.00001), INF‐γ(*p *=* *0.003), TGFβ3(*p *<* *0.00001) and IL‐17A(*p *<* *0.00001). HIV+ children showed decreased expression of CD38 and HLA‐DR, on both CD4 + (*p *=* *0.005) and CD8 + (*p *=* *0.004) T‐cells. Ki67 on CD4 + T‐cells were lower in the HIV+ group (*p *=* *0.0387). Exhaustion parameters on CD4 + T‐cells were elevated in the HIV+ group (TIGIT[*p *=* *0.017]; LAG‐3[*p *=* *0.0002]; PD‐1[0.0052]) including LAG‐3(*p *=* *0.0021) and PD‐1(*p *=* *0.0037) on CD8 + T‐cells. Among 32 children assessed for HIV‐1 DNA, a median of 32.5 copies/million cells was observed at 8 years.

**Conclusions: **Full restoration of immunity does not reach normality comparable to uninfected counterparts despite early, long‐term viral suppression, normalized CD4 counts and low cell‐associated HIV‐infectivity. Decreased immune activation, poor T‐cell proliferative capacity and immune exhaustion, may predispose to rapid viral rebound and lack of immune control on cessation of therapy. Any cure approaches need to attempt functional immune restoration prior to intervention, even when measures of viral infectivity are low.

### WEAA0102

#### Immune activation markers and CMV DNA‐aemia in children with perinatally acquired HIV infection

**L.‐M. Yindom^1^; V. Simms^2^; E.D. Majonga^2,3^; G. McHugh^2,3^; E. Dauya^3^; T. Bandason^3^; J. Rylance^4^; S. Munyati^3^; R.A. Ferrand^2,3^ and S.L. Rowland‐Jones^1^**

^1^University of Oxford, Nuffield Department of Medicine, Oxford, United Kingdom, ^2^London School of Hygiene and Tropical Medicine, London, United Kingdom, ^3^Biomedical Research and Training Institute, Harare, Zimbabwe, ^4^Liverpool School of Tropical Medicine, Liverpool, United Kingdom

**Background: **Soluble factors in blood plasma play a pivotal role in both the innate and adaptive immune responses to pathogens. Changes in their levels may impact on diagnosis and management of infectious diseases including HIV. In sub‐Sahara Africa, long‐term survival of older children with perinatally acquired HIV (PHIV) is associated with significant health problems that are not typical of HIV‐associated opportunistic infections or AIDS‐defining illnesses. They experience a range of chronic complications including growth impairment and chronic lung disease (CLD). Moreover, the beta herpes virus cytomegalovirus (CMV) which is ubiquitous in Africa, infects all children by age 18 months. We hypothesised that CMV reactivation is associated with changes in levels of biomarkers and comorbidities associated with persistent immune activation and/or inflammation in older children with PHIV.

**Methods: **Plasma samples were isolated from two cohorts of older children and adolescents aged 6 to 16 years with PHIV (n = 402) and HIV uninfected controls (n = 224). The HIV‐infected children were either newly diagnosed (antiretroviral therapy (ART) naïve) or known to be HIV+ but stable on ART. CMV DNA‐aemia was measured using quantitative polymerase chain reaction (qPCR). A multiplex bead array assay was used to measure the levels of 30 biomarkers of immune activation, inflammation, angiogenesis, fibrinolysis and apoptosis.

**Results: **At enrolment, CMV DNA‐aemia ≥ 1000 copies/ml (defined as “clinically significant”) was detected in 5.8% of uninfected children, 14.1% of HIV‐infected participants stable on ART and 22.5 % of the HIV‐infected ART‐naïve children (Chi^2^ = 23.4, *p *<* *0.001). High CMV load was independently associated with reduced lung function (adjusted odds ratio aOR = 3.15, 95%CI: 1.20 to 8.28, *p *=* *0.02) and stunting in the HIV+/ART‐naïve and ART‐treated groups, respectively. Data on CMV contribution to levels of immune activation and other biomarkers are being analysed and will be presented at the conference.

**Conclusions: **CMV DNA‐aemia is common in older children and adolescents with PHIV and may contribute to changes in the levels of biomarkers of immune activation/inflammation, even amongst those stable on ART, suggesting a role for inadequately controlled CMV infection in the pathogenesis of PHIV in Africa.

### WEAA0103

#### Deep sequence analysis of HIV adaptation following vertical transmission: Importance of human leucocyte antigen‐driven selection on the evolution of HIV

**S. Gaudieri^1,2,3^; J. Currenti^1^; M. John^3^; E. McKinnon^3^; S. Leary^3^; A. Chopra^3^; M. Pilkinton^2^; R. Smith^2^; L. Barnett^2^; W. McDonnell^2^; M. Lucas^4^; S. Mallal^2,3^; J. Conrad^5^ and S. Kalams^2^**

^1^University of Western Australia, School of Human Sciences, Nedlands, Australia, ^2^Vanderbilt University Medical Center ^VUMC^, Division of Infectous Diseases, Nashville, United States, ^3^Murdoch University, Institute for Immunology and Infectious Diseases, Murdoch, Australia, ^4^University of Western Australia, Nedlands, Australia, ^5^Vanderbilt University, Nashville, United States

**Background: **HIV can adapt to an individual's T cell immune response via mutations that affect antigen recognition and disease outcome. These viral adaptations are specific to the host's human leucocyte antigen (HLA) alleles, as these molecules determine which peptides are presented to T cells. Transmitted viral adaptations can be maintained or undergo reversion in a new host dependent on the cost‐benefit balance. We used the unique features of vertical HIV transmissions, primarily a known source of transmitted virus and sharing of HLA alleles that restrict T cell epitope specificity, to predict the *in vivo* replicative capacity and immune escape benefit of specific HIV adaptations that could be used to inform vaccine design.

**Methods: **A deep sequencing approach was utilised to determine the HIV clade B quasispecies in 26 confirmed mother‐to‐child transmission pairs where the potential for founder viruses to be pre‐adapted is high due to the pairs being haplo‐identical at HLA loci. This scenario allowed the assessment of the dynamics of known HIV adaptations following transmission in either a non‐selective environment (mediated by HLA mismatched to original selecting HLA), or selective immune environment (mediated by shared HLA alleles). Anti‐HIV‐specific IFN‐ϒ T cell responses were assessed using intracellular cytokine staining.

**Results: **Overall, the transmitted virus was highly adapted to the child's anti‐HIV T cell immune potential. The pattern of reversion and fixation of HIV adaptations following transmission was strongly influenced by the HLA‐driven selective environment of the recipient and provided an insight into the replicative capacity cost associated with specific adaptations. Furthermore, there was evidence of *de novo* post‐transmission adaptation, representing new targets of the child's T cell responses. These de novo adaptations were more likely to occur at sites relevant to paternally inherited HLA alleles compared to sites relevant to the mother's HLA alleles (*p *=* *0.008; mixed‐effects logistic regression); reflecting the transmission network.

**Conclusions: **HLA‐driven selection pressure is a major contributor to HIV evolution. An understanding of the balance between replicative capacity and immune escape benefit of these adaptations is an important consideration for vaccine and cure strategies for individuals exposed to adapted viruses via transmission or activated from reservoirs.

### WEAA0104

#### No evidence of HIV‐1 evolution in circulating reservoirs in children during more than six years of early suppressive antiretroviral therapy despite detectable levels of cell associated‐HIV‐RNA

**M. Moragas^1^; M.D. Golemba^1^; M. Distefano^1^; R. Bologna^2^ and A. Mangano^1^**

^1^Hospital de Pediatría ‘Prof. Dr. Juan P. Garrahan’, Laboratorio de Biología Celular y Retrovirus ‐ CONICET, Ciudad Autónoma de Buenos Aires, Argentina, ^2^Hospital de Pediatría ‘Prof. Dr. Juan P. Garrahan’, Servicio de Epidemiología e Infectología, Ciudad Autónoma de Buenos Aires, Argentina

**Background: **Whether HIV‐1 maintains its latent reservoirs by long‐term survival and proliferation of cells infected before the initiation of antiretroviral therapy (ART), or by ongoing viral replication, is still under debate. Our aim were to determine in HIV‐1‐infected children if viral evolution occur during early suppressive ART and if the viral diversity correlate with reservoir size.

**Methods: **Peripheral blood cells from 5 HIV‐1‐infected children were evaluated by MiSeq deep sequencing of *p17gag* region prior to ART initiation and during 6 to 15 years (3 to 5 samples/children) of viral suppression (VS). Haplotypes were constructed by merging sequences of frequencies below the error rate (< 0.5%) with the most common ones to reduce the effects of PCR and sequencing errors. Relationships among the sequences obtained before starting ART and throughout VS were evaluated using maximum likelihood method. Subsequently, molecular evolution was tested by root‐to‐tip analysis. Cell associated HIV‐1‐DNA and ‐RNA (CA‐HIV‐DNA and ‐RNA) were quantified by semi‐nested real time PCR.

**Results: **Children started ART and achieved VS at a median age of 4 and 10 months of life, respectively. Neither read depth nor levels of CA‐HIV‐DNA showed correlation with the number of haplotypes recorded: R^2^ =0.175 and R^2^ =0.105, respectively; suggesting no bias in the pipeline. Phylogenetic analysis showed no relationship between the viral sequences and sampling time, visualized as an intermingling of the haplotypes at different times. The linear regression obtained by the root to tip analysis showed an R^2^ from 0.003 to 0.154, indicating a poor correlation between genetic divergence and sampling time. Moreover, in the 5 children, 100, 94, 75, 66 and 34% of the haplotypes found at last visit (6 to 15 years under VS), representing virus from the circulating reservoir, were identical to those before starting ART. Surprisingly, CA‐HIV‐RNA was detectable at last visit in all patients with a median of 3.6 (IQR: 1.5 to 3.76) log10 copies/ug RNA.

**Conclusions: **These findings support that the reservoirs originate from cells infected closely to ART initiation and not by ongoing viral replication. However, the detection of active viral transcription needs further study. Our results contribute to the understanding of HIV‐1 reservoirs dynamics in children, which is important for eradication strategies.

### WEAA0105

#### Oral TLR7 agonist administration induces an immunostimulatory response in SIV‐infected ART‐suppressed infant Rhesus macaques

**K. Bricker^1^; V. Obregon‐Perko^1^; J. Hesselgesser^2^ and A. Chahroudi^1,3,4^**

^1^Emory University School of Medicine, Pediatric Infectious Diseases, Atlanta, United States, ^2^Gilead Sciences Inc., Foster City, United States, ^3^Emory University, Yerkes National Primate Research Center, Atlanta, United States, ^4^Emory+Children's Center for Childhood Infections and Vaccines, Atlanta, United States

**Background: **Globally, 2.1 million children are living with HIV‐1 and the majority of new infections occur postnatally through breast milk transmission. The major obstacle to HIV/AIDS cure is the presence of a reservoir of latently infected cells that persists even under ART treatment. Recent studies have demonstrated that a toll‐like receptor 7 agonist can reverse viral latency and alone or with use of a therapeutic CD8‐inducing vaccine may facilitate reduction of the viral reservoir. In this study, two dose levels of an orally delivered TLR7 agonist (GS‐986) were administered to SIV‐infected ART‐suppressed 7‐month old rhesus macaques (RMs) to evaluate tolerability and pharmacodynamic responses.

**Methods: **Two 5‐week‐old RMs were infected with SIVmac251 orally in two doses 24 h apart and placed on daily ART beginning at 4 wks post infection. Both animals were virologically suppressed for over 3 months before administration of GS‐986. At 7 months of age, RMs received 0.1 mg/kg GS‐986 via oral gavage (o.g.). Complete blood count (CBC), serum chemistry, plasma viral loads, plasma cytokine concentrations, and immune cell activation were monitored prior to administration, 24 h, and 1 wk post administration. Plasma was collected prior to and 30 min following administration for pharmacokinetic (PK) analysis. Following 4 wks of rest, animals received a second dose of 0.3 mg/kg (o.g.) and analyses were repeated.

**Results: **GS‐986 was well tolerated at both administered doses with no adverse clinical observations and normal CBC and chemistry at 24 h and 7 d post administration. Both RMs maintained undetectable viremia following administration. Concentrations of IFN‐α, IL‐1RA, IL‐6, IP‐10, and I‐TAC were elevated in the plasma at 24 h post‐administration and returned to pre‐dosing levels by 7 d post‐administration. Increases in monocytes (CD3^‐^CD4^int^ CD14^+^ CD16^+^) and circulating (CD169^+^) macrophages was observed 24 h following GS‐986 administration with a return to baseline by day 7.

**Conclusions: **In summary, we have demonstrated that oral administration of GS‐986 is tolerated in infant RMs, with induction of expected immune parameters. Future work will involve investigating the effect of GS‐986 with a therapeutic vaccination on viral reservoir and viral rebound following analytical treatment interruption.

### WEAA0201

#### Longitudinal dynamics of follicular CD4 + T cells in acute SIV infection

**L. Kuri‐Cervantes^1^; C. Deleage^2^; E. Roberts^1^; S. Nguyen^1^; V. Wu^1^; H. Gunzelman^1^; D. Carnathan^3^; T. Vanderford^4^; G. Silvestri^3^ and M. Betts^1^**

^1^University of Pennsylvania, Department of Microbiology, Philadelphia, United States, ^2^Frederick National Laboratory for Cancer Research, AIDS and Cancer Virus Program, Frederick, United States, ^3^Emory University, Emory Vaccine Center, Atlanta, United States, ^4^Emory University, Yerkes National Primate Research Center, Atlanta, United States

**Background: **Follicular T helper CD4 + (Tfh) cells play a critical role in germinal center (GC) formation and B cell maturation. GCs in lymph nodes (LN), particularly within Tfh cells, are sites for preferential SIV infection and replication. Changes in Tfh cells in early acute SIV infection may be a major determinant in the development of effective antibody‐mediated control of SIV infection.

**Methods: **Eighteen macaques were infected with SIVmac251 and underwent staggered necropsy during acute and chronic infection. Tfh cells from surface LN (sLN), mesenteric LN (mLN) and spleen were immunophenotyped. We further examined mLN to quantify and localize viral RNA (vRNA) using immunohistochemistry, and performed gene expression and pathway enrichment analyses on sorted Tfh cells from LNs in resting and stimulated conditions.

**Results: **The frequency of Tfh cells decreased after 10 days post‐infection (d.p.i.) and partially rebounded after 20 d.p.i in all tissues. Using principal component analyses we found similar phenotypic profiles in Tfh from mLN and sLN; in contrast, Tfh isolated from the spleen clustered separately after 10 d.p.i. Although plasma viremia (pVL) peaked at 10 d.p.i., vRNA in mLNs was detectable as early as 5 d.p.i. within follicles and the T cell zone. While pVL decreased after 20 days, tissue vRNA was increased until 90 d.p.i. but was not preferentially found within the follicles. Very early following infection, transcriptional profiling of Tfh‐related genes showed profound modulation of cytokine production and inflammatory pathways. We observed a decrease in Tfh responsiveness to stimulation as early as 5 d.p.i. Functions were partially recovered after 20 d.p.i. irrespective of the increasing vRNA in tissues. tSNE analyses showed independent clustering pre‐ and post‐infection, and Tfh cells from 90 d.p.i. had the closest profile to pre‐infection suggesting a partial recovery in responsiveness in later stages of infection.

**Conclusions: **SIV infection has a profound effect in Tfh frequencies, phenotypic and genetic profiles across tissues since acute infection. This effect suggests a temporal decrease in Tfh ability to provide B cell help during early stages of infection associated with high levels of viremia in blood and tissues, that may directly impact or delay the early induction of SIV‐specific antibody production.

### WEAA0202

#### HIV gp120‐mediated CD4^+^T cell activation is inhibited by non‐neutralizing gp120‐V2 loop antibodies

**C. Cicala^1^; L. Goes^1^; A. Sajani^1^; F. Nawaz^1^; D. Van Ryk^1^; J. Yolitz^1^; D. Wei^1^; R. Mason^1^; M. Roederer^1^; X.‐P. Kong^2^; J. Arthos^1^ and A.S. Fauci^1^**

^1^NIH, Bethesda, United States, ^2^NYU, New York, United States

**Background: **HIV‐1 preferentially replicates in inductive sites of GALT; however, the propensity to replicate in these tissues is not fully understood. Trafficking of both naïve and memory CD4^+^T cells to gut tissues is mediated, in part, by integrin α4β7binding to MAdCAM on high endothelial venules in the gut. The interaction between MAdCAM andα4β7delivers costimulatory signals to lymphocytes that, in the context of antigen specific signaling, activates lymphocytes.HIV and SIV gp120 also bind to integrin α4β7. This interaction is mediated primarily by the V2 loop of gp120, which mimic MAdCAM in the way that it binds to α4β7. We previously reported that MAdCAM costimulation through α4β7promotes CD4^+^T cell activation and proliferation thereby rendering cells more susceptible to viral replication. These observations prompted us to evaluate the capacity of gp120 V2‐mediated signaling to co‐stimulate cells in a manner to that of MAdCAM.

**Methods: **Freshly isolated CD4^+^T cells were stimulated with various combinations of anti‐CD3, HIV and SIV gp120, MAdCAM, anti‐CD28 and retinoic acid (RA). Activation and proliferation were evaluated by standard methods. Gene expression profiling was also carried out.

**Results: **We determined that gp120 proteins can promote the activation and proliferation of primary α4β7^high^/CD4^+^T cells. This activation is inhibited by an anti‐α4β7mAb. It is also inhibited by anti‐ V2 domain antibodies including non‐neutralizing mAbs that recognize an epitope in V2 that has been linked to reduced risk of acquisition in the RV144 vaccine trial.

**Conclusions: **The capacity of the V2 domain of gp120 to mediate signaling through α4β7on CD4^+^T cells likely impacts early events in infection immediately following transmission. In this regard, the ability of an anti‐α4β7mAb to block this activity raises the prospect that treatment with α4β7antagonists may inhibit pathogenic mechanisms associated with HIV disease, particularly in gut tissues. Finally, the capacity of non‐neutralizing V2 antibodies to block this activity provides a novel mechanism whereby such antibodies might impact both transmission and pathogenesis of HIV/SIV.

### WEAA0203

#### Tandem bispecific antibody prevents fully and induces prolonged T cell immunity against pathogenic SHIV in monkey models

**M. Niu; Y.C. Wong; L. Ling; X. Li; Y. Du; C.Y. Chan and Z. Chen**

AIDS Institute, Department of Microbiology, Li Ka Shing Faculty of Medicine, The University of Hong Kong, Hong Kong, China

**Background: **Passive immunization of highly potent and broadly neutralizing antibodies(bNAbs) have been shown to prevent and suppress viremia in animal models and human clinical trials. Our previous study has proved that the tandem bi‐specific bNAbs, BiIA‐SG, was highly effective in protection and treatment settings in humanized mice. Here, we further investigate the prophylactic and therapeutic potential of BiIA‐SG against simian‐human immunodeficiency virus (SHIV) challenge in Chinese‐origin rhesus macaques.

**Methods: **Rhesus macaques were infected intravenously with SHIVSF162P3 (5000TCID50). For prophylaxis, BiIA‐SG was administered intramuscularly 1 day before challenge, whereas for treatment, BiIA‐SG was given either 1 day or 3 days after challenge. Plasma viral loads were monitored by real‐time PCR. Antibody concentration and neutralizing activity was evaluated by ELISA and neutralization assay. ELISPOT and ICS assays were performed to assess T cell response.

**Results: **We show that the half‐life of BiIA‐SG is around 2.3 days in the macaques. A single intramuscular injection of BiIA‐SG one day before SHIVSF162P3infection conferred full protection in all rhesus macaques tested. In addition, with a single infusion of BiIA‐SG after 1 day or 3 days of SHIVSF162P3challenge, the peak viremia occurrence was significantly postponed, followed by undetectable setpointviral loads from 2 months post infection onwards. Importantly, the antibody infusion vastly reduced the chance of rapid progression to AIDS. Mechanistically, CD8^+^ T cells were involved in long term viral suppression. Rapid viral rebound was induced in animals treated with T‐cell‐depleting anti‐CD8β antibody, suggesting that early administration of BiIA‐SG may induce long‐lasting CD8^+^T cell immunity to durably suppress SHIVSF162P3 replication.

**Conclusions: **In summary, our study demonstrates the efficacy of BiIA‐SG in controlling SHIV infection. Our findings strongly support the investigation of BiIA‐SG immunotherapy for HIV‐1 in humans

### WEAA0204

#### Bone marrow stromal antigen‐2 (bst‐2) gene variants associate with HIV‐1 control in black South African individuals

**B.D.C. Dias^1^; M. Paximadis^1^; Z. Waja^2^; N. Martinson^2,3^; R.E. Chaisson^2^; O. Ebrahim^4^ and C.T. Tiemessen^1^**

^1^Centre for HIV and STIs, National Institute for Communicable Diseases, National Health Laboratory Service, and Faculty of Health Sciences, University of the Witwatersrand, Johannesburg, South Africa, ^2^Perinatal HIV Research Unit (PHRU), SA MRC Soweto Matlosana Collaborating Centre for HIV/AIDS and TB, University of the Witwatersrand, Johannesburg, South Africa, ^3^Center for TB Research, Johns Hopkins University, Baltimore, United States, ^4^School of Clinical Medicine, Faculty of Health Sciences, University of the Witwatersrand, Johannesburg, South Africa

**Background: **Variability in susceptibility to HIV‐1 acquisition and the clinical course of infection is influenced by host genetic factors, including viral restriction factors. Bone marrow stromal antigen‐2 (BST‐2 or tetherin), is a restriction factor which efficiently blocks the release of enveloped viruses. Few studies have assessed the role of *bst‐2* polymorphisms in HIV‐1 acquisition or disease progression, particularly in sub‐Saharan Africa.

**Methods: **We used size discrimination gel electrophoresis and allele‐specific SYBR^®^ green real‐time PCR assays to determine the frequency of four *bst‐2* variants, previously associated with the clinical course of HIV infection, namely: rs3217318 (19 bp deletion(Δ)→insertion (i)), rs12609479 (G→A), rs10415893(G→A) and rs113189798 (A→G) in a cohort of HIV‐1 uninfected black South Africans [n = 96].

**Results: **Interestingly, homozygosity for the rs12609479‐A (minor allele in Africans), previously predicted to decrease HIV‐1 acquisition risk by increasing expression, was notably underrepresented in our population (2%) compared to reference African populations (9%) and Europeans (61%) (*p* = 0.04 and *p* < 0.001, respectively).To assess the influence of these variants on HIV‐1 control, a purposely recruited group of HIV‐1 infected ART‐naïve participants were grouped into: progressors [n = 72] and controllers [n = 71], the latter including elite controllers [EC: n = 23; VL < 50 RNA copies/ml]. Our results revealed that rs12609479 (G/A) heterozygosity was enriched in progressors compared to ECs (47.2% vs 21.7%, OR = 3.50 [1.16 to 10.59], *p* = 0.03) whilst rs113189798 heterozygosity (A/G) showed a strong trend of overrepresentation in ECs compared to progressors (47.8% vs 26.4%, OR = 0.39 [0.14 to 1.04], *p* = 0.07). Owing to moderate linkage disequilibrium between loci, the influence of combined genotypes was investigated. Heterozygosity at both rs3217318 (i19/Δ19) and rs10415893(G/A) was associated with a faster rate of CD4 + T‐cell decline in progressors (*p* = 0.03). Possession of rs12609479 (G/G) and rs113189798 (A/G) combined genotype was associated with significantly lower VL in controllers (*p* = 0.0188) and higher CD4 + T‐cell counts within the progressor group (*p* = 0.0467), suggesting a protective genotype.

**Conclusions: **These data suggest possession of select combinations of *bst‐2* genotypes influence disease progression, implicating improved BST‐2 restriction of HIV‐1 in elite control and in preservation of CD4 + T‐cells in progressive infection.

### WEAA0301

#### Sting agonist as a kick and kill agent to target the HIV reservoir

**M. Mavigner^1^; A.D. Brooks^1^; A. Koblansky^2,3^; C. Galardi^2,3^; H. Madsen^2,3^; D.M. Margolis^3^; G. Silvestri^1^ and A. Chahroudi^1^**

^1^Emory University School of Medicine, Atlanta, United States, ^2^ViiV Healthcare, Research Triangle Park, United States, ^3^University of North Carolina, Chapel Hill, United States

**Background: **A favored strategy to eliminate the latent HIV reservoir is referred to as “kick and kill”, that aims to reactivate HIV gene expression followed by clearance of cells with reactivated virus. The signaling protein STING (STimulator of INterferon Gene) is a key mediator in innate immune sensing of viruses. STING‐mediated activation of IRF3, IRF7 and NF‐kB leads to production of type I IFN, proinflammatory cytokines TNFα and IL‐6, and chemokines such as IP‐10. STING agonism may also enhance antigen presentation and priming of antigen‐specific T‐cell responses. Activating the STING pathway represents a previously unexplored strategy for HIV cure with the potential to result in both latency reversal and boosting of HIV‐specific T‐cell responses.

**Methods: **We evaluated safety and efficacy of a small molecule STING agonist (STINGa) with a dose escalation study in rhesus macaques (RMs). Six RMs were infected i.v. with SIVmac239, treated with antiretroviral therapy (ART) for over a year, and then administered weekly STINGa at ascending doses. Pharmacokinetic and pharmacodynamic parameters were tracked, along with plasma viral loads.

**Results: **STINGa was well tolerated in ART‐suppressed SIV‐infected RMs, with complete blood counts and serum chemistries within normal limits. STINGa dose escalation resulted in increased drug exposure, as expected. Pharmacodynamic analyses showed a significant response at the highest dose. Specifically, STINGa administered at the highest dose resulted in a transient decrease in the frequency of (i) monocytes and (ii) T‐cells in 4/6 RMs, and (iii) B‐cells in 5/6 RMs. Furthermore, STINGa activity was confirmed by an increase in phosphorylated IRF3 in PBMCs and elevated plasma IL‐6 and IP‐10. Interestingly, STINGa induced transient but high‐level virus reactivation in 2/6 RMs with on‐ART viremia rising from < 60 to 2580 and 1450 copies/ml of plasma, respectively, 24 h after the last dose. These 2 RMs also demonstrated an increase in IFN‐g production in response to *gag* and *env* SIV peptides 5d after the last dose of STINGa.

**Conclusions: **This pilot study provides the first evidence for the potential to deploy a STING agonist as a “kick and kill” strategy for HIV latency reversal and enhancement of virus‐specific immune responses.

### WEAA0302

#### Different regimens of Romidepsin administration for reversion of SIV latency in a rhesus macaque model of complete virus control

**A. Kleinman^1^; S. Murali Kilapandal Venkatraman^1^; E. Penn^1^; B. Policicchio^1^; P. Sette^1^; E. Brocca‐Cofano^1^; M. Cottrell^2^; R. Sivanandham^1^; A. Valentine^1^; C. Xu^1^; K.D. Raehtz^1^; T.L. Dunsmore^1^; G. Haret‐Richter^1^; A. Kashuba^2^; J. Mellors^1^; I. Pandrea^1^ and C. Apetrei^1^**

^1^University of Pittsburgh, Pittsburgh, United States, ^2^University of North Carolina at Chapel Hill, Chapel Hill, United States

**Background: **HIV persistence in latent reservoirs requires lifelong antiretroviral treatment (ART) for control, necessitating a cure. The “shock and kill” approach utilizes latency reversing agents (LRAs) to reactivate and subsequently clear virus through viral cytolytic effects and cell‐mediated immune response. Histone deacetylase inhibitors are a well‐studied class of LRAs, the most potent being romidepsin (RMD), which has been shown to reactivate HIV/SIV reservoirs. Here, we investigated repeated and “double doses” of RMD in our spontaneous control model of SIV infection in rhesus macaques (RMs).

**Methods: **Five SIVsab‐infected RMs were monitored until viral loads (VLs) were suppressed (< 30 vRNA copies/mL) for minimum 2 months. RMs then received three rounds of RMD (7 mg/m^2^; 4‐hour slow‐perfusion) every two months. Three RMs received two additional rounds of “double dose” RMD administration (two doses, 48 hours apart) with two months between rounds. The remaining two animals were treated with CD8‐depleting antibody, M‐T807R1, after the first three RMD administrations.

**Results: **We found that in the infected macaques, the median RMD half‐life was 15 hours in blood, while in lymph nodes and gut RMD persisted for up‐to 9 days post‐treatment. RMD induced robust CD4^+^ T‐cell activation. In the absence of ART, SIV reactivated up to 10^4^ vRNA copies/mL. In three RMs, virus reactivation occurred after each round of RMD administration. Interestingly, in two RMs virus reactivation depreciated with each subsequent dose, concluding with no reactivation after the third. CD8 depletion resulted in loss of viral control in one animal that replicated the virus up‐to 10^6^ vRNA copies/mL. In the remaining RM, plasma vRNA remained undetectable. The switch to double dosage resulted in increased and more persistent immune activation and viral reactivation.

**Conclusions: **Our results show that the novel approach of giving double doses of RMD was readily tolerated by the animals and resulted in robust immune activation and viral reactivation. In a model of controlled SIV infection, RMD effectively and potently reactivated the latent reservoir. Importantly, the sequential decrease in viral reactivation with repeated RMD administration and complete lack of viral reactivation after CD8 depletion in one RM indicates that RMD may be decreasing the viral reservoir in this model.

### WEAA0303

#### Fingolimod treatment at ART initiation delays SIV rebound after ART interruption

**M. Pino^1^; C. King^1^; I. Shim^1^; H. Wang^1^; K. Nguyen^1^; S. Samer^1^; J. Harper^1^; J. Lifson^2^; A. Reynaldi^3^; C. Deleage^2^; K. Padhan^4^; B. Cervasi^1^; M.P. Davenport^3^; C. Petrovas^4^; M.M. Lederman^5^ and M. Paiardini^1,6^**

^1^Yerkes National Primate Research Center, Microbiology and Immunology, Emory University, Atlanta, United States, ^2^Leidos Biomedical Research, Frederick, United States, ^3^The Kirby Institute, UNSW Sydney, Sidney, Australia, ^4^Vaccine Research Center, NIAID, NIH, Bethesda, United States, ^5^Case Western Reserve University, Cleveland, United States, ^6^Emory School of Medicine, Atlanta, United States

**Background: **Lymph‐nodes (LN) are a critical site of HIV replication and persistence. Therefore, optimizing antiviral activity in lymphoid tissues is likely needed to reduce the HIV reservoir. We previously found that fingolimod (FTY720), a sphingosine‐1‐phosphate receptor modulator clinically approved for treatment of multiple sclerosis, promotes retention of cytolytic T‐cells in lymphoid sites of viral persistence in ART‐suppressed SIV‐infected rhesus macaques (RMs). In this new study, we treated SIV‐infected RMs with FTY720 at ART initiation, when HIV‐reactive cytolytic T‐cells are found in larger number than seen in ART‐treated aviremic animals. With this design, we aimed at exploring FTY720 potential to enhance SIV‐specific defenses in lymphoid sites during early infection thereby diminishing the size of SIV reservoirs.

**Methods: **14 RMs infected with SIVmac239 started ART at d42 post‐infection. Six RMs received only ART, while eight received FTY720 (daily, 500 mg/Kg) during the first 60 days of ART. ART was interrupted (ATI) after 11 months, and animals longitudinally followed for immunologic and virologic analyses.

**Results: **FTY720 at ART initiation was remarkably effective in reducing circulating CD4 + and CD8 + T‐cells (*p* < 0.0001), including those with cytolytic potential (*p* < 0.01), and induced a transient increase in frequencies of cycling (Ki‐67 + ) T‐cells in blood(*p* < 0.05). In FTY720‐treated animals the early kinetics of viral decay after ART initiation were similar (for productively infected short‐lived cells) or slower (productively infected long‐lived cells; *p* = 0.02) than those of controls, despite a dramatically reduced number of circulating CD4 + T‐cells. These data suggest that plasma SIV‐RNA levels persisting after ART initiation are generated in tissues and not dependent on circulating infected CD4 + T‐cells. Upon ATI, and nine months after the last dose of FTY720, 4 out of 8 treated RMs exhibited a delayed viral rebound when compared to rebound among controls(*p* = 0.048), suggesting that FTY720 treatment at ART initiation may have reduced the size of viral reservoir. Viral quantification in tissues is pending to confirm.

**Conclusions: **FTY720 administration at ART initiation retains T‐cells in lymphoid sites of SIV persistence and delays SIV rebound after ATI. These findings provide rationale for strategies designed to retain antiviral T‐cells in lymphoid tissues during early HIV infection to target HIV remission.

### WEAA0304

#### Vesatolimod (GS‐9620) is safe and pharmacodynamically active in HIV‐infected individuals

**S. Riddler^1^; M. Para^2^; C. Benson^3^; A. Mills^4^; M. Ramgopal^5^; E. DeJesus^6^; C. Brinson^7^; J. Cyktor^8^; J. Mellors^8^; S. Guo^9^; B. Doehle^10^; S. Markova^9^; H. Patel^9^; H. Graham^9^; J. Hesselgesser^9^; R. Geleziunas^9^; D. Brainard^9^; S. McCallister^9^ and D. Sengupta^9^**

^1^University of Pittsburgh School of Medicine, Infectious Diseases Division, Pittsburgh, United States, ^2^Ohio State University, Wexner Medical Center, Columbus, United States, ^3^University of California San Diego, UCSD Antiviral Research Center, San Diego, United States, ^4^SoCal Men's Medical Group, Los Angeles, United States, ^5^Midway Immunology & Research Center, LLC, Fort Pierce, United States, ^6^Orlando Immunology Center, Orlando, United States, ^7^Central Texas Clinical Research, Austin, United States, ^8^University of Pittsburgh, Department of Medicine, Pittsburgh, United States, ^9^Gilead Sciences, Foster City, United States, ^10^Gilead Sciences, Seattle, United States

**Background: **HIV infection requires lifelong treatment due to persistent viral reservoirs. Vesatolimod (VES; GS‐9620) is an investigational oral toll‐like receptor (TLR) 7 agonist that has led to viral remission in preclinical S(H)IV models when combined with an anti‐HIV antibody or CD8 vaccine. VES targets dendritic cells in gut associated lymphoid tissue (GALT) and the liver, resulting in a pre‐systemic response with localized cytokine production and innate immune modulatory effects. We evaluated the activity of VES in people living with HIV (PLH).

**Methods: **Virologically‐suppressed PLH were enrolled in a double‐blind, placebo‐controlled dose escalation study. Participants (n = 48) were randomized (6:2) to receive VES or placebo every other week in sequential dose escalation cohorts. Multiple (6 to 10) doses of 1 to 12 mg were tested. Measurements collected included: plasma HIV‐1 RNA, pharmacokinetic (PK), and pharmacodynamic (PD) parameters [interferon stimulated gene (ISG) mRNA expression, serum cytokines and cellular activation].

**Results: **The median (IQR) age of the participants (43 men, 5 women) was 47 (39, 54) years. The majority of individuals initiated antiretroviral therapy (ART) during chronic HIV‐1 infection, with a median of 8.1 years on ART. VES was well‐tolerated at all doses, with no study drug‐related Grade (G) 3 or 4 adverse events (AEs), no related serious AEs, and no AEs leading to study drug discontinuation. Study drug‐related AEs including mild, flu‐like symptoms consistent with VES PD, resolving within one day and not occurring with each dose, were observed in 10/40 participants at ≥ 2 mg. VES plasma exposure increased with dose escalation. Individuals receiving VES ≥ 4 mg had dose‐dependent induction in whole blood ISG mRNA at 24 to 48 hours post‐dose, and changes in the serum cytokines/chemokines ITAC, IP‐10 and IL‐1RA. Consistent T cell and NK cell activation occurred at doses ≥ 8 mg. Transient increases in plasma HIV‐1 RNA > 20 copies/mL (range 21 to 2430 copies/mL) were observed at least once in 13/48 participants (blinded) after 1 to 3 oral doses at different dose levels.

**Conclusions: **VES is well‐tolerated at doses of 1 to 12 mg and induced immune activation at higher doses. Clinical trials are in progress to evaluate the efficacy of VES, alone or in combination with other agents, to control viremia without ART.

### WEAB0101

#### Targeted screening and immediate start of treatment for acute HIV infection decreases time between HIV diagnosis and viral suppression among MSM at a Sexual Health Clinic in Amsterdam

**M. Dijkstra^1,2^; M.S. van Rooijen^1^; M.M. Hillebregt^3^; A.I. van Sighem^3^; C. Smit^3^; A. Hogewoning^1^; T. Heijman^1^; E. Hoornenborg^1^; M. Prins^12^; J.M. Prins^2^; M.F. Schim van der Loeff^1,2^; G.J. de Bree^2,4^ and on behalf of the H‐TEAM Initiative**

^1^Public Health Service Amsterdam, Department of Infectious Diseases, Research and Prevention, Amsterdam, Netherlands, ^2^Academic Medical Center of the University of Amsterdam, Department of Infectious Diseases, Amsterdam, Netherlands, ^3^HIV Monitoring Foundation, Amsterdam, Netherlands, ^4^Amsterdam Institute for Global Health and Development, Amsterdam, Netherlands

**Background: **Immediate start of antiretroviral therapy (cART) during acute HIV infection (AHI) is beneficial for patients and reduces onward transmission. An AHI trajectory among men who have sex with men (MSM) was implemented in Amsterdam in 2015; MSM diagnosed with AHI were referred to start cART within 24 hours. We evaluated the AHI trajectory by comparing MSM diagnosed through the AHI trajectory and through routine strategies regarding the proportion of AHI (Fiebig I‐II) among HIV diagnoses and the time between diagnosis and viral suppression.

**Methods: **Data from 1013 MSM newly diagnosed at the Sexual Health Clinic (2008 to 2017) were linked with data from HIV treatment centres by a Trusted Third Party. We compared time between HIV diagnosis and viral suppression using the log‐rank test for four cART‐initiation policies: (1) start cART at CD4 < 500 cells/mm^3^ (2008 to 2011); (2) start cART at CD4 < 500 cells/mm^3^ and in patients with AHI (2012 to 2015); (3) universal start of cART (2015 to 2017); and (4) immediate start of cART, AHI trajectory (2015 to 2017).

**Results: **In 2015 to 2017, the proportion of AHI among HIV diagnoses was 52.6% (10/19) in the AHI trajectory and 4.2% (5/118) using routine diagnostic procedures (Figure 1). The median time between diagnosis and viral suppression for cART‐initiation policy 1, 2, 3, and 4 was 569 (IQR 259 to 1031), 228 (IQR 129 to 435), 95 (IQR 63 to 136), 55 (IQR 31 to 72) days respectively, *p* < 0.001 (Figure 2).

**Conclusions: **Implementation of the AHI trajectory, along with changes in treatment guidelines, resulted in a higher proportion of AHI diagnoses and a decreased time between HIV diagnosis and viral suppression.

Abstract WEAB0101‐Figure 1. Fiebig stage for 1,013 newly diagnosed MSM stratified by diagnostic strategy at a sexual Health clinic in Amsterdam, 2008 to 2017.

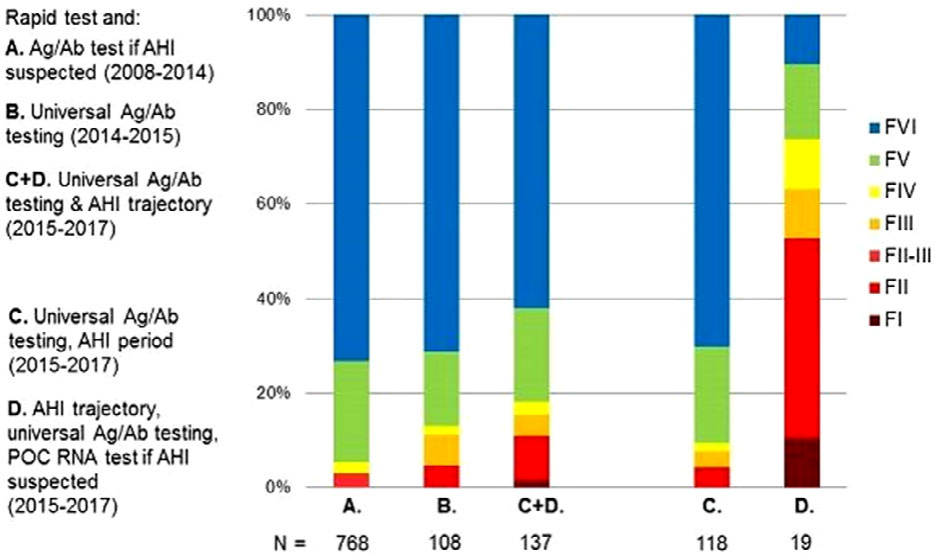

Abstract WEAB0101‐Figure 2. Median time from HIV diagnosis to viral suppression stratified by cART‐initiation policy among newly diagnosed MSM at a sexual health clinic in Amsterdam, 2008 to 2017.

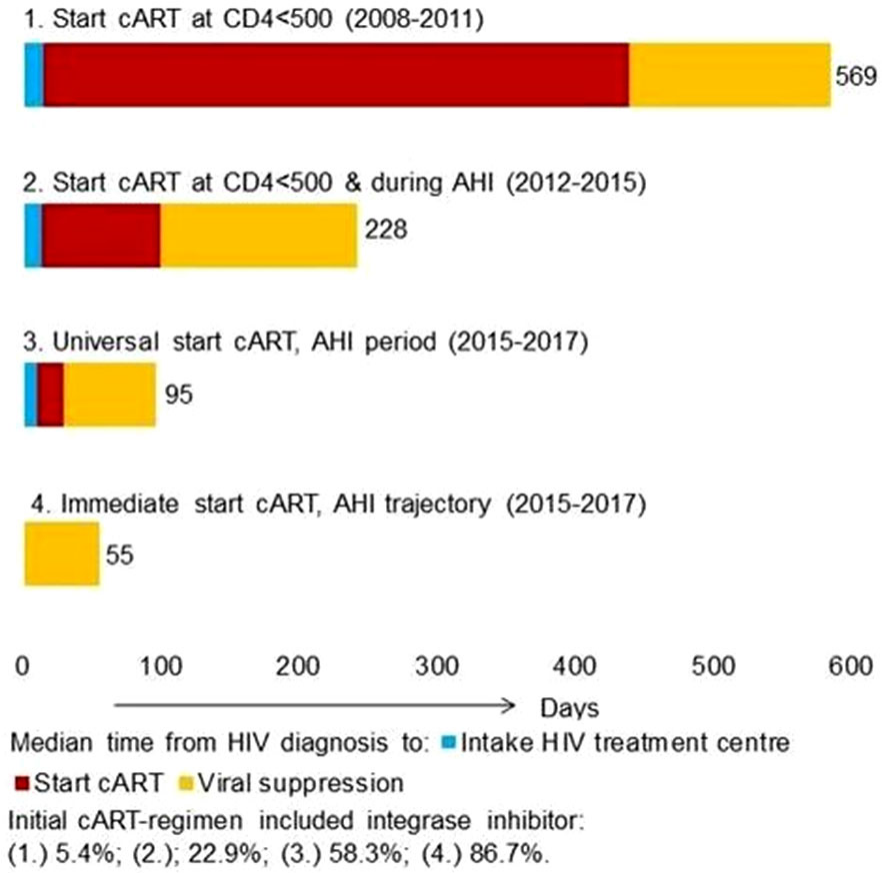

### WEAB0102

#### Same‐day antiretroviral therapy initiation in Thailand: Different models and initial outcomes from scale‐up in five provinces in Thailand

**P. Seekaew^1^; S. Amatavete^1^; N. Teeratakulpisarn^1^; P. Leenasirimakul^2^; S. Khusuwan^3^; P. Mathajittiphan^4^; A. Nilmanat^5^; P. Saenyakul^6^; P. Limbumrung^6^; A. Arunmanakul^6^; D. Donchaum^6^; K. Singhaseni^1^; R. Meksena^1^; D. Lingjongrat^7^; S. Janyam^8^; P. Chanlearn^9^; S. Sittikarn^10^; S. Charoenying^6^; S. Mills^6^; R. Vannakit^11^; P. Phanuphak^1^ and N. Phanuphak^1^**

^1^PREVENTION, Thai Red Cross AIDS Research Centre, Bangkok, Thailand, ^2^Nakornping Hospital, Chiang Mai, Thailand, ^3^Chiang Rai Prachanukroh Hospital, Chiang Rai, Thailand, ^4^Queen Savang Vadhana Memorial Hospital, Chonburi, Thailand, ^5^Hat Yai Hospital, Songkhla, Thailand, ^6^FHI 360 and LINKAGES, Bangkok, Thailand, ^7^Rainbow Sky Association of Thailand, Bangkok, Thailand, ^8^The Service Workers In Group Foundation, Bangkok, Thailand, ^9^Mplus Foundation, Chiang Mai, Thailand, ^10^Caremat, Chiang Mai, Thailand, ^11^USAID Regional Development Mission Asia, Office of Public Health, Bangkok, Thailand

**Background: **The World Health Organization (WHO) has recommended same‐day antiretroviral therapy (SDART) for clients who are ready. Since 2017, Thailand has scaled up SDART in five provinces, with different models based on laboratory requirements to facilitate ART initiation. This study aims to describe and evaluate initial outcomes from the various SDART models.

**Methods: **Data was obtained from HIV‐positive clients from nine facilities in five provinces (Chiang Rai, Chiang Mai, Chonburi, Bangkok and Songkhla) between July 2017‐December 2018. Baseline laboratory tests and chest X‐rays were performed according to national guidelines. ART eligibility was determined by a physician. Facilities were categorized, by the need to have laboratory results prior to ART initiation, into Group 1: no laboratory results needed, Group 2: CD4 count needed, and Group 3: safety laboratory results needed, without CD4 count. Logistic regression was performed to identify factors associated with successful SDART initiation and ART‐related adverse events.

**Results: **Results presented are in order of Groups 1, 2, and 3. Of 2876, 236, and 227 clients tested HIV‐positive, SDART acceptability was 89.4%, 99.6%, and 93%. ART was initiated in 86.5%, 84.7%, and 81.5%. Median (IQR) CD4 count was 298 (200 to 423), 290 (77 to 427), and 233 (80 to 371) cells/mm^3^ (*p* < 0.001). SDART initiation was 78.4%, 45.7%, and 57% (*p* < 0.001). There were 4 and 1 deaths in Groups 1 and 3, respectively. None was related to immune reconstitution inflammatory syndrome. Being in Group 1 (aOR 6.15, 95%CI 3.86 to 9.79, *p* < 0.001) and Group 3 (aOR 2.15, 95%CI 1.2 to 3.84, *p* = 0.010) increased the chance for SDART initiation. When looking at individual hospital and excluding a stand‐alone, VCT clinic, being newly diagnosed (aOR 0.51, 95%CI 0.29 to 0.88, *p* = 0.015), abnormal ALT (aOR 0.47, 95%CI 0.23 to 0.98, *p* = 0.044), and abnormal chest x‐rays (aOR 0.16, 95%CI 0.05 to 0.57, *p* = 0.004) decreased the likelihood of starting ART on same‐day. No factor was determined to be correlated to ART‐related adverse events.

**Conclusions: **Various SDART models have been explored in Thailand and all models were proved to be feasible and safe in different hospital settings. Requirement to have CD4 count prior to ART initiation, as still commonly practiced, could unnecessarily delay ART initiation without clinical benefits.

### WEAB0103

#### Treatment outcome among patients on ART in Southern Tanzania: Does time of ART initiation matter?

**B. Msongole^1^; J. Gamaliel^1^; B. Christian^2^; M. Mshana^3^; J. Bisimba^4^; E. Okechukwu^1^ and M. Njelekela^3^**

^1^FHI 360, Dar es Salaam, Tanzania, United Republic of, ^2^Management and Development for Health ^MDH^, Dar es Salaam, Tanzania, United Republic of, ^3^Deloitte Consulting Limited, Dar es Salaam, Tanzania, United Republic of, ^4^U.S. Agency for International Development (USAID), Dar es Salaam, Tanzania, United Republic of Tanzania

**Background: **Tanzania adopted test and treat recommendation by WHO aiming at achieving early ART initiation which is associated with better treatment outcome and reduces HIV transmission risk. ART Initiation on day of diagnosis is among approaches used to ensure improved linkage to treatment . However, there has been concerns that same day ART initiation will negatively affect patients’ readiness and eventually their adherence to treatment. Effect of time of ART initiation on retention rate and HIV Viral suppression rate was assessed.

**Methods: **Analysis of patient data from 5 hospitals in Iringa, Morogoro and Njombe regions was conducted. Treatment outcome of patients ≥ 15 years enrolled between April and September 2018 and initiated on ART on the same day of diagnosis were compared against those initiated under standard of care (1 to 14 days). Outcome of retention at 6 and 12 months and viral suppression at 12 months were measured. Data was extracted from CTC2 database and analysed using STATA.

**Results: **Among 1105 patients initiated on ART, 431 were initiated on same day of diagnosis while 674 were initiated in 1 to 14 days. Proportion of females was 72% and 63% in same day and 1 to 14 days group respectively. Retention at 6 months was high in same day group, 91% (95% CI 88% ‐94%) compared to 84% (95% CI 81% ‐ 87%, *p* = 0.0002) in 1 to 14 days group. Retention at 12 months was also high in same day group 88% (95% CI 84% ‐ 92%) compared to 84% (95% CI 79% ‐ 85%, *p* = 0.019) in 1 to 14 days group. There was no significant difference in viral suppression at 12 months between the two groups, 94% (95% CI 89% ‐ 99%) for same day and 95% (95% CI 92% ‐ 99%, *p* = 0.709) for 1 to 14 days group.

**Conclusions: **Patients initiated on treatment on the same day of diagnosis had better retention at 6 and 12 months compared to those initiated under standard of care. Viral suppression was high in both groups, there was no significant difference. Scaling up same day ART initiation as part of test and treat strategy is important in eliminating treatment gap caused by delays in treatment initiation.

### WEAB0104

#### High acceptability and feasibility of same‐day antiretroviral therapy services among HIV‐positive adolescents in Bangkok, Thailand

**P. Seekaew^1^; W.N. Songtaweesin^2,3^; T. Puthanakit^2,3^; S. Amatavete^1^; N. Teeratakulpisarn^1^; P. Surapuchong^1^; S. Teeratakulpisarn^1^; T. Chinbunchorn^1^; P. Jomja^1^; C. Hanaree^1^; P. Plodgratoke^4^; C. Saisaengjan^5^; K. Singhaseni^1^; R. Meksena^1^; S. Charoenying^6^; S. Mills^6^; R. Vannakit^7^; P. Phanuphak^1^ and N. Phanuphak^1^**

^1^Thai Red Cross AIDS Research Centre, PREVENTION, Bangkok, Thailand, ^2^Chulalongkorn University, Faculty of Medicine, Bangkok, Thailand, ^3^Chulalongkorn University, Center of Excellence in Pediatric Infectious Diseases and Vaccines, Bangkok, Thailand, ^4^Thai Red Cross AIDS Research Centre, Bangkok, Thailand, ^5^Thai Red Cross AIDS Research Centre, South East Asia Research Collaboration in HIV, Bangkok, Thailand, ^6^FHI 360 and LINKAGES, Bangkok, Thailand, ^7^USAID Regional Development Mission Asia, Office of Public Health, Bangkok, Thailand

**Background: **There has been an increase in global HIV incidence among adolescents; however, little is known about their care status after HIV diagnosis. Same‐day antiretroviral therapy (SDART) services have shortened preparatory steps while maintaining close relationships with clients after ART initiation. This study aims to assess the acceptability and feasibility of SDART among adolescents.

**Methods: **Data were collected among HIV‐positive clients aged 12 to 19 years at the Thai Red Cross Anonymous Clinic, the largest VCT clinic in Bangkok. Acceptability was self‐reported. Baseline laboratory tests and chest X‐rays were performed according to national guidelines. Physicians evaluated ART eligibility and initiated Tenofovir/Emtricitabine/Efavirenz, Nevirapine or Rilpivirine regimens on the day of diagnosis or as soon as clinically possible. Clients were screened for mental health problems as needed. ART was provided for 2 months and clients linked to long‐term ART according to their health insurance scheme. Time from care engagement to ART initiation and retention in care were calculated.

**Results: **From July 2017‐December 2018, 100 adolescents tested HIV‐positive: 82% were men who have sex with men (MSM), 16% general population and 2% transgender women (TGW). 95% accepted SDART: 96.3% among MSM, 87.5% general population, and 100% of TGW. Median (IQR) CD4 was 311 (253 to 381) cells/mm^3^; 27.5% tested reactive for syphilis (rapid plasma reagin test). 79% had same‐day ART initiation, another 17.8% within one week. Of 20 clients who had mental health screening, 4 had adjustment disorder, and 1 had major depressive disorder. Linkage to long‐term ART sites was successful in 81.3%. Retention among those linked successfully at 3, 6, and 12 months was 91.4%, 86.8%, and 80% respectively. 78% (25/32) of clients were virally suppressed after 6‐months of ART. Adolescents who were lost to follow‐up had lower income (100% vs. 42.9% with monthly income ≤ 10,000 THB, *p* = 0.042) than those retained.

**Conclusions: **The majority of newly diagnosed HIV‐positive adolescents were MSM. Acceptability of SDART was high and almost 80% started ART on the day of diagnosis. However, to ensure engagement in long‐term ART care, integrated mental health services and social support is needed for programmes serving adolescents.

### WEAB0105

#### “Test and treat” approaches to HIV care may affect the Xpert MTB/RIF testing impact in high burden TB/HIV settings: Results from a cohort frm a rural hospital in Southern Mozambique

**E. Nacarapa^1^; M.E. Verdu‐Jorda^1^; T. D. Moon^2^; G. Churchyard^3^ and E. Valverde^4^**

^1^Carmelo Hospital of Chokwe, Chokwe, Mozambique, ^2^Vanderbilt Institute of Global Health (VIGH), Division of Pediatric Infectious Diseases, Nashville, United States, ^3^The Aurum Institute, Johannesburg, South Africa, ^4^The Aurum Institute, Maputo, Mozambique

**Background: **Global roll out of Xpert MTB/RIF technology has resulted in dramatic changes in TB diagnosis, increasing bacteriologically confirmed TB cases two‐fold and detection of multi‐drug resistant TB cases eight‐fold. When endorsing Xpert MTB/RIF, WHO forecasted a two‐fold increase in the number of HIV‐associated TB cases reported. However, health system factors may limit the benefit in high‐burden TB/HIV settings, particularly when using “test and treat” approaches for HIV care.

**Methods: **The Carmelo Hospital of Chókwè (CHC) is a TB/HIV reference center in Gaza Province in Southern Mozambique. Xpert MTB/RIF testing was introduced in 2013. Implementation of a “test and treat” approach for ART started in 2016, following Ministry of Health guidelines. We conducted a retrospective cohort study of TB infected patients ≥ 15 years of age, diagnosed and treated at CHC between January 1, 2006 and December 31, 2017.

Patient characteristics, results of sputum acid fast bacilli smear and Xpert MTB/RIF, TB and HIV treatment starting dates, and treatment outcomes were recorded and compared before and after Xpert MTB/RIF and “test and treat” introduction. Tendencies in treatment outcomes were analysed with chi‐square.

**Results: **9739 patients ≥ 15 years of age were included in the analysis, 4357 (44.7%) were female. HIV testing was conducted in 9729 patients (99.9%), with 8132 (83.5%) having TB/HIV co‐infection. The number of TB/HIV co‐infected patients varied between 600 to 800 cases per year, with no observed increase after introduction of Xpert MTB/RIF testing. Percentages of bacteriologically confirmed cases dropped from 42.4% in 2008 to 22.0% in 2017. Death rates decreased to 12.1% in 2013, but later increased again up to 25.7% in 2017. The number of TB cases starting TB treatment prior to ART start declined from 58.0% in 2015 to 31.7% in 2017 after the introduction of “test and treat” approach (*p* < 0.0001).

**Conclusions: **Despite the impact of Xpert MTB/RIF introduction in TB diagnosis, challenges remain as to how to integrate Xpert MTB/RIF into diagnostic algorithms to maximize the effect of this new technology. HIV care “test and treat” approaches should be reviewed to highlight the need of reasonably excluding TB disease before ART start.

### WEAB0201

#### Describing the characteristics and long‐term outcomes of adolescents living with perinatally acquired HIV in the IeDEA‐Southern Africa Collaboration: 2004 to 2017

**P.R. Tsondai^1^; K. Braithwaite^2^; G. Fatti^3,4^; C. Bolton Moore^5^; C. Chimbetete^6^; H. Rabie^7^; S. Phiri^8^; S. Sawry^9^; B. Eley^10^; J.H. van Dijk^11^; J. Euvrard^12^; F. Tanser^13^; K. Taghavi^14^; A.H. Sohn^15^ and M.‐A. Davies^16^**

^1^University of Cape Town, Centre for Infectious Disease Epidemiology & Research, School of Public Health and Family Medicine, Cape Town, South Africa, ^2^Empilweni Services and Research Unit, Department of Paediatrics & Child Health, Rahima Moosa Mother and Child Hospital, Faculty of Health Sciences, University of the Witwatersrand, Johannesburg, South Africa, ^3^Kheth'Impilo, Cape Town, South Africa, ^4^Division of Epidemiology and Biostatistics, Department of Global Health, Faculty of Medicine and Health Sciences, Stellenbosch University, Cape Town, South Africa, ^5^Centre for Infectious Disease Research in Zambia (CIDRZ), Lusaka, Zambia, ^6^Newlands Clinic, Harare, Zimbabwe, ^7^Tygerberg Hospital, Stellenbosch University, Department of Pediatrics and Child Health, Cape Town, South Africa, ^8^Lighthouse Trust Clinic, Lilongwe, Malawi, ^9^Harriet Shezi Children's Clinic, Wits Reproductive Health and HIV Research Unit, University of Witwatersrand, Johannesburg, South Africa, ^10^Red Cross War Memorial Children's Hospital, and the Department of Paediatrics and Child Health, University of Cape Town, Cape Town, South Africa, ^11^SolidarMed, Masvingo, Zimbabwe, ^12^Centre for Infectious Disease Epidemiology & Research, School of Public Health and Family Medicine, University of Cape Town,, Cape Town, South Africa, ^13^Africa Centre for Health and Population Studies. University of KwaZulu‐Natal, Mtubatuba, South Africa, ^14^Institute of Social and Preventive Medicine (ISPM), University of Bern, Bern, Switzerland, ^15^TREAT Asia/amfAR ‐ The Foundation for AIDS Research, Bangkok, Thailand, ^16^Centre for Infectious Disease Epidemiology & Research, School of Public Health and Family Medicine, University of Cape Town, Cape Town, South Africa

**Background: **Adolescents living with perinatally acquired HIV (ALPH) have several unique characteristics resulting from their long‐term exposure to HIV and chronic exposure to antiretroviral drugs. We describe the characteristics and long‐term outcomes of ALPH within IeDEA‐Southern Africa (IeDEA‐SA).

**Methods: **We analysed routine data from 16 IeDEA‐SA sites (2004─2017) of ALPH entering HIV care aged < 13 years, who had ≥ 1 HIV care visits after age 10 years. Patient characteristics are described at enrolment, initiation of antiretroviral therapy (ART) and at different ages during adolescence. Using competing risks analysis, we estimated the outcomes: mortality, loss to follow‐up (LTFU: no visit in the 12 months before database closure) and transfers. We used Cox Proportional Hazards regression to determine predictors of mortality in the 6 years following their 13th birthday.

**Results: **Of 25,401 ALPH included, 51% were female. At enrolment, median (interquartile range [IQR]) age was 8.8 (5.9─10.9) years, with 51.8% (95% confidence interval [CI] 51.1─52.6) severely immunosuppressed (WHO 2007 criteria), 42.7% (41.6─43.7) underweight (weight‐for‐age z‐score < ‐2) and 50.6% (49.3─51.8) stunted (height‐for‐age z‐score < ‐2). Median (IQR) age at ART start and duration of follow‐up from ART initiation were 8.9 (6.1─10.9) years and 6.0 (3.0─8.6) years, respectively (Table 1).

Abstract WEAB0201‐Table 1. Characteristics of ALPH by age

**Table:** 

Characteristic, median (IQR)	At age 13 years (n = 14,359)	At age 15 years(n = 8407)	At age 18 years (n = 3170)
Duration on ART (years)	3.6 (1.7 to 6.0)	5.0 (3.3 to 7.0)	7.1 (5.8 to 8.8)
CD4 cell count (cells/µL)	601 (378 to 848)	567 (390 to 785)	488 (296 to 680)
Height‐for‐age z‐score	−2.07 (−2.90 to −1.21)	−1.91 (−2.77 to −1.11)	−1.48 (−2.29 to −0.77)
HIV‐RNA < 400 copies/mL (n/N)*; % (95% CI)	(3415/4693); 72.8 (71.5 to 74.0)	(2044/3045); 67.1 (65.4 to 68.8)	(742/1101); 67.4 (64.5 to 70.2)

*Non‐missing observations of patients at sites offering routine viral load monitoring.

During follow‐up, 2.6% died, 15.6% transferred and 21.4% were LTFU. Cumulative incidence (95%CI) for mortality, transfers and LTFU from age 10 years were 4.1% (3.7 to 4.4), 26.5% (25.7 to 27.3) and 32.1% (31.3 to 32.9) at age 18 years, respectively. Characteristics at age 13 years associated with subsequent confirmed mortality were: duration on ART (adjusted hazards ratio [aHR] per year increase 0.80 [95%CI 0.66 to 0.98]), immunosuppression (CD4 < 350: aHR 7.02 [4.41 to 11.18], CD4 350 to 500: aHR 2.59 [1.44 to 4.67] vs. CD4 > 500) and calendar year of their 13^th^ birthday (aHR per year increase 0.88 [0.81 to 0.95]).

**Conclusions: **Children with perinatally acquired HIV have suboptimal retention, viral suppression and survival during adolescence. Those initiating ART at older ages and those immunosuppressed during adolescence need careful follow‐up to optimize outcomes.

### WEAB0202

#### Predictors of treatment failure, time to switch and reasons for switching to second line antiretroviral therapy in HIV‐infected children receiving first line anti‐retroviral therapy

**A. Beyene**

Addis Ababa University, Pharmacology and Clinical Pharmacy, Addis Abeba, Ethiopia

**Background: **Treatment failure and delay in switching to second line regimen are major concerns in the treatment of HIV infected children in a resource limited setting. The aim of the THIS study was to assess the prevalence and predictors of 1^st^ line ART regimen failure, reasons for switching and time taken switch to 2^nd^ line ARV drugs after treatment failure among HIV‐infected children in Tertiary Care Hospital, Ethiopia.

**Methods: **A retrospective cohort study was conducted February 2003 to May 2018. All HIV infected children ≤ 15 years of age and who were taking first line ART for at least 6 months were included. Data was collected from patients’ chart . Binary and multivariable logistic regression statistics were used.

**Results: **Out of 318 enrolled HIV‐infected children, the prevalence of overall HIV treatment failure was found to be 22.6 % (72/318), among these 37 (51.4%) had only immunologic failure, 6 (8.3%) had only virologic failure and 24(33.3 %) had both clinical and immunological failure. The mean time taken to modify cART regimen was 12.67(4.96) weeks after treatment failure was confirmed. WHO Stage 3 and 4 [Adjusted Odds Ratio (AOR), 3.64, 95% CI 1.76 ‐ 7.56], not having both parents as primary caretakers [AOR, 2.72 95% CI, 1.05 ‐ 7.06], negative serology of care takers [AOR, 2.69 95% CI, 1.03 ‐ 7.03], and cART initiation at 11 month or younger were predicting factors of treatment failure. Of the 141(47.9%) children who had regimen switching or substitution, treatment failure (44.4%) and replacement of stavudine (d4T) (30.8%) were major reasons. A total of 6.6% (26/391) children died and only 6.6% (21/318) patients had received PMTCT service.

**Conclusions: **One fifth of the patients had experienced treatment failure. Advanced WHO stage at baseline, not being taken care of by mother and father, negative sero‐status caretakers, and younger age at initiation of cART were the predictors of treatment failure. PMTCT service uptake was very low. There was a significant time gap between detection of treatment failure and initiation of second line cART. Half of the patients encountered regimen switching or substitution of cART due to treatment failure and replacement of stavudine (d4T).

### WEAB0203

#### Dolutegravir containing regimens may need optimization for African youth failing ART

**V.M. Kouamou^1^; J. Manasa^1^; A. McGregor^1^; D. Katzenstein^2^; C.E. Ndhlovu^1^ and T.A. Makadzange^1^**

^1^University of Zimbabwe College of Health Sciences, Parirenyatwa Hospital, Harare, Zimbabwe, ^2^Biomedical Research and Training Institute, Harare, Zimbabwe

**Background: **Chronically infected youth receiving antiretroviral therapy (ART) struggle to maintain virologic suppression. In sub‐Saharan Africa youth who are viremic on ART, have a high frequency of drug resistance mutations (DRMs) and limited affordable therapeutic options. The integrase strand inhibitor (INSTI) dolutegravir (DTG) combined with tenofovir (TDF) and lamivudine (3TC) (TLD) is a new single tablet regimen (STR) proposed for use in public ART programmes in Africa.

**Methods: **We established a cohort of HIV‐1 infected youth on long‐term 1^st^ and 2^nd^ line ART with confirmed virologic failure (VL > 1000 copies/mL). A genotypic analysis of plasma virus was conducted and susceptibility scores to TLD and current 2^nd^ line therapies were calculated.

**Results: **Plasma virus from 160/185 (86%) participants was sequenced; 112(70%) on 1^st^ line and 48 (30%) on 2^nd^ line regimens. Median (IQR) age was 18 (15 to 19) years, and median duration on ART(IQR) was 6(4 to 8) years. Median (IQR) viral load was 4.51 (4.05 to 4.93) log10 copies/ml. DRMs were present in 94% and 67% of 1^st^ and 2^nd^ line failures respectively (*p* < 0.001). The lower rate of DRMs on 2^nd^ line therapy suggests PI use may reflect poor adherence and poor tolerance. Dual class resistance to NRTIs and NNRTIs was detected in 96 (60%) of 1^st^ line failures; PI DRMs were detected in a minority (10%) of subjects failing 2^nd^ line regimens. A total genotypic susceptibility score (tGSS) ≤ 2 that may potentially result in PI or DTG monotherapy, was observed in 11% and 42% of 1^st^ line failures switching to current PI based 2^nd^ line therapies and TLD respectively. The substitution of AZT for TDF in TLD could optimize 2^nd^ line therapy to achieve a tGSS > 2.

**Conclusions: **Current recommended PI based 2^nd^ line therapies may provide effective treatment for viremic youth failing 1^st^ line ART, but are poorly tolerated and demonstrate low rates of adherence. In 1^st^ line failure, TLD in the absence of genotyping may not be an optimal choice. Drug resistance data will inform strategies for the implementation of TLD as 2^nd^ and 3^rd^ line ART, while novel combinations and/or new agents are needed for this hard to treat population that requires decades of ART.

### WEAB0204

#### Cognitive function among cART‐treated children and adolescents with HIV in Zambia: Results from the HIV‐associated neurocognitive disorders in Zambia (HANDZ) study

**S. Mwanza‐Kabaghe^1,2^; H. Adams^3^; E. Grecian Mbewe^1^; P. Kabundula^1^; M. Mwiya^2^; C. Kankasa^2^; G.L. Birbeck^3^ and D.R. Bearden^3^**

^1^University of Zambia, Department of Educational Psychology, Sociology and Special Education, Lusaka, Zambia, ^2^University Teaching Hospital ‐ Paediatric HIV Center of Excellence, Lusaka, Zambia, ^3^University of Rochester Medical Centre, New York, United States

**Background: **A number of studies have demonstrated that children with Human Immunodeficiency Virus (HIV) are at increased risk for impaired cognition. Prior studies have been limited by including a mix of treated and untreated subjects, focusing on a restricted age range, and/or failure to include an appropriate control group. As part of the ongoing HIV‐Associated Neurocognitive Disorders in Zambia (HANDZ) study, we sought to evaluate cognitive function in virally suppressed cART‐treated children and adolescents living with HIV in Zambia compared to demographically matched HIV‐exposed uninfected controls

**Methods: **A total of 400 participants were recruited for the study consisting of 200 cART‐treated subjects with perinatally acquired HIV and 200 HIV exposed uninfected (HEU) controls, all 8 to 17 years old. Subjects with a history of CNS infection, pregnancy, epilepsy, or chronic kidney or liver disease were excluded. Demographics and subject characteristics were assessed using standardized subject and parent interviews, and comprehensive neuropsychological testing was performed using a combination of standard testing and iPad‐based performance measures using the NIH Toolbox. Cognitive impairment was defined using a global deficit score approach.

**Results: **In comparison to the HEU group, children with HIV performed significantly worse on a composite measure of cognitive function (Global Cognition standard score 82.8 vs. 74.8, *p* = 0.002), and were significantly more likely to be classified as impaired (34% vs. 5%, *p* = 0.001). Cognitive domains that were most affected included Attention, Working memory, Processing speed and Psychomotor Speed. In a multivariable logistic regression model, risk factors for impairment included socioeconomic status (OR 0.78), history of advanced WHO clinical stage (OR 1.9), late initiation of antiretroviral therapy(OR 2.0), and growth stunting (OR 2.7).

**Conclusions: **Cognitive function remains significantly worse in children with HIV compared to demographically similar controls, even in a relatively healthy population of cART‐treated virally suppressed subjects. There is a need for trials of interventions to improve development and cognitive function in children with HIV. This study suggests interventions to improve cognition in children with HIV might include earlier identification of subjects with HIV to initiate cART, and interventions to target lower SES families, such as cash transfers and nutrition support programmes.

### WEAB0205

#### Response to direct acting antivirals in vertically HIV/HCV co‐infected youths

**I. Carrasco^1^; T. Sáinz^2^; M.A. Frick^3^; S. Jiménez de Ory^1^; M. Montero^4^; C. Gavilán^5^; M.D. Falcón^6^; J.A. Couceiro^7^; J.I. Bernardino^2^; R. Rubio^8^; O. Bisbal^8^; C. Guerrero^9^; M.T. Aldámiz‐Echevarría^1^; P. Miralles^1^; J. Berenguer^1^; M.L. Navarro^1^ and CoRISpe‐ and Spanish National Cohort of HIV‐infected Children Adolescents**

^1^IiSGM ‐ Gregorio Marañon University Hospital, Madrid, Spain, ^2^La Paz University Hospital, Madrid, Spain, ^3^Vall d'Hebron University Hospital, Barcelona, Spain, ^4^La Fe University and Polytechnic Hospital, Valencia, Spain, ^5^San Juan de Alicante Hospital, Alicante, Spain, ^6^Virgen del Rocío University Hospital, Sevilla, Spain, ^7^Pontevedra Hospital Complex, Pontevedra, Spain, ^8^12 de Octubre Hospital, Madrid, Spain, ^9^Miguel Servet University Hospital, Madrid, Spain

**Background: **New direct acting‐antivirals (DAA) have altered HCV treatment in recent years. The absence of authorized drugs in children along with the natural evolution of the infection in childhood, generally asymptomatic until adolescence, results in little treatment experience in the population of vertically HIC/HCV co‐infected subjects. The objective of this study is to describe response to DAA treatment in this unique population.

**Methods: **Longitudinal observational study within The Spanish National Cohort of HIV‐infected children and adolescents (CoRISpe) including vertically HIV/HCV co‐infected children that had received treatment against HCV when visiting adult units. Demographic, analytical, clinical and virological parameters were collected before, and 12 weeks after finishing HCV treatment.

**Results: **From the 651 patients transferred to adult units, 80 were HCV co‐infected. Thirty‐four were excluded due to data unavailability and 46 were included in the analysis (3 of them lost to follow‐up and 5 deceased). 52.2% were women, median age of 26.5 years (IQR 24 to 30). In total, 30 patients had received treatment, at a median age of 22 years (IQR 19.7 to 25). At HCV‐treatment initiation, all patients were on ART, 92% virollogically‐suppressed, and a median CD4 T‐cell count of 646 cel/ul (IQR 551 to 1039), 13.3% below CD4 < 500cel/ul.

Genotipically, 60.6% were G1, 22.5%‐G4 and 15%‐G3. At treatment initiation, 24.1% presented fibrosis (F3‐F4), 17.2% F2 and 55% F0‐F1. Overall, 70% were treated with DAA; SOF/LED (14 patients), EBV/GZP (2p), OBV/PTR/r (2p), OBV/DSV/PTR/r (2p) y VLP/SOF (1p), plus RBV in 23%. Nine patients received interferon‐therapies; IFNpeg+RBV (7p), IFN/DCV+RBV (1p) and IFN/TPV+RBV (1p). DAA‐therapies were 8 to 12 weeks long while IFN therapies were from 12 to 48 weeks. The SVR rate with DAA was 100%, but 88.8% when IFNpeg+RBV regimens were used. After SVR at week 12 (SVR12), 38.5% improved their fibrosis stage, 15.4% worsened and 46.2% maintained their previous stage of fibrosis.

**Conclusions: **In our study, new DAA treatment guidelines achieved excellent cure rates (100%) in vertically HIV/HCV co‐infected patients. However, 24.1% of these patients showed advanced fibrosis (F3‐F4) at treatment initiation with no improvement despite treatment in 60%. To speed up access to new DAA treatments for pediatric populations is an urgent need.

### WEAB0301

#### Improved survival for people living with HIV, with and without tuberculosis, over time in Latin America

**S. Koenig^1^; A. Kim^2^; B. Shepherd^2^; C. Cesar^3^; V.G. Veloso^4^; C. Cortes^5^; D. Padgett^6^; B. Crabtree‐Ramirez^7^; E. Gotuzzo^8^; C. McGowan^2^; T. Sterling^2^ and J. Pape^9^**

^1^Brigham and Women's Hospital, Boston, United States, ^2^Vanderbilt University, Nashville, United States, ^3^Fundación Huésped, Buenos Aires, Argentina, ^4^Fundação Oswaldo Cruz, Rio de Janeiro, Brazil, ^5^University of Chile, Santiago, Chile, ^6^Hospital Escuela, Tegucigalpa, Honduras, ^7^INCMNSZ, Mexico City, Mexico, ^8^Hospital Nacional Cayetano Heredia, Lima, Peru, ^9^GHESKIO, Port‐au‐Prince, Haiti

**Background: **In 2006, 2009, and 2013, ART was recommended for persons living with HIV (PLWH) with CD4 count < 200 cells/mm^3^,< 350 cells/mm^3^, and < 500 cells/mm^3^, respectively. In 2015, universal ART was recommended. Earlier ART initiation was also recommended for patients with TB. We previously found that time from enrollment to ART initiation in Latin America dramatically decreased during this period. Here we characterize temporal trends in one‐year mortality, stratified by baseline TB status.

**Methods: **The study included PLWH from clinic sites in Brazil, Chile, Haiti, Honduras, Mexico, and Peru participating in CCASAnet. We included all persons ≥ 18 years old who were ART‐naïve at first clinic visit from 2006 to 2015. Baseline TB was defined as TB diagnosed within 30 days of enrollment. We estimated the probability of mortality within the first year of enrollment as a function of baseline TB status, CD4 count, and year of enrollment from a multivariable Cox regression model that included these variables, two‐way interactions between these variables, sex, education, and age, stratified by study site. Continuous variables were fit with natural splines to relax linearity assumptions.

**Results: **Of 19,197 patients, 1306 (7%) were diagnosed with baseline TB. Patients with TB were more likely to be male, older, less educated, have lower CD4 counts, and live in Haiti or Peru. Mortality was higher among patients with baseline TB (*p* = 0.003) but both groups had improved survival with later year of enrollment (*p* < 0.001). Survival was associated with higher baseline CD4 count (Figure) and improved over time for patients in all CD4 strata except for those with baseline TB and low CD4 counts, although the interaction between time and TB status was not statistically significant (*p* = 0.45).

**Conclusions: **Among PLWH in Latin America, mortality rates were higher in persons who presented with baseline TB, although survival improved over time regardless of TB status.

Abstract WEAB0301‐Figure 1. Estimated probability of one‐year survival.

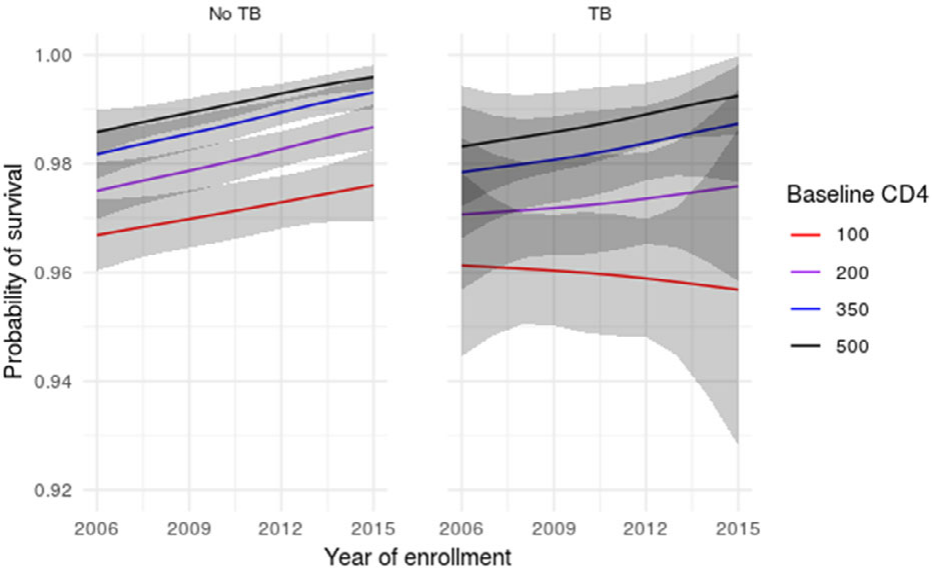

### WEAB0302

#### Social network characteristics are associated with prevalent tuberculosis infection among people living with and without HIV in nine communities in rural Uganda

**C. Marquez^1^; Y. Chen^2^; M. Atukunda^3^; J. Kironde^3^; G. Chamie^1^; L.B. Balzer^4^; L.B. Brown^1^; D. Kwariisima^3,5^; T.D. Clark^1^; M.R. Kamya^3,5^; E.D. Charlebois^1^; M.L. Petersen^6^; D.V. Havlir^1^ and SEARCH Collaboration**

^1^University of California, San Francisco, United States, ^2^University of Washington, Seattle, United States, ^3^Infectious Diseases Research Collaboration, Kampala, Uganda, ^4^University of Massachusetts, Amherst, United States, ^5^Makerere University College of Health Sciences, Kampala, Uganda, ^6^University of California, Berkeley, United States

**Background: **Social network analysis (SNA) has the potential to elucidate tuberculosis (TB) transmission dynamics between community‐based contacts, which are estimated to account for the majority of new TB cases.

**Methods: **We assessed the associations between social network characteristics and prevalent TB infection among adults (≥15 years) living in 9 rural communities in Uganda participating in the SEARCH Trial (NCT01864603). We built community‐wide social networks, excluding household members, using data from a baseline census from 2013 to 2014. Among individuals who received a tuberculin skin (TST) as part of a household survey enriched for persons living with HIV (PLWH), we evaluated whether network characteristics predicted prevalent TB infection, defined as a positive TST with induration > 10 mm or > 5 mm in PLWH, after adjusting for individual‐level variables (age, sex, TB contact, wealth, and BCG vaccination) with Targeted Maximum Likelihood. Network clustering was assessed with a permutation test.

**Results: **Among the 3355 adults surveyed, 32% had a positive TST, 20% were PLWH, and 4% reported a household TB contact. 2395 (75%) were linked in the non‐household network (Figure 1). Clustering by TST status was statistically significant (*p* < 0.05) in 4 communities. Adjusting for individual‐level risk factors, individuals with the most connections (top 10%) were more likely than those in the bottom 90% to have prevalent TB (aRR: 1.3, 95% CI: 1.1 to 1.5). Persons with more (top 10%) contacts living with HIV and more (top 10%) male contacts had a higher risk of prevalent TB, aRR: 1.21 (95% CI 1.1 to 1.4) and aRR: 1.5 (95% CI: 1.4 to 1.8) respectively, compared to those in the remaining 90%.

**Conclusions: **A high network degree and characteristics of the people in one's network, specifically having more links with men and PLWH, are associated with a higher risk of prevalent TB. TB transmission within a social network may explain prevalent TB not associated with a household contacts.

Abstract WEAB0302‐Figure 1. Visualization of a social network in one rural community in Eastern Ugandan, by tubercullin skin test (TST) status. In this community, clustering by TST status (positive pairs and negative pairs was statistically significant (*p* < 0.001) using a permutation test.

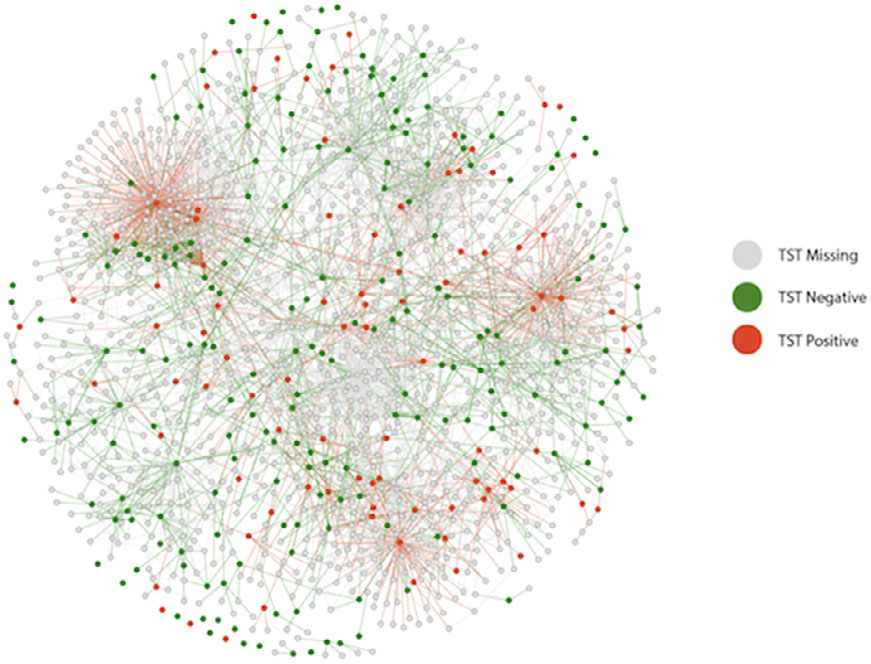

### WEAB0303

#### HCV reinfection among HIV/HCV co‐infected individuals in Europe

**S. Amele^1^; L. Peters^2^; A. Rodger^1^; L. Vandekerckhove^3^; T. Benfield^4^; A. Milinkovic^5^; C. Duvivier^6^; H.‐J. Stellbrink^7^; H. Sambatakou^8^; N. Chkhartishvili^9^; L. Caldeira^10^; M. Laguno^11^; P. Domingo^12^; G. Wandeler^13^; R. Zangerle^14^; E. Kuzovatova^15^; G. Dragovic^16^; B. Knysz^17^; R. Matulionyte^18^; J. Rockstroh^19^; J. Lundgren^2^; A. Mocroft^1^ and The EuroSIDA Study Group**

^1^UCL, Centre for Clinical Research, Epidemiology, Modelling and Evaluation, Institute for Global Health, London, United Kingdom, ^2^CHIP, Department of Infectious Diseases, Rigshospitalet, Copenhagen, Denmark, ^3^Ghent University Hospital, Ghent, Belgium, ^4^Hvidovre Hospital, Hvidovre, Denmark, ^5^Chelsea and Westminster Hospital, London, United Kingdom, ^6^Hôpital Necker‐Enfants Malades, Paris, France, ^7^ICH Study Center, Hamburg, Germany, ^8^Ippokration General Hospital, Athens, Greece, ^9^Infectious Diseases, AIDS & Clinical Immunology Research Center, Tbilisi, Georgia, ^10^Santa Maria University Hospital, University of Lisbon, Lisbon, Portugal, ^11^Infectious Diseases Service, Hospital Clinic, Barcelona, Spain, ^12^Hospital de la Santa Creu i Sant Pau, Barcelona, Spain, ^13^University of Bern, Department of Infectious Diseases, Bern University Hospital, Bern, Switzerland, ^14^Medical University of Innsbruck, Innsbruck, Austria, ^15^Nizhny Novgorod Scientific and Research Institute, Nizhny Novgorod, Russian Federation, ^16^University of Belgrade, School of Medicine, Belgrade, Serbia, ^17^Wroclaw Medical University, Wroclaw, Poland, ^18^Vilnius University Hospital Santaros Klinikos, Vilnius, Lithuania, ^19^Universitäts Klinik, Bonn, Germany

**Background: **While Directly Acting Antivirals (DAA) can clear HCV in nearly all HIV/HCV co‐infected individuals, high rates of reinfection may hamper efforts to eliminate HCV in this population. This analysis aimed to examine reinfection after achieving sustained virologic response (SVR) in HIV/HCV co‐infected individuals in Europe.

**Methods: **Individuals from EuroSIDA that achieved SVR 12 or 24, with ≥ 24 months follow‐up and ≥ 1 HCV‐RNA test after SVR were included. Factors associated with the odds of reinfection were assessed using multivariable logistic regression.

**Results: **Overall, 675 individuals were included. The median age was 45.9 (IQR 39.8 to 51.5 years), 78.4% were male, 48.7% were IDUs, 30.2% were MSM, and the majority received an interferon‐based regimen (610, 90.4%). Overall, 89 (13.2%, 95% confidence interval (CI) 10.6%‐15.7%) individuals were re‐infected by 24 months. Central‐East Europe had the highest proportion of reinfections (20.0%), while Southern Europe had the lowest (8.0%; *p* = 0.0097). Reinfections in MSM were 14.2%, similar to IDUs (13.5%; *p* = 0.67). After adjustment, Central‐West and Central‐East Europe had higher odds of reinfection (compared to Southern Europe; Figure 1), as did those with CD4 count > 500 cells/mm^3^. Those who achieved SVR ≥ 2014 had lower odds of reinfection There was no statistically significant association between age, gender, prior use of treatment, use of DAAs and reinfection, although all had wide confidence intervals.

**Conclusions: **The proportion of reinfections among HIV/HCV co‐infected individuals within 24 months of achieving SVR was 13%, with evidence suggesting this is decreasing over time. We cannot rule out that some late relapses could have been misclassified as reinfection, though this is unlikely, and that clinics have targeted testing to those at highest risk or with signs of reinfection. Active surveillance to detect early HCV reinfection with an offer of early treatment is essential as is harm reduction in those treated to reduce rates of reinfection.

Abstract WEAB0303‐Figure 1. Adjusted odds ratio (aOR) of being reinfected‡ after achieving SVR§.

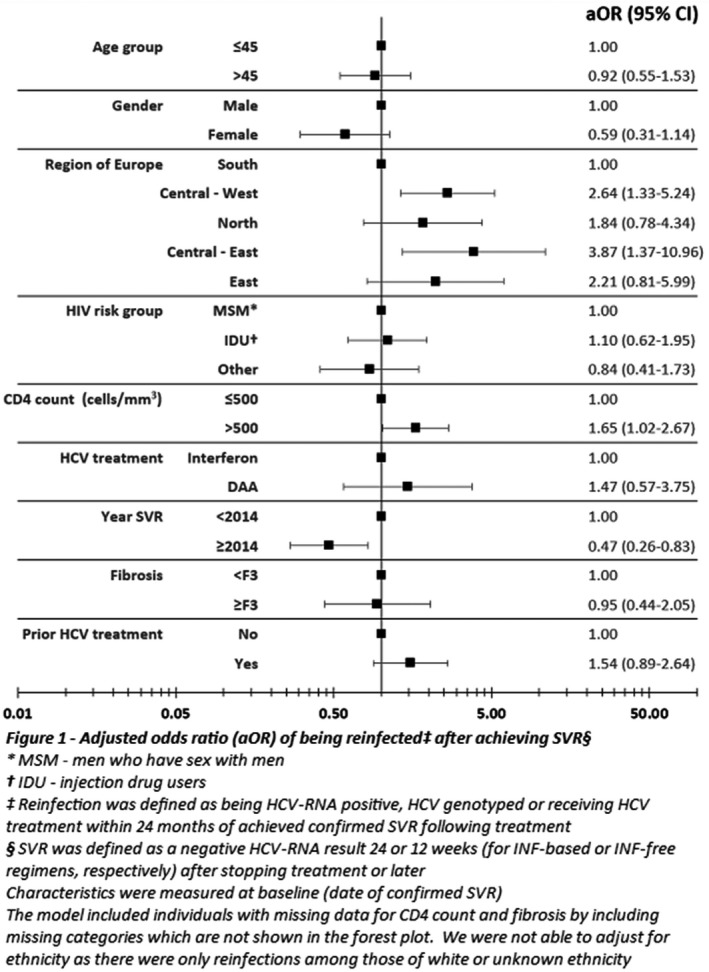

### WEAB0304

#### Evaluation of hepatitis C virus rapid diagnostic test in HCV mono‐ and HCV/HIV co‐infected patients from low and middle income countries

**B. Vetter^1^; E. Ivanova Reipold^1^; R. Audu^2^; M. Alkhazashvili^3^; A. De Weggheleire^4^; S. Ongarello^1^ and K. Fransen^4^**

^1^Foundation for Innovative New Diagnostics, Geneva, Switzerland, ^2^Nigerian Institute of Medical Research, NIMR, Lagos, Nigeria, ^3^National Centre for Diseases Control and Public Health Georgia, Lugar Centre, Tbilisi, Georgia, ^4^Institute of Tropical Medicine, Antwerp, Belgium

**Background: **Hepatitis C Virus (HCV) rapid diagnostic tests (RDTs) for the detection of anti‐HCV antibodies play an important role in reaching high risk populations in low and middle income countries (LMICs).

Past studies have compared performance of HCV RDTs mostly in HCV‐mono infected patients, often located in high income countries. Although HIV is a commonly found co‐infection in HCV‐infected individuals, substantial data on RDT performance in HCV/HIV co‐infected patients are lacking.

In the present study, we aimed to evaluate sensitivity, specificity and performance characteristics of thirteen RDTs on 1'800 HCV mono‐ or HCV/HIV co‐infected samples collected in different geographic regions, to fill the data gap in LMICs and particularly the HIV co‐infected population.

**Methods: **This is an observational, retrospective multicentre laboratory study on archived EDTA plasma samples from Cambodia, Nigeria, Georgia and Belgium.

Sensitivity and specificity were evaluated in each of 400 samples of HCV mono‐infected and HCV/HIV co‐infected individuals and each of 500 samples of HCV‐uninfected or HIV mono‐infected individuals, respectively. Results were compared to a reference method composed of two Enzyme Immuno Assays and a Line Immunoassay.

Each sample was tested on two lots per RDT and each RDT result was read by three independent readers. Additionally, the rate of invalid runs was assessed.

**Results: **This large head‐to‐head performance evaluation provides insightful information on the RDTs performance of sensitivity versus specificity in the HCV mono‐ and HCV/HIV co‐infected sample cohorts. While some RDTs showed very high specificity (up to 100%), the sensitivity was variable (81 to 100%), particularly in the HCV/HIV co‐infected sample cohort. Conversely, where sensitivity was high, there was a clear compromise in specificity and once again, this effect was more pronounced in the HCV/HIV co‐infected samples.

Inter‐lot and inter‐reader variability was mostly within an acceptable range (≤3%), while the rate of invalid runs appeared to be related to the technical configuration of the test.

**Conclusions: **The results of our study provide a comprehensive picture of HCV RDT performance in patients from different LMICs, with and without HIV co‐infection. Performance appeared impacted by HIV co‐infection, which should be taking into consideration when deciding on the most suitable HCV RDT for screening.

### WEAB0305

#### Prevalence, characteristics and outcomes of patients with Cryptococcal meningitis in Maputo, Mozambique

**R. Deiss^1,2^; C. Loreti^3^; A. Gutierrez^1^; M. Tatia^2^; H. Vivaldo^1^; L. Molfino^1^; N. Tamayo Antabak^1^; S. Issufo^3^ and I. Ciglienicki^4^**

^1^Medecins Sans Frontieres, Maputo, Mozambique, ^2^Hospital Geral Jose Macamo, Ministerio da Saude de Mocambique, Maputo, Mozambique, ^3^Ministerio da Saude de Mocambique, Maputo, Mozambique, ^4^Medecins Sans Frontieres, Geneva, Switzerland

**Background: **Cryptococcal meningitis (CCM) is a leading cause of HIV‐related mortality in sub‐Saharan Africa. Nonetheless, routine screening for serum cryptococcal antigen (CrAg) has not been widely implemented for patients with advanced HIV, despite World Health Organization (WHO) recommendations. Moreover, CCM treatment in Africa is often suboptimal with inadequate access to liposomal amphotericin (LAmB) and flucytosine (5‐FC). We report outcomes following implementation of systematic screening, diagnosis and treatment of CCM at a tertiary level hospital in Maputo, Mozambique.

**Methods: **We analysed retrospective clinical data between March‐December 2018. All HIV+ patients admitted to the emergency department underwent CD4 count screening; those with CD4 < 200 cells/ul received point‐of‐care serum CrAg testing (IMMY; Norman, OK, USA). Cerebrospinal fluid (CSF) was obtained on serum CrAg+ patients; those with CSF CrAg+ received 7 days of LAmB/5‐FC followed by fluconazole. Post‐discharge outcomes were obtained by telephone contact and/or review of ambulatory records.

**Results: **Among 1782 patients screened, the total prevalence of cryptococcal antigenemia was 5.6% (N = 100). Of these, 61% were diagnosed with CCM, yielding a total prevalence of 3.4%. In the overall sample, 49.9% (N = 890) of individuals had CD4 < 200 cells/uL; additional patient characteristics are highlighted in Table 1. Complete induction treatment data was available on 40/61 patients: 32 (80%) completed and eight (20%) died during hospitalization. At 12 weeks, 18% (11/61) patients remained in ambulatory care, 34% (21/61) had died, and 34% (21/61) could not be reached. An additional eight patients (13%) had not yet completed 12 weeks of follow‐up.

Abstract WEAB0305‐Table 1.Characteristics of patients admitted to emergency department with CD4 < 200 cells/ul

**Table:** 

	Serum CrAg+ (N = 100)	Serum CrAg‐ (N = 890)	CSF CrAg+ (N = 61)	CrAg‐ CSF (N = 27)^a^
Median Age (IQR)	38 (33, 43)	38 (32, 45)	38 (33.5, 43.5)	35 (30, 42)
Male [N; (%)]	53 (53)	423 (47.5)	33 (54.1)	12 (44.4)
Median CD4 (IQR)	32 (15.5, 75.5)	51 (22, 102)	34 (16.5, 82)	38 (14.5, 134)

^a^CSF not obtained in 12 serum CrAg+ patients.

**Conclusions: **This is the first report of CCM prevalence and treatment outcomes in Maputo, Mozambique. Introduction of serum CrAg screening and CCM diagnosis for advanced HIV disease in an emergency department was feasible. Treatment with LAmB/5‐FC resulted in high early survival, but a high proportion of deaths occurred in the early post‐hospitalization period, emphasizing the need for close outpatient monitoring following initial CCM treatment.

### WEAB0306

#### HPV genotyping is as important as cytology in anal cancer early diagnosis

**M. Digaetano^1,2^; F. Spatafora^3^; C. Rogati^1^; A. Farinetti^3^; R. Gelmini^3^; M. Pecorari^4^; S. Tagliazucchi^4^; R.P. Iachetta^5^; R.D. Villani^5^ and C. Mussini^1,3^**

^1^AOU Policlinico di Modena, Infectious Diseases Clinic, Modena, Italy, ^2^Alma Mater Studiorum ‐ Università degli Studi di Bologna, Scuola di Specializzazione in Malattie Infettive, Bologna, Italy, ^3^University of Modena and Reggio Emilia, Surgical, Medical and Dental Department of Morphological Sciences Related to Transplant, Oncology and Regenerative Medicine, Modena, Italy, ^4^AOU Policlinico di Modena, Unit of Microbiology and Virology, Modena, Italy, ^5^Nuovo Ospedale Civile di Sassuolo (Modena), Pelvic Floor Center, Department of Proctology, Sassuolo, Italy

**Background: **Anal cancer is increasing among HIV+ men who have sex with men (MSM). Our study aims to identify the best method of screening.

**Methods: **This prospective single centre study involved HIV+ MSM who underwent an anal cancer screening programme using the anal Pap test, HPV genotyping and, in case of positive cytology or high risk HPV genotype detection, high resolution anoscopy (HRA).

**Results: **121 performed Pap tests were performed: 50 (41.3% of) were positive for HPV related lesions, 44 (88%) low grade squamous intra‐epithelial lesions (LSIL) and 5 (10%) atypical squamous cells of undetermined significance (ASCUS), high grade lesions (HSIL) were found in 1 patient (2%). 71 of 121 screened (58.6%) performed also HPV genotyping: 3 resulted negative, 11 low risk HPV genotype carriers and 57 high risk HPV (HRHPV) genotype carriers. HPV16 was found in 13 patients (18.3%) and it was the most frequently identified genotype. HPV18 was found in 8 patients (11.2%). Only 21 HRHPV carriers (37%) had a positive Pap test. A total 86 screened (71.7%) had the indication for HRA (50 positive Pap tests plus 36 HRHPV with negative cytology). Among 42 HRAs performed until today (39 in Pap test + and 3 in HRHPV carriers with Pap test ‐), 21 (50%) showed LSIL and 8 HSIL (19%). 2 HSILs were found in HRHPV carriers with Pap test ‐, 6 in Pap test + . HRA confirmed cytology in 23 cases, showed a worsening in 7 cases and a lower grade lesion in 11 cases. 3 cases of clinical progression were detected at control, 1 with HRHPV but negative cytology became LSIL, 1 LSIL became HSIL and 1 carcinoma in situ was found in a patient treated for HSIL four months before.

**Conclusions: **HPV‐related dysplasia is common among HIV+ MSM and is likely to evolve in a short period of time especially in the presence of high‐risk genotypes. We recommend the association of HPV genotyping with cytology as first level of screening and HRA for treatment and follow up of lesions as this bundle allows to identify lesions in subjects with a negative PAP‐test.

### WEAC0101

#### Transmission Linkages among persons with incident HIV‐1 infection in North Carolina, 2014 to 2018

**A. Dennis^1^; S. Frost^2^; A. Cressman^3^; N. Adams^4^; J. Eron^1^; W. Miller^5^; M. Cohen^6^; V. Mobley^4^ and E. Samoff^4^**

^1^University of North Carolina, Chapel Hill, United States, ^2^University of Cambridge, Cambridge, United Kingdom, ^3^UNC Chapel Hill, Chapel Hill, United States, ^4^North Carolina Division of Public Health, Raleigh, United States, ^5^The Ohio State University, Columbus, United States, ^6^University of North Carolina at Chapel Hill, Chapel Hill, United States

**Background: **Despite widespread prevention, HIV incidence in the Southern US remain stable to rising among young MSM (YMSM, < 30 years) subgroups. Innovative strategies to increase engagement and retention in HIV care, such as through network‐based recruitment, are needed. We evaluated features of transmission networks involving persons diagnosed during primary HIV infection (PHI) to assess network‐based opportunities for intervention.

**Methods: **We investigated genetic clusters involving persons diagnosed with PHI from 2014 to 2018 and reported to North Carolina (NC) surveillance. PHI is defined as acute (negative antibodies with detectable RNA) or recent infection (positive antibody within 3 months of seronegative testing). Pol sequences generated from resistance genotypes are routinely reported to surveillance and analysed for clustering. Clusters were defined as groups with < 1.5% genetic distance between all sequences and involving ≥ 1 PHI case. Assortativity coefficients (r) were calculated to estimate mixing among cluster features in the network.

**Results: **Of 296 persons (7.2%) reported with PHI among 4105 HIV diagnoses 2014 to 2018 with sequences, most were male (89%), black (64%), young (44% 18 to 24 years), and reported MSM risk (78%). Most PHI cases (n = 209; 71%) had a sequence linked to another sequence. These cases were in 152 clusters involving 1202 persons (PHI members: median 1, range 1 to 5). Of cluster members diagnosed during established infection, a substantial number were prior diagnoses (n = 297; 30% diagnosed < 2014). Among prior diagnoses, factors associated with membership in PHI clusters compared to persons not in a PHI cluster included: MSM risk (78% vs. 38%), age 18 to 24 years at diagnosis (54% vs. 22%), more recent HIV diagnosis (median 2011 vs. 2005), greater percentage time spent viremic (2014 to 2018: estimated mean 42% vs. 37% days above viral load 1500 copies/mL) [*p* < 0.01]. Positive assortativity was found for geographic region (r = 0.44), race (r = 0.22), risk (0.14), and age (r = 0.22).

**Conclusions: **We identified the transmission linkages for a high proportion of YMSM with PHI through statewide molecular surveillance. YMSM with prior diagnoses are frequently identified in these PHI clusters with high assortativity by demographic features. Enhanced partner services for YMSM to support retention in HIV care and facilitate further case finding may have a high impact on reducing onward transmission.

### WEAC0102

#### Applications of HIV genetic networks in Mexico: Implications for prevention

**S. Avila‐Rios^1^; A. Piñeirúa^2^; A. Chaillon^3^; C. García‐Morales^1^; D. Tapia‐Trejo^1^; M. Matías‐Florentino^1^; D.M. López‐Sánchez^1^; M. Pérez‐García^1^; H.E. Paz‐Juárez^1^; P.K. Jiménez‐Pastor^4^; M.A. Becerril‐Rodríguez^2,5^; S.J. del Arenal‐Sánchez^1^; V. Ruiz^5^; P. Iracheta‐Hernández^2^; I. Macías‐González^5^; J. Tena‐Sánchez^5^; B.M. Delgado‐Orozco^6^; L.A. González‐Hernández^7^; N.P. Quintero‐Pérez^8^; G. Amaya‐Tapia^9^; S.O. Ruiz‐Torres^10^; S.R. Mehta^3^; A.E. Campos‐Loza^4^; F. Badial^2^; A. González‐Rodríguez^5^ and G. Reyes‐Terán^1^**

^1^National Institute of Respiratory Diseases, Centre for Research in Infectious Diseases, Mexico City, Mexico, ^2^Clinica Especializada Condesa Iztapalapa, Mexico City, Mexico, ^3^University of California ‐ San Diego, Division of Infectious Diseases, San Diego, United States, ^4^COESIDA Jalisco, Zapopan, Mexico, ^5^Clinica Especializada Condesa, Mexico City, Mexico, ^6^Centro Estatal de Laboratorios, Secretaría de Salud, Guadalajara, Mexico, ^7^Hospital Civil de Guadalajara ‘Fray Antonio Alcalde’, Guadalajara, Mexico, ^8^Hospital Civil de Guadalajara ‘Dr. Juan I. Menchaca’, Guadalajara, Mexico, ^9^Hospital General de Occidente, Zapopan, Mexico, ^10^CAPASITS Puerto Vallarta, Puerto Vallarta, Mexico

**Background: **HIV genetic networks might help to understand HIV transmission dynamics and design more effective interventions. We present two examples of the use of HIV networks in different epidemiological contexts within Mexico: A state‐level molecular surveillance system in Jalisco and adolescents and young persons within the network of the Mexico City metropolitan area.

**Methods: **HIV *pol* Sanger sequences were obtained from 671 persons starting first‐line ART in Jalisco, 01/2017 to 11/2018. HIV *pol* sequences were obtained by next generation sequencing from 2447 individuals initiating first‐line ART at Condesa Clinic, 09/2016 to 06/2018. Genetic networks were inferred with HIV‐TRACE, establishing putative transmission links with genetic distances < 1.5%. Newman's assortativity coefficients were estimated using igraph.

**Results: **In the case of Jalisco, putative links with at least one other sequence were found for 258/671 (38.5%) sequences, forming 89 clusters from 2 to 9 individuals (57% dyads). The network was assortative by risk factor for HIV acquisition (*p* = 0.001) and municipality of residence (*p* < 0.001). Clustering individuals were younger (mean age: 31 vs. 33, *p* = 0.01), included a higher proportion of MSM (78% vs. 65%, *p* = 0.01), and were diagnosed more frequently by COESIDA community testing programme (69% vs. 61%, *p* = 0.02).

In the case of Mexico City, putative links were found for 963/2447 (39%) sequences, forming 326 clusters from 2 to 20 individuals. A higher proportion of young persons (≤ 21 yo) was observed within clusters (51% vs. 38%, *p* < 0.001). Of all putative links in the network (1158), 24% (278) included at least one young person. Within links including young persons, 42% (117/278) included one 22 to 25 yo, 38% (106/278) one 26 to 40 yo, 6% (16/278) one person > 40, and 23% (63/278) were between two persons ≤ 21 yo. Importantly, the inferred transmission network was not assortative by age (*p* = 0.6), suggesting frequent putative links between persons of diverse age groups.

**Conclusions: **We observed frequent HIV transmission among young MSM in a geographically assortative network in Jalisco. We also observed higher detection of transmission chains through community testing, underscoring the importance of NGO participation in focused interventions. The inferred Mexico City HIV genetic network demonstrates the importance of persons ≤ 21 yo in putative transmission chains, with frequent links with older persons.

**Funding: **This work was supported by grants from the Mexican Government (Comisión de Equidad y Género de las Legislaturas LX‐LXI y Comisión de Igualdad de Género de la Legislatura LXII de la H. Cámara de Diputados de la República Mexicana) (GRT), CONACYT SALUD‐2017‐01‐289725 (SAR), and the US National Institutes of Health AI093163 (SRM).

### WEAC0103

#### High burden of recent and prevalent HIV infection among men who have sex with men (MSM) in Hanoi, Vietnam

**D. Vu^1^; G. Le^2,3^; L. An^2,3^; T. Thai^4^; R. Bhatia^1^; H. Bui^2,5^; T. Tran^1^; H. Bui^1^ and A. Abdul‐Quader^1^**

^1^Centers for Disease Control and Prevention, Vietnam Office, Hanoi, Vietnam, ^2^Hanoi Medical University, Centre for Research and Training on HIV/AIDS, Hanoi, Vietnam, ^3^Hanoi Medical University, Institute of Preventive Medicine and Public Health, Hanoi, Vietnam, ^4^Ho Chi Minh City University of Medicine and Pharmacy, Ho Chi Minh City, Vietnam, ^5^The Kirby Institute for Infection and Immunity, University of New South Wales, New South Wales, Australia

**Background: **HIV in Vietnam is concentrated in key populations, including MSM. The Asian Epidemic Mathematical model estimated a prevalence of 4.8% and annual incidence of 0.36% among MSM in Hanoi. Using a validated, novel rapid test to detect recent infection, we directly estimated and characterized HIV incidence in this population.

**Methods: **In 2017, we recruited 800 MSM aged > 16 years into the Hanoi MSM cohort using time‐location sampling based on comprehensive mapping of all MSM venues in the city. We collected baseline data on demographics, sexual and risk behaviours, healthcare seeking practices and performed HIV and STI testing. HIV positive cases underwent Asanté™ HIV‐1 Rapid Recency^®^ Assay testing and viral load testing. We used data on recent HIV infection (defined as Asanté recent and HIV ≥ 1000 copies/ml); a mean duration of recent infection (MDRI) of 161 [141 to 174] days; and a positive false rate (PFR) of 0 to derive annual incidence. We conducted weighted stratified analysis and logistic regression, taking into account variability of the venue size and selection probability.

**Results: **Of 80 HIV cases, 75 underwent Asanté testing. The HIV prevalence was 10.9% [7.4%‐15.8%] of which 21.6% [9.5 to 42.0%] were recently infected, yielding a weighted, annual HIV incidence of 5.8% [0.8%‐10.6%]. Nearly all (92.8% [72.5 to 98.4%]) recently‐infected MSM were ≤ 24 years old, with an average last‐month income of 232 ± 55 USD; 95.2% [79.9 to 99.0%] reported having sex with partners met via websites or mobile applications; 89.4% [69.5 to 96.9%] experienced STI symptoms in last six months. Few recently infected MSM had syphilis (12.2% [3.1 to 37.8%]) and reported using amphetamines during sex (chemsex) in the past six months (0.9% [0.1 to 6.0%]). Compared to long‐term infection, recent infection was associated with lower income of 130 USD (AOR 28.0 [1.0 to 774.2]); it's negatively associated with syphilis (AOR 0.04 [0.00 to 0.47]) and chemsex in the past six months (AOR 0.03 [0.00 to 0.48]).

**Conclusions: **Using a novel recency test, we found double the HIV prevalence and 16 times the HIV incidence than predicted by mathematical modelling for MSM in Hanoi. Our findings highlight the urgent need for enhanced prevention including routine HIV and STI screening, partner notification services and PrEP, especially in lower income MSM.

### WEAC0104

#### Acute HIV infection among individuals who start PrEP: The ImPrEP experience, a demonstration project in the context of combination prevention in Brazil, Mexico and Peru

**J.V. Guanira^1,2^; B. Hoagland^3^; G.M. Calvo^2^; S. Díaz^4^; K.A. Konda^2^; B. Grinsztejn^3^; E.H. Vega^5,6^; C.F. Cáceres^2^; V.G. Veloso^3^ and ImPrEP Study Team**

^1^Investigaciones Medicas en Salud, Lima, Peru, ^2^Universidad Peruana Cayetano Heredia, Centro de Investigación Interdisciplinaria en Sexualidad Sida y Sociedad (CIISSS), Lima, Peru, ^3^Instituto Nacional de Infectologia Evandro Chagas (INI‐Fiocruz), Rio de Janeiro, Brazil, ^4^Mexico City HIV/Aids Prevention and Comprehensive Care Center, Mexico City, Mexico, ^5^Condesa & Condesa‐Iztapalapa Specialized Clinics, Mexico City, Mexico, ^6^National Institute of Psychiatry Ramon de la Fuente Muñiz, Mexico City, Mexico

**Background: **Pre exposure prophylaxis (PrEP) is recommended as part of combination prevention for individuals at high risk for HIV infection. Before initiating PrEP, individuals must test HIV negative to avoid including HIV positives, thereby preventing resistance, and assure adequate care. During early acute HIV infection (AHI), neither antibodies nor antigens can be detected by serologic tests, but virus is detectable with molecular methods. Therefore, some individuals with AHI may be enrolled in PrEP, as molecular methods are not included in conventional PrEP screenng. ´ImPrEP´ is a PrEP demonstration study to assess feasibility of daily oral PrEP provided to MSM and transgender people at risk for HIV in Brazil, Mexico and Peru.

**Methods: **Individuals with AHI were defined as those enrolling with a negative HIV rapid test (4^th^generation in Mexico and Peru, 3^rd^ generation in Brazil) with detectable virus using molecular methods. At enrollment, all participants provide data related to potential factors for HIV acquisition (number of sexual partners, condomless anal sex, STIs, among others), use of post‐exposure prophylaxis in the last year and signs or symptoms suggestive of AHI in the last 30 days. At enrollment, participants only receive 1 month of PrEP, to assure the exclusion of individuals with AHI.

**Results: **From January‐December 2018, 3433 individuals were enrolled, 2160 in Brazil, 702 in Mexico and 571 in Peru. Nine individuals with AHI were identified. The overall prevalence of AHI visit was 0.26%, of which 0.28% in Brazil, 0.35% in Peru and 0.14% in Mexico. All AHI cases had 2 or more HIV risk factors and 3/9 had used PEP in the last year. PrEP exposure was short (8 ‐ 30 days). In Brazil, 5/6 started ART the day they stopped taking PrEP. In Peru and Mexico all the cases were referred to treatment and care facilities the day they stopped PrEP. Only one AHI case presented symptoms of potential acute infection at enrollment.

**Conclusions: **AHI is a rare event among ImPrEP participants, and initiation of PrEP the same day of screening is safe, only exposing those with AHI for a limited period, decreasing the theoretical risk of resistance.

### WEAC0105

#### HIV incidence among men who have sex with men and transgender women in Tijuana, Mexico

**B. Skaathun^1^; H.A. Pines^1^; T.L. Patterson^2^; A. Harvey‐Vera^3^; G. Rangel^4,5^; S.J. Semple^2^ and S.R. Mehta^1^**

^1^University of California, San Diego, Infectious Diseases and Global Public Health, San Diego, United States, ^2^University of California, San Diego, Psychiatry, La Jolla, United States, ^3^Universidad Xochicalco, Tijuana, Mexico, ^4^United States‐Mexico Border Health Commission, Tijuana, Mexico, ^5^El Colegio de la Frontera Norte, Tijuana, Mexico

**Background: **Accurate estimates of HIV incidence among men who have sex with men (MSM) and transgender women (TW) in Tijuana, Baja California, Mexico are unavailable despite being the most heavily impacted risk populations (HIV prevalence: MSM/TW = 20%, persons who inject drugs = 4%, female sex workers = 6%), with 89% of MSM/TW unaware of their infection. Without HIV incidence data, we cannot evaluate the impact of current interventions. This study estimates and characterizes recent HIV infection among a cohort of newly diagnosed HIV‐positive MSM/TW in Tijuana.

**Methods: **Recency was determined using Limiting Antigen (LAg)‐Avidity testing (detects infection ˜130 days) for the Enlaces cohort of newly‐diagnosed HIV‐positive MSM/TW (n = 195) recruited via venue‐based and respondent‐driven sampling in Tijuana (03/2015 to 11/2018). Logistic regression was used to determine characteristics associated with recent infection. We also examined whether recent infections clustered using HIV‐1 partial *pol* sequences generated from blood samples from 141 participants. We used HIV‐TRACE to measure TN93 genetic distances between all pairs of sequences and infer putative transmission links between participants whose sequences had genetic distances ≤ 1.5%. Clustering was defined as having ≥ 1 putative transmission link within the inferred HIV transmission network.

**Results: **Twenty‐two (11%) of the 195 participants tested had recent HIV‐infections. Of those with sequence data, 64% (9/14) of recent infections clustered, compared with 33% (38/116) of chronic infections. Two recent infections belonged to the same cluster (maximum cluster size:15). In the adjusted analysis, those with recent infection lived in Tijuana longer (OR 1.05; 95% CI 1.01 to 1.09) and were more likely to use cocaine (past month) (OR 8.57; 95% CI 2.18 to 33.69).

**Conclusions: **A high proportion of newly diagnosed MSM/TW in Tijuana were recently infected. The low clustering between the recent infections suggests continued onward HIV transmission, rather than a network outbreak. Given the majority of HIV‐positive MSM/TW in Tijuana are unaware, this estimate serves as a starting point for resource allocation.

Abstract WEAC0105‐Table 1. Estimated number of recent infections among MSM/TW, characteristics of those with recent infections

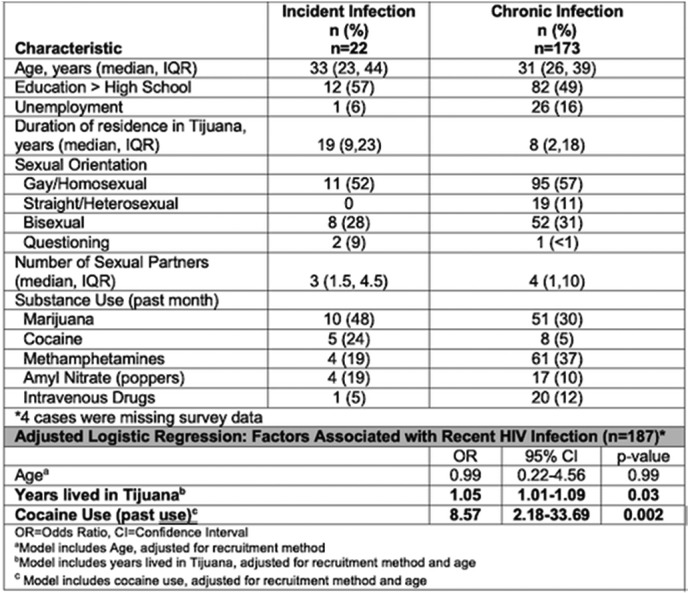

### WEAC0201

#### Does the use of HIV self‐testing kits lead to unintended effects? Evidence from female sex workers in Malawi

**P. Mee^1^; M. Neuman^1^; M. Kumwenda^2^; M. Sambo^2^; W. Lora^2^; P.P. Indravudh^1^; K. Hatzold^3^; C. Johnson^4^; E.L. Corbett^12^ and N. Desmond^2^**

^1^London School of Hygiene and Tropical Medicine, London, United Kingdom, ^2^Malawi‐Liverpool Wellcome Trust Clinical Research Programme, Blantyre, Malawi, ^3^Population Services International, Johannesburg, South Africa, ^4^World Health Organization, Department of HIV/AIDS, Geneva, Switzerland

**Background: **In Malawi, undiagnosed HIV has been highly prevalent among female sex workers (FSW). HIV self‐testing (HIVST) can be highly accurate, safe and effective when well supported, but concerns remain about unintended consequences particularly for vulnerable groups. Here we explore FSW experiences during the introduction of peer‐based HIVST services.

**Methods: **Existing peer‐educators were trained to provide oral kits and support HIVST and subsequent linkage to care and prevention by FSWs. Between March and September 2017, FSWs were recruited by the peer‐educators and given up to 2 HIVST kits. Interviews were conducted with FSWs at recruitment and 3‐months, including questions on coerced HIVST use or results disclosure, intimate partner violence (IPV), regrets about taking the HIVST and relationship problems.

**Results: **Of 131 SWs who completed both interviews and reported having used the HIVST, 11(8.4%) were first time testers. 87(12.6%) reported that HIVST had been initiated by themselves. 44 (22.7%) reported being pressured to self‐test or to share results, 42 by peer distributors and 2 by partners or spouses; of these, 5(3.8%) were also pressured to share their self‐test result. Immediate regrets about HIVST were expressed by 21(16.0%) and by 12(9.2%) 3‐months later, respectively, most commonly in FSWs who had not initiated HIVST themselves and for those aged < 26 years or > 36 years.

Abstract WEAC0201‐Table 1. Reports of regret and relationship problems at the 3 month interview

**Table:** 

			Immediate regret about HIVST		Regret now about HIVST		Relationship problems caused by HIVST	
Variable	Category	Total	%	*p*‐value	%	*p*‐value	%	*p*‐value
Test initiator	Self	87	12.6	0.20	9.2	1.0	3.4	0.06
	Other	44	22.7		9.1		13.6	
HIVST result	Positive	45	15.6	1.00	6.7	0.54	8.9	0.25
	Negative	86	16.3		10.5		5.8	
Age in years	16 ‐ 25	66	18.2	0.40	16.7	<0.01	9.1	
	26 ‐ 35	53	11.3		0.0		3.8	0.51
	> 36	12	25.0		8.3		8.3	
	TOTAL	131	16.0		9.2		6.9	

High rates of IPV in the previous 3‐months were reported (48.4% at enrolment and 30.5% at 3‐months).

**Conclusions: **Introducing HIVST through peer‐distributors in Malawi led to frequent experiences of FSWs feeling pressurised into testing and sharing results, and frequent expression of regrets and relationship difficulties ‐ although regrets diminished over time. Background rates of IPV were high. Care needs to be taken when introducing HIVST to ensure uptake is voluntary. Alternative strategies to the use of peer‐distributors among FSW should be explored.

### WEAC0202

#### Closing the testing gap: High uptake of HIV self‐testing among men in rural and peri‐urban KwaZulu‐Natal, South Africa

**A.E. Shapiro^1,2^; A. van Heerden^3^; M. Krows^1^; K. Sausi^3^; N. Sithole^4^; T.T. Schaafsma^1^; O. Koole^4^; H. van Rooyen^3,5^; D. Pillay^4^; C.L. Celum^1,2,6^ and R.V. Barnabas^1,2,6^**

^1^University of Washington, International Clinical Research Center, Department of Global Health, Seattle, United States, ^2^University of Washington, Department of Medicine, Division of Allergy and Infectious Diseases, Seattle, United States, ^3^Human Sciences Research Council, Sweetwaters, South Africa, ^4^Africa Health Research Institute, Mtubatuba, South Africa, ^5^University of Witwatersrand, School of Clinical Medicine, Faculty of Health Sciences, Johannesburg, South Africa, ^6^University of Washington, Department of Epidemiology, Seattle, United States

**Background: **In South Africa, HIV‐infected men are less likely than women to test and know their status (the first UNAIDS “90‐90‐90” target), and men have worse outcomes across the HIV care cascade. HIV self‐testing (HIVST) has the potential to address this testing disparity but questions remain over the venues for distribution and linkage following a positive test result.

**Methods: **We conducted an implementation study of multi‐venue HIVST kit distribution targeting men in two rural and peri‐urban regions in KwaZulu‐Natal (KZN), South Africa. We distributed HIVST kits at community points, workplace, and social venues for either on‐site or take‐home use. Clients could choose blood or oral fluid tests and elect to watch an in‐person or video demonstration. We provided a USD2 incentive to facilitate reporting test results by phone or SMS. Persons with positive screen results were provided immediate (if used HIVST on‐site) or were referred to confirmatory testing (if took HIVST home) with linkage to care.

**Results: **Between July‐November 2018, we distributed 4355 HIVSS kits in 2 regions of KZN (96% to men, median age 28 (IQR 23‐35). A majority (N = 2488, 57%) chose blood‐based HIVST and (N = 1867, 43%) chose oral‐swab kits. 11% of men were testing for the first time and 40% had last tested more than 12 months ago. 2692 (62%) of testers reported their test result to the study team to date, with 244 (9%) screening positive. 1279 (48%) used the kit at home. 25% of testers reported receiving assistance using the kit. 10% of kit users reported they would have preferred a different type (oral vs blood) of kit than the type they used.

**Conclusions: **HIVST is acceptable to men and rapid distribution feasible (>1000 kits per month) in rural and peri‐urban settings. HIVST kits successfully reached the key population of younger men and identified undetected infections. A majority chose blood‐based HIVST. Scaling up HIVST distribution and guidance may increase the number of first‐time testers among men and help achieve the first UNAIDS “90” for men in South Africa.

Abstract WEAC0202‐Table 1. HIV self‐testing (HIVST) kits distributed in KwaZulu‐Natal regions, Jul‐Nov 2018

**Table:** 

Total kits distributed	Peri‐urban area (N = 1870)	Rural area (N = 2485)
Atomo i‐Test, OraQuick	984 (53%), 886 (47%)	1504 (61%), 981 (40%)
*HIVST results reported*	*1490 (80%)*	*1202 (48%)*
HIVST result: Positive, Negative	145 (10%), 1345 (90%)	99 (8%), 1103 (92%)

### WEAC0203

#### Peer‐mobilized HIV self‐testing increases case detection and linkage to ART among key populations in Burundi

**D. Gashobotse^1^; T. Lillie^2^; G. Kamariza^1^; A. Nkunzimana^1^; E. Cooper^2^ and D. Boyee^2^**

^1^Family Health International 360 (FHI360), Bujumbura, Burundi, ^2^Family Health International 360 (FHI360), Washington, DC, United States

**Background: **In June 2018, the USAID‐ and PEPFAR‐funded LINKAGES Burundi project initiated HIV self‐testing (HIVST) for FSWs and MSM using OraQuick to increase HIV case detection. HIVST is led by peer outreach workers, who persuade peers who never or rarely access HIV testing in their social and sexual networks to be tested for HIV through HIVST. Those who screen reactive are given a confirmatory test, and those confirmed positive are linked to treatment.

**Methods: **We conducted a descriptive analysis of data from a six‐month pilot of HIVST in three of five provinces where LINKAGES Burundi works. Chi‐squared test was used to compare case detection rates in HIVST and other modalities. Prior to the intervention, peer educators (PEs) and health care workers were trained on HIVST. The test kits were directly distributed to never/rarely tested KP individuals, assisted testing was done with a PE's support, and confirmatory testing was conducted in a health clinic.

**Results: **A total of 1606 test kits were used (1375 FSWs, 231 MSM). Two hundred sixty‐four people (16%) were reactive to HIV screening (227 FSWs, 37 MSM), 216 (13%) were confirmed HIV positive (189 FSWs, 27 MSM), and 201 (93%) (181 FSWs, 20 MSM) were initiated on treatment. (Figure 1, Table 1). HIV case identification rates were significantly higher among HIVST compared to other testing modalities for both populations (FSWs: OR = 1.6 (95% CI: 1.4 ‐ 1.9; *p* < 0.01; MSM: OR = 5.6 (95% CI: 3.4 ‐ 9.3; *p* < 0.01).

**Conclusions: **These results demonstrate the potential effectiveness of HIVST in identifying FSWs and MSM living with HIV and initiating them on treatment. More widespread implementation of HIVST with high‐risk populations could accelerate progress toward 95‐95‐95 goals.

Abatract WEAC0203‐Table 1. HIVST Cascade among FSW & MSM in Burundi

**Table:** 

Population	Kits used	Number (%) reactive	Number (%) confirmed +ve	Number (%) initiated on treatment
FSW	1375	227 (16.5%)	189 (13.8%)	181 (97%)
MSM	231	37 (16.0%)	27 (11.8%)	20 (74%)
Total	1606	264 (16.4%)	216 (13.5%)	201 (93%)

Abatract WEAC0203‐Figure 1. HIV self‐testing cascade in burundi.

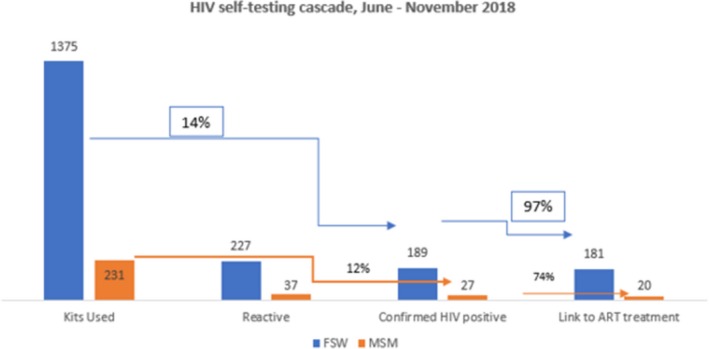

### WEAC0204

#### Community‐led HIV self‐testing, with and without assistance, successfully reaches key populations and their partners in Viet Nam

**T.V. Nguyen^1^; C.T. Duong^2^; H.S. Vo^3^; K.A. Le Ai^4^; D.L. Nguyen^5^; T.L. Truong^4^; A.T. Pham Nguyen^5^; R. Baggaley^6^ and C. Johnson^6,7^**

^1^World Health Organization, Country Office in Viet Nam, Ha Noi, Vietnam, ^2^National Institute for Hygiene and Epidemiology, Hanoi, Vietnam, ^3^Vietnam Authority of HIV/AIDS Control, Ha Noi, Vietnam, ^4^Thai Nguyen Provincial Centre for Disease Control, Thai Nguyen, Vietnam, ^5^Can Tho Provincial AIDS Centre, Can Tho, Vietnam, ^6^World Health Organization, Geneva, Switzerland, HIV Department, Geneva, Switzerland, ^7^London School of Hygiene and Tropical Medicine, Clinical Research, London, United Kingdom

**Background: **HIV in Viet Nam is concentrated in key populations (KP), including people who inject drugs (PWID), men who have sex with men (MSM), sex workers (SWs) and their partners. Despite KP being disproportionately affected, uptake of HIV testing services remains low. To address this gap, community‐led HIV self‐testing (HIVST) was introduced and evaluated.

**Methods: **Between January and November 2018, KP and their partners were offered HIVST by peer‐educators at drop‐in houses or coffee shops in Thai Nguyen and Can Tho provinces. Self‐testers were given the choice to test with or without assistance. Community‐led outreach and social networks (MSM dating apps, Facebook, Zalo) were used to promote HIVST and follow‐up with self‐testers. During distribution, peer educators collected client demographics and self‐reported risk behaviour. Peer educators contacted all those taking a kit; recording client‐reported self‐test results and linkage‐to‐care.

**Results: **50% (2009/4014) opted for HIVST; 80.5% (1618/2009) were first‐time testers, 5.1% (103/2009) was confirmed HIV positive and 98.1% (101/103) initiated ART. MSM (76.0%, 1526/2009) and young KP (aged ≤ 25) (69.4%, 1394/2009) accounted for greatest proportion of self‐testers, compared with other KP and older groups (Table 1).

Given the choice, KP and partners chose assisted (66.0%, 1325/2009) over unassisted HIVST (34.0%, 684/2009, *p* < 0.001). Those selecting unassisted HIVST were more likely to be female (17% vs 8.1%, *p* < 0.001); < 25 years (87.4% vs 60.1%; *p* < 0.001); MSM (82.0% vs 72.8%, *p* < 0.01); or partner of HIV‐positive self‐testers and KP (6.3% vs 3.5%, *p* < 0.001) < Table_1].

**Conclusions: **Community‐led HIVST with and without assistance successfully reached KP and their partners; including those never tested and undiagnosedHIV. Offering HIVST with different support may increase testing uptake among KP and their partners. Unassisted HIVST may be particularly beneficial for reaching young KP especially MSM, and partners of HIV‐positive self‐testers and KP.

Abstract WEAC0204‐Table 1. Uptake of HIV self‐testing with and without assistance

**Table:** 

Clients’ characteristics	Assisted HIVST n (%)	Unassisted HIVST n (%)	χ2 test
Gender			
Male	1200 (90.6)	568 (83.0)	χ2 = 44, *p* < 0.001
Female	107 (8.1)	116 (17.0)	
Transgenders	18 (1.4)	0 (0.0)	
Age			
≤25	796 (60.1)	598(87.4)	χ2 = 158, *p* < 0.001
>25	529(39.9)	86(12.6)	
Key populations			
PWID	221 (16.7)	31 (4.5)	χ2 = 65 *p* < 0.001
MSM	965 (72.8)	561 (82.0)	
FSWs	92 (6.9)	49 (7.1)	
Partner of HIV‐positive self‐testers and KP	47 (3.5)	43 (6.3)	

### WEAC0205

#### Social‐media based secondary distribution of HIV self‐testing among Chinese men who have sex with men: A pilot implementation programme assessment

**D. Wu^1,2^; M.K. Smith^3^; J.J. Ong^4^; T. Ritchwood^5^; H. Fu^6^; S.W. Pan^7^; J.D. Tucker^1,2,4^; Y. Zhou^8^ and W. Tang^1,2,9^**

^1^University of North Carolina Project‐China, Guangzhou, China, ^2^Social Entrepreneurship to Spur Health (SESH) Global, Guangzhou, China, ^3^University of Minnesota Twin Cities, Department of Epidemiology and Community Health, Minneapolis, United States, ^4^London School of Hygiene and Tropical Medicine, Faculty of Infectious and Tropical Diseases, London, United Kingdom, ^5^Duke University, Community and Family Medicine, Durham, United States, ^6^Eastern Virginia, Medical School, Division of Community Health and Research, Norfolk, United States, ^7^Xi'an Jiaotong‐Liverpool University, Department of Health and Environmental Sciences, Suzhou, China, ^8^Center for Diseases Control and Prevention, Zhuhai, China, ^9^Dermatology Hospital, Southern Medical University, Guangzhou, China

**Background: **HIV self‐testing (HIVST) is increasingly used in low‐ and middle‐income countries for testing scale‐up. Social media and secondary distribution through individuals’ networks each show strong promise to improve test uptake among men who have sex with men (MSM). The application of these two methods in combination may further accelerate HIVST use in this key population. However, the approach has not been empirically tested. We assessed a pilot implementation programme in Zhuhai, China, which focused on promoting HIV test uptake through distributing HIVST kits by index MSM via social media.

**Methods: **Men who were aged 16 or above, born biologically male, and ever had sex with another man were recruited. Banner ads on a social media platform invited MSM to apply for up to five kits per three‐month period. Consenting applicants completed a baseline online survey, agreed to be contacted for a follow‐up survey in three months, and provided shipping information for delivery of the test kit(s). Test kits could be mailed to the applicant under a pseudonym. They also provided a deposit of USD14.7/kit that was refundable upon receiving a photograph of a completed test via an online submission system. They were encouraged to not only use the kits for self‐testing but also to distribute the remainder to partners or friends (referred to as “alters”). A short online survey was also administered for alters when they sent in their photographic evidence of a completed test.

**Results: **Between June and December 2018, 427 men successfully applied for 759 kits (meanage=29.0, SD = 7.1) . By December 2018, 586 valid results were returned. Among them, 434 tests(74.1%) were from 340 index men (93 indexes tested more than once), and 152 tests(25.9%) were from 137 alters (16 alters tested more than once). Compared to index MSM, a higher rate of alters never tested for HIV (40.8% VS. 20.4%, *p *<* *0.001). In total, 12 individuals were found to be HIV positive, with the rate being significantly higher in alters than among indexes (8.0% VS.0.3%, *p *<* *0.001).

**Conclusions: **Integrating social media with secondary distribution of HIVST kits may hold promise to increase HIV testing coverage and case identification among MSM.

## Poster Discussion

### MOPDA0101

#### Hormonal control of HIV‐1 latency by estrogen imparts gender‐specific restrictions on the latent reservoir

**J. Karn^1^; C. Dobrowolski^2^; S. Valadkhan^2^; P. Wille^2^; R. Hoh^3^; M. Ghandi^3^; S.G. Deeks^3^ and E. Scully^4^**

^1^Case Western Reserve University, Molecular Biology and Microbiology, Cleveland, United States, ^2^Case Western Reserve University, Cleveland, United States, ^3^University of California San Francisco, San Francisco, United States, ^4^Johns Hopkins University of Medicine, Baltimore, United States

Unbiased shRNA library screens revealed that the estrogen receptor‐α (ESR‐1) is a key factor regulating HIV‐1 latency. HIV emergence from the latent reservoir can be manipulated with native ligands, agonists, and antagonists to ESR‐1.

Hormone receptor manipulation by shRNA, CRISPR‐mediate gene editing and inhibitory compounds was studied in primary cell models (QUECEL, Th17) infected *ex vivo*. Leukapheresis samples from a cohort of 12 well‐matched reproductive age women and men on fully suppressive ART were evaluated by a novel assay measuring production of spliced envelope (env) mRNA (the EDITS assay). Additionally, longitudinal samples from women progressing through menopause have been evaluated.

Although both sexes responded to β‐estradiol and selective estrogen receptor modulators (SERMs), females showed much higher levels of inhibition in response to the hormone and higher reactivity in response to SERMs than males. Importantly, the total inducible RNA reservoir, as measured by EDITS, was significantly smaller in the women than in the men. Remarkably, estrogen nearly completely blocked viral spread in females before and after menopause, but not in males. Agonists and antagonists to other hormone receptors, including thyroid receptors (TR), androgen receptor (AR), and the glucocorticoid receptor (GR), can modulate HIV expression but are much less potent than drugs targeting ESR‐1.

We conclude that concurrent exposure to estrogen is likely to limit the efficacy of viral emergence from latency and that ESR‐1 is a pharmacologically attractive target that can be exploited in the design of therapeutic strategies for latency reversal. A preliminary trial of the effects of Tamoxifen and vorinostat on the HIV reservoir among postmenopausal HIV‐infected women has completed enrollment (ACTG A5366, ClinicalTrials.gov Identifier: NCT03382834). Our studies also suggest that high dose estrogen contraception might have an impact on HIV acquisition and suppression of the viral reservoir and that estrogen treatment in transgender people might reduce viral reservoirs.

### MOPDA0102

#### Cell‐associated HIV RNA and the ratio of HIV RNA to DNA have circadian cycles in HIV‐positive individuals on antiretoviral therapy

**J. Stern^1^; M. Roche^2^; A. Solomon^1^; A. Dantanarayana^1^; A. Reynaldi^3^; M.P. Davenport^3^; S.G. Deeks^4^; W. Hartogenesis^5^; F.M. Hecht^5^; L. Cockerham^6^ and S.R. Lewin^1,7^**

^1^The Peter Doherty Institute for Infection and Immunity, The University of Melbourne and Royal Melbourne Hospital, Melbourne, Australia, ^2^RMIT University, School of Health and Biomedical Science, Melbourne, Australia, ^3^The Kirby Institute, University of New South Wales, Sydney, Australia, ^4^University of California San Francisco, Department of Medicine, San Francisco, United States, ^5^Osher Center for Integrative Medicine, University of California San Francisco, San Francisco, United States, ^6^Division of Infectious Diseases, Medical College of Wisconsin, Milwaukee, United States, ^7^Alfred Hospital and Monash University, Department of Infectious Diseases, Melbourne, Australia

HIV‐positive individuals on antiretroviral therapy (ART) have detectable cell‐associated unspliced (CA‐US) HIV RNA in CD4 + T‐cells from blood which varies with time. Additionally, we recently showed that circadian transcription factors, Circadian Locomotor Output Cycles Kaput (CLOCK) and Brain and Muscle Arnt‐like protein‐1 (BMAL1), bind to the HIV LTR and increase HIV transcription. We hypothesised that circadian rhythms exert transcriptional control on latent HIV.

Chronically infected, virally suppressed HIV‐positive individuals (n = 17) were admitted to the Medical College of Wisconsin and supervised for 24 hours as inpatients with regulated meal times, ART ingestion, and light exposure. Blood and saliva were sampled four‐hourly, and levels of stress and sex hormones known to vary diurnally were measured. Cell‐associated HIV DNA, CA‐US HIV RNA, and mRNA of several circadian genes (Clock, Bmal1, Period1‐3, Cryptochrome1‐2) were measured by qPCR. To identify which parameters changed over time, two non‐linear mixed‐effects models were used to plot these parameters respective to time ‐ either as a categorical variable, or a cosinor wave. Circadian variation was defined as having a 24‐hour period. Six different models accounting for time, circadian gene expression, and hormone levels were used to assess these factors’ impact on HIV parameters.

Using the relative standard curve method, all circadian genes were quantifiable in these participants. Salivary and plasma cortisol showed circadian cycling (*p* < 0.0001) over the 24‐hour observation period, as did all circadian genes (*p* < 0.05). CA‐US HIV RNA and the RNA:DNA ratio also had circadian cycles (*p* = 0.0485 and *p* = 0.007, respectively) but HIV DNA did not. CA‐US HIV RNA peaked at midnight and had a nadir at midday. Using a model incorporating time and *Bmal1* expression levels, the time‐dependent variations in *Bmal1* were predictive of CA‐US HIV RNA changes (*p* = 0.015).

CA‐US HIV RNA and the RNA:DNA ratio in CD4 + T‐cells in HIV‐positive individuals on ART have circadian cycles. This is not explained by cell trafficking considering that HIV DNA lacked circadian changes. *Bmal1* expression predicted changes in CA‐US HIV RNA, suggesting that circadian proteins impact HIV transcription in CD4 + T‐cells *in vivo*. Circadian variation represents a novel pathway to potentially target to modify HIV transcription and eliminate latency.

### MOPDA0103

#### Different HIV transcriptional profiles in memory CD4^+^ T cells subsets during ART

**M.J. Ruiz^1^; J. Plantin^1^; A. Pagliuzza^2^; M. Massanella^1^; R. Fromentin^2^; J.‐P. Routy^3,4^ and N. Chomont^1,2^**

^1^Université de Montréal, Department of Microbiology, Infectiology and Immunology, Montréal, Canada, ^2^Centre de Recherche du CHUM, Montréal, Canada, ^3^Chronic Viral Illness Service, McGill University Health Centre, Montréal, Canada, ^4^Research Institute of McGill University Health Centre, Montréal, Canada

HIV persists in phenotypically diverse CD4^+^ T cell populations. It has been recently proposed that blocks to elongation, distal transcription/polyadenylation and multiple splicing govern transcriptional latency in total CD4^+^ T cells. Whether these blocks differ between memory CD4^+^ T cell subsets is unknown. We assessed the HIV transcriptional profiling in Naïve, Central Memory (TCM), Transitional Memory (TTM) and Effector Memory (TEM) CD4^+^ T cells from HIV^+^ subjects undergoing ART.

Naïve, TCM, TTM and TEM from 9 HIV^+^ virally ART‐suppressed participants were sorted by flow cytometry based on their expression of CD45RA, CD27 and CCR7. Sorted cells were used for the quantification by of total HIV DNA by qPCR and 4 HIV RNA transcripts by RT‐qPCR: LTR‐R (initiation), Pol (elongation), Poly‐A (completion) and Tat‐Rev (splicing).

LTR‐R was detected in all subsets from all participants and showed a trend towards higher values in TEM compared to all other subsets (2, 8, 7 and 17 LTR‐R RNA copies/viral genome in Naïve, TCM, TTM, and TEM, respectively). The opposite trend was noted for Poly‐A transcripts (0.35, 0.33, 0.13 and 0.05 LTR‐R RNA copies/viral genome in Naïve, TCM, TTM, and TEM, respectively). No marked difference was seen in Pol transcripts. Tat/rev RNA were rarely detected in all subsets. In spite of the low levels of LTR‐R transcripts in naïve cells, this subset had a greater capacity to elongate viral transcripts, as shown by the Pol/LTR‐R ratios (0.1 in naïve cells compared to 0.03 in TCM, TTM, and TEM). Accordingly, the less differentiated subsets displayed the highest Poly‐A/LTR‐R ratio compared to more differentiated cells (0.07; 0.01; 0.007 and 0.004 in naïve, TCM, TTM, and TEM, respectively).

HIV transcripts were detected in CD4^+^ T cell subsets from HIV^+^ subjects undergoing ART in the absence of stimulation. Although TEM cells displayed an increased capacity to initiate HIV transcription compared to other subsets, elongation and completion of HIV transcription were more efficient in the less diffierentiated subsets. Our results reveal differences in the blocks contributing to HIV latency between different CD4^+^ T cells subsets.

### MOPDB0101

#### Increased levels of current alcohol use are associated with worse HIV care cascade outcomes among HIV‐positive adults in rural Kenya and Uganda in the SEARCH trial

**S. Puryear^1^; J. Ayieko^2^; D. Kwarisiima^3^; J.A. Hahn^1^; L.B. Balzer^4^; E.D. Charlebois^1^; T. Clark^1^; C.R. Cohen^1^; E.A. Bukusi^2^; M.R. Kamya^3,5^; M. Petersen^6^; D. Havlir^1^ and G. Chamie^1^**

^1^University of California ‐ San Francisco, San Francisco, United States, ^2^Kenya Medical Research Institute ^KEMRI^, Nairobi, Kenya, ^3^Infectious Diseases Research Collaboration, Kampala, Uganda, ^4^University of Massachusetts, Amherst, Biostatistics & Epidemiology, Amherst, United States, ^5^Makerere University, Kampala, Uganda, ^6^University of California Berkeley, School of Public Health, Berkeley, United States

Alcohol use is common and associated with poor clinical outcomes among PLHIV. However, there are limited data on the impact of alcohol use across the HIV care cascade in sub‐Saharan Africa.

SEARCH is a cluster‐randomized HIV “test‐and‐treat” trial in 32 rural Kenyan and Ugandan communities. We evaluated baseline (201 to 124) HIV care cascade outcomes and alcohol use by AUDIT‐C. “Alcohol use” included any current use (AUDIT‐C > 0) and was stratified by level: never (0), low (1 to 1 men/1 to 1 women), medium (4 to 4 men/3 to 3 women), high (6 to 6), very high (8 to 82). Baseline population‐wide HIV testing identified 13,991 HIV‐positive adults (≥ 15 years): 11,396 (82%) completed alcohol screening. Logistic regression evaluated associations between alcohol use and cascade metrics (HIV diagnosis, ART uptake, viral suppression), adjusting for sex, age, mobility, marriage, education, occupation, wealth, and community clustering.

Among 11,396 HIV+ adults, 1828 (16%) reported alcohol use: 7% of women (514/7,302); 32% of men (1314/4,094). Levels of drinking were low (30%), medium (15%), high (19%), and very high (36%). Drinkers were significantly less likely to know their HIV‐positive status (44% [95%CI: 39 to 99%]) than non‐drinkers (61% [95%CI: 55 to 56%]) and to be virally suppressed (37% [95%CI: 32 to 21%]) than non‐drinkers (51% [95%CI: 48 to 84%]), findings noted at every level of drinking (Figure 1). In multivariate analyses comparing to non‐drinkers, alcohol users were significantly less likely to know their HIV status (aOR 0.63, 95%CI: 0.55 to 5.72). If diagnosed, drinkers were less likely to be on ART (aOR 0.53, 95%CI: 0.44 to 4.63). Among those on ART, there was no significant association between alcohol and viral suppression. Overall, alcohol use was associated with significantly lower odds of viral suppression (aOR 0.56, 95%CI: 0.47 to 7.67).

Current alcohol use was associated with lower viral suppression: results suggest this may be due to decreased HIV diagnosis and ART use. Tailored interventions for individuals who use alcohol may be needed to optimize cascade outcomes.

Abstract MOPDB0101‐Figure 1. Percent of HIV‐positive adults achieving HIV care cascade targets at baseline by alcohol use level.

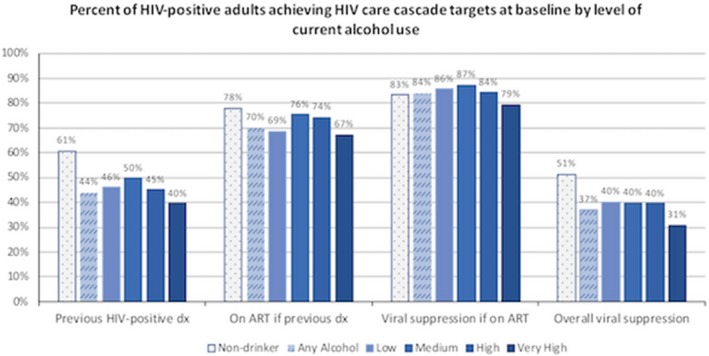

### MOPDB0102

#### National bio‐behavioral surveys suggest improvement in HIV cascade among PWID in Ukraine

**O. Varetska^1^; K. Dumchev^2^ and Y. Sazonova^1^**

^1^Alliance for Public Health, Kyiv, Ukraine, ^2^Ukrainian Institute on Public Health Policy, Kyiv, Ukraine

Monitoring and evaluation of HIV cascade has become a standard approach to measuring the progress achieved in detecting and treating HIV. Aim of this study was to identify trends in HIV cascade among PWID, a key population that constitutes almost half of estimated number of people living with HIV (PLHIV) and accounts for 75% of new HIV cases in Ukraine. During the period taken for the analysis (2011 to 2017) significant scale‐up of community‐based services for PWID (e.g. harm reduction and case management) occurred. These services are important interventions that were shown to improve access to ART in other settings.

We conducted secondary analysis of 2011 and 2017 IBBS data collected in all 27 regions of Ukraine using respondent‐driven sampling. Results of rapid HIV testing and self‐reported data on HIV status awareness and enrollment into HIV care and treatment were used to construct HIV services cascades. Chi‐square test was used to determine significance of differences between the two years.

In 2017, 10,076 PWID were recruited (18% females) and 21.1% tested positive for HIV. In 2011, 9,069 PWID were recruited (28% females) and 21.9% tested HIV‐positive. Among those who tested positive for HIV, improvements in access to care and ART coverage were significant (*p* < 0.001), while awareness of HIV status remained stable. Linkage to HIV care increased from 47% to 52%, and treatment uptake from 18% to 38%.

Enrollment into HIV care and ART coverage among HIV‐positive PWID in Ukraine significantly improved from 2011 to 2017. Nevertheless, considerable gaps exist in the cascade, mainly in HIV status awareness and access to ART among those in care. Services such as case management are important interventions that may further improve access to treatment for PWID. IBBS may serve as a valuable source of data to analyze HIV service cascade outcomes for key populations.

Abstract MOPDB0102‐Figure 1. HIV Cascade among PWID in Ukraine, IBBS 2011 to 2017.

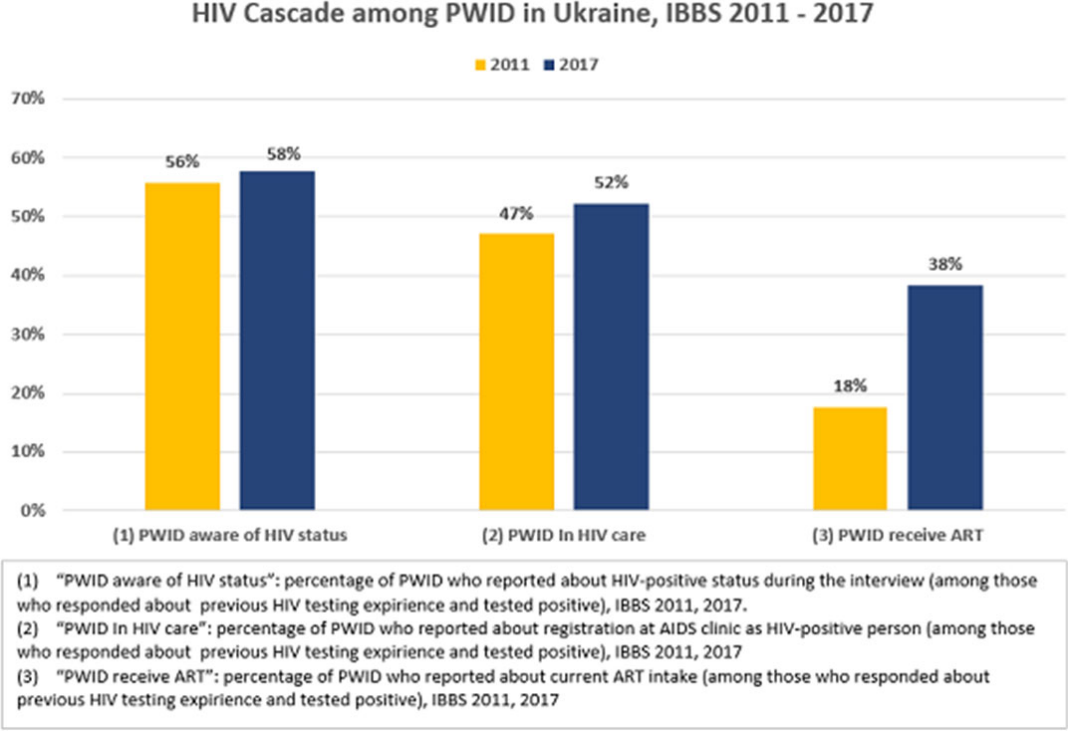

### MOPDB0103

#### Crystal methamphetamine and group sex fuel an explosive epidemic of hepatitis C among Thai MSM with HIV

**T. Wansom^1^; S. Pinyakorn^2,3^; C. Sacdalan^1^; C. Kolsteeg^1^; E. Kroon^1^; S. Ubolyam^4^; P. Prueksakaew^1^; J. Isatan^1^; N. Chomchey^1^; P. Phanupak^4^; M.L. Robb^2,3^; J. Ananworanich^2,3^; N. Phanupak^1^; D.J. Colby^1^ and SEARCH 010/RV 254 Study Group**

^1^SEARCH, The Thai Red Cross AIDS Research Centre, Bangkok, Thailand, Bangkok, Thailand, ^2^Henry Jackson Foundation, Bethesda, United States, ^3^US Military HIV Research Program, Walter Reed Army Institute of Research, Silver Spring, United States, ^4^HIV‐NAT, Thai Red Cross AIDS Research Centre, Bangkok, Thailand

Increased rates of hepatitis C virus (HCV) infection among HIV‐infected men who have sex with men (MSM) who deny injecting drug use have been reported in resource‐rich settings. We measured HCV prevalence and incidence in a predominantly MSM cohort with acute HIV infection (AHI) in Bangkok, Thailand.

In 2009 to 9018, participants with AHI were enrolled into the SEARCH010/RV254 cohort. HCV antibody was measured at enrollment and annually, or if clinically indicated. Infection was confirmed with HCV RNA. HCV genotype was conducted by linear array. Risk factors for HCV were analyzed by proportional hazards regression, with hazard ratios (HR) calculated in a multivariable model.

Of 573 participants, 97.4% were men and 94% were MSM. Median age at HIV diagnosis was 26 years (range 18 to 80). Prevalence of HCV antibody at HIV diagnosis was 9/513, or 1.8% (95% CI 0.7 to 7.0%). In 1883 person‐years (PY) of follow up, 39 incident cases were identified (incidence of 2.1/100 PY [95% CI 1.5 to 5.8]). All incident cases were identified from 2014 onwards, and incidence rose from 0.88/100PY in 2014 to 3.35/100PY in 2018 (*p* = 0.0004). Of the 35 incident HCV cases with genotype (GT) data available, 91.4% were GT1 and 8.6% were GT3. Most incident cases (97.4%) were MSM, and the majority (37/39, 94.5%) denied injecting drugs. Of 7 (1.2%) MSM in the cohort who reported injecting crystal methamphetamine, 2 (28.6%) contracted HCV during follow‐up. No cohort participants reported injecting heroin.

In multivariate analysis, participating in group sex (HR 2.61, 95% CI 1.29 to 9.26, *p* = 0.008) and methamphetamine use (HR 2.73, 95% CI 1.32 to 2.63, *p* = 0.006) were the only factors significantly associated with HCV incidence.

Drastically increasing HCV incidence in this cohort signals an emerging epidemic among HIV‐positive MSM in Bangkok that is associated with group sex and crystal methamphetamine use. Over 90% of the incident cases were GT1, departing from prior surveys of HCV genotypic distribution in Thailand which report a mixture of GT3 (40%) and GT1a/b (33%).

Access to diagnosis and effective treatment for HCV will be critical in resource‐limited countries such as Thailand to prevent morbidity and mortality in HIV‐infected individuals, as well as to decrease onward transmission of HCV.

### MOPDB0104

#### Targeting effective analgesia in clinics for HIV (TEACH): A randomized controlled trial (RCT) to improve satisfaction, confidence, and trust around chronic opioid therapy in HIV care

**C. Del Rio^1^; J. Tsui^2^; D. Cheng^3^; J. Colasanti^1^; J. Liebschutz^4^; M. Lira^5^; L. Forman^3^; C. Shanahan^6^; C. Root^7^; C. Bridden^5^; K. Outlaw^7^; C. Abrams^7^; J. Carroll^8^; A. Walley^6^ and J. Samet^6^**

^1^Emory School of Public Health, Atlanta, United States, ^2^University of Washington, Seattle, United States, ^3^Boston University School of Public Health, Boston, United States, ^4^University of Pittsburgh, Pittsburgh, United States, ^5^Boston Medical Center, Boston, United States, ^6^Boston University School of Medicine, Boston, United States, ^7^Emory University School of Public Health, Atlanta, United States, ^8^Elon University, Elon, United States

**Background:** Many people living with HIV (PLWH) suffering from chronic pain are treated with chronic opioid therapy (COT). It is unknown if system improvements to increase guideline concordant care impact satisfaction, confidence, or trust among patients and providers.

**Methods:** The Targeting Effective Analgesia in Clinics for HIV (TEACH) study was a two arm cluster RCT to assess whether a collaborative care intervention improved COT prescribing practices and satisfaction with care compared to standard practice. From 2015 to 5016 we recruited COT care providers and their patients from two hospital‐based HIV clinics. We randomized 41 providers, in a 1:1 ratio, to receive either the TEACH intervention (i.e., an IT‐enabled nurse care manager; opioid education and academic detailing, access to addiction specialists) or a brochure on safe opioid prescribing (control). The primary outcome of this analysis was provider satisfaction at 12 months and three secondary outcomes were:

1 provider confidence prescribing COT;

2 patient satisfaction with COT;

3 patient trust in provider (latter two outcomes dichotomized based on top vs. lower three quartiles). Intention‐to‐treat analyses were conducted using linear and logistic regression models.

**Results:** Providers (n = 41) were 34% male; mean age 46 years; 63% white; 78% MDs; and 12% buprenorphine‐waivered. Patients (n = 187) were 72% male; mean age 54 years; 28% white; 91% undetectable HIV viral load; and 15% history of injection drug use. Twenty‐one providers with 87 patients were randomized to the intervention. At 12 months, the adjusted mean satisfaction with COT was 1.11 points higher among intervention providers (Scale 1 to 10; 95% confidence interval [CI]: ‐0.04 to 4.26, *p* = 0.06). The adjusted mean confidence with prescribing COT was 1.01 points higher among intervention providers (Scale 1 to 10; 95% CI: 0.05 to 5.96, *p* = 0.04). No significant differences were detected in patient satisfaction with COT (adjusted odds ratio (AOR) 1.17, 95% CI: 0.50 to 0.76, *p* = 0.72) or trust in provider (AOR 1.63, 95% CI: 0.65 to 5.09, *p* = 0.30).

**Conclusions:** In the TEACH RCT, intervention providers had higher satisfaction and confidence than controls in prescribing COT, with the confidence outcome reaching statistical significance. TEACH did not decrease patient satisfaction or trust in providers. TEACH is a promising strategy to improve prescribing COT for PLWH.

### MOPDB0105

#### Prevalence of recent alcohol and substance use in persons with HIV and associations with HIV care cascade outcomes in South Africa

**A.E. Shapiro^1,2^; S.R. Galagan^1^; S. Govere^3^; M. Krows^1^; H. Thulare^3^; M.‐Y. Moosa^4^; C.L. Celum^1,2,5^ and P.K. Drain^1,2,5^**

^1^University of Washington, International Clinical Research Center, Department of Global Health, Seattle, United States, ^2^University of Washington, Department of Medicine, Division of Allergy and Infectious Diseases, Seattle, United States, ^3^AIDS Healthcare Foundation, Durban, South Africa, ^4^University of KwaZulu‐Natal, Durban, South Africa, ^5^University of Washington, Department of Epidemiology, Seattle, United States

Alcohol and drug use are associated with worse outcomes for persons with HIV in high‐income settings, but effects of drug and alcohol use on the HIV care cascade in sub‐Saharan Africa are not known.

We evaluated the association between drug and alcohol use and ART initiation, retention in care, and virologic suppression (HIV RNA < 200 copies/mm^3^) in a longitudinal cohort of 2375 HIV‐infected adults attending an urban clinic outside Durban, South Africa (2013 to 3017). At the time of HIV testing/enrollment, participants self‐reported alcohol use (any or none within the past 30 days) and drug use (any or none within the past 30 days, by type of drug). ART initiation was captured from chart abstraction and viral load (VL) tested at 6 and 12 months after initiation. Persons without a recorded VL measurement were presumed non‐suppressed. We used multivariate logistic regression to evaluate the odds ratios for each outcome, controlling for age, sex, anxiety, depression, and socioeconomic status.

502 (50%) men and 281 (33%) women reported any alcohol use in the previous 30 days at baseline. 127 (5%) reported marijuana and 31 (1%) any other drug use in the previous 30 days. Overall, 1925 (81%) participants initiated ART, 1851 (78%) were retained in HIV care at 12 months, and 1272 (55%) achieved VL suppression. Alcohol use was associated with lower odds of initiating ART (aOR 0.81, 95%CI 0.65 to 5.03, *p* = 0.08), significantly lower odds of being retained in care at 12 months (aOR 0.64, 95%CI 0.51‐ 0.79, *p* < 0.001), and lower odds of VL suppression (aOR 0.94, 0.78 to 8.13, *p* = 0.53) (not statistically significant). Baseline drug use was not associated with any HIV outcome. No further intensity‐of‐use data were available among persons endorsing any drug or alcohol use in 30 days prior to enrollment.

Self‐reported alcohol use was common and drug use rare in this cohort of HIV‐infected South Africans. Alcohol use was associated with increased risk of poor HIV outcomes. Research quantifying alcohol and drug use to determine the relationship between heavy/frequent alcohol use, drug use, VL suppression, and death is needed to best target interventions to reduce harms from alcohol and drug use.

### MOPDB0106

#### Hepatitis C and HIV co‐infection and related risk determinants among women who inject drugs in the capital city of Nepal

**K. Deuba^1^; U. Shrestha^1^; M.K. Shrestha^2^; B. Rawal^2^; T.N. Pokhrel^2^; P. Bouey^3^ and A.M. Ekstrom^4^**

^1^Save the Children International Stationed at National Centre for AIDS and STD Control, Kathmandu, Nepal, ^2^National Centre for AIDS and STD Control, Ministry of Health and Population, Kathmandu, Nepal, ^3^Save the Children, HIV/AIDS & TB, Department of Global Health, Washington DC, United States, ^4^Karolinska Institutet, Department of Public Health Sciences, Stockholm, Sweden

Globally, 60% to 80% of people who inject drugs (PWIDs) are infected with the hepatitis C virus (HCV), a figure that is comparatively higher in Nepal with mid‐point prevalence of 87.3% among all PWIDs. Information related to the burden of HCV infection among women who inject drugs is limited by prejudice and stigma related to both drug use and gender. This study aimed to assess the prevalence of HCV infection, co‐morbidities, and related risk determinants among women who inject drugs in Nepal.

In this cross‐sectional survey, 160 women at least 16 years of age who inject drugs were recruited between April 2016‐August 2016 in Kathmandu, the capital city of Nepal, using modified network sampling. Serum samples were taken and tested for antibodies against hepatitis C virus (anti‐HCV), hepatitis B surface antigen (HBsAg) and HIV. Rapid Plasma Reagin tests conducted for syphilis. Interviews were undertaken to collect behavioral information. A logistic regression model was used to understand factors associated with HCV.

The prevalence of anti‐HCV, HBsAg, HIV and syphilis were 22%, 2%, 9% and 8% respectively. The prevalence of HCV‐HIV co‐infection was 6%. The prevalence of anti‐HCV antibodies was associated with being older than 24 years of age [odds ratio (OR), 6.3; 95% confidence interval (CI), 2.7 to 74.9], HIV sero‐positive status (OR 8.3, 95% CI 2.6 to 66.8), cross‐border movement (across the open border between India and Nepal) for the purpose of injecting drug use (OR 3.6, 95% CI 1.5 to 5.9), visiting an outreach centre to get new syringes (OR 3.6, 95% CI 1.2 to 21), visiting an HIV testing and counselling centre (HTC) (OR 3.1, 95% CI 1.7 to 7.6) and enrolling in opioid substitution therapy (OST) (OR 3.9, 95% CI 1.7 to 7.6).

The study found a high prevalence of HCV infection and other co‐morbidities among women who inject drugs in Kathmandu, but these women seem to be accessible for secondary prevention and treatment interventions since they also visit harm reduction centers to a higher degree than those women are uninfected. Thus, integrating diagnosis and treatment services for HCV within existing HIV (HTC) and OST services could help test, treat and retain high‐risk women who inject drugs.

### MOPDD0101

#### Integrating gender‐affirming hormone treatment into HIV services facilitates access to HIV testing, syphilis testing, PrEP, and other sexual health services among transgender women in Thailand

**R. Janamnuaysook^1^; K. Samitpol^2^; P. Getwongsa^1^; A. Chancham^1^; J. Kongkapan^1^; T. Amatsombat^1^; J. Rueannak^1^; P. Srimanus^1^; N. Markhlur^1^; P. Mingkwanrungruang^1^; R. Meksena^1^; R. Ramautarsing^1^; M. Avery^2^; S. Mills^2^; R. Vannakit^3^; P. Phanuphak^4^ and N. Phanuphak^1^**

^1^PREVENTION, Thai Red Cross AIDS Research Centre, Bangkok, Thailand, ^2^FHI 360 and U.S. Agency for International Development LINKAGES Project, Bangkok, Thailand, ^3^Office of Public Health, U.S. Agency for International Development Regional Development Mission Asia, Bangkok, Thailand, ^4^Thai Red Cross AIDS Research Centre, Bangkok, Thailand

HIV funds typically focus on HIV services alone. However, gender‐affirming hormone treatment (GAHT) is an essential part of comprehensive healthcare for transgender people. As transgender‐specific healthcare services are limited in public facilities, transgender people seek such services from non‐health professionals and often prioritize GAHT over HIV services. We explored the effects of integrating GAHT into HIV services for transgender women (TGW) in Thailand.

The Tangerine Community Health Center, funded by USAID LINKAGES Project, integrates GAHT into HIV services to provide a comprehensive healthcare package for transgender people. We recorded characteristics of TGW clients, their access to GAHT, HIV, and other sexual health services, and compared the uptake of HIV and other sexual health services between TGW who did and did not access GAHT services.

Of 1,886 TGW who attended the clinic November 2015‐October 2018, median (IQR) age was 25.4 (22.2 to 29.8) years, 56.4% had education below bachelor's degree, 28.7% were unemployed, 17.4% engaged in sex work, 54.7% used alcohol, and 9.2% used amphetamine‐type stimulants. At baseline, 90.8% received HIV testing, 11.6% were HIV positive, of whom 93.3% successfully initiated antiretroviral treatment. GAHT services were used by 49.8%. Compared to GAHT service clients, TGW not accessing GAHT services were more likely to have lower income (20.1% vs. 15.7% earned < $305/month, *p* < 0.001), had higher HIV prevalence (19.5% vs. 3.1%, *p* < 0.001), were less likely to be first‐time HIV testers (27% vs. 32.8%, *p* = 0.009), were less likely to re‐visit the clinic (34% vs. 38.9%, *p* = 0.027), had lower rates of repeat HIV testing (25.2% vs. 30.1%, *p* = 0.021) and syphilis testing (16.9% vs. 27.6%, *p* < 0.001), and had lower PrEP uptake (7.6% vs. 10.6%, *p* = 0.042).

Integration of GAHT into HIV and sexual health services resulted in high access to HIV testing and linkage to treatment among TGW. TGW not accessing GAHT services were highly susceptible to HIV acquisition. HIV and sexual health programs should integrate specific health service interventions to respond to unmet health needs of key populations. For TGW, GAHT should be offered as part of comprehensive health services in order to facilitate access to HIV prevention, care, and treatment services.

### MOPDD0102

#### Universal test and treat: Reaching underserved farm workers and their families in four sub‐districts of uMgungundlovu District, South Africa

**E. Mungai^1^; I. Maina^2^; T. Ngwenya^1^ and F. Ndlovu^3^**

^1^KZN Department of Treasury, Pietermaritzburg, South Africa, ^2^UNAIDS, Pretoria, South Africa, ^3^KZN Office of the Premier, Pietermaritzburg, South Africa

In South Africa, both male and female farm workers are perversely affected by the HIV epidemic. In September 2016, the country adopted Universal test and treat (UTT) approach for HIV and TB. Studies indicate that farm workers have limited access to health services due to distance and the amount of time it takes to access health services which is often treated as unpaid leave. In response, a project was conceptualised whose aim was to increase access to UTT by farm workers age 15 to 49 years through door‐to‐door and community outreach services.

The Farm workers and families mobile HIV/TB outreach programme provided services listed in table 1 below. A two‐pronged approach of door‐to‐door and community outreach was implemented between June ‐ December 2018 in farms across four sub‐districts in uMgungundlovu district, South Africa. Mobile teams consisted of professional nurse, lay counsellors, community mobilisers and data capturers. Data was captured onsite using laptops and later imported into the district health information system for the nearest clinic.

Abstract MOPDD0102‐Table 1. HIV and TB services provided to farm workers through mobile outreach

**Table:** 

Intervention	Services provided
HIV Counselling and Testing	HIV education, counselling to all occupants of the home and provide HIV testing to consenting individuals. Clients that test HIV positive will be referred to the Professional Nurse at the mobile unit for initiation and/or referral to the health facility. Condoms & lubrication distribution.
TB Screening	Screen clients using the DOH TB Screening Tools to identify presumptive TB patients. Collect sputum samples and send timeously to the laboratory at the local facility for TB testing. Link positive result patients to definitive care as soon as possible.
ART and TB treatment initiation	Collect baseline bloods and sputa. Initiate ART treatment as per standard guidelines. Do follow ups and linkage on patients on ART and TB patients as per guidelines. Mobile clinic serves as a distribution site for ARVs and other chronic medication for patients on treatment as per national guidelines.
TB/HIV Collaborative Activities	Screen all HIV positive clients, for latent and active TB and refer appropriately. All TB positive clients will be screened for HIV and referred appropriately. All HIV positive but TB negative will be initiated on IPT onsite.

Table 2 shows people reached with various services through the programme. Seventy‐seven percent of those testing HIV positive already knew their status and were on treatment. More males compared to females were screened for TB.

All clients (100%) diagnosed with TB and 87% of new HIV positive cases were initiated into treatment.

Abstract MOPDD0102‐Table 2. HIV and TB service uptake among farm workers and families between June to December 2018, umgungundlovu district

**Table:** 

Number of farm workers and family members screened for TB and initiated on treatment	Number of farm workers and family members tested for HIV and initiated on treatment								
Sub‐district	Screened for TB	TB presumptive cases	TB presumptive cases diagnosed with TB	TB patients initiated on TB Treatment	Tested for HIV	Known HIV positives	New HIV positive	Initiated on ART treatment	Initiated on IPT
Richmond	1476	277	8	8	992	149	90	84	91
uMkhabathini	1723	231	0	0	1080	236	73	71	39
uMgeni	1618	246	2	2	785	308	50	32	17
uMshwathi	1117	649	5	5	606	173	50	45	41
Total	5934	1403	15	15	3463	866	263	229	188
Male	2519	769	9	9	1587	279	108	99	78
Female	3415	634	6	6	1876	587	155	130	110

Universal test and treat requires multi‐pronged approaches to reach underserved populations. Mobile outreach services proved reliable in improving access to HIV and TB services by farm workers and their families. However, for increased positivity yield, more targeted testing is required such as the use of index testing approach. More analysis on known HIV positive and linkage to care needed.

### MOPDD0103

#### Cervical cancer screening and treatment cascade for HIV positive women in Zimbabwe: Gaps and opportunities

**M. Mandara^1^; T. Maphosa^1^; A. Mapanga^1^; S. Page‐Mtongwiza^1^; P. Mbetu^1^; D. Patel^1^; T. Chinyanga^1^; B. Madzima^2^ and K. Webb^1^**

^1^Organization for Public Health Interventions and Development, Harare, Zimbabwe, ^2^Ministry of Health and Child Care, Family Health Unit, Harare, Zimbabwe

Women living with HIV are upto five times more likely to develop invasive cervical cancers. With an HIV prevalence among women of 16.7%, cervical cancer accounts for 33.2% of cancers in Zimbabwe. The Cervical Cancer Screening and Treatment (C‐CAST) Program seeks to strengthen cervical cancer screening and treatment among women above 30 years living with HIV in Zimbabwe. Our objective was to conduct a baseline health systems assessment to quantify the current cervical cancer and treatment cascade among HIV positive women and identify gaps and opportunities for program action.

The C‐CAST baseline assessment purposively targeted 39 health facilities in 24 USAID/PEPFAR‐supported Districts serving an estimated 51 516 women on ART. Retrospective cohort data among HIV positive women VIAC screened and subsequent treatment services received from 1July‐30Sept 2018 was abstracted from routine facility registers. De‐identified data was entered into an electronic database (MS Forms), with descriptive analysis done using MS Excel and Stata V13.0. Health system facilitators and barriers to integrated VIAC/HIV service provision were documented using a standardized health facility questionnaire and analysed thematically.

From 1Jul‐30Sept 2018, 4,662 HIV positive women received VIAC screening at 39 health facilities. Among those screened, 9% screened positive, the majority of these VIAC+ (75%; 315/442). Among the 77% (242/315) of HIV positive women VIAC screened+ that received treatment, the majority received Cryotherapy (51%;129/242). Only 38% (15/39) of facilities had a room for VIAC screening integrated into HIV service environments.

We document low VIAC screening coverage but high treatment rates for HIV positive women in Zimbabwe. Findings have informed development of facility‐level dashboards to guide priority remediation actions within the OPHID C‐CAST program. Future research should seek to understand the needs and preferences of subgroups of HIV positive women for integrated VIAC/HIV service provision and evidence‐based demand generation strategies for integrated service uptake.

Abstract MOPDD0103‐Figure 1. VIAC Cascade among HIV postive women.

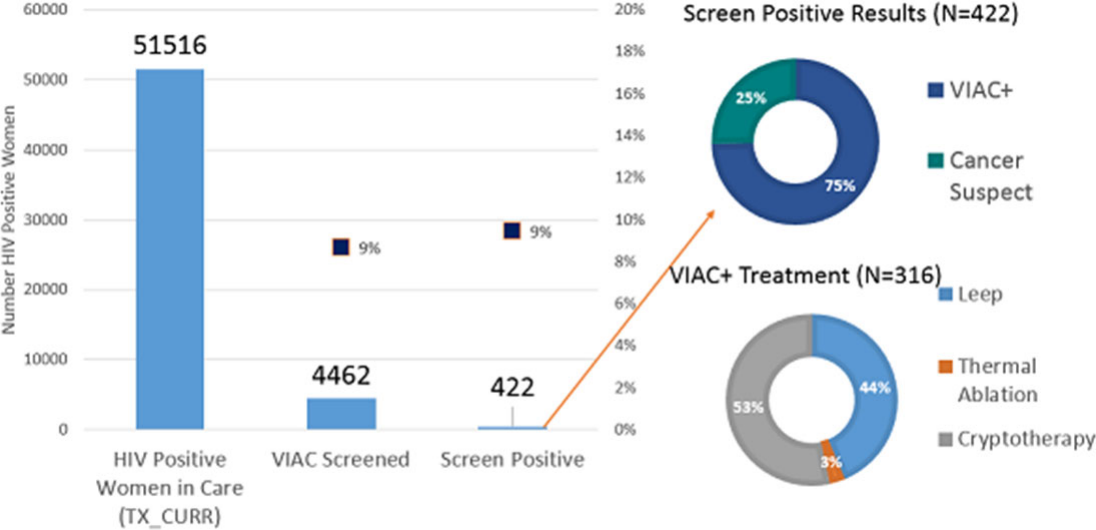

### MOPDD0104

#### Beyond the caregiver: diffusion of early childhood development knowledge and practices within the social networks of HIV‐positive mothers in Malawi

**K. Dovel^1,2^; S. Ejigu^2^; P. Kalande^2^; E. Udedi^2^; C. Mbalanga^2^; L. Bruns^1^ and T. Coates^1^**

^1^University of California Los Angeles, Division of Infectious Diseases, David Geffen School of Medicine, Los Angeles, United States, ^2^Partners in Hope, Lilongwe, Malawi

Early childhood development (ECD) is critical to the growth and well‐being of children. It is particularly important for children exposed to HIV ‐ who experience worse health and developmental outcomes than non‐exposed children. ECD programs targeting caregivers improve ECD outcomes. However, such programs are often limited in reach, focusing on individual caregivers; impact on the caregivers’ broader community and social‐network is unknown. We assessed ECD‐related perceptions and behavior among the social networks of HIV‐positive mothers who participated in a 9‐month integrated ECD‐antiretroviral therapy (ART) program in Malawi.

A subset of 30 randomly selected HIV‐positive mothers who completed the integrated ECD‐HIV program were asked to give study invitations to 7 friends/relatives > 18 years of age with whom they speak regularly. As a comparison, community‐based surveys were completed with adults > 18 years, using census data for randomization. Both populations completed a one‐time survey to assess ECD‐related knowledge and practices for infants < 12 months, using validated measures. Multivariate analyses (adjusted for age and sex) were conducted to examine differences between social‐networks and the broader community.

A total of 615 individuals were recruited (203 social‐network, 412 community) and 563 completed a survey (172 social network, 391 community). Social‐network and community respondents had a mean age of 31 and 38 years, 3 and 4 children, and 79% and 70% were married, respectively. Knowledge about the importance of ECD was dramatically higher among social network as compared to community respondents: 83% vs. 68% believed telling stories to infants was important (AOR:3.10 *p* < 0.001); and 97% vs. 57% believed singing was important (AOR:10.57 *p* < 0.001). There was also significant difference in practices that promote ECD among infants, such as actually making toys for infants (84% vs. 32%; AOR:8.03, *p* < 0.001), singing (87% vs. 63%; AOR:3.65, *p* < 0.001), telling stories (75% vs 49%; AOR:2.79, *p* < 0.001), and father involvement in feeding/bathing (68% vs 51%; AOR:2.51; *p*=0.001).

An integrated ECD‐HIV program targeting HIV‐positive mothers is associated with diffusion of ECD information and practices to mothers’ social‐networks. The reach of ECD programs may be greater than initially anticipated and should be explored further.

### MOPDD0105

#### Feasibility and outcomes of integrating diabetes screening into routine viral load monitoring among patients on antiretroviral therapy in Malawi

**V.H. Singano^1,2^; J.J. van Oosterhout^1,3^; A. Gondwe^1^; P. Nkhoma^1^; F. Caltado^1^; J. Theu^1^; M. Hosseinipour^4^; W. Ching'ani^5^; E. Singogo^1,6^ and A. Amberbir^1,7^**

^1^Dignitas International, Zomba, Malawi, ^2^Mothers2Mothers, Lilongwe, Malawi, ^3^College of Medicine, Department of Medicine, Blantyre, Malawi, ^4^University of North Carolina‐Malawi Project, Lilongwe, Malawi, ^5^Ministry of Health, District Health Office, Zomba, Malawi, ^6^University of Malawi ‐ Chancellor College, Department of Mathematical Sciences, Zomba, Malawi, ^7^Dalla Lana School of Public Health, University of Toronto, Toronto, Canada

People Living with HIV are at increased risk of diabetes mellitus due to HIV infection and exposure to antiretroviral therapy (ART). Despite this, structured diabetes screening has not been explored in African HIV clinics, possibly because of logistical challenges and uncertainty about the frequency of screening. We explored the feasibility and outcomes of diabetes screening, using the existing routine viral load (VL) monitoring schedule.

A mixed methods study was conducted from January to July 2018 among patients on ART aged ≥ 18 years at an urban HIV clinic in Zomba Central Hospital, Malawi. Patients who were due for routine 2‐yearly VL monitoring underwent a finger‐prick for simultaneous point‐of‐care glucose measurement and dried blood spot sampling for a VL test. Diabetes was diagnosed according to WHO criteria. Quantitative data on demographics and medical history were collected using an interviewer administered questionnaire and electronic medical records. In‐depth interviews were conducted among patients diagnosed with diabetes on the access, experience and perceptions regarding the integrated diabetes screening program.

1316 of 1385 (95%) patients undergoing routine VL monitoring had simultaneous screening for diabetes during the study period. The median age was 44 years (IQR: 38 to 83); 61 % were female; 28% overweight or obese; median ART duration was 83 months (IQR: 48 to 815); 49% were previously exposed to stavudine and 92% were virologically suppressed (<1,000 copies/mL). At the start of ART, median CD4 count was 199 cells/mm^3^ (IQR: 102 to 277), and 63% were in WHO clinical stages I or II.

Diabetes prevalence was 2.4%. Only two of 31 patients with diabetes were newly diagnosed. In multivariable analyses, diabetes diagnosis was associated with age > 40 years (aOR = 2.7; 95%CI: 1.6 to 4.6) and being on a protease inhibitor‐based regimen (aOR = 3.3; 95%CI: 0.8 to 13.0). Patients appreciated integrated screening saying it could lead to early diabetes diagnosis and easy access to diabetes care.

Integrating diabetes screening with routine 2‐yearly VL monitoring was feasible and appreciated by patients on ART. Diabetes prevalence was low. Cost‐effectiveness needs to be studied and could benefit from prioritizing adults above 40 years and on protease inhibitor‐based regimens.

### MOPDD0201

#### Sexual health education in a digital savvy adolescent generation: Efficacy of gamified learning in a low‐tech setting

**H. Haruna**

^1^University of Hong Kong, Faculty of Education, Hong Kong, Hong Kong, SAR of China, ^2^Ministry of Health, Human Resource for Health Development, Dodoma, Tanzania, United Republic of

This study provides a preview of the benefits of using innovative gamified learning solutions to educate adolescents on healthy sexual behaviour and reduce the increasing trend in chronic diseases related to unhealthy sexual habits. The relevance of our findings is significant given the geographical location to which we focused our intervention and the public health crisis associated with sexually transmitted diseases. A majority of adolescents affected with HIV/AIDS, engaging in underage sexual intercourse, sexual violence and abuse, and adolescents´ pregnancy are from Sub‐Saharan Africa (SSA). This study investigated the effectiveness of deploying innovative game‐based pedagogies in promoting healthy sexual behaviour and preventing risk sexual practices through health education among natives adolescent students in SSA.

In three iterations, a participatory research design involving active game‐users and other key stakeholders helped to develop and revise the digital gamified‐learning platform for sexual health education. A quasi‐experimental research design was conducted using two experimental conditions—game‐based learning and gamification—with an existing traditional teaching method serving as a control condition. In total, 348 (55.5% boys and 44.5% girls) students aged 11 to 15 were recruited from three secondary schools based in Dar es Salaam, Tanzania, to participate in a series of sexual health education topics.

In the three iterations of developing and revising, students under gamified‐learning achieved significantly more improvements in their sexual health knowledge than those under the traditional conditions: F(2, 345) = 210.554, *p* < 0.000. Moreover, feedback from gamified‐learning indicates that the two experimental conditions significantly improved the students´ motivation, boosted their attitude change, enhanced their knowledge acquisition, and fostered their engagement in active learning. In contrast, the traditional teaching method was shown to have largely failed to add value or generate students´ interest to engage in active learning.

These results suggest that digital gamified learning designed for sexual health education have a potential of fostering health and reproductive health education necessary for positive changes in healthy sexual behaviour, attitude, and practices among today´s digitally‐savvy adolescents. We believe that this study offers a significant contribution to addressing sexual health education and the public health concerns associated with unhealthy sexual behaviour among adolescent students in SSA.

### MOPDD0202

#### Managing stock levels of HIV commodities using electronic systems in Baylor Uganda, Rwenzori region

**J. Kabanda^1^; A. Mutebi^1^; A. Ketikinwa^2^; F. Abura^3^ and L. Namale^4^**

^1^Baylor College of Medicine ‐ Children's Foundation Uganda, Pharmacy, Kampala, Uganda, ^2^Baylor College of Medicine ‐ Children's Foundation Uganda, Clinical, Kampala, Uganda, ^3^Mulago National Referral Hospital, Pharmacy, Kampala, Uganda, ^4^Baylor College of Medicine ‐ Children's Foundation Uganda, Health Systems Strengthening, Kampala, Uganda

Availability of HIV Rapid Test kits and antiretroviral (ARV) medicines are contributing significantly contributed to attainment of the 95‐95‐95 UNAIDS targets. However, stock levels of HIV commodities in Uganda have been intermittent in the past one year with at least 1 to 1 commodities getting stocked out. Additionally available stock monitoring techniques have proven to be inaccurate due to the quality of the data and complexity of the tools. We assessed the contribution of using a Real‐time ARV Stock Status Monitoring tool (RASS) on stock management in Rwenzori region.

As part of project implementation, a computer based system was developed by METS/CDC to monitor weekly stock levels of ARVs and Rapid Test kits in health facilities. RASS is a dashboard based tool that monitors data on ARV supplies while integrating it with WAOS data (Patient numbers, orders and distributions) for enhanced decision making. Health workers were trained on use of the manual tool, SMS reporting and online reporting. They were also provided with smart electronic reporting devices. District mentors were identified and trained as super users to support and mentor persistently stocked out health facilities. Data used in this analysis was extracted from the RASS dashboard between June and September 2018.

Following the interventions stock out reporting rates of HIV commodities have declined from 13% in January 2018 to < 1% in December 2018. Use of the RASS tool has improved stock levels of HIV commodities through evidenced based redistributions/redirection of stock to low stocked health facilities hence combating any eminent stock outs. The program was also able to save close to $130,000 in costs for the ARVs distributed using the RASS tool.

The RASS tool provides accurate data for better decision making and it's easy and simple to use to all health workers across the region. Health service interruption was reduced and the country is on course to meet the 95‐95‐95 UNAIDS targets. Advocacy for other commodities like Anti‐TB medicines, OI medicines and EMHS should be added on the RASS tool to reduce the stock out of these commodities in health facilities which impacts on the care and treatment of clients.

### MOPDD0203

#### Empowering with PrEP (ePrEP) – a peer‐delivered online social network intervention for PrEP adoption among young Black and Latinx men who have sex with men: Cluster randomized controlled trial

**V.V. Patel^1^; N. Rios^1^; K.J. Horvath^2^; S.A. Golub^3^; C. Zhang^1^; K.H. Mayer^4,5^; R.S. Kim^6^; Z. Ginsburg^1^ and J.H. Arnsten^1^**

^1^Albert Einstein College of Medicine/Montefiore Health System, General Internal Medicine, Bronx, United States, ^2^University of Minnesota, School of Public Health, Minneapolis, United States, ^3^Hunter College of City University of New York, Psychology, New York, United States, ^4^Fenway Institute, Boston, United States, ^5^Harvard Medical School, Boston, United States, ^6^Albert Einstein College of Medicine, Epidemiology, Bronx, United States

Peer‐driven strategies leveraging existing online social networks may be an effective approach to engage young Black/Latinx men who have sex with men (YBLMSM) for HIV prevention. We developed and tested the feasibility, acceptability, and preliminary impact of a theory‐based, online intervention addressing barriers to PrEP adoption in a cluster randomized control trial.

ePrEP was a six‐week online campaign addressing PrEP barriers, developed and delivered by YBLMSM Influential Peers (IPs) via private Facebook/Instagram groups to their existing online‐social‐networks (e.g., friends/followers) in New York City. We recruited and randomized 10 IPs to ePrEP (n = 5) or an attention‐matched control (n = 5). IPs then recruited YBLMSM participants (aged 18 to 89, Black or Latinx, New York City resident, HIV‐uninfected, at high‐risk) from their online networks to a private online Facebook/Instagram group. IPs then posted condition specific contents to their respective groups and facilitated discussions about the contents. Outcomes included retention, acceptability, and PrEP related‐knowledge, ‐communication skills,‐stigma, and ‐use collected through online surveys (baseline, 6‐ and 12‐weeks).

Over three weeks from May‐June 2017, IPs recruited 423 YBLGBM; 155 screened eligible and were enrolled (ePrEP [n = 83] and control [n = 72]). Baseline characteristics were similar across groups and 18% were on PrEP. At 12‐weeks, >90% of participants were retained in both arms, 82% would continue participating, 78% reported high satisfaction, and 75% would recommend friends to participate. Engagement with online campaign posts was also similar in both arms (*p* = 0.7). At 12‐weeks, ePrEP participants compared to control, showed greater increases in knowledge (*p* = 0.02), communication skills about PrEP use (*p* = 0.06), and decreases in PrEP‐stigma (*p* = 0.06). Among those not on PrEP at baseline, there was a trend towards more new PrEP initiations in ePrEP (5/52) vs. Control (3/52) (*p* = 0.2) at 6‐weeks, but similar by 12‐weeks (6 vs. 5).

A peer‐developed and delivered online social‐network intervention was highly feasible, acceptable, and efficient in engaging YBLGBM at high‐risk of HIV‐infection for a PrEP‐uptake intervention, and may have utility for incorporation into programs to enhance PrEP uptake, adherence, and maintenance.

### MOPDD0204

#### Effectiveness of HIV Telehealth Training Program in increasing the level of knowledge of HIV care personnel in the Philippines: A pilot study

**R. Olete; K. Leyritana and C. O'Connor**

Sustained Health Initiatives of the Philippines, Mandaluyong City, Philippines

The rapid increase of HIV infection in the Philippines requires an equally aggressive response from the medical community. The paucity of infectious disease specialists has led physicians of general and varied specialties to head HIV centers and treat patients despite lack of rigorous training. Moreover, financial and geographic barriers present challenges in acquiring continuing medical education (CME) and referral systems to specialty centers needed for complicated clinical scenarios. HIV Telehealth Training Program (HTTP) was created as innovative model of learning, with HIV experts as teachers, to improve the level of knowledge among non‐specialist HIV care providers.

A single‐group, pre‐ and post‐test quasi‐experimental research was used to implement a bimodular web‐based curriculum to 62 HIV care providers purposively recruited from 12 HIV facilities in 2 regions within four months. The curriculum was divided into Basic and Advanced HIV and AIDS Management. Standardized online surveys using a 5‐point Likert scale was administered to measure perception toward the effectiveness of the program while a 10‐item quiz measured the level of knowledge before and after each class. Responses were quantitatively analyzed using heteroscedastic t‐test.

There is a significant improvement in the level of knowledge among HTTP participants reflected by a change in average scores from 5.4/10 to 6.9/10 in lecture‐based quizzes (*p* =< 0.05). Results of Likert scale revealed an improvement in perceived level of knowledge among trainees, with scores from 3.32 to 4.14 (*p* =< 0.05); trainees regarded HTTP topics as practical (4.27), useful for their work (4.15), and very satisfactory in terms of overall quality (4.20). The only barrier to HTTP's success is the internet strength perceived as neither poor nor excellent (3.88). All participants strongly agreed that HTTP can be used to strengthen referrals among health providers.

HTTP is acceptable and practical in terms of overall quality despite challenges in internet connectivity. It is also a cost‐effective approach in providing HIV non‐specialists with CME and a venue for referrals and shared expertise with colleagues and experts, especially for those located in rural and remote areas of the country.

### MOPDD0206

#### #endHIVbg: An innovative, targeted national campaign to engage hard‐to‐reach at‐risk MSM and transgender people with a confidential rapid oral home HIV test, in partnership with Grindr^TM^

**M. Baev^1,2^; E. Naseva^3^; I. Dimov^4^ and E. Patterson^5^**

^1^Single Step Foundation, HIV Program Manager, Sofia, Bulgaria, ^2^Medical University of Sofia, Department of Social Medicine, Sofia, Bulgaria, ^3^Medical University of Sofia, Faculty of Public Health, Sofia, Bulgaria, ^4^Single Step Foundation, Sofia, Bulgaria, ^5^Grindr for Equality, Project Manager, Washington D.C., United States

HIV testing services in Bulgaria are largely institutionalized, presenting a barrier to MSM and transgender people (the Target) who don't want to be associated with HIV testing due to stigma. The main hypothesis tested is that a large number of the Target don't use protection and don't regularly test for HIV. The objective of the research is to show that traditional methods are ineffective for these hard‐to‐reach communities and that home HIV testing marketed through Grindr^TM^ might be the only viable alternative for the Target in Bulgaria.

The methodology of the initiative included combination of distributing 900 free at‐home HIV tests, Grindr^TM^, and targeted online campaigns. The study was held for 1 month in October 2018 on a country level in Bulgaria (population of 7.1 mm), covering 1,574 MSM and transgender people, age 16 to 62. The study population includes MSM and transgender people (2.5%) of Bulgarian origin and 7% of minority background. Data collection was carried out through an online questionnaire. Pearson chi square test was applied (Fisher´s exact test when applicable). *p* < 0.05 were considered as significant.

Results show that 54% of the Target have never tested for HIV and that 71.5% prefer home HIV testing over traditional methods at centers or mobile clinics. 29.7% rarely or never use a condom during sex, and 52.9% do not know their HIV status. 67.8% of those unaware of their HIV status never or rarely use condom. The proportion of the respondents reporting usage of drugs is 13.7% and they rarely tend to use condoms compared to those who don't use drugs (*p* < 0.01).

Results show that a considerable number of at‐risk MSM and transgender people in Bulgaria have never been tested for HIV. The confidential, oral, at‐home HIV test distributed through Grindr^TM^ provides the only viable alternative for those who would not visit a medical center for testing. Changes in national legislation are needed to allow for more accessible testing outside the capital. This first of its kind community‐driven pilot model covering the territory of an entire country can be replicated in other countries with similar demographics and problems.

### MOPDD0207

#### Achieving 96% ARV collection compliance through innovative ATM technology

**S. Hendriksz**

Right to Care, Right ePharmacy, Pretoria, South Africa

The first Pharmacy Dispensing Unit (PDU) was deployed in South Africa in 2017 as part of a pilot project in partnership with National Department of Health to improve access to chronic medicine for public health patients. Consequently, PDU technology has been deployed to 5 different sites across the country in 2018. The PDU works like an ATM for medication, with Skype‐like audio‐visual interaction between patient and tele‐pharmacists, cloud based electronic software and robotic technology to dispense and label medication. It provides an innovative solution to chronic patients through increasing convenience, quality of service, ease of access and allows for timeous follow up of patients. This solution has proved particularly beneficial to HIV patients collecting antiretrovirals on a regular basis.

Standard data extraction is done monthly through the electronic interface to the PDU system. These extractions provide statistical figures on patient dispenses, including demographic information per region of operation.

Patient surveys were conducted to gage patient experience with this technology. Surveys were done through an external partner who obtained ethical approval.

The PDUs have made 106 314 dispenses by the end of Nov 2018, achieving up to a 96% collection rate over the 18 active PDUs. 68% of patients are women, mainly between the age of 40 and 49 years. Patients receiving 1^st^ line antiretroviral treatment as part of their chronic medication make up 79% of all patients served. PDU patients show a 99% overall satisfaction rate compared to 63% for patient attending local primary health clinics. It takes PDU patients a median of 10 minutes to collect medication while it takes 240 minutes at the clinic

The high collection rate and level of satisfaction for patients using the PDU compared to primary health clinics is indicative of the success of this solution for HIV patients. This solution potentially has the power to increase adherence and compliance through increasing the ease of access to quality pharmaceutical services for HIV patients. Viral Load monitoring of PDU patients is ongoing and will form the basis of reporting on clinical stability for these patients in future.

### TUPDA0101

#### Maternal HIV infection alters the community composition and dynamics of the enteric microbiome of associated infants

**B. Brown^1,2^; D. Chopera^3^; E. Havyarimana^4^; S. Jaumdally^4^; D. Martin^5^; A. Varsani^6^; H. Jaspan^1,2,4^ and InFANT Study Team**

^1^Seattle Children's, Center for Global Infectious Disease Research, Seattle, United States, ^2^University of Washington, Schools of Medicine and Public Health, Seattle, United States, ^3^Africa Health Research Institute, Durban, South Africa, ^4^University of Cape Town, Institute of Infectious Disease and Molecular Medicine, Cape Town, South Africa, ^5^University of Cape Town, Computational Biology Group, Cape Town, South Africa, ^6^Arizona State University, The Biodesign Center for Fundamental and Applied Microbiomics, Tempe, United States

Uninfected infants born to HIV‐infected mothers (HEU) are more vulnerable to diseases and have altered immunity and enteric microbial communities. The gut virome plays an important role in modulating both the bacterial microbiota and immunity of HIV infected individuals, yet the role of maternal HIV status in structuring the infant enteric virome remains unexplored.

Here, we longitudinally characterize the enteric viral communities of 5 HIV‐infected/exposed and 5 HIV‐uninfected/unexposed (HU) mother‐infant dyads from South Africa. Using shotgun sequencing of DNA isolated from virus‐like particles, we illustrate significant shifts in the community composition and diversity of the fecal virome between HIV‐infected mothers and their infants as compared to uninfected dyads.

We report significant shifts in the longitudinal dynamics of various eukaryotic virus and bacteriophage families between HEU and HU infants, and strong inverse correlations between phage diversity and bacterial target abundance, suggesting bacteria‐phage antagonism. Specifically, we describe elevated diversity of *Streptococcus* phage OTUs and significantly decreased *Streptococcus* abundance, as assessed via 16S rDNA sequencing. We also identified significantly greater similarity between viral communities of mothers and related infants than to unrelated infants, suggesting some amount of vertical viral transmission. Additionally, we have identified a suite of novel and known viruses from stools from HEU and HU infants and their mothers, including a novel crAssphage genome, the most abundant bacteriophage in the human gut. Across the crAAssphage genome, we report genome‐wide positive selection, with hotspots of selection in phage tail proteins, indicating bacteria‐phage antagonistic evolution. We further report average nucleotide identities across the crAssphage genome of > 99% within a dyad, as compared to 96 to 67% between dyads and compared to global strains, further supporting maternal transmission.

Overall, our results are the first to describe how maternal HIV infection affects isolate and community wide shifts in the enteric virome of HEU infants.

Abstract TUPDA0101‐Figure 1. The fecal virome of HIV infected mothers and exposed infants is more diverse than HU dyads.

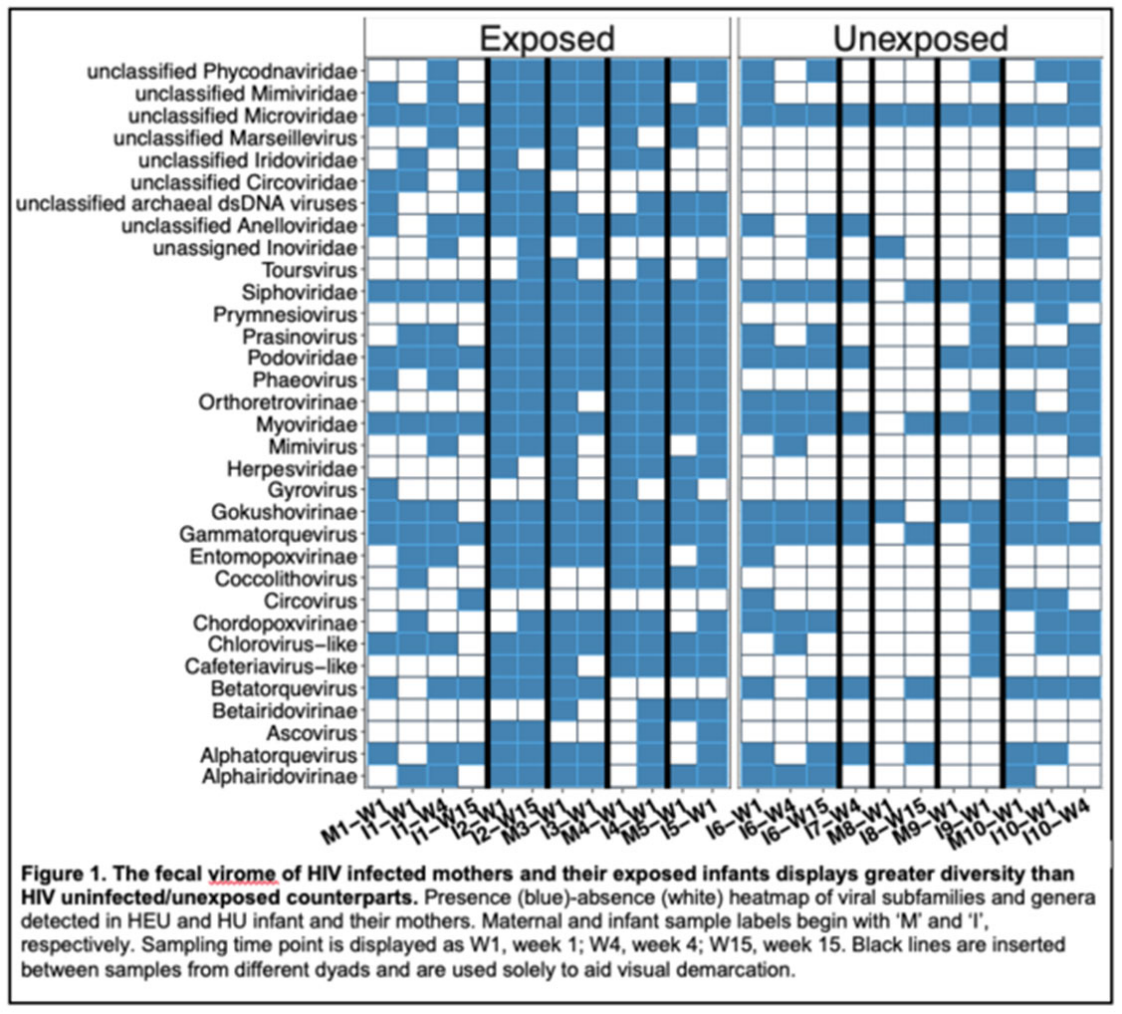

### TUPDA0102

#### Glycomic determinants of gut microbial dysbiosis and translocation during suppressed HIV infection

**L. Bertoni Giron^1^; C. E Tanes^2^; A. Anzurez^1^; P. A Engen^3^; L. M Mattei^2^; M. H Schleimann^4^; K. Bittinger^2^; P. W Denton^4^; F. Bushman^5^; H. Tateno^6^; A. Keshavarzian^3^; A. L Landay^3^ and M. Abdel‐Mohsen^1^**

^1^The Wistar Institute, Philadelphia, United States, ^2^CHOP Microbiome Program, Children's Hospital of Philadelphia, Philadelphia, United States, ^3^Rush University Medical Center, Chicago, United States, ^4^Aarhus University Hospital and Department of Clinical Medicine, Aarhus University, Arhus, Denmark, ^5^University of Pennsylvania, Philadelphia, United States, ^6^National Institute of Advanced Industrial Science and Technology ^AIST^, Tokyo, Japan

An emerging paradigm suggests that gut glycosylation is a key force in maintaining a homeostatic relationship between the gut and its microbiota. In the general population, changes in the gut glycome can alter the gut microbial composition, leading to microbial dysbiosis and gut inflammation. In HIV‐infected individuals, microbial dysbiosis and translocation contribute to the vicious cycle between HIV and immune activation/ inflammation. This vicious cycle likely contributes to the development of non‐AIDS inflammatory‐related illnesses and HIV persistence. However, how gut glycosylation machinery contributes to this cycle is yet to be characterized.

We used ileum, right colon, and sigmoid colon biopsies, as well as plasma, from 20 HIV‐infected individuals on suppressive antiretroviral therapy (ART) to examine: 1) gut glycomes using lectin microarray; 2) mucosal‐associated microbiome using 16S rRNA marker gene sequencing; 3) plasma markers of inflammation/microbial translocation using ELISA, and 4) gut‐associated HIV DNA levels using qPCR. Analysis was performed using Spearman´s rank correlation coefficient and linear mixed effects models. Nominal *p*‐values and Spearman´s Rho are reported.

Increased levels of mucosal‐associated, hypo‐sialylated *O* glycans (glycans with low sialic acid) correlated with lower gut microbiome diversity (*p = 0.001*,* rho *=* *‐0.68), higher *Bacteroidetes*/*Firmicutes* ratio (a marker of microbial dysbiosis; *p = 0.003*,* rho *=* *0.64*),* higher plasma levels of sCD14 *(*a marker of LPS‐mediated inflammation; *p = 0.007, rho *=* *0.58*),* and higher levels of ileum‐associated HIV DNA (*p = 0.028, rho *=* *‐0.56). Levels of mucosal‐associated a1 to 1 branched fucose correlated with higher microbiome diversity (*p = 0.032, rho *=* *0.48), lower *Bacteroidetes*/*Firmicutes* ratio (*p = 0.009, rho *=* *‐0.57*),* and lower plasma levels of sCD14 (*p = 0.03, rho *=* *‐0.48). Last, levels of ileum‐associated galactosylation correlated with lower levels of sCD14 (*p *<* *0.0001, *rho *=* *‐0.8).

Our pilot study provides the first proof‐of‐concept evidence that differential gut glycomic patterns (mainly sialylated and fucosylated glycans), during ART‐suppressed HIV infection, support distinct microbiome compositions that predispose to microbial translocation, inflammation, and HIV persistence. Our data are consistent with previous general population reports which demonstrated that sialic acid catabolism drives microbial dysbiosis and intestinal inflammation and that gut fucosylation sustains host‐commensal symbiosis as well as prevents gut inflammation. Exploiting gut glycosylation machinery may allow the design of strategies to manipulate it to treat HIV and/or prevent/delay the development of HIV‐associated co‐morbidities.

### TUPDA0103

#### Relevance of Reg3α and I‐FABP on microbial translocation, inflammation and reservoir size in people living with HIV

**S. Isnard^1,2^; R. Ramendra^1,2^; F.P. Dupuy^1,2^; V. Mehraj^1,2,3^; J. Lin^1,2^; N. Kokinov^1,2^; B. Lebouché^1,2^; C. Costiniuk^1,2^; P. Ancuta^3^; N.F. Bernard^1,2^; M. Durand^3^; C. Tremblay^3^; J.‐P. Routy^1,2,4^; Montréal Primary HIV‐infection Study Group. Canadian HIV and Aging Cohort, and Canadian HIV Infected Slow Progressors Study**

^1^Research Institute of McGill University Health Centre, IDIGH, Montréal, Canada, ^2^McGill University Health Centre, Chronic Viral Illness Service, Montréal, Canada, ^3^Centre de Recherche du Centre Hospitalier de l'Université de Montréal, Microbiologie, Infectiologie, Immunologie, Montréal, Canada, ^4^Division of Hematology McGill University Health Centre, Montréal, Canada

Epithelial gut damage persists during HIV‐infection despite antiretroviral therapy (ART) and has been associated with microbial translocation, immune activation, and the development of non‐AIDS events. Regenerating islet‐derived protein 3α (Reg3α) is an intestinal anti‐microbial protein secreted by Paneth cells in response to mucosal damage. Reg3α plasma levels have been reported to be a predictor of graft vs host disease and elevated in people with inflammatory bowel diseases and obesity. We assessed the association between two markers of gut damage, Reg3α and intestinal fatty acid binding protein (I‐FABP), and microbial translocation, inflammation, as well as reservoir size in people living with HIV (PLWH).

169 adult PLWH, categorized into early (EHI n = 51) or chronic HIV infection (CHI n = 88) or elite controllers (EC n = 30), and uninfected controls (UC n = 30) were analyzed. A sub‐group of EHI were assessed prospectively. Plasma Reg3α levels were measured by ELISA and correlated with markers of disease progression, epithelial gut damage, and microbial translocation [lipopolysaccharide (LPS) and (1‐>3)‐β‐D‐glucan (βDG)]. Size of HIV reservoir was assessed by integrated HIV DNA via nested qPCR in CD4 T cells.

In a cross‐sectional analysis, plasma Reg3α levels were significantly elevated in untreated EHI (1938 ± 374 pg/ml), CHI (3084 ± 293), ART‐treated CHI (2441 ± 630) and EC (1442 ± 270) compared to uninfected controls (715 ± 243). Over the course of two years, plasma Reg3α levels increased in 11 PLWH without ART (*p* = 0.03) and decreased in 10 individuals who initiated ART during EHI (*p* = 0.048). Spearman analyses revealed that Reg3α levels positively correlated with viral load (r = 0.37, *p* = 0.0009), I‐FABP (*p* < 0.0001,r = 0.34), LPS (*p* < 0.0001,r = 0.46) and βDG (*p* = 0.02,r = 0.17). Conversely, Reg3α levels negatively correlated with CD4 count (r = ‐0.29, *p* = 0.0002) and CD4/CD8 ratio (r = ‐0.31, *p* < 0.0001). Compared to I‐FABP, Reg3α levels had stronger correlations with CD4 Count, CD4/CD8 ratio, VL, LPS, and 5 pro‐inflammatory cytokines. In addition, Reg3α but not I‐FABP levels correlated with the frequency of integrated HIV DNA in CD4 T cells (r = 0.3, *p* = 0.04 vs r = ‐0.15, *p* = 0.29).

Plasma levels of Reg3α were increased during HIV infection and did not normalize with ART. Reg3α represents a more promising epithelial gut damage marker than I‐FABP in PLWH, and may help evaluate HIV remission interventions.

### TUPDA0104

#### HIV acquisition risk and genital inflammation associated with hormonal contraceptives is dependent on the vaginal microbiome

**L. Noel‐Romas^1^; M. Perner^1^; R.P. Molatlhegi^2^; C. Farr Zuend^1^; A. Mabhula^3^; S. Mutch^1^; A. Lamont^1^; K. Birse^1^; A. Berard^1^; S. McCorrister^4^; G. Westmacott^4^; A. Leslie^3^; V. Poliquin^1^; R. Heffron^5^; L.R. McKinnon^1^ and A.D. Burgener^1^**

^1^University of Manitoba, Winnipeg, Canada, ^2^University of KwaZulu Natal, Durban, South Africa, ^3^African Health Research Institute, Durban, South Africa, ^4^Public Health Agency of Canada, Winnipeg, Canada, ^5^University of Washington, Seattle, United States

The injectable hormonal contraceptive depot medroxyprogesterone acetate (DMPA) has been associated with increased risk of HIV‐1 acquisition in women, but observations have been inconsistent between studies. We used a proteomics‐based systems biology approach to examine whether the vaginal microbiome influences inflammation and rates of HIV incidence in women using different hormonal contraceptives in an HIV prevention trial in South Africa (n = 685).

Cervicovaginal mucosal specimens from 61 women who went on to acquire HIV within the trial (cases), and all available women who remained uninfected (controls, n = 624), were analyzed by mass spectrometry‐based proteomics.

Nearly all women were using hormonal contraceptives (97.7%) including DMPA (65.6%), norethisterone enanthate (NET‐EN) (18.0%), and combined oral contraceptives (COC) (14.2%). Two major vaginal microbiome profiles were observed, one dominated by *Lactobacillus* species (59.4%), and the other that was non‐*Lactobacillus* dominant (40.6%), where *Gardnerella vaginalis* predominated with other facultative and anaerobic bacteria. Microbiome groups were similarly distributed among contraceptive types. A case‐control analysis showed the probability of HIV infection was not different in those using DMPA when compared to NET‐EN and COC users as a single group (OR: 1.56, 95% CI: 0.87 to 2.95, *p = *0.151). In non‐*Lactobacillus* dominant women, the risk of HIV acquisition was not significantly higher in those using DMPA compared to all other hormonal contraceptives (OR: 0.95, 95% CI: 0.44 to 2.15, *p = *0.895). However, in *Lactobacillus*‐dominant women, DMPA use was associated with a > 3‐fold increase in HIV acquisition risk for DMPA users relative to women using other hormonal contraceptives (OR: 3.27; CI: 1.24 to 11.24, *p = *0.0305). Interaction analysis suggested a statistical trend toward the vaginal microbiome having an impact on DMPA‐associated HIV‐risk (*p = *0.0686). Serum MPA levels associated with increased glucose metabolism and immune activation pathways in cervicovaginal mucosa in *Lactobacillus*‐dominant women, which were already activated in non‐*Lactobacillus* dominant women, and these biomarkers associated with increased frequency of activated cervical CD4 + T‐cells (HLA‐DR+CD38 + ).

This study provides evidence that women with vaginal *Lactobacillus* may be more susceptible to the negative influences of DMPA‐associated genital inflammation and HIV acquisition risk

### TUPDA0105

#### Recent injectable contraception correlates with reduced cervicovaginal mucosal growth factor expression in South African women

**R.P. Molatlhegi^1,2^; L.J. Liebenberg^2,3^; A. Leslie^4^; L. Noel‐Romas^5^; A. Mabhula^4^; M. Perner^6,7^; K. Birse^5,8^; S. Ngcapu^1,2^; B. Tlou^9^; J.H. Adamson^4^; K. Govender^10^; N.J. Garrett^2^; N. Samsunder^2^; A.D. Burgener^5,11,12^; S.S. Abdool Karim^2,5^; Q. Abdool Karim^2,5^; J.‐A.S. Passmore^2,13^ and L.R. McKinnon^2,8,12^**

^1^University of KwaZulu‐Natal, Medical Microbiology, Durban, South Africa, ^2^CAPRISA, Durban, South Africa, ^3^University of KwaZulu‐Natal, Durban, South Africa, ^4^African Health Research Institute, Durban, South Africa, ^5^Public Health Agency of Canada, National HIV and Retrovirology Labs, Winnipeg, Canada, ^6^University of Manitoba, Department of Medical Microbiology, Winnipeg, Canada, ^7^University of Manitoba, Department of Obstetrics & Gynecology, Winnipeg, Canada, ^8^University of Manitoba, Medical Microbiology, Winnipeg, Canada, ^9^University of KwaZulu‐Natal, School of Nursing and Public Health, Durban, South Africa, ^10^Africa Health Research Institute, Durban, South Africa, ^11^University of Manitoba, Medical Microbiology and Obstetrics & Gynecology, Winnipeg, Canada, ^12^Karolinska University Hospital, Unit of Infectious Diseases, Department of Medicine Solna, Center for Molecular Medicine, Karolinska Institute, Stockholm, Sweden, ^13^University of Cape Town, Institute of Infectious Disease and Molecular Medicine, Cape Town, South Africa

Injectable contraceptives have been associated with HIV‐acquisition risk and mucosal immune changes, but studies have reported inconsistent results. Challenges include the inaccuracy of self‐reported data, unknown timing of injection, and interactions with other mucosal transmission co‐factors. To address this, we quantified the concentration of injectable contraceptives in plasma, estimating how recently women received an injection.

Plasma medroxyprogesterone acetate (MPA) was quantified in women from the CAPRISA004 study (n = 722), with parallel quantitation of 48 cytokines in cervicovaginal lavage (CVL). Each cytokine was tested as an outcome in linear‐mixed models; the main explanatory variables included MPA concentration, modelled as a categorical variable, along with additional covariates (age, study arm, study site, number of sex acts, condom use and baseline HSV‐2 serostatus) in multivariable models. Interaction analyses examined the impact of age, HSV‐2 and the vaginal microbiome on MPA‐cytokine associations. Mass spectrometry was used to profile host CVL‐proteins associated with MPA and/or cytokine concentrations (n = 443).

Almost all women (467/481; 97.1%) who reported DMPA use had detectable plasma MPA (>10 pg/ml), while MPA was detected (mostly at low levels) in 13.9% of non‐DMPA users. Compared to women with undetectable levels, those with high MPA levels (≥800 pg/ml) had reduced CVL concentrations of GCSF, MCSF, IL16, CTACK, LIF, IL1A, and SCGFB. Similar results were observed in multivariable analyses. The strongest associations were with GCSF and MCSF. Host proteomic analyses revealed significant clustering by MPA concentration; low MPA/high GCSF and MCSF women had higher MUC16, MUC5B and PIGR levels, proteins important for mucosal fluid function, factors involved in keratinization, growth factors (GF), protein processing, integrin binding, and sugar metabolism. While pro‐inflammatory cytokines were not associated with MPA levels, in stratified analyses we observed positive IP10‐MPA associations in older women and women who were HSV‐2 seropositive. MPA‐cytokine associations also frequently differed based on the expression of vaginal microbiome proteome, with *Gardnerella vaginalis*‐dominant samples having elevated IL‐8, MCP‐1 and IP‐10.

High plasma MPA concentration, reflective of recent DMPA injection, was associated with reduced levels of several genital cytokines including GFs is a dose‐dependent fashion. Age, HSV‐2 and the vaginal microbiome may modify DMPA‐CVL cytokine associations.

### TUPDA0106

#### The effect of hepatitis C (HCV) cure on markers of macrophage activation and microbial translocation among HIV seropositive women

**K. Burke^1^; E. Daubert^2^; K. Weber^2^; E. Seaberg^3^; M. Peters^4^; M. Augenbraun^5^; K. Workowski^6^; R. Morack^2^; R. Franco^7^; M. Fischl^8^; M. Kuniholm^9^; S. Kassaye^10^; A. Adimora^11^ and A. French^1^**

^1^CORE Center/Cook County Health, Chicago, United States, ^2^Chicago WIHS/ CORE Center of Cook County Health, Chicago, United States, ^3^Johns Hopkins University Bloomberg School of Public Health, Baltimore, United States, ^4^University of California San Francisco, San Francisco, United States, ^5^State University of NY, Downstate, Brooklyn, United States, ^6^Emory University, Atlanta, United States, ^7^University of Alabama, Birmingham, Birmingham, United States, ^8^University of Miami, Miami, United States, ^9^Albert Einstein University, Bronx, United States, ^10^Georgetown University, Washington, United States, ^11^University of North Carolina, Chapel Hill, United States

HCV infection is highly prevalent in HIV and is associated with excess liver‐related and all‐cause mortality. HCV cure decreases risk of hepatocellular carcinoma and liver‐related mortality however the effect of HCV cure on other HIV associated morbidities is unclear. We examined the effect of HCV cure on markers of macrophage activation and microbial translocation (soluble CD163 and sCD14 respectively) which have been implicated in the pathogenesis of serious non‐AIDS morbidities.

We studied 126 HIV/HCV coinfected Women´s Interagency HIV Study (WIHS) participants who had been successfully treated for HCV as of March 2018 and had available serum from pre‐ and post HCV treatment. HCV cure was defined as undetectable HCV RNA at least 12 weeks after completion of HCV therapy. sCD163 and sCD14 were measured in duplicate by ELISA (R&D Systems) on samples frozen at ‐80C. The final result was the mean of the duplicate tests. Multivariate analysis of variances was used to compare differences in log‐transformed pre‐ and post‐ treatment sCD163 and sCD14.

Participants were 52% African‐American, 16% Hispanic, 20% other/multiracial. Pretreatment the mean age was 56.3 years, mean CD4 was 615, 72% had suppressed HIV RNA and 10% had cirrhosis by APRI or FIB‐4. 114 participants had directly acting agent based HCV therapy and 12 had interferon based therapy. Results were similar when therapy groups were analyzed separately so results are presented for the full group. sCD163 and sCD14 significantly decreased from pre to post‐treatment in unadjusted analyses (Table). In analyses using log‐transformed values and adjusting for age, race, liver fibrosis stage, drug and alcohol use, CD4 and viral suppression status, the decrease in sCD163 and sCD14 remained significant.

Successful treatment of hepatitis C significantly decreased markers of microbial translocation and macrophage activation in HIV/HCV co‐infected women. Larger, longitudinal analyses are needed to determine if these changes in markers predict reductions in HIV associated non‐AIDS co‐morbidities.

Abstract TUPDA0106‐TABLE 1. Pre‐ and post‐HCV treatment serum markers of macrophage activation and microbial translocation

**Table:** 

Mean (SD)	Pre‐treatment	Post‐Treatment	*p* value	Wilks´ Lambda (F) for adjusted	Adjusted *p* value
sCD163 (ng/mL)	1461.7 (920.8)	901.7 (539.4)	<0.001	0.948 (6.07)	0.015
sCD14 (pg/mL)	2256332 (5231256)	2102740 (497910)	0.017	0.948 (6.03)	0.016

### TUPDB0101

#### Apples and oranges: Assessment of the care cascade in sub‐Saharan Africa

**C. Mugglin^1^; D. Klaeger^1^; A. Gueler^1^; F. Vanobberghen^2^; B. Rice^2^ and M. Egger^1^**

^1^University of Bern, Institute of Social and Preventive Medicine, Bern, Switzerland, ^2^London School of Hygiene & Tropical Medicine, Faculty of Public Health and Policy, London, United Kingdom

In 2014 UNAIDS adopted the 90‐90‐90 targets to track progress towards ending the HIV epidemic. To achieve universal access to HIV care and treatment with viral suppression, each HIV‐positive individual must progress along the cascade of care. We examined the methods used in published studies of the HIV care cascade, focussing on sub‐Saharan Africa (SSA), where most people with HIV live.

Systematic review to identify papers reporting on at least two steps of the HIV care cascade. We assessed definitions used for numerators and denominators for each step in the UNAIDS 90‐90‐90 cascade and an adapted WHO cascade framework.

Fifty‐eight studies met the inclusion criteria: 44 cohorts, 10 cross‐sectional and 4 mixed design studies. Fourteen SSA countries were represented; nearly half (N = 27) of the studies were from South Africa. Eight studies covered the whole cascade from PLHIV to viral suppression. The steps covered most frequently were retention on antiretroviral therapy (ART) (N = 22) and viral suppression (N = 34). The proportion of studies reporting definitions for numerator and denominator ranged from 36% to 100% (Table). Definitions used for calculating percentages at each step differed. For example, for PLHIV, denominators were population‐based, clinic‐based or participants of testing campaigns. For linkage to pre‐ART care, denominators included self‐reported or laboratory confirmed test results. For ART initiation, denominators ranged from those newly‐diagnosed with HIV infection to those eligible for ART, and numerators included self‐reported ART initiation, first recorded prescription of ART or evidence of antiretrovirals in blood samples.

Definitions used for the steps of the cascade were not reported in many published studies. Where reported they were heterogeneous, that results cannot be compared across studies. To allow tracking of progress along the care pathway and towards the 90‐90‐90 targets, more complete reporting, comparable measures and clear definitions of numerator and denominator at each step are urgently needed.

Abstract TUPDB0101‐Table 1. Number of papers reporting definitions and different definitions used for the eight steps of the HIV care cascade in sub‐Saharan Africa (n = 58)

**Table:** 

HIV care cascade step	No. of studies reporting on step (%)	No. of studies reporting definition for numerator and denominator (%)	No. of definitions used for numerator	No. of definitions used for denominator
People living with HIV	8 (14%)	3 (38%)	3	3
Diagnosed with HIV	24 (41%)	17 (71%)	4	4
Linked to pre‐ART care	26 (45%)	17 (65%)	7	6
Retention in pre‐ART care	17 (29%)	13 (76%)	6	3
ART initiation	33 (57%)	12 (36%)	7	4
On ART	12 (21%)	7 (58%)	5	2
Retention on ART	22 (38%)	20 (91%)	4	2
Viral suppression	34 (64%)	34 (100%)	7	2

### TUPDB0102

#### Gaps in the continuum of care. Loss to follow‐up and return to care: Who is at risk?

**B.A. Martinez‐Guerra; A. Camiro‐Zuñiga; Y. Caro‐Vega; P.F. Belaunzarán‐Zamudio; J. Sierra‐Madero and B. Crabtree‐Ramirez**

Instituto Nacional de Ciencias Médicas y Nutrición Salvador Zubirán, Departamento de Infectología, Mexico City, Mexico

People living with HIV (PLWH) require constant adherence to antiretroviral therapies (ART) and clinical care. Patients linked to care but not retained may contribute to HIV transmission. Little is known about the gaps in the continuum of care, loss to follow‐up (LTFU) frequency and its associated factors in Mexico. We aimed to compare characteristics of PLWH constantly‐retained in care (CRIC), definitively‐lost to follow‐up (dLTFU) and returning to‐care (RTC) after a period of absence.

Observational retrospective cohort study including HIV‐positive adults with at least one clinical visit (CV) from 1987 to 2017 at an HIV Clinic in Mexico City. Patients who missed CVs for 12 months or longer were considered LTFU. When CVs were registered after LTFU, patients were classified as RTC. Patients dLTFU didn't have any CV after the initial LTFU episode. We compared demographic and clinical characteristics at enrollment between patients CRIC, dLTFU and RTC.

2,967 patients were included (89% were male); 1,565(53%) were CRIC, 826(28%) dLTFU and 576(19%) RTC. Median time to LTFU was 1.28(0.25 to 5.48) years. Among RTC patients, median absence lasted 1.59(1.21 to 1.62) years and median number of LTFU episodes was 2(1 to 1). RTC patients were younger at enrollment (32yo) than those CRIC and dLTFU (34yo each, *p *<* *0.01). Table‐1 summarizes demographic and clinical characteristics. RTC (23.8%) and dLTFU (19.4%) were more frequently initiated on protease‐inhibitor based regimens than CRIC patients (10.6%, *p *<* *0.01). Median time to ART initiation in the CRIC was significantly shorter than in those dLTFU and RTC (3[0.71 to 1.71] *vs* 6.07[1.86 to 61] weeks, *p* < 0.001).

Gaps in the continuum of care were frequent. A high proportion of patients LTFU (41%) returned to care. Young age, heterosexual transmission, low literacy, advanced HIV‐infection, delayed ART initiation and initiation of IP‐based regimens were more frequent in people with gaps in the continuum of care. The importance of prompt initiation of ART is underscored.

Abstract TUPDB0102‐Table 1. Demographic and clinical characteristics at enrollment

**Table:** 

Characteristic at enrollment	CRIC group n = 1,565	dLTFU group n = 826	RTC group n = 576	*p*
Men who have sex with men n(%)	1,077 (68.8)	525 (63.6)	389 (67.5)	
Heterosexual men n(%)	222 (14.2)	149 (18.0)	91 (15.8)	<0.01 for comparison between transmission modes
Heterosexual women n(%)	140 (8.9)	87 (10.5)	71 (12.3)	
Other n(%)	126 (8.1)	65 (7.8)	25 (4.3)	
Enrollment before 2010 n(%)	635 (40.6)	582 (70.5)	501 (87.0)	<0.01
School education ≤ 9 yr n/available data (%)	327/1,507 (21.7)	200/757 (26.4)	105/478 (22.0)	0.04
AIDS events n/available data (%)	499/1,536 (32.5)	326/811 (40.2)	222/571 (38.9)	<0.01
CD4 cell count < 200cell/uL n(%)	752 (48.1)	455 (55.1)	227 (39.4)	<0.01

### TUPDB0103

#### Understanding gaps in the HIV treatment cascade in 11 West African countries: Findings from the regional Community Treatment Observatory

**G. Oberth^1^; S. Baptiste^2^; W. Mosime^3^; A. Maouan^4^; P. Garcia^4^; T. Taro^5^; O.B.K. Gueye^4^; A.M. Traore^4^ and R. Boka^4^**

^1^University of Cape Town, South Africa, Center for Social Science Research ^CSSR^, Cape Town, South Africa, ^2^International Treatment Preparedness Coalition ^ITPC^, Johannesburg, South Africa, ^3^International Treatment Preparedness Coalition ^ITPC^, Gaborone, Botswana, ^4^International Treatment Preparedness Coalition ^ITPC^, Abidjan, Cote D'Ivoire, ^5^International Treatment Preparedness Coalition ^ITPC^, New York, United States

In West and Central Africa, 48% of people living with HIV (PLHIV) are aware of their status, 40% are accessing antiretroviral therapy (ART), and 29% are virally suppressed. Progress is stymied by drug stock‐outs, weak health systems, human rights barriers, and low quality of care. In February 2017, the International Treatment Preparedness Coalition (ITPC) established the Regional Community Treatment Observatory in West Africa (RCTO‐WA) to increase accountability for the 90‐90‐90 targets.

ITPC trained and supported national networks of PLHIV to collect and analyze facility‐level data along the HIV treatment cascade from 103 health centers in 11 West African countries. From July 2017‐June 2018, the RCTO‐WA conducted 538 health center visits, 279 key informant interviews, and 110 focus group discussions. In this paper, we share the first year of RCTO‐WA community monitoring findings, analyzed using the ‘Five As’ framework—availability, accessibility, acceptability, affordability and appropriateness.

*Availability:* ART stock‐outs were recorded during 23.4% of health facility visits (95% confidence interval [CI] 19.8%‐27.0%), lasting an average of 40.5 days (95% CI 34.2 to 26.7). Stock‐outs were less common for HIV tests and viral load supplies. *Accessibility*: Long distances to health centers was the top cited barrier to HIV testing and ART. Linkage to care was high overall (4,692 positive tests; 4,354 ART initiations), but was lower among key and vulnerable populations, and countries without test‐and‐treat. Among 81,817 people on ART, 16,491 viral load tests were performed. *Acceptability:* A third of participants rated the quality of services a 3 or less out of 5. A quarter of viral load test results were returned within two weeks, with faster turnaround time associated with improved viral suppression (*p* < .05). *Affordability:* Payment was not cited as a major barrier to services. *Appropriateness:* Key and vulnerable populations made up 16% of positive tests but just 7% of people on ART. Young men were less likely to access services than young women.

To achieve the 90‐90‐90 targets, ongoing community monitoring is critical. The RCTO‐WA highlights key access gaps along the HIV treatment cascade. National and regional advocacy should focus on expanding differentiated service delivery and removing gender‐ and human rights‐related barriers.

### TUPDB0104

#### The “Failure Cascade” for patients with unsuppressed viral load in Zambia: Results from a large HIV treatment cohort

**R. Warrier^1,2^; J.J. Pry^1,3^; P. Elish^1^; P. Kaumba^1^; H. Smith^1^; I. Sikazwe^1^; C. Bolton^1,2^ and M. Herce^1,4^**

^1^Centre for Infectious Disease Research in Zambia, Lusaka, Zambia, ^2^University of Alabama at Birmingham, Birmingham, United States, ^3^University of California Davis, Davis, United States, ^4^University of North Carolina at Chapel Hill, Chapel Hill, United States

Achieving the 3^rd^ 90 demands sustained anti‐retroviral therapy (ART) and routine viral load (VL) monitoring for people living with HIV (PLHIV) to ensure viral suppression (i.e. VL < 1,000 copies(c)/ml). In Zambia, ART‐treated PLHIV with VL ≥ 1,000 c/ml enter a complex “failure” cascade requiring enhanced adherence counseling (EAC), follow‐up VL testing (within 90 days), and possible switch to second‐line ART. Here, we report the first “failure” cascade from Zambia for a large PEPFAR‐funded HIV treatment cohort supported by the Centre for Infectious Disease Research in Zambia (CIDRZ).

We abstracted routine data from electronic health records for all PLHIV > 18 years who accessed ART services in 74 CIDRZ‐supported facilities across 3 Zambian provinces and had ≥ 1 documented VL between January 1, 2016—September 30, 2018. We describe the failure cascade using summary statistics.

Figure 1 depicts patient flow in the failure cascade. Of 118,266 patients with a documented first VL, 12.1% (n = 14,291) were unsuppressed. Of those, 9.2% had a follow‐up VL drawn within 90 days, at a median of 266 days (IQR: 174 to 402). Time to first follow‐up VL did not differ by gender (*p* = 0.23), but was faster for adolescents (18 to 84 years) compared to older (>25 years) PLHIV (*p* < 0.001). Half of patients with a follow‐up VL achieved viral suppression (n = 2,519, 50.6%), while 49.4% (n = 2,459) experienced virological failure (i.e. two consecutive unsuppressed VLs). Of 2,459 with virological failure, only 720 (29.3%) switched to second‐line ART per guidelines.

For ART‐treated PLHIV with an unsuppressed routine VL in Zambia during the evaluation period, we observed gaps with provision of follow‐up VL testing and substantial testing delays. Of those with virological failure, only about one‐third receive second‐line ART. New differentiated service delivery models are needed that offer unsuppressed patients expedited clinical and laboratory services, including EAC, follow‐up VL and HIV genotype testing, and ART regimen change.

Abstract TUPDB0104‐Figure 1. Patient Flow in the HIV Viral Load Failure Cascade.

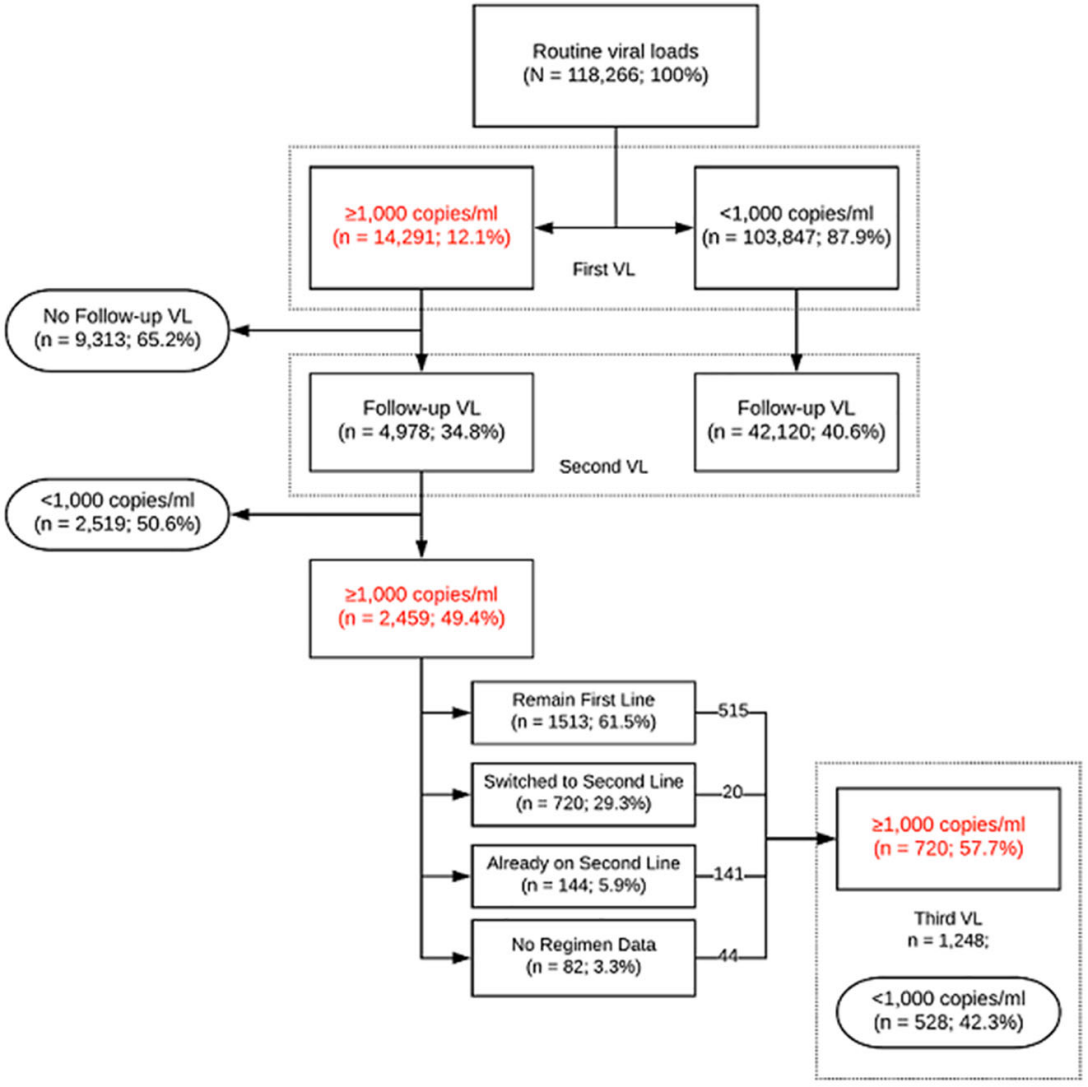

### TUPDB0105

#### The hepatitis C treatment cascade in the era of direct‐acting antivirals (DAAs), and barriers to DAA treatment initiation, among US men and women with and without HIV

**D. Haley^1,2^; A. Edmonds^2^; C. Ramirez^2^; A. French^3^; P. Tien^4^; C. Thio^5^; E. Seaberg^5^; M. Plankey^6^; M. Witt^7^; M. Cohen^8^; C. Oramasionwu^2^ and A. Adimora^2^**

^1^Northeastern University, Boston, United States, ^2^University of North Carolina at Chapel Hill, Chapel Hill, United States, ^3^Stroger ^Cook County^ Hospital, Chicago, United States, ^4^University of California San Francisco, San Francisco, United States, ^5^Johns Hopkins University, Baltimore, United States, ^6^Georgetown University Medical Center, Washington, DC, United States, ^7^David Geffen School of Medicine at UCLA, Los Angeles, United States, ^8^Stroger Hospital, Chicago, United States

People with HIV are disproportionately co‐infected with the Hepatitis C virus (HCV) and experience accelerated liver‐related morbidity and mortality. Direct‐acting antivirals (DAAs) are well‐tolerated and yield high sustained virologic response (SVR) rates. However, DAA uptake is low. This study characterizes the DAA‐era HCV treatment cascade among US men and women with and without HIV and identifies treatment barriers.

We constructed HCV treatment cascades using data from two observational cohorts: Women's Interagency HIV Study (women, six semiannual visits, 2015 to 5018, n = 2,447) and Multicenter AIDS Cohort Study (men, one visit, 2016 to 6017, n = 2221). Cascades included HCV treatment‐eligible individuals, defined as HCV RNA+ or reported DAAs. Surveys captured clinical (e.g., CD4/viral load, poor health), patient (e.g., missed visits), system (e.g., appointment access), and financial (e.g., insurance) barriers.

323 women and 92 men were HCV RNA+ or reported DAAs. Most women/men had HIV (77%/70%) and were Black (69%/63%); median age (interquartile range) was 56 (51 to 10) and 58 (55 to 53), respectively. Despite similar treatment interest, HIV+ women were more likely to attain cascade outcomes than HIV‐ women (82% vs. 61% recommended, 69% vs. 43% initiated, with 63% of HIV+ vs. 37% of HIV‐ women achieving SVR); similar discrepancies were noted for men (Figure 1). Men were less likely to progress through the cascade. Individuals reporting substance use and Black men were less often treated. Women initiating treatment (vs. those not) differed in proportions of visits with reported patient (14%/33%) and system barriers (34%/17%), but not clinical (32%/35%) or financial barriers (26%/24%). Among men not treated, clinical barriers were most often reported (53%), compared to patient (1%), system (2%), and financial (2%) barriers.

In our cohorts, people with HIV were more likely to receive treatment for HCV and attain SVR. HIV‐related care may facilitate navigation of HCV treatment barriers. HIV‐ individuals, Black men, and substance users may need additional support.

Abstract TUPDB0105‐Figure 1. Direct‐acting antiviral Hepatitis C treatment cascade among US men and women, by HIV status (n = 415).

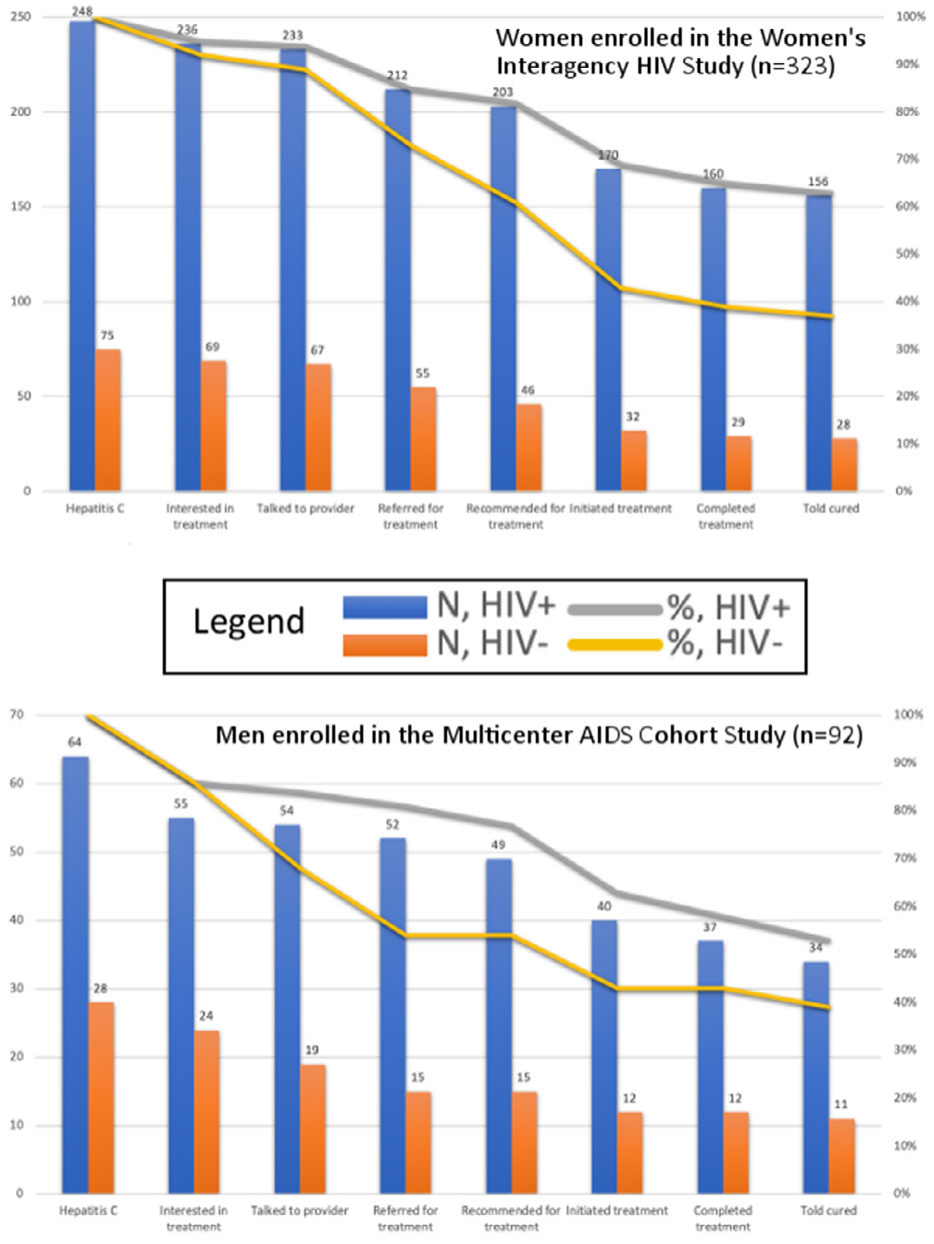

### TUPDB0106

#### *Actively contributing to a cascade of change:* Analysis of the TB treatment cascade among children and adolescents living with HIV in six high TB/HIV burden countries

**J. Bacha^1,2,3^; A. Kay^2,4,5^; T. Devezin^2,5^; D. Dhillon^2,5^; R. Golin^6^; S. Dlamini^4^; N. Chidah^7^; S. Ahmedov^6^; N. Fida^8^; R.S. Wanless^3^ and A.M. Mandalakas^2,5^**

^1^Baylor College of Medicine Children´s Foundation‐Tanzania, Mbeya, Tanzania, United Republic of, ^2^Baylor College of Medicine, Houston, United States, ^3^Baylor International Pediatrics AIDS Initiative, Houston, United States, ^4^Baylor College of Medicine Children´s Foundation‐Swaziland, Mbabane, Eswatini, ^5^The Global Tuberculosis Program, Houston, United States, ^6^United States Agency for International Development, Washington, United States, ^7^Baylor College of Medicine Children´s Foundation‐Botswana, Gaborone, Botswana, ^8^USAID Regional HIV/AIDS Program, Pretoria, South Africa

While substantial attention has been given to facility‐based TB symptom screening, analyses of the subsequent TB diagnostic and treatment cascade has been remarkably limited. To inform comprehensive TB care for children and adolescents living with HIV (C/ALHIV), this study analyzed retention within the cascade among C/ALHIV in sub‐Saharan Africa.

Patient data from 2013 to 2017 were analyzed from electronic medical records and national paper registers utilized at seven BIPAI Centres of Excellences (COEs): Botswana, Eswatini, Lesotho, Malawi, Tanzania‐Mbeya, Tanzania‐Mwanza, and Uganda. Data were analyzed on C/ALHIV (ages 0‐ 18.99 years in Tanzania; ages 0 to 09.99 years in the remaining five sites) along the TB cascade. TB symptom screening, diagnosis, and treatment practices followed national and COE protocols. TB treatment outcomes were defined in accordance with WHO definitions.

Of the 22490 patients analyzed, 96% (21587/22490) completed TB symptom screening, and 58% (12466/21587) had a positive screen at one or more visit during the analytic period. After clinical evaluation, 41% (5077/12466) of C/ALHIV with a positive symptom TB screen were classified as presumptive TB, and 27% (1380/5077) of those were diagnosed with TB. TB treatment was initiated in 84.1% (1160/1380) of C/ALHIV with TB, of which 73.9% (857/1160) had favorable outcomes (cured or treatment completed). Among C/ALHIV with available data, TB was bacteriologically confirmed in 32% (216/675) of those initiating anti‐TB treatment (ATT).

Facility‐based TB symptom screening is feasible early in the TB cascade. High rates of positive TB symptom screening and presumptive TB cases, combined with drop offs in TB diagnoses, ATT initiation, and favorable outcomes underscore the need for improved retention throughout the TB cascade. Thorough follow‐up and action along the cascade are needed to ensure that presumptive TB cases receive appropriate diagnostic evaluation, workup, and treatment, particularly in the late downstream steps.

Abstract TUPDB0106‐Figure 1. Pediatric TB Cascade (Bacha) – BCM.

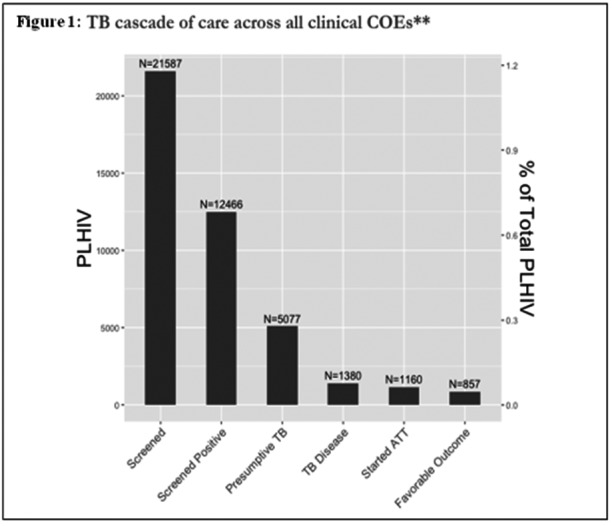

### TUPDC0101

#### Measuring perceptions of sexual risk among adolescent girls and young women taking PrEP: A new qualitative method using visual timelines in HPTN 082

**F. Scorgie^1^; N. Khoza^1^; S. Delany‐Moretlwe^1^; N. Mangxilana^2^; M. Atujuna^2^; P. Musara^3^; K. Matambanadzo^3^; L. Makhale^1^; J. Velloza^4^; C.L. Celum^4^ and The HPTN 082 Study Group**

^1^Wits RHI, University of the Witwatersrand, Faculty of Health Sciences, Johannesburg, South Africa, ^2^Desmond Tutu HIV Foundation, University of Cape Town, Cape Town, South Africa, ^3^University of Zimbabwe College of Health Sciences Clinical Trials Research Centre, Spilhaus Clinical Research Site, Harare, Zimbabwe, ^4^University of Washington, Department of Global Health, Seattle, United States

Perceived risk of HIV may motivate use of pre‐exposure prophylaxis (PrEP) but is dynamic and challenging to elicit and measure. Existing methodological approaches are often constrained by social desirability bias. We tested a novel approach to identifying when young women perceive ‘seasons of risk’ and therefore when PrEP could offer significant HIV protection.

HPTN 082 was an open label study of PrEP uptake and adherence in 16 to 65 year‐old HIV‐uninfected women in Cape Town and Johannesburg, South Africa and Harare, Zimbabwe. In a qualitative sub‐study, we used a visual method with 24 participants to obtain information about their past and current sexual relationships and perceived exposure to HIV. Participants sketched relationship ‘timelines’, and an interviewer probed about condom use, relationship power, substance use, intimate partner violence (IPV), concurrent relationships, sexually transmitted infections (STIs), and pregnancies associated with each relationship. Participants assigned a “risk score” to each relationship, based on a weighing up of factors they identified as associated with HIV risk. Interviews were audio‐recorded and transcripts were analysed using NVivo 11.

Early sexual debut, having an older partner, sex while intoxicated, and transactional sex were highly prevalent, but women seldom considered those factors in rating relationships as ‘high risk’. Women rated relationships ‘low risk’ if they were casual, if condoms were used consistently, or if sex was infrequent. Risk assessment was also based on their trust and confidence in the relationship; loving partners were rated ‘low risk’ and unhappy relationships involving conflict rated ‘high risk’, regardless of other risk factors. Women indicated that creating the timelines revealed and encouraged new reflections on aspects of their HIV risk in relationships.

Self‐assessment of risk is challenging when reflecting on intimate relationships. The “visual storytelling” approach using sexual history timelines facilitated discussions and recalibration of personal risk among young African women, who use different parameters than public health professionals to assess risk. This approach is relevant both for researchers seeking to understand the relationship between risk perception and PrEP use, and potentially for providers as a tool to support young women to assess their risk and adopt protective behaviours, including PrEP.

### TUPDC0102

#### High uptake and adherence to periconception PrEP among women in South Africa

**L.T. Matthews^1,2^; M. Jaggernath^3^; Y. Kriel^3^; C. Psaros^2^; K. O'Neil^2^; P. Smith^2^; K. Bennett^4^; C. Hendrix^5^; J.E. Haberer^2^; J.M. Baeten^6^; K. Wirth^7^; D.R. Bangsberg^8^ and J.A. Smit^3^**

^1^University of Alabama at Birmingham, Infectious Disease, Birmingham, United States, ^2^Massachusetts General Hospital, Boston, United States, ^3^MatCH Research Unit ^MRU^, University of the Witwatersrand, Durban, South Africa, ^4^Bennett Statistical Consulting, Ballston Lake, United States, ^5^Johns Hopkins School of Medicine, Baltimore, United States, ^6^University of Washington, Seattle, United States, ^7^Harvard T.H. Chan School of Public Health, Boston, United States, ^8^Oregon Health and Science University ^OHSU^, Portland, United States

Women who plan to conceive while exposed to HIV need strategies to mitigate HIV acquisition risks. We are conducting a longitudinal study in Durban, South Africa to evaluate use of TDF/FTC as PrEP among HIV‐exposed women planning for pregnancy.

We enroll HIV‐uninfected women aged 18 to 85 years with personal or partner plans for pregnancy in the next year, not using long‐acting contraception, and with a stable partner living with HIV or of unknown‐serostatus. Safer conception counseling occurs at each post‐enrollment study visit and PrEP is offered along with quarterly adherence counseling. We follow women for one year; those who become pregnant are followed through pregnancy. The primary objective is to evaluate periconception and pregnancy PrEP uptake and adherence. Adherence is defined as the number of electronic pill cap openings divided by number of days of expected PrEP use. Women provide blood for plasma tenofovir levels quarterly. We present data for the first 147 participants including adherence data for the first 52 participants completing 3‐month follow‐up.

Between October 2017 and December 2018 we enrolled 147 women with median age 24 (range 18 to 85) years and 146 (99%) identifying as black South African. Partner HIV‐serostatus was unknown by 96%. Among 135 women completing safer conception counseling, nearly two‐thirds (N = 86) chose to initiate PrEP. At the time of analysis, 52 had completed the 3‐month follow‐up visit with adherence data. During these first months, mean weekly adherence was 71% (95% CI: 66 to 67%). Nearly 50% of participants achieved > 80% adherence; 37% had 50 to 00% adherence, and 15% averaged < 50% adherence to daily PrEP. Among 40 PrEP‐initiators providing plasma at the 3‐month visit, 45% had detectable tenofovir; 38% had levels associated with daily dosing (>40 ng/mL).

Among women at‐risk for HIV acquisition, and planning for pregnancy, most choose PrEP as a safer conception strategy. Adherence is high with nearly one‐half with detectable tenofovir. Ongoing mixed‐methods analysis will explore how to refine adherence support to optimize adherence for those women who want, but struggle, to use PrEP to safely achieve reproductive goals. These data indicate high demand for and acceptability of periconception PrEP in South Africa.

### TUPDC0103

#### Safer conception services: a model to support progress towards 90‐90‐90 goals for men and women with horizontal and vertical HIV transmission risks

**N. Davies^1^; S. Mullick^2^; N. Naidoo^2^ and S. Schwartz^3,4^**

^1^Wits RHI, Health Systems Strengthening, Johannesburg, South Africa, ^2^Wits RHI, Implementation Science, Johannesburg, South Africa, ^3^Wits RHI, Johannesburg, South Africa, ^4^Johns Hopkins Bloomberg School of Public Health, Baltimore, United States

Safer conception services have the potential to support HIV treatment, prevention and elimination‐of‐mother‐to‐child (EMTCT) targets, however implementation remains limited. We explored a safer conception service through a 90‐90‐90 lens to assess potential for impact and scale‐up.

Between June 2015‐April 2017, a safer conception demonstration project was implemented in a high‐volume, public sector primary healthcare clinic in Johannesburg, South Africa. HIV‐affected individuals and couples desiring pregnancy enrolled in the service and received a comprehensive package of care including: HIV testing, ART initiation/optimisation, viral load monitoring and other interventions to minimise peri‐conception horizontal and vertical HIV transmission risks. Clinical records, laboratory results and structured interviews were used to establish client achievement of 90‐90‐90 targets.

Overall, 692 individuals enrolled, 454 women and 238 men. Of the 462 couples represented, 230 enrolled as dyads and 232 as unaccompanied individuals. Of these couples, 225 (49%) were HIV seroconcordant, 159 (34%) were serodifferent and 78 (17%) had one serounknown partner. Overall, 93%, 98% and 87% of HIV‐positive individuals achieved the 1^st^,2^nd^ and 3^rd^ 90s respectively (figure1).

Out of the those not on ART at enrolment, 85% (80% of men and 91% of women) initiated treatment through the service. Out of 120 recorded pregnancies, 72 resulted in live births with 63 HIV‐exposed infants. No vertical or horizontal transmissions were observed.

Females attending this safer conception service achieved all three 90s, boding well for EMTCT outcomes. Furthermore, although male partners achieved only the second 90, linkage to ART initiation was good and the clear progress achieved towards 90‐90‐90 may indicate that safer conception services represent a promising intervention to improve cascade outcomes for heterosexual men. Overall, this service performed well compared to South Africa's public sector programme, narrowly missing the 90‐90‐90 targets for the HIV‐positive partners reached. Safer conception scale‐up could support greater progress towards 90‐90‐90 targets.

Abstract TUPDC0103‐Figure 1. 90‐90‐90 outcomes achieved in a safer conception service cohort in Johannesburg.

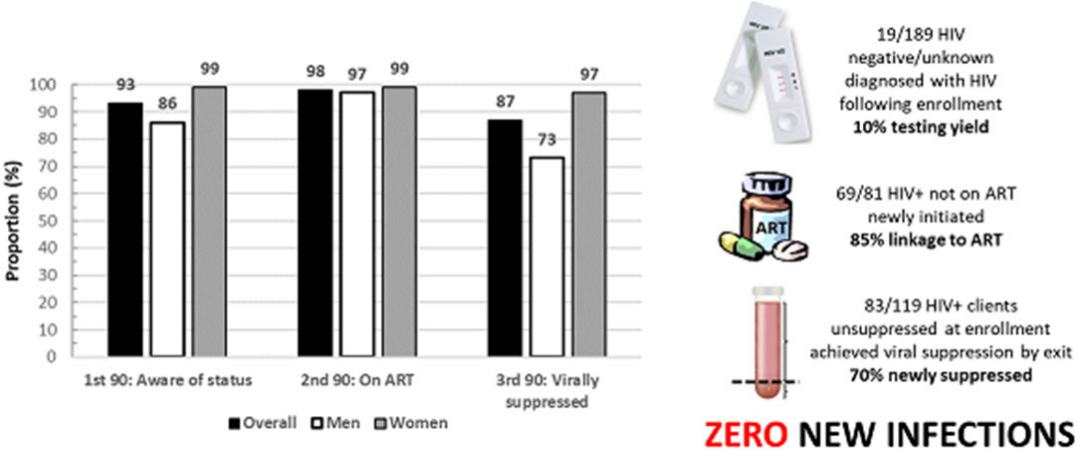

### TUPDC0104

#### Prevalence of HIV and other sexually transmitted infections among female sex workers in Moscow, Russia: results from a community‐based, cross‐sectional study using respondent driven sampling methodology

**D. Pataut^1^; A. Bernier^1^; N. Volkova^2^; I. Pchelin^2^; O. Maximov^3^; T. Rumyantseva^1,4^; C. Malet^1^; E. Derriennic^1^; J. Bouscaillou^1^; Y. Kyburz^1^; A. Guschin^4^; N. Luhmann^1^ and E. Wisse^1^**

^1^Médecins du Monde ^MdM^, Paris, France, ^2^Shagui, Moscow, Russian Federation, ^3^Médecins du Monde ^MdM^, Moscow, Russian Federation, ^4^Central Research Institute for Epidemiology, Moscow, Russian Federation

In Russia, it is estimated by the Ministry of health that 0,8 million people are living with HIV, and that 85.800 new infections occurred in 2017. Despite female sex workers (FSW) being known as a key population for HIV and other sexually transmitted infections (STI), data on HIV/STIs prevalence in this group are scarce in Russia. The objective of this study was to estimate HIV and other STIs prevalence among FSW in city of Moscow and Moscow region.

A cross‐sectional study was implemented by an international non‐governmental organization (NGO), a Russian NGO and a Russian research institute using the respondent driven sampling methodology. The recruitment took place between October 2017 and July 2018. Data collection included a face‐to‐face questionnaire, HIV and syphilis rapid tests, throat swab and self‐collected vaginal and anal swabs for the detection of 4 other STIs (*Neisseria gonorrhoeae, Chlamydia trachomatis, Trichomonas vaginalis* and *Mycoplasma genitalium*). Statistical analysis was conducted using weights based on the RDS‐II estimator. Factors associated with HIV infection were identified using a weighted multivariate logistic regression.

In total, 385 participants were included in the analysis, including 18 seeds. Among them, 53.5% worked as indoor FSW and 46.5% as outdoor FSW. The median age was 30.0 years. Regarding ethnic origin, 73.2% were Russian, 19.5% came from Former Soviet Union States and 5.7% were African. The median age of sex work debut was 23 years and the median weekly number of clients was 8. In the previous 30 days, 36.9% declared unsystematic condom use with clients. Weighted HIV prevalence was 3.1% (95% CI: 1.5%‐7.0%). Other STI prevalence was comprised between 4.1% [2.2%‐8.0%] (*Neisseria gonorrhoea*) and 14.9% [10.5%‐21.0%] (*Mycoplasma genitalium*). Factors associated with HIV infection were: being 25 years and less (OR = 0.06; 95% CI: 0.00 to 0.77, *p* = 0.03); coming from Former Soviet Union States (4.55 [1.12 to 28.50], *p* = 0.03) or Sub‐saharan Africa (24.76 [2.51 to 143.81], *p* = 0.006); and having taken drugs in the previous 6 months (7.84 [1.42 to 23.20], *p* = 0.01).

These results show high HIV/STIs prevalence among FSW in Moscow region, highlighting the need for better access to SW‐friendly prevention and care services in Russia.

### TUPDC0105

#### PrEP use and sexually transmitted infections are not associated longitudinally in a cohort study of young men who have sex with men in Chicago

**E. Morgan^1^; C. Dyar^1^; M. Newcomb^1^; R. D'Aquila^2^ and B. Mustanski^2^**

^1^Northwestern University, Chicago, United States, ^2^Northeastern University, Chicago, United States

In the United States, rates of sexually transmitted infections (STIs) have risen steadily in recent years as has PrEP use to prevent HIV infection. Our goal was to understand longitudinal patterns in the association between PrEP user and STIs. These analyses are a key target in prevention efforts aimed at disrupting the downstream HIV infection risk.

Data were collected as part of RADAR, a cohort study of young men who have sex with men (YMSM) and transgender women (TGW; aged 16 to 69) living in Chicago. Unadjusted and adjusted longitudinal lagged regression models were utilized to assess the relationship between PrEP use and odds of rectal STI acquisition. Analyses included data from six study visits. Mediation models were also utilized to consider the potential pathway between PrEP use, condomless sex, and STI diagnosis.

Two hundred and eight‐two (24.1%) participants reported PrEP use at least once across all study visits while 374 (31.9%) participants had a positive rectal STI test at least once. In longitudinal models, no significant association was observed between PrEP use and STI diagnosis (aOR = 1.07, 95% CI: 0.63 to 3.82). This same finding was observed when comparing PrEP users to non‐users as well as when comparing consistent PrEP users to those who varied their used between study visits. In mediation models, PrEP use was significantly associated with increased likelihood of condomless anal sex at the next study visit (CAS; aOR = 1.61, 95% CI: 1.10 to 0.36), however, CAS was not associated with STI status (aOR = 0.95, 95% CI: 0.58 to 8.57). Nor was there a significant difference in relationship between PrEP and STIs when stratifying either of these analyses by race/ethnicity.

We demonstrated that, overall, PrEP use was not associated with STIs among YMSM but did observe that PrEP users were more likely report increased participation in CAS at the subsequent study visit. In the talk, theories will be explored as to why an association exists between PrEP use and CAS but not between CAS and STIs.

### TUPDC0106

#### Prospective, multicenter study to assess point prevalence, incidence and recurrence of sexually transmitted infections in men who have sex with men in Germany: BRAHMS study

**H. Streeck^1^; K. Janssen^2^; T.A. Crowell^3^; H. Jessen^4^; C. Cordes^5^; S. Scholten^6^; S. Schneeweiss^6^; N. Brockmeyer^7^; C.D. Spinner^8^; M. Bickel^9^; S. Esser^10^; J. Hartikainen^11^; A. Stoehr^12^; C. Lehmann^13^; U. Marcus^2^; J.‐J. Vehreschild^13^; A.‐L. Brillen^1^; C. Tiemann^14^; M.L. Robb^3^ and N.L. Michael^3^**

^1^Institute for HIV Research, Essen, Germany, ^2^Robert Koch Institute, Berlin, Germany, ^3^U.S. Military HIV Research Program, Bethesda, United States, ^4^Private Practice Jessen+Jessen, Berlin, Germany, ^5^Private Practice Cordes, Berlin, Germany, ^6^Private Practice Hohenstaufenring, Cologne, Germany, ^7^Walk im Ruhr ^WiR^, Bochum, Germany, ^8^Technical University of Munich, Munich, Germany, ^9^Infektiologikum Frankfurt, Frankfurt, Germany, ^10^HPSTD clinic, University Duisburg‐Essen, Essen, Germany, ^11^MVZ Diesener Str., Berlin, Germany, ^12^institute for interdisciplinary medicine, Hamburg, Germany, ^13^University of Cologne, Cologne, Germany, ^14^Laboratory Krone, Bad Salzuflen, Germany

Rates of new HIV infections in Germany are moderately declining, and knowledge on impact of novel HIV prevention methods, such as HIV pre‐exposure prophylaxis (PrEP), including its impact on sexual transmitted infections (STI) are sparse.

Here we report on a prospective, multicenter study to assess point prevalence, incidence and recurrence of STIs in 1000 men who have sex with men (MSM) at risk for HIV infection.

The BRAHMS study is a prospective study conducted at ten sites in 7 major German cities enrolling MSM at risk for HIV infection. Participants are seen every three months and systematically screened for all STIs including HIV, HAV, HBV, HCV, Gonorrhea (NG), Syphilis (TP), Chlamydia trachomatis (CT) and Mycoplasma genitalium (MG) among others. Diagnosis was performed from blood samples as well as urine, anal swab, and oropharyngeal swab specimens. Sexual behavior questionnaires are assessed at each time point.

Among participants at risk for HIV infection we found high point prevalence of sexually transmitted infections (NG: 10%, CT, 13% MG 20%, TP 5%). Overall seropositivity for TP was 20.3%, which includes participants with a positive EIA and history of previously‐treated disease. In addition, we found a point prevalence of HIV and acute HCV infection of each 0.5%. 25% of participants were positive for more than one STI. Over 90% of STI cases were asymptomatic. CT, MG and GO infections were predominantly rectal (CT:67%; MG:59%; GO:43%), while GO was also frequently pharyngeal (CT: 15%; MG: 14%; GO 43%). 68% of individuals at risk for HIV infection initiated PrEP. There were no significant differences in the point prevalence of STIs in individuals that already took PrEP before enrollment into the study and non‐PrEP users. All HIV infections were detected in non‐PrEP users.

We detected high frequencies of asymptomatic, rectal STIs in MSM but low point prevalence of previously undiagnosed HIV infection.

### TUPDD0101

#### Measuring anticipated sex‐work stigma: Scale validation and association with HIV and non‐HIV service utilization

**C. Stewart^1^; E. Oga^2^; J. Kraemer^3^; D. Kuria^4^; M. Stockton^5^ and L. Nyblade^6^**

^1^RTI International, Research Triangle Park, United States, ^2^RTI International, Rockville, United States, ^3^Georgetown University, Department of Health Systems Administration, Washington, United States, ^4^Independent Consultant, Nairobi, Kenya, ^5^University of North Carolina at Chapel Hill, Gillings School of Global Public Health, Department of Epidemiology, Chapel Hill, United States, ^6^RTI International, Washington, United States

Stigma research has largely focused on HIV‐related stigma; however, the importance of measuring and addressing stigmas that intersect with HIV is growing. Anticipated HIV stigma has been linked with delay of HIV testing and avoidance of health services but there is limited research on the association between anticipated sex‐work stigma and healthcare utilization. This abstract presents results of analysis to validate a scale for anticipated sex‐work stigma and examine associations between anticipated stigma and HIV and non‐HIV service utilization among sex workers in Kenya.

Using a pool of 23 items on anticipated sex‐work stigma, we tested items with data from a cross‐sectional survey of 729 sex workers (232 males, 497 females, 172 HIV‐positive), collected in January‐February 2015 in four sites (Nairobi, Kitui, Homa Bay, Busia). Confirmatory factor analysis (CFA) was used to identify the best scale and logistic regression was used to assess associations between anticipated sex‐work stigma and HIV and non‐HIV service utilization in the past 12 months.

CFA supported a 6‐factor anticipated sex‐work stigma scale (chi‐square *p* < 0.001; root mean square error of approximation [RMSEA]=0.08; standardized root mean square residual [SRMR] = 0.04); and comparative fit index [CFI]=0.92. The final 18‐item scale included 6 subscales. The scale demonstrated excellent internal consistency (α=0.93). Higher scores on certain subscales of anticipated sex‐work‐stigma scores were associated with avoidance or delay of HIV testing: healthcare workers (aOR: 1.09 [1.01 to 1.35]) and police (aOR: 1.11 [1.02 to 2.21]). Anticipated stigma from healthcare workers was also associated with avoidance or delay of HIV treatment (aOR:1.17 [1.01 to 1.35]). Higher scores on all subscales were associated with avoidance or delay of non‐HIV health services.

Abstract TUPDD0101‐Table 1. Association stigma and delay or avoidance of HIV and non‐HIV services

**Table:** 

Anticipated stigma subscales^a^	Avoidance/delay HIV testing (n = 729), adjusted odds ratios (95% CI)^b^	Avoidance/delay HIV treatment (n = 245) adjusted odds ratios (95% CI)^b^	Avoidance/delay of non‐HIV health Services (n = 729), adjusted odds ratios (95% CI)^b^
Healthcare workers	1.09 (1.01 to 1.19)^c^	1.17 (1.01 to 1.35)^c^	1.09 (1.03 to 3.16) c
Family	1.03 (0.94 to 4.05)	0.99 (0.84 to 4.17)	1.12 (1.06 to 6.18) c
Community and friends	1.00 (0.95 to 5.05)	1.08 (0.99 to 9.17)	1.07 (1.04 to 4.10) c
Police	1.11 (1.02 to 2.21)^c^	1.05 (0.91 to 1.23)	1.10 (1.02 to 2.19) c
Social exclusion	1.04 (0.99 to 9.09)	1.01 (0.95 to 5.08)	1.06 (1.03 to 3.09) c
Physical violence	1.02 (0.96 to 6.09)	1.01 (0.92 to 2.11)	1.06 (1.02 to 2.11) c

^a^Anticipated stigma subscales modeled as continuous variables; ^b^adjusted for weekly income, city of residence and presence of depression; ^c^significant at α=0.05.

Sex‐work stigma is a recognized, yet understudied, barrier to effective HIV responses. This validated measure of sex‐work stigma can be used in conjunction with existing HIV stigma measures to explore intersectional stigma as a barrier to HIV testing, prevention, and treatment.

### TUPDD0102

#### Mapping key population hotspots in Nigeria for targeted HIV program planning

**J. Lo^1^; S. Nwafor^2^; A. Mitchell^1^; O. Ogbeh^2^; V. Sebastian^2^; A. McIntyre^4^; A. Schwitters^3^; V. Adamu^3^; J. Gwamna^3^; M. Swaminathan^5^ and M. Charurat^1^**

^1^University of Maryland School of Medicine, Institute of Human Virology, Baltimore, United States, ^2^University of Maryland Baltimore, Maryland Global Initiative Corporation, Abuja, Nigeria, ^3^Centers for Disease Control and Prevention, Division of Global HIV and TB, Abuja, Nigeria, ^4^Centers for Disease Control and Prevention, Division of Global HIV and TB, Atlanta, United States, ^5^Centers for Disease Control and Prevention, Abuja, Nigeria

Nigeria has the second highest HIV burden globally. In 2017, UNAIDS demonstrated high prevalence among Nigeria's key populations (KP): 14.4% among female sex workers (FSW), 23.0% among men who have sex with men (MSM), and 3.4% among people who inject drugs (PWID). Reliable and accurate mapping of KP hotspots is necessary for strategic placement of services and allocation of limited resources for targeted interventions.

During August 2018, 261 trained staff from 36 KP‐led community‐based organizations in seven U.S. President's Emergency Plan for AIDS Relief (PEPFAR) priority states mapped and validated hotspots identified during a recent formative assessment. Geographic coordinates, peak times of activity, and estimated number of KP individuals were recorded.

Of the 13,862 KP hotspots mapped, 69.1% were FSW, 11.3% were MSM, and 19.6% were PWID. Although generally clustered around the city centers, many hotspots were in less populated areas of each state. We found far fewer MSM hotspots compared to FSW and PWID hotspots. The highest number of hotspots were observed in Lagos, Cross River, and Rivers states.

We identified many new and previously undocumented KP hotspots. The low number of MSM hotspots relative to FSW and PWID hotspots might be attributed to the Same Sex Marriage (Prohibition) Act, which has reduced the visibility of MSM activities. Engaging local KP throughout this activity increased access to previously undocumented hotspots and strengthened partnerships for future collaboration with HIV programs. The information obtained from this exercise will be used by the Government of Nigeria to design more strategically located and appropriately scaled KP‐specific interventions.

Abstract TUPDD0102‐Table 1. Hotspots identified in seven U.S. President's Emergency Plan for AIDS Relief priority states in Nigeria by key population

**Table:** 

	FSW	MSM	PWID	Total
Akwa ibom	708	276	312	1296
Benue	1098	265	351	1714
Cross rivers	1782	268	616	2666
Federal capital territory	977	116	105	1198
Lagos	2599	131	239	2969
Nasarawa	985	242	313	1540
Rivers	1423	273	783	2479
Total	9572	1571	2719	13862

Absrtract TUPDD0102‐Figure 1. Distribution of key population hotspots by local government area in Nigeria.

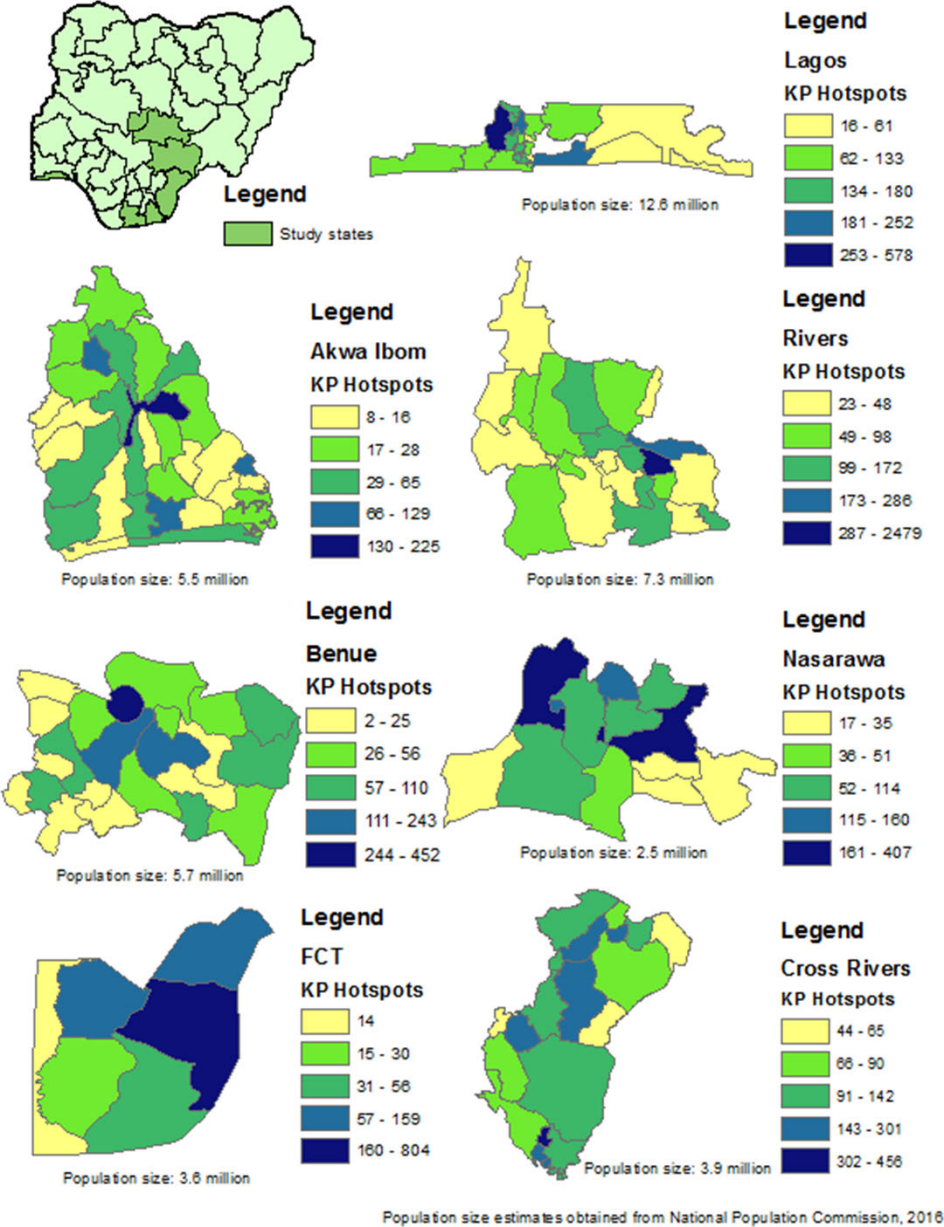

## TUPDD0103

### The M‐Spot 2 Study: Feasibility of measuring viral load longitudinally using home‐collected dried blood spot specimens of high‐risk MSM living with HIV

**R. Teran^1^; M.E. Sobieszczyk^2^; M.A. Chiasson^1,2^; A.C. Uhlemann^2^; J. Weidler^2^; J.G. Shah^2^; J.Y. Chang^2^ and S. Hirshfield^3^**

^1^Columbia University Mailman School of Public Health, Department of Epidemiology, New York, United States, ^2^Columbia University Irving Medical Center, Department of Medicine, Division of Infectious Diseases, New York, United States, ^3^SUNY Downstate Medical Center, Department of Medicine, Brooklyn, United States

The “Undetectable=Untransmittable” campaign emphasizes the need for individuals living with HIV to reach and maintain viral suppression to prevent new infections. Emerging prevention strategies to monitor viral load (VL) are needed for those disengaged from care or sub‐optimally adherent to antiretroviral therapy (ART). eHealth studies with HIV‐positive MSM report high acceptability of self‐collecting biological specimens, including dried blood spots (DBS) to measure VL; however, little information exists on longitudinal monitoring of VL. We report longitudinal research‐based VL test results from home‐collected DBS specimens among HIV‐positive MSM recruited online.

In 2018, US HIV‐positive MSM (n = 78) identified as having detectable VL (DVL) were invited to participate in a study to measure VL longitudinally via self‐collected DBS specimens. Consenting participants received DBS kits at baseline and 3‐month follow‐up with instructions to collect and mail specimens to a research laboratory. RNA was extracted using the Qiagen RNeasy kit. TaqMan‐based Real‐time Quantitative PCR research assay was used to quantify VL. The lower limit of quantification (LLQ) was estimated at 500 copies/mL. Results were reported as undetectable (UVL), below the LLQ, or a quantitative result if VL > 500 copies/mL.

Of 56 consenting participants, 68% were White, 14% Black, and 16% Hispanic. Median age was 42; 51/56 (91%) returned specimens for testing at baseline and 43/51 (84%) at 3‐month follow‐up. At baseline, 2 specimens were below the LLQ, and 6 had a DVL (median = 1475; range = 603 to 2867 copies/mL); at 3‐month follow‐up, 4 specimens were below the LLQ, and 3 had DVL (median = 1804; range = 1245 to 14709 copies/mL). While 74% of men had UVL specimens at both time points, the remainder had VL fluctuations from baseline to follow‐up (DVL to UVL = 4; UVL to DVL = 5; DVL to DVL = 2).

Our longitudinal study of HIV‐positive MSM with a past DVL showed that at‐home DBS collection and research‐lab monitoring of VL is feasible. Fluctuating viremia over a 3‐month period was identified in a subset of participants. Findings signal interest in DBS home collection by HIV‐positive MSM with sub‐optimal ART adherence. This approach may improve research data collection and potentially provide a complementary VL monitoring approach in clinical care to increase the proportion of MSM with UVL.

## TUPDD0104

### Developing and validating a model for risk‐based differentiation of HIV prevention and testing services for female sex workers: Experiences from Maharashtra, India

**J. Kirubakaran^1^; L. Gabhane^2^; P. Deoraj^2^; M. Setia^3^; P. Goswami^1^; S. Panyam^4^; J. Patil^5^; M.R. Parthasarathy^1^; M. Doddamane^6^; G.S. Shreenivas^1^; J. Baishya^7^ and B. George^1^**

^1^FHI 360, New Delhi, India, ^2^Maharashtra State AIDS Control Society, Mumbai, India, ^3^Consultant, Mumbai, India, ^4^Consultant, Hyderabad, India, ^5^Technical Support Unit of Maharashtra, Mumbai, India, ^6^FHI 360, Mumbai, India, ^7^USAID India, New Delhi, India

India's national AIDS control program delivers HIV services for key populations through targeted interventions (TI). Optimum use of limited resources demands prioritization to maximize impact. We aimed to develop and validate a model to prioritize female sex workers (FSWs), based on risk/vulnerability characteristics, for HIV diagnosis and risk‐reduction services.

We analyzed routinely collected program data covering demographics, risk behavior, vulnerabilities, and biological outcomes from three FSW TIs in Maharashtra from April 2016 to March 2018. We linked each individual's behavioral data from the previous quarter to the HIV test results of the reporting period, generating 16,228 data points. Penalized and regular logistic regression analyses were used to identify any associations between the odds of HIV positivity and prospective explanatory variables including age, years of FSW activity, years of TI association, number of sex acts in the past week, condom use, and history of sexually transmitted infections. We used receiver operating characteristic curve analysis and the Youden's index method for each numerical variable and optimal cutoff, respectively. Weighted scores for each indicator were computed using dominance analysis. The cutoff with the best sensitivity for HIV positivity in the high‐risk group and best specificity for HIV negativity in the low‐risk group were identified. After the model was completed, it was applied prospectively in two of the above‐mentioned FSW TIs to study its efficiency for segmenting FSWs into high, medium, and low priority for HIV testing and risk‐reduction services.

Among 2,239 FSWs categorized into priority groups, 684 (30.5 percent) were considered high‐priority. Of the 11 cases of HIV detected, all were from this high‐priority group (Table 1).

Abstract TUPDD0104‐Table 1. HIV testing and detection by priority group (April to September 2018)

**Table:** 

Female sex workers	High‐priority	Medium‐priority	Low‐priority	Total
Number (percentage) in priority group	684 (30.5%)	1,068 (47.7%)	487 (21.8%)	2,239 (100%)
Number tested	573	840	311	1724
Number of HIV‐positive cases	11	0	0	11

The model demonstrated effective and precise categorization of FSWs at increased HIV risk, corroborating the need for intensified interventions among the high‐priority group. This would include augmenting the frequency and intensity of high‐threshold prevention services through differentiated strategies within the concentrated HIV epidemic in India.

## TUPDD0105

### Receipt of medication‐assisted treatment halves the risk of HIV‐1 RNA viral load rebound for HIV‐positive women who use illicit drugs

**J. Adams^1^; B. Marshall^1^; E. Nosova^2^; S. Nolan^2^; R. Barrios^3^ and M.‐J. Milloy^2,4^**

^1^Brown University, Department of Epidemiology, Providence, United States, ^2^University of British Columbia, BC Centre on Substance Use, Vancouver, Canada, ^3^University of British Columbia, BC Centre for Excellence in HIV/AIDS, Vancouver, Canada, ^4^University of British Columbia, Department of Medicine, Vancouver, Canada

Women living with HIV are less likely to achieve optimal antiretroviral adherence and retain HIV‐1 RNA viral load non‐detectability compared to men. Risk factors for viral rebound may differ by sex. This study's objective was to evaluate the impact of sociodemographic, behavioral, and clinical factors on the hazard of viral rebound after achieving viral suppression for a cohort of women living with HIV who use illicit drugs.

We used longitudinal data from 2005 to 2017 for women living with HIV who use illicit drugs enrolled in the ACCESS study, a prospective cohort with systematic HIV viral load monitoring. Women were included if they achieved viral suppression (i.e., HIV‐1 RNA viral load < 50 copies/mL) following antiretroviral therapy initiation and had more than one study interview. Sociodemographic as well as substance use, addiction treatment, and HIV clinical factors were evaluated as predictors of viral rebound (i.e., HIV viral load > 1000 copies/mL). Cox regression using a recurrent events framework, time‐varying covariates, and robust standard errors were used.

Of the 206 women included, nearly half (46%) are Indigenous, 4.4% are transgender women, and a quarter self‐identified as a sexual minority. Over the 12‐year period, 40% (n = 83) experienced at least one virologic failure, accumulating a total of 115 viral rebound events. In adjusted analysis, the only factor associated with viral rebound was receipt of medication assisted treatment (MAT) in the past six months. Figure1 compares probability of viral rebound by MAT status. Women who received MAT had half (adjusted hazard ratio: 0.49, 95% confidence limits: 0.28 to 0.88) the hazard of viral rebound compared to women who had not received MAT.

This study provides additional evidence that MAT can improve HIV treatment outcomes among women. Efforts to improve access to and retention within MAT programs may improve rates of viral suppression for HIV‐infected women who use illicit drugs.

Abstract TUPDD0105‐Figure 1. Kaplan‐Meier curve of time to first viral rebound following suppression by MAT status.

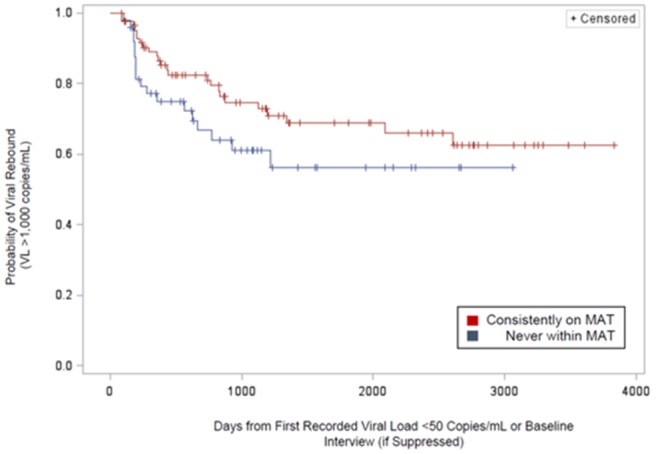

## TUPDD0106

### Police harassment and alcohol and drug abuse is associated with poorer 6‐month retention among transgender women starting ART in a clinical trial in Argentina

**I. Aristegui^1,2^; P. Radusky^1,3^; V. Zalazar^1^; C. Frola^1,4^; N. Cardozo^1,5,6^; M. Duarte^1,5^; S. Fabian^1,7^; A. Gun^1^; P. Cahn^1^ and O. Sued^1^**

^1^Fundacion Huesped, Research Department, Buenos Aires, Argentina, ^2^Universidad de Palermo, Buenos Aires, Argentina, ^3^Universidad de Buenos Aires, Buenos Aires, Argentina, ^4^Hospital Juan A. Fernández, Infectious Diseases Unit, Buenos Aires, Argentina, ^5^Asociación de Travestis, Transexuales y Transgéneros de Argentina ^A.T.T.T.A.^, Buenos Aires, Argentina, ^6^REDLACTRANS, Buenos Aires, Argentina, ^7^Asociación Civil Gondolin, Buenos Aires, Argentina

In Argentina, HIV morbidity and mortality is still high among transgender women (TGW). Previous studies describe that syndemic factors act as barriers to access health services. Our objective was to identify psycho‐social factors associated with retention in care in treatment‐naive TGW in a clinical trial.

Naive TGWs were offered to start ART in a trans‐sensitive health‐care service. All participants initiated Dolutegravir plus TDF‐FTC. Psychosocial interviews were applied longitudinally. The questionnaire collected data regarding socio‐demographic characteristics, alcohol and drug use, depression, anxiety, personality traits, HIV‐related stigma, sexual behavior, interactions with police, healthcare access, housing, education, work, and experiences of stigma and discrimination in these settings. Relevant variables in this analysis were: Retention (completing assessment week‐24, +/‐ 1 month), gender identity‐related police harassment (detained longer, threatened, beaten or sexually abused by policemen last year), subscale alcohol‐related problems (AUDIT), substantial and severe drug abuse (DAST score > 6).

The sample included 61 TGW, median age 28 (IQR 25‐ 32), 19.7% had less than 200 CD4 at screening. At week 24, 82% (n = 50) were retained in treatment and 77.6% were virologically suppressed. Baseline characteristics showed high levels of vulnerability: 60.7% less than high‐school education, 53.3% unstable housing, 29.5% foreign born, 77% sex work, 65.6% regular drug use and 32.8% suffered sexual abuse in the last year. Moreover, 31% reported being arrested and 18% experienced police harassment last year. The only factors significantly associated with failure of retention but not with virological failure were: police harassment (OR = 0.16, 95%CI 0.03 to 0.71), alcohol‐related problems (t(46) = ‐2.43, *p* = 0.019), drug/alcohol consumption with clients during sexual encounters (t(56) = ‐3.26, *p* = 0.002), and drug abuse (OR = 0.07, 95% CI 0.01 to 0.76).

Trans‐competent health care provides a safe space for vulnerable TGW to access health services and may counteract the negative effect of previous discriminatory experiences in health settings. Nevertheless, the contextual risk environment where TGW live, work and socialize, with high exposure to violence, drugs, and discrimination and stigma may jeopardize ART expansion in this population. Future clinical research and interventions targeting this group should consider self‐empowerment, drug harm‐reduction and address structural‐discrimination of TGW in order to improve their retention in HIV healthcare.

## TUPDD0201

### Exploring the mediating role of tobacco use in the relationship between intersectional stigma and HIV clinical outcomes among women living with HIV in Canada

**C. Logie^1^; Y. Wang^1^; M. Kazemi^2^; B. Gagnier^2^; T. Conway^2^; S. Islam^2^; M. Lee^3^; K. Beaver^2^; A. Kaida^3^; D.P. Alexandra^4^ and M. Loutfy^2^**

^1^University of Toronto, Toronto, Canada, ^2^Women's College Research Institute, Toronto, Canada, ^3^Simon Fraser University, Vancouver, Canada, ^4^McGill University, Montreal, Canada

Intersectional stigma harms women living with HIV's (WLHIV) health and wellbeing. Coping strategies such as tobacco use may reduce stigma‐related stress. Yet there are serious adverse health effects associated with long term tobacco use among WLHIV. Limited research has explored intersectional stigma and tobacco use. We examined coping strategies (tobacco use, resilience) as mediators of the relationship between intersectional stigma (HIV‐related stigma, gender discrimination, racial discrimination) and HIV clinical outcomes among WLHIV in Canada.

We analyzed baseline survey data from a national cohort study with WLHIV in three Canadian provinces. Structural equation modeling (SEM) using weighted least squares estimation methods was used to test the direct effects of intersectional forms of stigma (HIV‐related stigma, gender discrimination, racial discrimination) on HIV‐related clinical outcomes (>90% ART adherence, CD4 count > 200 cells/mm3, undetectable viral load), and the indirect effects via resilience and current tobacco use, adjusting for socio‐demographic factors.

Among 1422 participants (median age: 42.5 years; IQR = 35 to 50), most were women of colour (29.40% African, Caribbean and Black; 22.36% Indigenous; 7.17% other ethnicities; 41.07% white). Over one‐third (43.66%; n = 616) were current tobacco users, 12.05% (n = 170) formerly used tobacco, and 44.29% (n = 624) never used tobacco. SEM results suggest that racial discrimination had a direct effect on ART adherence (B = ‐0.215, *p* < 0.001: direct effect; B = 0.046, *p* < 0.01: indirect effect), resilience partially mediated this relationship. Current tobacco use fully mediated the relationship between gender discrimination and lower CD4 count (B = ‐0.061, *p* < 0.01) and detectable viral load (B = ‐0.055, *p* < 0.01). Current tobacco use partially mediated the relationship between gender discrimination and ART adherence (B = ‐0.044, *p* < 0.01). Resilience fully mediated the relationship between HIV‐related stigma (B = 0.040, *p* < 0.01) and racial discrimination (B = 0.027, *p* < 0.01) with CD4 count, and between HIV‐related stigma (B = 0.021, *p* < 0.05) and racial discrimination (B = 0.014, *p* < 0.05) with viral load. Fit indices suggest good model fit (CFI = 0.937; RMSEA = 0.048 [90% CI: 0.43 to 0.069]; SRMR = 0.030).

Intersectional stigma based on HIV, race and gender contributed to increased tobacco use and reduced resilience, that in turn contributed to lower CD4 count and detectable viral load. There is an urgent need for intersectional stigma reduction interventions and strategies to support WLHIV who use tobacco as an intersectional stigma coping strategy.

## TUPDD0202

### Implementation of social capital theory and process to strengthen state‐level combination prevention outcomes in 12 states in Mexico

**A. Luna^1,2,3^; C. Coria^4^ and K. Morrison^4,5,6^**

^1^MoKexteya, Direction, Mexico City, Mexico, ^2^Instituto Nacional de Salud Publica, Diplomado Sida, Cuernavaca, Mexico, ^3^Consorcio de Investigación sobre VIH SIDA TB CISIDAT, Research and Training, Cuernavaca, Mexico, ^4^MoKexteya, Mexico City, Mexico, ^5^Consorcio de Investigación sobre VIH SIDA TB CISIDAT, Cuernavaca, Mexico, ^6^Instituto Nacional de Salud Publica, Diplomado Sida, Cuernvaca, Mexico

Prevention, especially in marginalised groups, KPs, has not kept up with the advances in treatment. Combination prevention provides a frame within to work at state level. While stigma and discrimination are often cited as the principle obstacle to effective prevention, little concentrated emphasis has been put on addressing internal stigma. Social capital theory (helping excluded persons and groups increase sense of belonging, trust and influence) provides a social process approach to addressing the effects of internal stigma and provide for multisectoral comprehensive response to prevention.

This combination prevention project involved working with 3 key sectors (community, health service providers and government representatives or consumers, producers, and overseers) using three forms of networking (bonding, bridging and linking). It was undertaken in 12 states : in each state., 30 representatives of the three sectors participated cooperatively in a 3‐step process: 1) diagnosis and planning, 2) collaborative and complementary implementation, 3) collective assessment. Each state addressed 5 aspects of combination prevention (structural prevention was broken down into quality of services; polices and procedures; and community mobilization and development).

The project resulted in a collective comprehensive and cohesive approach to combination prevention in all states with notable advances in understanding and addressing needs of key populations. Yearly assessment meetings at the end of a 10‐month process as well as The results of an external qualitative assessment of 10 states showed significant progress in collaborative action, uptake in services, and in some states an increase in state HIV/STI prevention budgets. It showed the need for more emphasis on social cohesion for trans persons and sex workers. Process has been adjusted and now focusing on improved health care services for trans in the states as part of the integrated approach to HIV prevention.

The overall process, refined over 5 years, provides a systematic process approach to building social capital as a key part of the response to effective HIV prevention while addressing issues of stigma, discrimination and internal sigma in a collective multisectoral manner that greatly impacted the perception to and use of differentiated health services for key affected populations.

## TUPDD0203

### Frustrated patients, frustrated providers: A comprehensive integrated conceptual model to explain why patients delay, decline, or discontinue HIV medication and strategies to boost sustained HIV viral suppression in populations at‐risk

**M. Gwadz^1,2^; C. Cleland^3^; B. Martinez^1^; A. Ritchie^1^; R. Freeman^1^; N. Leonard^1^; Y. Allen^1^; N. Bobb^1^; D. Kennedy^1^; D. Sherpa^1^; D. Jonas^1^; A. Kutnick^1^ and Heart to Heart 2 Collaborative Research Team**

^1^New York University Silver School of Social Work, New York, United States, ^2^New York University, Center for Drug Use and HIV Research ^CDUHR^, New York, United States, ^3^New York University School of Medicine, Department of Population Health, New York, United States

At least 40% of persons living with HIV (PLWH) in the U.S. do not sustain HIV viral suppression (VS), with the lowest rates among low‐socioeconomic status and African American/Black and Hispanic populations. This presentation will synthesize a set of mixed methods studies on the complex multi‐level barriers this subgroup experiences to VS, and facilitators of VS, *from their own perspectives*, and describe promising intervention approaches to increase rates of VS in this population at‐risk.

We formed a multi‐disciplinary interpretive community and, guided by an ecological model, the theory of triadic influence, and critical race theory, compared/contrasted, critically evaluated, interpreted, and synthesized findings from studies we conducted with this population from 2008 to 2018. Studies focused on diverse topics including effects of concentrated poverty, experiences in institutional settings, the history of HIV, emotions, “forgetting” medication, substance use/mental health, reasons for stopping/starting medications, resilience, selling medication, and efficacious culturally salient behavioral interventions to increase VS. A comprehensive integrated conceptual model of intersecting barriers to and facilitators of VS was developed, along with efficacious or promising intervention approaches to modify or address barriers, at multiple levels of influence. Emphasis was placed on under‐studied barriers, and facilitators, resilience, and potential interventions.

Barriers to and facilitators of VS were articulated at all levels of the ecological model, highlighting their complexity. Poverty was central at all levels of the ecology, and structural racism was a second major contextual influence. The history of the HIV epidemic was potent, where the collective memory of ineffective AZT monotherapy contributed to social norms and individual beliefs regarding medical distrust, counter‐narratives about the origins of/cures for HIV, and fear of medication, producing emotions leading to medication avoidance. Incarceration among men caused trauma and disrupted care, and contributed to intersectional stigma. Substance use problems were pervasive. Promising intervention approaches included those made culturally salient by addressing these barriers. An example of an efficacious behavioral intervention to boost VS will be provided, along with other promising intervention approaches that emerged from the analysis.

This comprehensive integrated conceptual model provides insights into intervention approaches to increase rates of VS among populations at‐risk.

## TUPDD0204

### Estimating plausible ranges on the scale of implementation for evidence‐based HIV/AIDS interventions in the US

**E. Krebs^1^; B. Enns^1^; X. Zang^1^; C. Del Rio^2^; J. Dombrowski^3^; C. Behrends^4^; D.J. Feaster^5^; R. Granich^6^; B. Marshall^7^; S.H. Mehta^8^; L. Metsch^9^; B.R. Schackman^4^; S. Shoptaw^10^; S.A. Strathdee^11^; B. Nosyk^1^ and on behalf of the Localized Economic Modeling Study Group**

^1^BC Centre for Excellence in HIV/AIDS, Vancouver, Canada, ^2^Emory University, Rollins School of Public Health, Atlanta, United States, ^3^University of Washington, Seattle, United States, ^4^Weill Cornell Medical College, New York, United States, ^5^University of Miami, Miami, United States, ^6^International Association of Providers of AIDS Care, Washington, United States, ^7^Brown School of Public Health, Providence, United States, ^8^Johns Hopkins Bloomberg School of Public Health, Baltimore, United States, ^9^Columbia University Mailman School of Public Health, New York, United States, ^10^University of California Los Angeles, Los Angeles, United States, ^11^University of California San Diego, San Diego, United States

Simulation modeling plays a critical role in priority setting for HIV treatment and prevention interventions; however, interventions may vary substantially in their ability to deliver value at different levels of scale and in different epidemiological contexts. To inform a U.S. six‐city microepidemic HIV transmission model, we executed a targeted literature review to identify plausible ranges of the scale of delivery for a set of evidence‐based interventions for the treatment and prevention of HIV/AIDS among adults.

We identified 14 evidence‐based interventions from the US CDC's Compendium of Evidence‐Based Interventions and Best Practices for HIV Prevention and from the recently published literature, ranking the quality of the evidence using the Oxford Centre for Evidence‐based Medicine ‐ Levels of Evidence scale. Using the Reach Effectiveness Adoption Implementation Maintenance (RE‐AIM) framework, we defined the scale of delivery (i.e., the proportion of a target population who are provided with the intervention) as a plausible rate of annual expanded access for HIV prevention programs and as the product of healthcare setting‐specific reach and adoption for HIV testing, antiretroviral therapy (ART) engagement, and ART re‐engagement interventions.

We synthesized evidence from 11 peer‐reviewed publications, 12 public health and surveillance reports, and 3 publicly‐available data sets. Plausible annual rates of expanded access ranged from 7%‐15% increase for syringe service programs to 74%‐107% for pre‐exposure prophylaxis (PrEP). Plausible ranges of the scale of delivery for HIV testing interventions ranged from 3%‐9% (nurse‐initiated) to 19%‐51% (opt‐out). We estimated ART engagement could reach from 8%‐25% (coordinated care) to 29%‐84% (EMR‐prompt) diagnosed people living with HIVAIDS. The ART re‐engagement interventions were estimated to reach from 7%‐49% of those who have discontinued ART (Figure 1).

Basing simulation analyses and estimating impacts of evidence‐based interventions delivered at feasible levels of implementation is critical to assessing their potential population‐level health and economic effectiveness.

Abstract TUPDD0204‐Figure 1. Plausible Ranges of Scale of Delivery.

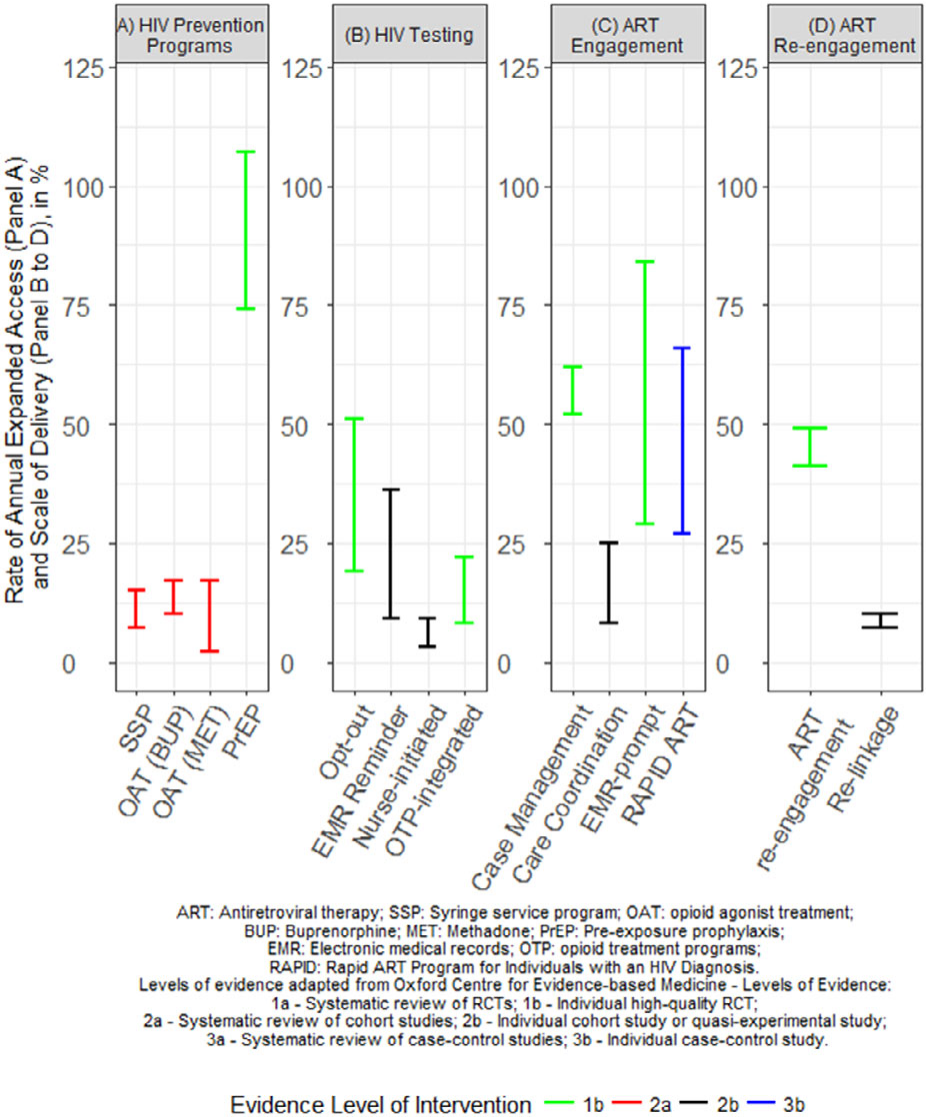

## TUPDD0205

### Extrapolation of population size estimates for key populations: Impact of method of extrapolation on estimates produced using regional estimates of female sex workers from Tanzania as an illustrative example

**A. Rao^1^; V. Loo^2^; T. Saidel^3^; A. Datta^4^ and S. Baral^1^**

^1^Johns Hopkins Bloomberg School of Public Health, Epidemiology, Baltimore, United States, ^2^Partnership for Epidemic Analysis ^PEMA^, Honolulu, United States, ^3^Partnership for Epidemic Analysis ^PEMA^, Lyons, United States, ^4^Johns Hopkins Bloomberg School of Public Health, Biostatistics, Baltimore, United States

Population size estimates(PSE) play a critical role in allocation of funding and planning, in disease surveillance, and in models to understand transmission dynamics. Methods for direct PSE, including capture‐recapture, multiplier methods, etc. each have their own limitations, but have been applied widely. Countries must extrapolate direct PSE from selected areas to obtain estimates at the national or sub‐national levels without direct data. Multiple methods for extrapolation have been applied, but with few metrics for assessing validity. Here, we show the impact of different methods of extrapolation and an approach for assessing the validity of results compared to known values. Publicly available data on population size of female sex workers(FSW) in Tanzania are used in an illustrative example.

Extrapolation results were obtained using three methods of extrapolation: simple imputation, stratified imputation, and regression using seven, direct FSW PSE. We allowed for variation in specification of how the estimate was to be modeled(estimate or proportion). We allowed variation in covariates selected. The different methods and variations by method resulted in 21 model permutations. Covariates included urbanicity, literacy, HIV prevalence, and deliveries in health facilities(%HF). We assessed validity of results using the leave‐one out approach for three of the permutations.

Depending on how the estimate was modeled, we observed a maximum 358% difference in extrapolated estimates holding all else constant (in Simuyi region using estimate itself:4491 vs. using proportion of women living in urban areas:980). Across methods, there was as much as a 342% difference in extrapolated estimates holding all else constant (in Njombe region using simple imputation:4491 vs. using multiple regression:19894). Differences were also observed depending on the covariate selected. Using the leave‐one out approach, multiple regression performed the best.

Extrapolation can be used as an analytical tool to optimize use of existing data and provide data‐driven program planning, but choice of how the estimates are modeled, choice of models, and choice of covariates to include in models can have a dramatic impact on conclusions reached. Understanding of the estimates that do exist, what they represent, and discussions with local stakeholders and community groups are key to making informed decisions on extrapolation.

## WEPDB0101

### Evolution of HIV drug resistance surveillance in Brazil: A declining trend to celebrate

**R.E.G. Gonçalves Pinho; N.M.C. Veras; A.R.P. Pascom; J.B. Alonso; F.M. Rick; A.S. Benzaken and G.F.M. Pereira**

Ministry of Health ‐ Brazil, Department for Surveillance, Prevention and Control of STIs, HIV/AIDS and Viral Hepatitis, Brasília, Brazil

In 1996, Brazil´s Ministry of Health implemented a program for people living with HIV/AIDS, offering free, universal access to antiretroviral drugs (ARV). However, ARV resistance remains an obstacle to sustaining HIV suppression during antiretroviral therapy (ART). This study describes HIV drug resistance (DR) between 2008 and 2018 in Brazil.

HIV protease (PR) and reverse transcriptase (RT) sequences from 2008 to 2018 were selected from 18 + ARV‐experienced individuals. The presence of HIV DR (Stanford HIVdb Program) was characterized for main PR and RT inhibitors used in first‐ and second‐line regimens in Brazil. HIV subtype (Rega HIV Subtyping) and ART history data were assessed.

We analyzed 52,658 PR‐RT sequences. For all analyzed ARVs, HIV DR shows a continuous decline over the years, with a comprehensive drop of 39.7% for EFV and 29.3% for 3TC from 2015 on (Figure 1). The most common mutations occurred at codons M46 and I54 for PI, M184 for NRTI and K103 for NNRTI. Higher levels of resistance were observed in individuals exposed to 8 + ARVs, except 3TC and EFV, to which resistance was higher for those using 2 to 4 ARVs. From 2015 on, resistance to TDF increased in the last group when compared to those who used 5 + ARVs. Overall, subtype B was most prevalent, varying from 72%, in 2008, to 64% in 2018. Subtype C increased 183%, from 6% to 17%. Subtype F and recombinant forms remained around 11% and 9%, respectively. HIV resistance to PI was lower for subtype C, but no remarkable difference was observed for RT inhibitors across subtypes.

HIV DR decreased over the decade, reflecting ART improvement within Brazil's program. In 2018, viral suppression was 92% for individuals on ART. Differences in HIV resistance to PIs highlight the importance of monitoring subtype distribution. The latter must also be considered for HIV treatment policy implementation.

Abstract WEPDB0101‐Figure 1. HIV resistance to protease and reverse transcriptase inhibitors in Brazil, 2008 to 2018.

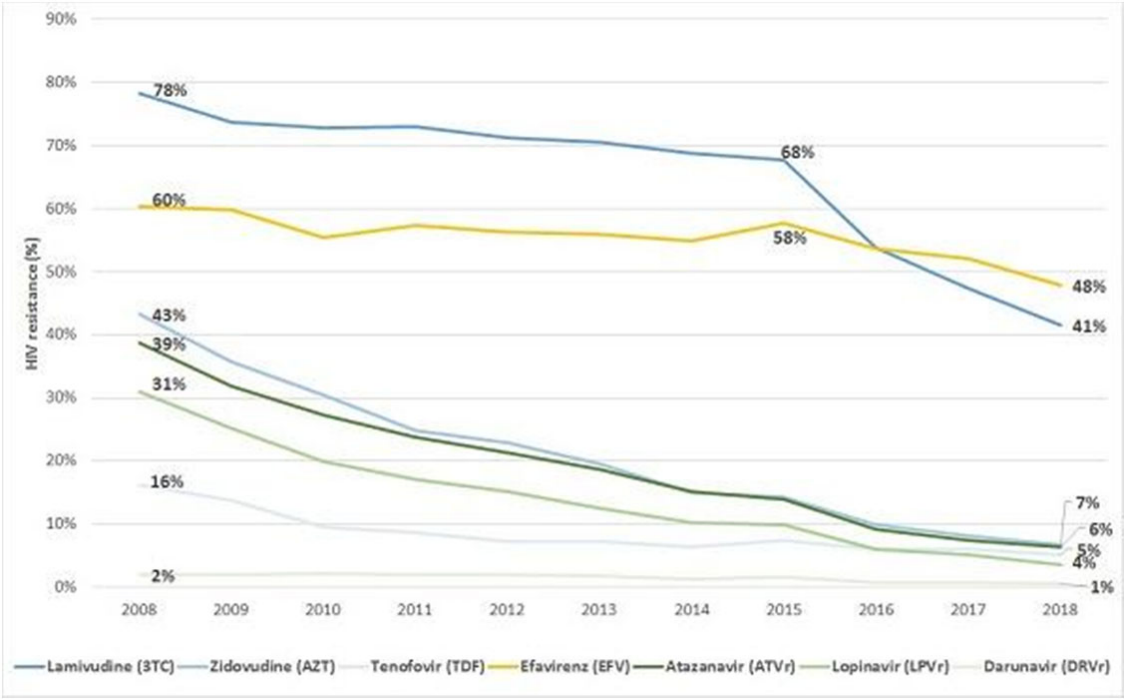

## WEPDB0102

### Thresholds of pre‐treatment HIV‐1 drug resistance indicate regions for priority actions in the antiretroviral therapy program of Cameroon

**J. Fokam^1,2^; M. Santoro^3^; D. Takou^1^; V. Tala^2^; G. Teto^1^; C. Chenwi^2^; G. Beloumou Angong^1,3^; E. Ngoufack Semengue^1,3^; S. Djupsa^1^; S.C. Billong^2,4^; J.‐B. Elat Nfetam^4^; J.‐B. N. Elat^4^; F. Ceccherini‐Silberstein^3^; V. Colizzi^3^; C.‐F. Perno^3,5^; A. Ndjolo^1,2^ and on behalf of VIROFORUM‐CIRCB**

^1^Chantal BIYA International Reference Centre for Research on HIV/AIDS Prevention and Management ^CIRCB^, Yaounde, Cameroon, ^2^Faculty of Medicine and Biomedical Sciences, University of Yaounde I, Yaounde, Cameroon, ^3^University of Rome Tor Vergata, Rome, Italy, ^4^National AIDS Control Comittee, Yaounde, Cameroon, ^5^University of Milan, Milan, Italy

The “Treat‐All” strategy ensures safer‐life among HIV‐infected individuals. Conversely, on‐going threats of HIV drug‐resistance (HIVDR) might vary by settings and impairs differently the benefit of first‐line antiretroviral therapy (ART). Our objective was to ascertain the thresholds and patterns of pre‐treatment drug resistance (PDR) by region and its possible association with subtype‐diversity.

A sentinel surveillance of PDR was conducted in seven regions of Cameroon from 2014 to 2018. Sequencing of HIV‐1 protease and reverse transcriptase was performed, drug resistance mutations (DRMs) was interpreted using Stanford HIVdb.v.8.7, and statistical analyses performed using EPI‐Info v7.2.2.6, with *p* < 0.05 considered statistically significant.

A total of 282 sequences (1 per patient) were generated in patients initiating antiretroviral therapy. The number of sequences per region was; 61, 53, 43, 41, 30, 30, and 24 for the Northwest, Centre, East, Littoral, West, Southwest, and North, respectively. The overall prevalence of PDR was 12.41% (35/282), distributed by drug‐class as follow: 10.28% (29/285) for NNRTIs, 7.45% (21/282) for 1st generation NNRTIs (NVP and EFV), 7.09% (20/282) for 2^nd^ generation NNRTIs (RPV and ETR), 2.84% (8/282) for NRTIs and 1.42% (4/282) for PIs (Fig.1A). The predominant mutations were: K103N (10), E138K/A/G (8), A98G (3), Y181C (2), G190A (2) for NNRTIs; M184V/I (3), K219N / E (3), T215S (2), K65R (2), M41L (2) for the NRTIs and M46L, L90M, V82F, L89V and G73S for the IPs. There was a disparity of the PDR between regions (North: 0%, Littoral: 9.76%, Centre: 7.55%, Northwest: 11.48%, West: 10.00%, Southwest: 23.33% and East: 23.26%), with similar regional trend of NNRTIs‐DRM (North: 0%, Littoral: 9.76%, Centre: 7.55%, North‐West: 9.84%, West: 6.67%, South‐West: 16.67% and East 18.60%), as depicted in Fig.1B. Overall, recombinants were predominant (237/282, 84.04%), CRF02_AG being having 68.09% (192/282), as shown in Fig1C. No statistically significant difference was observed between the PDR in recombinant forms and the pure strains (12.66% vs 11.11%) as well as between CRF02_AG and non‐AG subtype (11.46% vs 14.44%, *p* = 0.56).

The heterogeneous PDR reveals two regions with EFV/NVP‐PDR beyond 10%, thus requiring either closer monitoring, transition to Dolutegravir‐based first‐line ART‐regimens, or affordable HIVDR‐testing for patients initiating ART in these country‐regions

## WEPDB0103

### Prevalence and predictors of etravirine resistance mutations in HIV‐positive individuals failing second‐line antiretroviral therapy in Uganda

**G. Namayanja‐Kaye^1^; A.C. Awor^2^; C. Katureebe^3^; J. Nakaweesi^4^; M. Ssonko^4^; E. Namusoke Magongo^3^; I. Sewanyana^5^; H. Nansumba^5^; M. Adler^1^; C. Watera^6^; K. Jacobson^1^; A. Namale^1^; E. Raizes^7^ and F. Ssali^8^**

^1^CDC Uganda, Health Services Branch, Kampala, Uganda, ^2^CDC Uganda, Strategic Information Branch, Kampala, Uganda, ^3^Ministry of Health, AIDS Control Program, Kampala, Uganda, ^4^Mild May Center Uganda, Kampala, Uganda, ^5^Central Public Health Laboratory, Kampala, Uganda, ^6^Uganda Virus Research Institute, Entebbe, Uganda, ^7^CDC Atlanta, Care and Treatment Branch, Atlanta, United States, ^8^Joint Clinical Research Centre, Kampala, Uganda

Uganda's guidelines recommend HIV drug resistance (HIVDR) genotyping for patients failing protease inhibitors (second line). WHO recommends Etravirine (ETR) as one of the three backbone drugs for third‐line antiretroviral therapy (ART). Individuals failing second‐line therapy may have resistance mutations hindering use of ETR for effective third line. This analysis assesses prevalence and predictors of ETR resistance in HIV patients failing second‐ line therapy in Uganda.

Dried blood spots (DBS) from upcountry facilities and plasma from peri‐urban facilities were sent to central public health laboratories for routine viral load (VL) testing. HIVDR genotyping for clients failing second line began June 2017. All 6‐month repeat VLs ≥ 1000 copies/ml were sent for genotyping. Genotypes were used to determine third‐line regimens by the National Third Line Committee. A retrospective cross‐sectional review of de‐identified data for patients failing second line June 2017 to November 2018 was conducted. ETR resistance‐associated mutations (RAMs) were scored using Tibotec genotypic weighting scale, ETR Score (ETR‐S) > 2.5 was considered ETR resistance.

Genotype results for clients failing second line during the study period numbered 267, with two excluded for missing data were reviewed. Females were 114 (43.0%), children < 15 years 104 (39.2%). Overall, 146 (55.1%) had prior Nevirapine (NVP) exposure, 71 (26.8%) EFV, 37 (14%) both, and 11 (5%) neither. Those with NVP as first line had a mean ART duration of 4.8 years, versus 4.5 years for EFV (*p* = 0.512). Median initial detectable VL for prior NVP exposure was 19,904 copies/ml versus EFV 66,084. Median repeat VLs were 13,900 copies/ml (NVP) versus 30,257 (EFV). Altogether, 225 (84.9%) clients had non‐nucleoside reverse transcriptase inhibitor (NNRTI) mutations. The mean ETR‐S was 1.5 (SD ± 0.66). Overall, 176 (66.4%) had a score of < 2.5, while 89 (33.6%) had a score ≥ 2.5. The mean ETR‐S for children was 1.64, while that of adults was 1.49 (*p* = 0.477). The mean ETR‐S for past NVP was 1.7 versus 1.1 for past EFV (*p* = 0.0031).

ETR resistance among patients needing third‐line ART was high, with one‐third of eligible clients already resistant. NVP exposure predicted ETR resistance, with implications for third‐line ART in Uganda and beyond.

## WEPDB0104

### HIV‐1 drug resistance and third‐line outcomes among children and adolescents failing second‐line therapy in Malawi

**L. Lee^1^; I. Mbingwani^2^; T. Kalua^3^; S. Spiers^1^; S. Duranti^1^; B. Schramm^4^; R. Kamba^1^; E. Szumilin^5^; L. Salumu^5^ and D. Maman^1^**

^1^Médecins Sans Frontières, Chiradzulu, Malawi, ^2^Ministry of Health, Chiradzulu District Hospital, Chiradzulu, Malawi, ^3^Ministry of Health, Department of HIV and AIDS, Lilongwe, Malawi, ^4^Epicentre, Paris, France, ^5^Médecins Sans Frontières, Paris, France

Children and adolescents living with HIV are more likely than adults to have poor adherence and fail treatment. Using routinely collected data from a Médecins Sans Frontières supported project in rural Malawi, we report second‐line resistance and third‐line outcomes among children and adolescents failing second‐line treatment.

We conducted a retrospective cohort analysis on child and adolescent patients (< 20 years old) failing second‐line (an ART regimen that includes a PI) who received a genotype between 2014 to 2018. Treatment failure was defined as two consecutive high viral loads (VL). Third‐line was defined as an ART regimen that changes at least two drugs and includes one integrase inhibitor. Resistance to an ARV was defined as a score of 30 or above according to Stanford algorithms.

Among 99 patients that received a genotype, 85 were receiving LPV/r and 14 ATV/r. Median time on second‐line was 26 months [IQR 14,43]. 28 patients were resistant to at least one PI; 75 to at least one NRTI; 86 to at least one NNRTI. PI resistance was higher among those on second‐line for more than two years (35% vs. 20% for less than 2 years), but the difference was not significant using multivariate analysis (aOR 2.25; 95%CI 0.89, 5.71).

Abstract WEPDB0104‐Table 1. Association between PI resistance and risk factors among children and adolescents failing 2nd line ART treatment in Malawi, multivariate logistic mode

**Table:** 

		No. of patients (n = 99)	No. with PI res	Adjusted Odds Ratio	95% CI
Months on 2nd line before genotype	Less than 24 months	45	9 (20%)	ref.	[0.89,5.71]
	24 months or more	54	19 (35%)	2.25	
Age at genotype	3 to 11 years	50	15 (30%)	ref.	[0.34,2.05]
	12 to 19 years	49	13 (27%)	0.84	
Patient sex	Female	41	9 (22%)	ref.	[0.64,4.54]
	Male	58	19 (33%)	1.78	

Among 33 patients switched to third‐line (25 on DTG, 8 on RAL, 21 on DRV), retention in care at 12 months was 97% (95%CI: 80%,100%). VL suppression at 6 and 12 months following 3^rd^ line initiation were 86% (19/22) and 85% (11/13), respectively. Among 66 patients remaining on second‐line, retention in care 12 months following the genotype was 88% (95%CI: 74%,95%). VL suppression at 6 and 12 months following the genotype were 39% (17/44) and 33% (8/24), respectively.

ARV resistance among children and adolescents failing second‐line was common, making access to genotypes important. Additional studies are needed on children and adolescents failing second‐line but not switched to third‐line. Third‐line pediatric formulations should be developed.

## WEPDB0105

### HIV‐1 drug resistance surveillance among parturient women on anti‐retroviral therapy in the Eastern Cape, South Africa: Implications for elimination of mother‐to‐child transmission

**O.V. Adeniyi**

^1^Walter Sisulu University, Department of Family Medicine, East London, South Africa, ^2^University of Fort Hare, Microbiology, Alice, South Africa

HIV drug resistance poses threat to the goal of elimination of mother‐to‐child in South Africa. In this study, we assessed the burden of HIV‐1 drug resistance mutations (DRMs) within the public sector prevention of mother‐to‐child transmission (PMTCT) programme in Eastern Cape, South Africa. We also examined the potential transmissibility of mutant viruses within the cohort.

We conducted genetic analysis on viral isolates (n = 80) from plasma samples of women with virologic failure at delivery between January and May 2018 from two large maternity centres in the Eastern Cape. Partial pol gene sequences were amplified and sequenced according to standard protocol. DRMs were determined by submitting the generated partial pol sequences to the Stanford drug resistance database for query as well as to the IAS guidelines for DRMs interpretations. These curated algorithms provide an online software for determining genotypic resistance associated mutations in HIV pol sequences.

The age of parturient women ranged from 16 ‐ 43 years. The majority of the parturient women were in WHO clinical stage 1 (62.0%), currently on Efavirenz‐based regimen (first line ART) (82.5%) and had been on ART for more than 12 months (65.0%). The prevalence of DRMs was 72.5% (n = 58). The CD4 count demonstrated a negative linear association with the DRMs (*p* = 0.002). Sub‐type C accounted for nearly all the DRMs (98.3%; n = 78). We found a CRF02_AG and URF. The predominant DRMs were K103N (n = 43; 74.1%), M184V (n = 28; 48.3%) and K65R (n = 11; 19%). Among the parturient women on current treatment of EFV‐based regimen; 79.1% already had K103N while nine patients on protease inhibitor‐based regimen still harbors K103N. Other mutations conferring resistance to NNRTIs include: V106M (15.5%) and P225H (17.2%). The majority of the M184V mutations were observed in parturient women on first line regimen (n = 23; 82.1%). The mean viral load (transmissibility risks) in DRMs was significantly higher than the wild type (174515 versus 52426).

We found a high prevalence of DRMs in women delivering at high viral loads in Eastern Cape, South Africa. Surveillance system for tracking pregnant women on ART will assist in identifying those with virologic failure and drug resistance for interventions.

## WEPDB0106

### Going beyond guidelines: HIV‐1 drug resistance testing at low‐level viremia, a South African experience

**A. Bangalee^1,2^; K. Steegen^3,4^; S. Carmona^3,4^ and L. Hans^3,4^**

^1^University of Witwatersrand, Medical Virology, Johannesburg, South Africa, ^2^National Health Laboratory Service, Medical Virology, Johannesburg, South Africa, ^3^University of Witwatersrand, Haematology and Molecular Medicine, Johannesburg, South Africa, ^4^National Health Laboratory Service, Haematology and Molecular Medicine, Johannesburg, South Africa

A subset of patients on antiretroviral treatment (ART) present with viral load (VL) levels that range between 50 to 1000 cp/mL, termed low‐level viremia (LLV). LLV has been associated with drug resistance mutations (DRMs) across drug classes. We identified a need for investigation of LLV DRMS in South African patients as national guidelines recommend referral for drug resistance (DR) testing only at VLs > 1000 cp/mL, as well as for an evaluation of genotyping assay success rate at this level

We conducted an observational, retrospective, cohort study on patient samples with LLV referred for routine DR testing at a Johannesburg laboratory from August 2017 ‐ October 2018. Genotyping was performed using a previously validated nested RT‐PCR assay. The genotyping success rate was evaluated for different viremia ranges. HIV‐1 drug resistance analysis was done using Sanger sequencing and sequences were loaded onto the Stanford HIVdb genotypic resistance tool (v 8.7) for drug resistance interpretation.

Plasma samples from 123 HIV‐1 infected, treatment‐experienced adults were analysed. Most patients were female (53.7%), median age was 42.7 years (IQR: 49 to 37). Assay success rate was 75.6% of which 75.8% (72/95) had DRMs. The in‐house assay performed best for samples with a VL 401 to 999 with an overall genotyping success rate of 88%.

ART regimen at the time of HIVDR testing was available for 100(81%) patients of which 12(12%) and 88(88%) were on a NNRTI and PI‐based regimen respectively.

DRMs were commonly encountered in patients with LLV ranging from 401 to 999 cp/mL. Mutations detected were: NNRTIs at 47% (45/95), most commonly K103N/Q (84%); NRTIs at 68% (65/95) with M184V/I at 89%, TAMS at 86%, as well as the Q151M complex coupled with the K65R in 2 patients, indicating multinucleoside resistance. Finally major PI mutations including M46I/L/V and V82A were detected in 9 out of 95 (9%) patients. Eight patients with PI mutations were known to be on a PI‐based regimen at the time of genotyping.

Virological failure guidelines may keep patients on failing regimens for longer. Our data suggests that genotyping at LLV is feasible and implementation could result in earlier identification and referral of patients requiring third line regimens

## WEPDC0101

### #HIVSelfTest: A social media campaign to promote HIV self‐testing among young people in Nigeria

**J. Iwelunmor^1^; O. Ezechi^2^; C. Obiezu‐Umeh^1^; U. Nwaozuru^3^; T. Gbajabiamila^2^; F. Falodun^4^; A. Eklu^5^; C. Ihidero^6^ and J. Tucker^7^**

^1^Saint Louis University, College for Public Health and Social Justice, Saint Louis, United States, ^2^Nigerian Institute of Medical Research, NIMR, Lagos, Nigeria, ^3^Saint Louis University, College for Public Health and Social Justice, Saint Louis, Nigeria, ^4^ID Africa, Lagos, United States, ^5^ID Africa, Lagos, Nigeria, ^6^Pinpoint Media, Lagos, Nigeria, ^7^Nigerian Institute of Medical Research, NIMR, Lagos, United States

Despite a persistent HIV incidence among young people in Nigeria, HIV testing remains low among this population. Novel and youth‐centered strategies are needed to increased uptake of HIV testing among this population. The objective of this study was to evaluate a youth‐focused social media campaign to promote HIV self‐testing (HIVST) among young people in Nigeria.

Between October‐December 2018, we disseminated messages emphasizing HIV self‐testing through social media platforms such as Facebook, Instagram, WhatsApp, digital channels and social media influencers in Lagos, Nigeria. We used social media metrics to examine the reach of a social media campaign focused on HIVST among Nigerian youth.

With over 89.5% of output being original HIV self‐testing content, the social media campaign reached over 3.5 million people with an estimated 17.2 million impressions. Majority of the engagement content across our various social media platforms (Facebook, Instagram, WhatsApp) were grouped into the following themes: 1) Informational (55%) (e.g. provided youth with facts, statistics on HIVST and HIV testing in general); Collaborative (31%) (e.g. collaborated with youth to promote HIVST; and 3) Empowerment (14%) (e.g. delegated youth with decision‐making to promote HIVST).

Findings show the dynamics and potential utility of using social media platforms for meaningful youth engagement, effective messaging and real‐time monitoring of campaigns to promote HIV self‐testing among young people in Nigeria. Additional HIV self‐testing social media campaigns led by and promoted by young people themselves should be attempted and evaluated.

Abstract WEPDC0101‐Figure 1. Social media campaign on instagram.

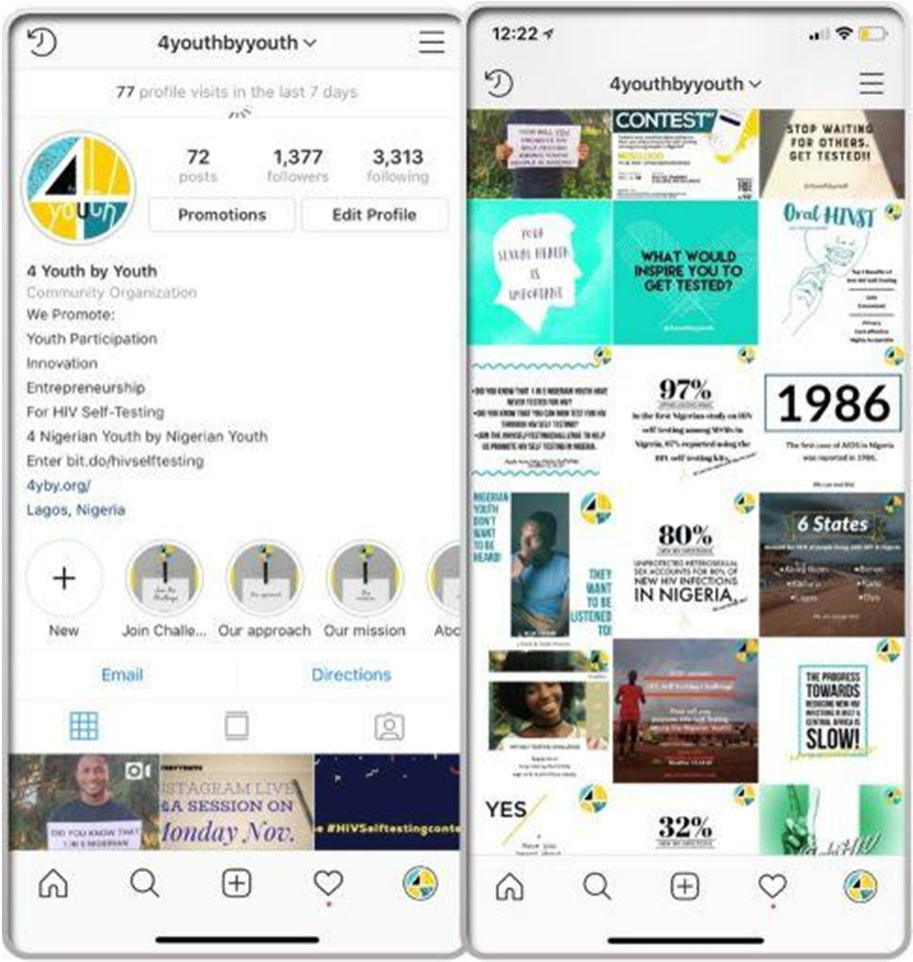

## WEPDC0102

### Developing a novel mobile app to support HIV testing and PrEP uptake among young MSM: The LYNX Study

**A. Liu^1,2^; K. Coleman^1^; J. Vinson^1^; R. Muench^3,4^; K. Bojan^3,4^; P.A. Serrano^3,4^; T. Oyedele^3,4^; A. Garcia^5^; E. Enrique‐Bruce^5^; P. Emmanuel^5^; J. Jones^6^; K. Muessig^7^; C. Horvitz^7^; S. Mullin^6^; J. Roberts^7^; S. Buchbinder^1,2^; P. Sullivan^6^; L. Hightow‐Weidman^7^ and H. Scott^1,2^**

^1^San Francisco Department of Public Health, San Francisco, United States, ^2^University of California ‐ San Francisco, San Francisco, United States, ^3^Ruth M. Rothstein CORE Center, Chicago, United States, ^4^John H. Stroger Hospital, Chicago, United States, ^5^University of South Florida, Tampa, United States, ^6^Emory University, Rollins School of Public Health, Atlanta, United States, ^7^University of North Carolina at Chapel Hill, Chapel Hill, United States

Young men who have sex with men (YMSM) have the highest HIV incidence and lowest uptake of HIV testing and pre‐exposure prophylaxis (PrEP) in the US. Nearly universal mobile phone ownership among youth provides an opportunity to leverage mobile‐health apps to increase testing and PrEP uptake among YMSM.

Using the Information, Motivation, Behavioral Skills Model, we developed the LYNX app (available on iOS/Android) which features an electronic diary to track sexual behaviors, a personalized SexPro score to promote accurate risk perception, testing reminders and access to home‐based HIV/STI test kits and geospatial testing/PrEP sites, and bi‐directional chat support (Figure). Within the Adolescent Trials Network iTech U19, we conducted iterative focus groups among YMSM in Chicago and Tampa to refine the app, followed by a two‐month open pilot to optimize usability; preliminary feasibility and acceptability were assessed through app analytics and the System Usability Scale (SUS).

30 YMSM participated in two focus groups (mean age 20, 43% Latino, 43% Black). Overall, the app was well‐received, especially the sexual diary and gamification features (sex‐positive badges). They recommended making the goal of LYNX (to “marry pleasure with prevention”) clear during app onboarding. Regarding SexPro, they suggested adding information on behaviors contributing to their risk scores and how to improve them. In the open‐pilot with 16 YMSM (median age 22, 44% Latino, 19% Black), 93% used the app ≥ 2 times, with an average of 9 login sessions and 9/16 features accessed. Median SUS score was 72/100 (“good” range). Overall, 85% were very satisfied with LYNX, and 86% would recommend the app to a friend for HIV/STI testing or accessing PrEP.

The LYNX app, developed through iterative feedback from YMSM, was found to be feasible and acceptable in early testing. Additional studies are underway to assess acceptability and efficacy in broader youth populations.

Abstract WEPDC0102 Figure 1. Screenshots of LYNX app.

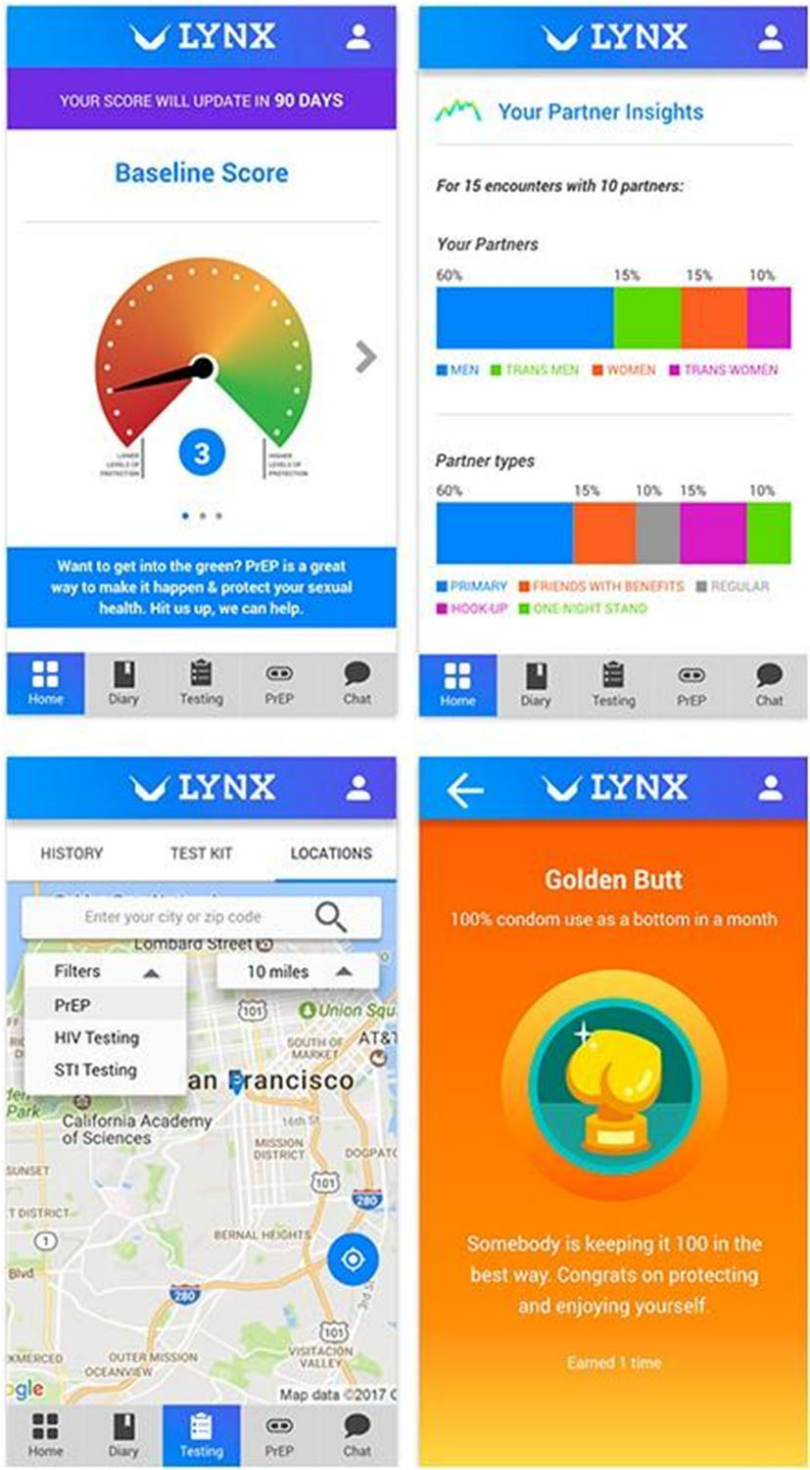

## WEPDC0103

### Stigma and online sex‐seeking among men who have sex with men and transgender women in Tijuana, Mexico

**C. Espinosa da Silva^1^; L. Smith^1^; T. Patterson^2^; S. Semple^2^; A. Harvey‐Vera^3^; S. Nunes^1^; G. Rangel^4,5^ and H.A. Pines^1^**

^1^University of California, San Diego, Medicine, La Jolla, United States, ^2^University of California, San Diego, Psychiatry, La Jolla, United States, ^3^Universidad Xochicalco, Tijuana, Mexico, ^4^United States‐Mexico Border Health Commission, Tijuana, Mexico, ^5^Colegio de la Frontera Norte, Tijuana, Mexico

Stigma toward sexual and gender minorities is an important structural driver of HIV epidemics among men who have sex with men (MSM) and transgender women (TW) globally. Sex‐seeking websites and applications are popular among MSM and TW. Interventions that harness these online platforms may be particularly effective for engaging MSM and TW in HIV prevention and treatment services in settings with widespread stigma towards these vulnerable populations.

To assess the feasibility of this approach, we determined the prevalence of online sex‐seeking and examined the effect of factors that shape or are influenced by stigma toward sexual and gender minorities on online sex‐seeking among MSM and TW in Tijuana, Mexico.

From 2015 to 2018, 529 MSM and 32 TW were recruited through venue‐based and respondent‐driven sampling. Interviewer‐administered surveys collected information on online sex‐seeking (past 4 months) and factors that shape or are influenced by stigma toward sexual and gender minorities (traditional *machismo*, internalized stigma related to same‐sex sexual behavior or gender identity, outness related to same‐sex sexual behavior or gender identity; MSM only: sexual orientation, history of discrimination related to same‐sex sexual behavior). Logistic regression was used to examine the association between each stigma measure and online sex‐seeking.

Twenty‐nine percent of our sample reported seeking sex partners online. Online sex‐seeking was negatively associated with greater endorsement of traditional *machismo* values (adjusted odds ratio [AOR]=0.37, 95% confidence interval [CI]: 0.19 to 0.71) and greater levels of internalized stigma (AOR = 0.96, 95% CI: 0.94 to 0.99). Online sex‐seeking was positively associated with identifying as gay (AOR = 2.04, 95% CI: 1.30 to 3.21), greater outness (AOR = 1.17, 95% CI: 1.06 to 1.28), and a history of discrimination (AOR = 1.85, 95% CI: 1.10 to 3.14).

Online sex‐seeking is relatively common among MSM and TW in Tijuana, suggesting that it may be feasible to leverage online platforms to engage these vulnerable populations in HIV prevention and treatment services. However, such interventions may still poorly engage those most affected by stigma toward sexual and gender minorities (i.e., those who express greater endorsement of traditional *machismo* values, greater levels of internalized stigma, less outness, and non‐gay identification) given that within our sample they were least likely to seek sex online.

## WEPDC0104

### Transgender‐led social media interventions effectively identify transgender woman subpopulations with substantial risk of HIV acquisition and successfully link to HIV prevention, care and treatment services

**R. Janamnuaysook^1^; K. Samitpol^1^; J. Kongkapan^1^; A. Chancham^1^; T. Amatsombat^1^; P. Getwongsa^1^; J. Rueannak^1^; K. Termvanich^1^; J. Uttayananon^1^; P. Mingkwanrungruang^1^; R. Ramautarsing^1^; M. Avery^2^; S. Mills^2^; R. Vannakit^3^; P. Phanuphak^4^ and N. Phanuphak^1^**

^1^PREVENTION, Thai Red Cross AIDS Research Centre, Bangkok, Thailand, ^2^FHI 360 and U.S. Agency for International Development LINKAGES Project, Bangkok, Thailand, ^3^Office of Public Health, U.S. Agency for International Development Regional Development Mission Asia, Bangkok, Thailand, ^4^Thai Red Cross AIDS Research Centre, Bangkok, Thailand

Transgender communities are often neglected in mainstream health care facilities due stigma, discrimination, and unavailability of transgender‐specific and transgender‐friendly services. Transgender people are therefore more likely to seek health information online, and to rely on gender‐affirming hormone treatment (GAHT) experience from transgender peers and transgender social influencers. We utilized transgender‐led, targeted social media interventions to raise health awareness and facilitate uptake of HIV testing, syphilis testing, pre‐exposure prophylaxis (PrEP), and post‐exposure prophylaxis (PEP) through integrated GAHT services among transgender women (TGW) in Thailand.

The Tangerine Community Health Center in Bangkok provides HIV and other sexual health services integrated with GAHT services for TGW. Beginning in August 2017, transgender social media influencers conducted Tangerine Facebook Live Sessions as the primary online demand generation platform to transform online networking to offline health care services. Characteristics of TGW who were reached through social media and who subsequently accessed HIV and related health services at Tangerine were recorded.

Of 1,360 TGW who attended the Tangerine Community Health Center between August 1, 2017, and December 25, 2018, 999 (73%) were reached through the Tangerine Facebook Live Sessions. Among those, the median age was 25 years, 54% had education below a bachelor's degree, 15% were unemployed, and 14% engaged in sex work. GAHT services were the primary purpose of clinic visits for 63%. Among the 999, 928 (93%) received HIV testing and 67 (7%) tested HIV‐positive, of whom 62 (93%) successfully initiated antiretroviral treatment. Of the 928 who received HIV testing, 333 (36%) were first‐time HIV testers. Among the 999, 372 (37%) received syphilis testing, and 64 (17.2%) tested reactive to syphilis. Fifty‐eight (6%) were prescribed PrEP, and 88 (9%) were prescribed PEP.

Transgender‐led social media inventions reached vulnerable TGW subpopulations, identified those TGW at substantial risk of HIV acquisition, and facilitated linkages to HIV testing and treatment. As GAHT services were the primary entry point into care for most TGW reached online, addressing specific transgender health needs through virtual engagement should be brought to scale in order to increase the uptake of HIV prevention, care and treatment services among transgender populations.

## WEPDC0106

### Pilot study of a gamified, social networking app shows improvements in PrEP adherence among YMSM in the US

**L. Hightow‐Weidman^1^; K. Knudtson^1^; A. Mcgee^1^; M. Cottrell^2^; E. Adam^3^; M. Paul^4^; K. Desir^5^; D. Futterman^6^; Z. Woytowich^7^; K. Muessig^8^; K. Claude^1^ and S. LeGrand^9^**

^1^University of North Carolina‐Chapel Hill, Institute for Global Health and Infectious Diseases, Chapel Hill, United States, ^2^University of North Carolina‐Chapel Hill, Pharmacology, Chapel Hill, United States, ^3^Emory University, Rollins School of Public Health, Epidemiology, Atlanta, United States, ^4^Baylor College of Medicine, Pediatric Retrovirology and Global Health, Houston, United States, ^5^Childrens Hospital of Philadelphia, Philadelphia, United States, ^6^Montefiore Children's Hospital, Bronx, United States, ^7^Ayogo, Inc., Vancouver, Canada, ^8^University of North Carolina‐Chapel Hill Gillings School of Public Health, Department of Health Behavior, Chapel Hill, United States, ^9^Duke University, Duke Global Health, Durham, United States

P3 (Prepared, Protected, emPowered) is a theory‐based, comprehensive social networking PrEP adherence app designed for young men who have sex with men (YMSM) and transwomen (YTW), which includes game‐based elements to encourage engagement. Strengths‐based adherence counseling delivered by a centrally‐located adherence counselor via in‐app messaging addresses individuals’ unique barriers to PrEP adherence.

We conducted a one‐month field trial of P3. At baseline and follow‐up, participants completed a computer‐assisted survey and dried blood spots were collected for PrEP adherence measurement (i.e. TFVdp/FTCtp concentrations). Descriptive analyses of baseline variables are presented. A Wilcoxon signed‐rank test was used to assess changes in adherence measures between visits.

We enrolled 16 participants newly starting or reporting PrEP adherence challenges at three US cities. Mean age was 21.3 years, 25% were Black and 69% Hispanic. All identified as male and 15 (93.8%) as gay. Length of time on PrEP was > 3 months (25%), 1 to 3 months (50%), and < 1 month (25%). At baseline, half reported prior PrEP usage and discontinuation, half reported an STI diagnosis and 43.8% reported condomless anal sex with an HIV+ or unknown status partner in the prior 3 months. Retention was 94%. Self‐reported adherence (i.e. mean percent of time taken PrEP in the past month) improved from 72.5% to 89.3% between visits (*p* = 0.010). Median TFVdp and FTCtp increased by 44% and 62%, respectively between visits, although the increase was not statistically significant (*p* > 0.1). All measures of PrEP self‐efficacy improved, with significance detected in ability to follow a PrEP plan, take PrEP on a weekend, at a social outing or party, and take PrEP when having medication side effects.

While our pilot results should be interpreted cautiously, the observed improvements in both self‐reported and biologically‐confirmed adherence measures are encouraging. While some participants recently started PrEP, self‐reported time on PrEP was not correlated with baseline TFVdp/FTCtp concentrations. Thus, it is unlikely that the trend of increased concentration is an artifact of accumulation to steady‐state. A randomized controlled efficacy trial of P3 will begin Spring 2019 at seven US Adolescent Trials Network sites.

## WEPDC0107

### Digitizing interventions: An internet‐based approach to reach out to the ‘hidden network of men who have sex with men’ in Mumbai, India

**A. Palkar^1^; S. Acharya^1^; M. Setia^2^; M.R. Parthasarathy^3^ and P. Keskar^1^**

^1^Mumbai Districts AIDS Control Society, Mumbai, India, ^2^Consultant Dermatologist and Epidemiologist, Mumbai, India, ^3^FHI 360 and LINKAGES, New Delhi, India

Enhanced Peer Outreach Approach (EPOA) is an important strategy to reach the unreached key populations (KPs). We conducted an internet based EPOA to reach out to high‐risk men‐who‐have sex with men (MSM) who solicit through dating apps in Mumbai, India.

240 MSM (25 seeds and 215 wave participants) were recruited using an internet‐based snowball sample during 2017 to 18. Information on demographics, sexual behaviors and preferences, exposure to targeted interventions was collected.

Of the 240 MSM enrolled, 25 (10%) were seeds, 52 (22%) in wave one, 64 (27%) in wave 2, 33 (18%) in wave 3 and 4, 17 (7%) in wave 5, and 15 (6%) in wave 6. Nine seeds didn't recruit any MSM; the values ranged from 2 to 80 in the other seeds. The mean age (SD) was 23.9 (5.5) years; there was no difference in mean ages across different waves (*p* = 0.32). Though most MSM in seeds and initial waves were students, a higher proportion of working MSM were recruited in subsequent waves (after 3) compared with seeds (*p* = 0.02). None of the individuals after wave four knew about the targeted prevention interventions (TI) under national program; this was significantly lower compared with earlier waves (10%) (*p* = 0.04). Grinder (44%) was most common source to find partners, followed by Facebook (25%) and Whatsapp (21%); the preferences differed significantly across waves (*p* < 0.001). About 3% were HIV infected and 9% were reactive for syphilis. Most of the MSM infected by syphilis were recruited by one seed (30% vs. 4%, *p* < 0.001) (Pic_01).

Internet‐based EPOA is useful to reach hidden & unreached MSM networks. Thus, these EPMs should be integrated into MSM interventions in India to reach the hidden population, where social framework is not conducive for MSM to ‘come‐out’ and become a part of HIV prevention programs.

Abstract WEPDC0107‐Figure1. Showing the network and VDRL reactivity in EPOA from 25 seeds.

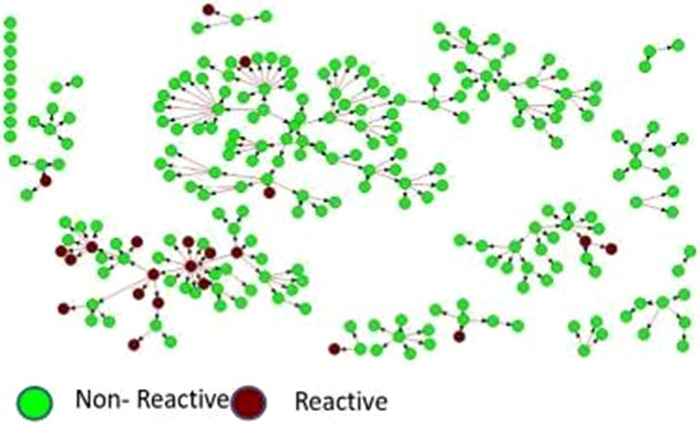

## WEPDC0201

### Healthcare providers’ attitudes and experiences delivering oral PrEP to adolescent girls and young women: Implementation research to inform PrEP rollout in Kenya, South Africa, and Zimbabwe

**M. Lanham^1^; K. Stankevitz^1^; K. Ridgeway^1^; M. Mireku^2^; D. Nhamo^3^; D. Pillay^4^; M. Murire^4^; J. Kyongo^2^; N. Makahamadze^3^; S. Pradhan^1^; M. Lydon^1^; L. Digolo^2^; P. Jeckonia^2^; P. Shamu^4^; T. Bhatasara^5^; G. Ncube^5^; J. Murungu^3^; W. Mukoma^2^ and S. Mullick^4^**

^1^FHI 360, Durham, United States, ^2^LVCT HEALTH, Nairobi, Kenya, ^3^Pangaea Zimbabwe AIDS Trust, Harare, Zimbabwe, ^4^Wits Reproductive Health and HIV Institute ^Wits RHI^, Johannesburg, South Africa, ^5^Zimbabwe Ministry of Health and Child Care, Harare, Zimbabwe

Oral PrEP is offered to populations at substantial HIV risk in Kenya, South Africa, and Zimbabwe, including adolescent girls (AG) 15 to 17 and young women (YW) 18 to 24. We examined providers’ attitudes and experiences delivering PrEP to AGYW to inform provider training and service delivery.

We surveyed providers (Kenya = 290, South Africa = 192, Zimbabwe = 127) and conducted follow‐up qualitative interviews (Kenya = 40, South Africa = 48, Zimbabwe = 27). Participants included clinicians, nurses, counselors, pharmacists, and community‐based workers at public and private facilities; 334 had experience with PrEP delivery, and 274 did not. We descriptively analyzed survey data in STATA 13 and thematically analyzed interviews using NVivo 11.

Although PrEP delivery differs across countries, providers shared similar attitudes. While some survey participants agreed “it's better to tell sexually active unmarried women (AG 49%, YW 36%) to abstain from sex rather than give her PrEP,” providers in interviews acknowledged that many girls engage in sex before 18 and could benefit from PrEP. More providers (75%) believed YW were responsible enough to take PrEP consistently compared to AG (49%), stating that delivering services to YW is easier because they are “more mature” while some AG “don't listen.”

Providers delivering PrEP to AGYW reported that clients’ lack of PrEP knowledge and lack of disclosure were barriers to uptake, adherence, and retention. Side effects, lack of relationship power, and access barriers were also cited in Kenya. Providers thought AGYW should disclose PrEP use to parents (34% AG) and partners (52% AG, 57% YW; highest in Kenya, lowest in South Africa) to facilitate adherence but were concerned about negative reactions from parents/partners because of low PrEP awareness and HIV stigma. Providers shared strategies they used to help AGYW use PrEP successfully, including intensive adherence and relationship counseling, phone follow‐ups, home visits, peer counseling, and community awareness‐raising. Additional differences between countries and AG/YW will be presented.

Providers were generally supportive of PrEP for AGYW, with more reservations about AG. Results are informing provider training in these countries to address these reservations. Additional community sensitization about PrEP as a prevention option for AGYW—particularly targeting parents and partners—could make it easier for AGYW to use PrEP.

## WEPDC0202

### Providers’ attitudes towards and experiences with oral pre‐exposure prophylaxis (PrEP) implementation in the SEARCH trial in Kenya and Uganda

**C.S. Camlin^1^; M. Getahun^2^; C.A. Koss^3^; J. Ayieko^4^; M. Atukunda^5^; A. Owaraganise^5^; L. Owino^4^; H. Itiakorit^6^; C. Akatukwasa^7^; I. Maeri^4^; A. Onyango^4^; R. Bakanoma^6^; F. Atwine^7^; D. Kwarisiima^5^; N. Sang^4^; J. Kabami^7^; G. Chamie^3^; M.L. Petersen^8^; T. Clark^3^; C.R. Cohen^9^; E.A. Bukusi^4^; M.R. Kamya^5^; D.V. Havlir^3^ and E.D. Charlebois^3^**

^1^University of California ‐ San Francisco, Obstetrics, Gynecology & Reproductive Sciences, Oakland, United States, ^2^University of California ‐ San Francisco, Obstetrics, Gynecology & Reproductive Sciences, San Francisco, United States, ^3^University of California ‐ San Francisco, Medicine, San Francisco, United States, ^4^Kenya Medical Research Institute ^KEMRI^, Kisumu, Kenya, ^5^Infectious Diseases Research Collaboration, Kampala, Uganda, ^6^Infectious Diseases Research Collaboration, Mbale, Uganda, ^7^Infectious Diseases Research Collaboration, Mbarara, Uganda, ^8^University of California Berkeley, Berkeley, United States, ^9^University of California ‐ San Francisco, San Francisco, United States

Understanding the attitudes and experiences of pre‐exposure prophylaxis (PrEP) providers is critical for informing global implementation, yet to date limited research in Africa has explored providers’ views.

SEARCH (NCT01864603), a population‐based HIV test‐and‐treat trial, implemented a PrEP intervention in which providers received didactic training and ongoing support from local senior clinicians. In this qualitative study, researchers conducted semi‐structured interviews with providers and counselors (n = 19) in 5 communities in Kenya and Uganda from January‐September 2017 to explore perceptions and experiences with PrEP delivery. Transcripts were inductively coded using a framework developed by an 8‐person team.

Providers had heterogenous attitudes towards PrEP: some expressed enthusiasm for PrEP and others ambivalence. Some doubted patients’ ability to adhere, and feared being blamed for PrEP ‘failures’ (i.e. HIV seroconversions) in their communities. Offering PrEP presented a moral dilemma for some who feared that PrEP could lead to increased ‘immorality’, HIV and STI incidence, and mistrust within couples. Providers supported PrEP in HIV‐discordant couples, where mutual support for daily pill‐taking facilitated harmony and protection for seronegative partners. However, even providers supportive of PrEP struggled to communicate messages about usage and adherence, particularly identifying ‘seasons of risk’ and explaining complex guidelines for safely stopping and restarting PrEP. Assessing HIV risk was often difficult; providers accepted clients’ self‐referral for PrEP even when risk was not evident. Providers felt that PrEP uptake was hampered for women by difficulties negotiating use with partners, and for youth due to the need for parental consent; and that barriers to PrEP continuation included transportation costs, stigma, daily pill burden and side effects. Providers felt that continuation was facilitated by counseling, proactive management of side effects, and home or community‐vs. clinic‐based PrEP provision**.**

Providers are not neutral ‘implementation actors’, but rather should be the first‐line targets of interventions to promote adoption of new evidence‐based practices and technologies such as PrEP, as their attitudes and social roles affect dissemination. Providers need training and opportunities to build networks of mutual support to address the complex challenges of PrEP implementation. Their perspectives can and should inform PrEP policy frameworks and communications strategies.

## WEPDC0203

### Implementation challenges and strategies in integration of PrEP into maternal and child health and family planning services: Experiences of frontline healthcare workers in Kenya

**K. Beima‐Sofie^1^; A. Wagner^1^; J. Pintye^1^; F. Abuna^2^; H. Lagat^2^; J. Baeten^1,3,4^; J. Kinuthia^5^; G. John‐Stewart^1,3,4^ and G. O'Malley^1^**

^1^University of Washington, Department of Global Health, Seattle, United States, ^2^University of Washington in Kenya, Nairobi, Kenya, ^3^University of Washington, Department of Epidemiology, Seattle, United States, ^4^University of Washington, Department of Medicine, Seattle, United States, ^5^Kenyatta National Hospital, Nairobi, Kenya

Delivering PrEP to adolescent girls and young women (AGYW) through maternal and child health (MCH) and family planning (FP) clinics in Africa may substantially reduce HIV acquisition in this population. Evaluation of implementation challenges and strategies within health systems are critical to inform future scale‐up.

We conducted focus group discussions (FGDs) with healthcare workers (HCWs) offering PrEP in MCH and FP clinics as part of the PrEP Implementation for Young Women and Adolescents (PrIYA) Program in Kisumu, Kenya. Topic guides were based on the Consolidated Framework for Implementation Research (CFIR). An analysis of FGD audio and debrief reports was conducted to identify implementation challenges and employed strategies.

Overall, 50 HCWs from 26 facilities participated in 8 FGDs. HCWs felt that PrEP met the needs of AGYW by providing a female controlled prevention strategy, and aligned with policy priorities of elimination of mother‐to‐child HIV transmission. They were universally enthusiastic about PrEP provision to AGYW through MCH clinics, noting the relative advantage of this approach because it:

1 enabled high coverage,

2 harmonized PrEP and MCH visits, and

3 lowered stigma compared to PrEP offered through HIV care clinics.

HCWs noted implementation challenges including:

1 increased workload and documentation burden amid healthcare workforce shortages,

2 physical space constraints,

3 drug and paperwork stockouts,

4 multiple implementing partners with different PrEP priorities and documentation practices at the same site, and

5 increased HIV testing sessions.

HCWs employed various implementation strategies to overcome implementation challenges, including task shifting from nurses to HIV Testing Service (HTS) providers, facility‐specific patient flow modifications (including fast‐tracking PrEP clients to reduce wait times), PrEP demand‐generation and myth‐busting during health talks, provider education, dedicated PrEP delivery rooms, and coordination with adolescent friendly services. Additional suggested strategies to improve PrEP integration included community education to increase broader PrEP awareness and enable shorter counseling sessions, and task‐shifting data entry and client risk assessments.

HCWs were enthusiastic about the feasibility, acceptability, and potential sustainability of integrating PrEP services into MCH and FP clinics. Challenges and strategies focused on overcoming provider time and space constraints, and addressing provider and client knowledge.

## WEPDC0204

### Surmounting PrEP delivery challenges through adaptation of implementation guidelines: Lessons learned from HIV care clinics in Kenya

**E. Irungu^1,2^; K. Ngure^2,3^; K. Mugwanya^2^; N. Mugo^1,2^; E.A. Bukusi^1,2^; J. Odoyo^1^; E. Wamoni^1^; J. Morton^2^; G. O'Malley^2^; J.M. Baeten^2^ and for the Partners Scale‐Up Project**

^1^Kenya Medical Research Institute, Nairobi, Kenya, ^2^University of Washington, Seattle, United States, ^3^Jomo Kenyatta University of Agriculture and Technology, Juja, Kenya

Roll out of pre‐exposure prophylaxis (PrEP) has begun in several African countries. In Kenya, PrEP delivery is largely in public health facilities, which successfully provide services to large numbers of clients in spite of facing multiple challenges including understaffing, long waiting times, poor infrastructure, and commodity stock outs. The Partners Scale‐Up Project is an on‐going prospective implementation science evaluation that aims to catalyze integration of PrEP in 24 public HIV care clinics in Kenya using existing facility infrastructure and personnel. As of December 2018, participating clinics have initiated 4000 clients on PrEP. We describe how public clinics are adapting PrEP implementation guidelines to facilitate successful delivery.

To understand the service integration process, we conducted qualitative interviews with health providers and documented clinic observations in technical assistance (TA) reports over eighteen months. Using a combination of deductive and inductive approaches, we analyzed 71 health provider interviews and TA reports from the 24 clinics to identify clinic level adaptations to national PrEP implementation guidelines.

Clinics tried multiple adaptations to facilitate PrEP delivery. First, renal function testing is recommended (but not required) by Kenyan guidelines but due to unavailability of creatinine tests, clients were often initiated on PrEP without such testing if otherwise healthy. Second, to address long waiting times, almost all clinics reported fast‐tracking PrEP users. Third, some clinicians reported dispensing PrEP medication from the clinical room, saving PrEP users time associated with waiting at the pharmacy, a practice that also mitigated the stigma associated with being seen at a pharmacy that predominantly serves HIV infected persons. Fourth, while Kenyan guidelines recommend monthly refill appointments, the majority of clinics issued PrEP users 2 to 3 months of pills at a time, depending on client request and adherence; this adaptation also eased provider workload. Finally, when stock outs of tenofovir/emtricitabine (TDF/FTC) occurred, clinics readily dispensed tenofovir/lamivudine (TDF/3TC) as PrEP.

Health providers in public HIV care clinics instituted practices and made innovative adaptations to recommendations in order to overcome PrEP delivery challenges and reduce barriers for clients and staff. Enabling clinic level adaptations to national implementation guidelines will facilitate the scale‐up of PrEP delivery.

## WEPDC0205

### Training, communication, and prescribing patterns of pre‐exposure prophylaxis (PrEP) among a sample of nurse practitioners in the United States: Important gaps and opportunities

**M. Ellis^1,2^; S. Scroggins^3^; E. Shacham^3^ and K. Moore^3^**

^1^Saint Louis University, College for Public Health and Social Justice, Saint Louis, United States, ^2^Washington University School of Medicine, Saint Louis, United States, ^3^Saint Louis University, Saint Louis, United States

Currently, nurse practitioners (NPs) provide a significant proportion of primary healthcare, which is anticipated to increase amidst current and future expected shortages of primary care physicians. Given their prominence in delivering healthcare and prevention, it is important to know the extent to which NPs are trained, communicate on and prescribe the effective HIV prevention medication, pre‐exposure prophylaxis (PrEP), which is not well understood. The purpose of this study was to evaluate these factors in a national sample of NPs in order to identify gaps in the delivery of PrEP that may impact provision of care to persons at risk of HIV.

A cross‐sectional survey was completed by a national subset of NPs in attendance at their annual American conference (n = 271). Sociodemographics were collected in addition to a history of training on PrEP, communication of PrEP to patients, prescribing patterns, and associated self‐confidence. Descriptive statistics present the scope and magnitude of these PrEP‐related issues.

The majority of the sample identified as white (n = 214, 79%), female (n = 223, 82.3%), and middle age (46.0 + 11.34 years), with 16% (n = 44) identified as a sexual minority. Nearly two‐thirds of NPs (60.1%, n = 163) reported having no prior PrEP training or education. A similar proportion of NPs (62.4%) reported never initiating a conversation about PrEP. Only half of NPs reported being ‘confident’ in discussing PrEP efficacy with patients, discussing PrEP as an option with a patient, monitoring side effects of PrEP, or testing PrEP patients for HIV. In terms of prescribing practices, 66.8% of NPs reported that they were currently not prescribing PrEP to any patients.

Studies continue to find a lack of provider communication or promotion of PrEP to be a key barrier in its uptake. In our study, we found that the majority of NPs: 1) had no PrEP training/education PrEP; 2) had never initiated a conversation with a patient about PrEP; 3) had low confidence in delivering PrEP‐related care; and 4) had no patients being prescribed PrEP. Developing and delivering educational and training interventions for Nurse Practitioners and their institutions in international settings could serve to dramatically increase HIV preventative care.

## WEPDC0206

### Attitudes, confidence and knowledge towards antiretroviral pre‐exposure prophylaxis (PrEP) prescription among healthcare providers in Thailand

**R. Sirijatuphat^1^; P. Wisutep^1^; P. Phatharodom^2^; O. Navanukroh^2^; P. Werarak^2^ and W. Rattanasuwan^2^**

^1^Mahidol University, Department of Medicine, Faculty of Medicine Siriraj Hospital, Bangkok, Thailand, ^2^Mahidol University, Department of Preventive and Social Medicine, Faculty of Medicine Siriraj Hospital, Bangkok, Thailand

HIV pre‐exposure prophylaxis (HIV‐PrEP) is one of effective methods for preventing HIV transmission and is recommended for high risk populations. Attitudes, confidence and knowledge towards HIV‐PrEP prescription among healthcare providers have not been investigated in Thailand where HIV‐PrEP is a novel issue for the healthcare system.

A questionnaire‐based descriptive study was administered to Thai healthcare providers during May‐September 2018.

A total of 500 questionnaire paper survey was distributed. The reply rate was 92% (460 participants). The study participants included 336 physicians (48 infectious disease (ID) physicians and 288 non‐ID physicians) and 124 non‐physicians (70 nurses, 35 pharmacists and 19 others). Eighty‐one percent of participants had positive attitudes towards HIV‐PrEP. Non‐ID physicians, taking care < 100 HIV‐infected patients/month, having prior HIV‐PrEP knowledge, believing in HIV‐PrEP efficacy and believing that HIV‐PrEP is not associated with higher incidence of sexually transmitted infections were significant factors associated with positive attitudes towards HIV‐PrEP. The most concerning issue for participants who had negative attitudes towards HIV‐PrEP was the patient's adherence to antiviral drugs. Only 57% of participants had confidence to prescribe HIV‐PrEP. Factors associated with confidence to prescribe HIV‐PrEP were ID physicians, believing in HIV‐PrEP efficacy, believing in safety of antiviral drugs and believing that HIV‐PrEP is not associated with developing of HIV drug resistance. The results of knowledge testing about HIV‐PrEP by set of 8 questions were categorized into good score (≥7/8) and fair score (≤6/8). Fifty‐five percent of participants had a good score result. Factors associated with a good score result were ID physicians, having experience of HIV‐PrEP prescription and believing in HIV‐PrEP efficacy.

Most of Thai healthcare providers had positive attitudes towards HIV‐PrEP but the major concerning barrier was the patient's adherence to medication. Moreover, only about half of participants had confidence to prescribe HIV‐PrEP and a good score result in HIV‐PrEP knowledge testing. Successful HIV‐PrEP implementation in Thailand will require continuing education and improving experience of healthcare providers to strengthen knowledge and confidence to prescribe HIV‐PrEP.

## Late Breaking Abstracts

## MOAD0405LB

### Improving uptake for VMMC in traditional initiation settings

**K. Bellis^1^ and C. Bonnecwe^2^**

^1^Clinton Health Access Initiative, Pretoria, South Africa, ^2^National Department of Health, HIV Directorate, Medical Male Circumcision, Pretoria, South Africa

**Background: **Traditional male circumcision as a rite of passage is seen as critically important in some cultures in South Africa. It marks the right of passage from adolescence to manhood and occurs at ages between 15 and 20 years of age depending on the cultural group involved.

The study aimed at increasing the number of adolescent boys over a two year period who underwent initiation school training but opted to be circumcised by qualified medical practitioners and nurses to ensure the full removal of the foreskin as a HIV prevention measure.

**Methods: **The research was conducted on behalf of the National Department of Health (NDoH) by the Clinton Health Access Initiative (CHAI) and engaged accredited PEPFAR funded partners to offer circumcision services in collaboration with local chiefs, Traditional Leadership authorities and local traditional Fora representing medical professions within the traditional leadership. Data were collected and verified in accordance with national proscribed standards in Nkangala district where there were known to be initiation cycles taking place amongst adolescent males. Data focussed on the number of initiates in traditional camps and the number of initiates circumcised per year.

**Results: **Numbers were compared against agreed PEPFAR targets for each district over a two year period Financial years 2017 to 2018 and 2018 to 2019. The 2016 to 2017 financial year was used as a baseline to show the expansion in uptake of service.

Generally, initiation camps occur on a four year cycle and there were expectations that the numbers would decrease in a non initiation cycle year but instead have continued to climb.

Abstract MOAD0405LB‐Table 1. MMC undertaken in traditional initiation sites in Nkangala District

**Table:** 

Year	Annual Target	Annual Performance	Initiation Campaign Target	Initiation Campaign Performance
2016 to 2017 (baseline)	30,852	6188	0	3472
2017 to 2018	28,649	44,508	13,697	19,964
2018 to 2019	26,445	43,362	7645	39,777

**Conclusions: **Integration of traditional initiation and MMC techniques is possible.

Engagement of traditional and cultural leadership and gender sensitive medical staff is key to a successful sustained MMC programme based in traditional initiation schools.

There is potential to increase uptake of MMC overall in districts outside of the traditional initiation time cycles.

The provision of data collection teams that feed into the NDoH systems in initiation schools is key to monitoring and evaluating effective programmes.

## MOAX0101LB

### HPTN 078: primary results of a randomized study to engage men who have sex with men (MSM) living with HIV who are virally unsuppressed in the US

**R.H. Remien^1,2^, T. Gamble^3^, J.E. Farley^4^, Z. Wang^5^, C. Del Rio^6^, D.S. Batey^7^, K.H. Mayer^8,9^, C. Foster^10^, J. Glorioso^11^, W. Graves^8^, K.J. King^12^, S. Shurbaji^13^, I.C. Balán^1,2^, L. McKinstry^5^, V. Cummings^14^, S.H. Eshleman^14^, M. Stirratt^15^, A. Adeyeye^16^, J.P. Hughes^5,17^, C. Beyrer^18^ and for the HPTN 078 Study Team**

^1^NY State Psychiatric Institute, HIV Center for Clinical and Behavioral Studies, New York, United States, ^2^Columbia University, New York, United States, ^3^FHI 360, HPTN Leadership and Operations Center, Durham, United States, ^4^Johns Hopkins School of Nursing, The REACH Initiative, Baltimore, United States, ^5^Fred Hutchinson Cancer Research Center, Vaccine and Infectious Disease Division, Seattle, United States, ^6^Emory University School of Medicine, Atlanta, United States, ^7^University of Alabama at Birmingham, Department of Social Work, Birmingham, United States, ^8^The Fenway Institute, Boston, United States, ^9^Harvard Medical School, Boston, United States, ^10^Emory University, Rollins School of Public Health, Emory Center for AIDS Research, Atlanta, United States, ^11^Johns Hopkins University, The REACH Initiative, School of Nursing, Baltimore, United States, ^12^University of Alabama at Birmingham, Birmingham, United States, ^13^Washington State Department of Health, Seattle, United States, ^14^Johns Hopkins School of Medicine, Department of Pathology, Baltimore, United States, ^15^National Institute of Mental Health, Division of AIDS Research, Bethesda, United States, ^16^National Institutes of Health, Prevention Science Program, Division of AIDS, NIAID, Bethesda, United States, ^17^University of Washington, Department of Biostatistics, Seattle, United States, ^18^Johns Hopkins Bloomberg School of Public Health, Epidemiology, Baltimore, United States

**Background: **HIV infection in the United States (US) is increasingly concentrated among men who have sex with men (MSM), particularly MSM of color. To achieve the goals of “Ending the HIV Epidemic: A Plan for America,” everyone living with HIV, including MSM, must be identified, linked to care and supported so that they achieve and maintain viral suppression for their own health and to prevent onward HIV transmission.

**Methods: **MSM who were living with HIV and virally unsuppressed were recruited in four US cities (Birmingham, AL, Atlanta, GA, Baltimore, MD, and Boston, MA) and were randomized to either an enhanced case management (CM) intervention or standard of care (SOC). The CM intervention had three components: access to a CM and referral services, counseling using motivational interviewing techniques, and automated adherence and motivational messaging. Critically, participants determined intervention intensity by choosing frequency and content of CM interactions and automated messaging. Viral suppression (<200 copies/mL) across arms was compared at Month 12 using logistic regression. Those who did not provide a 12‐month sample (aside from deaths) were treated as unsuppressed.

**Results: **1305 MSM were screened; 154 were living with HIV and unsuppressed; 144 were enrolled. 91% were retained at Month 12. The enrolled cohort was 84% Black, 7% Latinx and the average age was 39. Most were educated (90%≥ high school diploma), but not employed (67%), with an income below $20,000 (64%). 81% had health insurance. The majority (86%) were ART experienced by self‐report. At baseline, the median viral load was 19,459 copies/mL, and at Month 12, 48% were virally suppressed, with no difference between the CM and SOC arms (OR = 0.615 [*p *=* *0.1526, 95% CI = 0.315, 1.197]).

**Conclusions: **HPTN 078 demonstrated that MSM living with HIV, but out of care, are willing to re‐engage when reached, with nearly half achieving and maintaining viral suppression at 12 months. The CM intervention did not, however, enhance viral suppression; half of the men, overall, were not virally suppressed at 12 months. Greater investment for more intensive interventions is likely needed to address the multiple societal and behavioral challenges among disenfranchised MSM in the US.

## MOAX0102LB

### Progressive rises in weight and clinical obesity for TAF/FTC/DTG and TDF/FTC/DTG versus TDF/FTC/EFV: ADVANCE and NAMSAL trials

**A. Hill^1^, W.F. Venter^2^, E. Delaporte^3^, S. Sokhela^2^, C. Kouanfack^4^, M. Moorhouse^2^, K. McCann^5^, B. Simmons^5^ and A. Calmy^6^**

^1^University of Liverpool, Translational Medicine, Liverpool, United Kingdom, ^2^University of Witwatersrand, Health Sciences, Johannesburg, South Africa, ^3^INSERM, University of Montpelier, Montpelier, France, ^4^Hôpital Central de Yaoundé, Hôpital Militaire, Site ANRS, Yaounde, Cameroon, ^5^Imperial College, Faculty of Medicine, London, United Kingdom, ^6^University Hospital and University Geneva, Geneva, Switzerland

**Background: **In previous clinical trials and cohort studies, dolutegravir (DTG) has been associated with rises in body weight and clinical obesity. These effects have been most pronounced in black people and women; the use of tenofovir disoproxil fumarate (TDF) is associated with lower body weight, compared to tenofovir alafenamide (TAF), abacavir or NRTI sparing treatment.

**Methods: **In the 96 week NAMSAL trial, 613 treatment naïve patients in Cameroun were randomised to TDF/3TC/DTG or TDF/3TC/EFV. Body weight was measured at baseline and Week 48. In the 96‐week ADVANCE trial, 1053 treatment naïve patients in South Africa were randomised to TAF/FTC/DTG, TDF/FTC/DTG or TDF/FTC/EFV. Body weight was measured at baseline and every 12 weeks; DEXA scans evaluated limb and trunk fat at baseline, Week 48 and Week 96. For both trials, changes in body weight, Body Mass Index (BMI), and trunk fat (ADVANCE only) were compared between treatments.

**Results: **In the NAMSAL trial, mean weight rose + 7.3% for TDF/3TC/DTG versus + 5.3% for TDF/3TC/EFV (*p *<* *0.001); treatment‐emergent clinical obesity (BMI > 30 kg/m^2^) was recorded for 12% on TDF/3TC/DTG versus 5% for TDF/3TC/EFV (*p *=* *0004). BMI rose + 1.7 kg/m^2^ for TDF/3TC/DTG versus + 1.2 kg/m^2^ for TDF/3TC/EFV. In ADVANCE (Results in Table), there were progressive, linear rises in body weight to Week 96 for women treated with TAF/FTC/DTG and TDF/FTC/DTG (Table 1); in men, mean body weight rose in the DTG arms to Week 48 and then stabilised to Week 96. Trunk fat also rose significantly in the TAF/FTC/DTG arm of ADVANCE.

**Conclusions: **In the NAMSAL and ADVANCE trials first‐line DTG is associated with rises in body weight, clinical obesity and increased trunk fat. These rises are higher if used in combination with TAF/FTC. Rises in body weight on TAF/FTC/DTG are progressive: longer‐term follow up and re‐analysis of other studies is required to evaluate clinical consequences.

Abstract MOAX0102LB–Table 1. ADVANCE trial – changes in weight, clinical obesity and trunk fat at Week 48 (96)

**Table:** 

Treatment arm	TAF/FTC/DTG	TDF/FTC/DTG	TDF/FTC/EFV
Change in weight, men to Wk 48 (96)	+5 kg (+5 kg)	+3 kg (+4 kg)	+1 kg (−2 kg)
Change in weight, women to Wk 48 (96)	+6 kg (+10 kg)	+3 kg (+5 kg)	+2 kg (+3 kg)
Clinical obesity, men, by Wk 48 (96)	+8% (0%)	+3% (0%)	+4% (0%)
Clinical obesity, women, by Wk 48 (96)	+20% (23%)	+10% (16%)	+7% (5%)
% Change in weight, men, to Wk 48 (96)	+7% (+7%)	+5% (+7%)	+1% (−2%)
% Change in weight, women, to Wk 48 (96)	+10% (+16%)	+5% (+9%)	+3% (+5%)
Change in Trunk fat, men, to Wk 48 (96)	+0.2 kg (+0.2 kg)	−0.3 kg (−0.2 kg)	−0.5 kg (−1.2 kg)
Change in Trunk fat, women, to Wk 48 (96)	+1.7 kg (+1.6 kg)	+0.5 kg (+1.1 kg)	0 kg (−0.7 kg)

## MOAX0103LB

### Comparison of pregnancy incidence among African women in a randomized trial of intramuscular depot medroxyprogesterone acetate (DMPA‐IM), the levonorgestrel (LNG) implant, and the copper intrauterine device (IUD)

**M. Onono^1^, K. Nanda^2^, K. Heller^3^, D. Taylor^2^, P. Gichangi^4^, R. Heffron^3^, M. Kasaro^5^, C. Louw^6^, C. Morrison^2^, N. Mugo^7^, Z. Nhlabatsi^8^, J.A. Smit^9^, I. Wakhungu^1^, I. Yakobson^2^, J.M. Baeten^3^ and on behalf of the ECHO Trial team**

^1^Kenya Medical Research Institute, Center for Microbiology Research, Kargeno Research and Policy Hub, Kisumu, Kenya, ^2^FHI360, Durham, United States, ^3^University of Washington, Seatte, United States, ^4^Technical University of Mombasa, Mombasa, Kenya, ^5^University of North Carolina Kamwala, Kamwala, Zambia, ^6^Madibeng Centre for Research, Madibeng, South Africa, ^7^Kenya Medical Research Institute, Center for Clinical Research, Nairobi, Kenya, ^8^Family Life Association of Eswatini, Manzini, Eswatini, ^9^MatCH Research Unit ^MRU^, University of the Witwatersrand, johannesburg, South Africa

**Background: **Sub‐Saharan Africa is disproportionately affected by high rates of unintended pregnancy. Contraceptive method failure is an important contributor to unintended pregnancy. Few data are available that compare pregnancy rates among different long acting contraceptive methods.

**Methods: **We analyzed data from the ECHO Trial, which assessed HIV incidence among 7829 women from 12 sites in Kenya, the Kingdom of Eswatini, South Africa, and Zambia who were seeking effective contraception and consented to be randomized to DMPA‐IM, LNG implant, or copper IUD. Cox proportional hazards regression adjusted for condom use during last vaginal sex was used to compare pregnancy incidence during both perfect use (from initiation of method until first discontinuation for any reason) and typical use (from initiation of method until decline or change to a different contraceptive method). Cumulative pregnancy probabilities at 12‐months were estimated using Kaplan‐Meier methods.

**Results: **7710 women contributed to this analysis. 70 pregnancies occurred during perfect use and 85 during typical use. Perfect use pregnancy incidence rates were 0.61 per 100 woman‐years (wy) for DMPA‐IM (95% CI 0.36 to 0.96), 1.06 for copper IUD (95% CI 0.72 to 1.50), and 0.63 for LNG implants (95% CI 0.39 to 0.96), with 12‐month cumulative probabilities of 0.62% (95% CI 0.37 to 1.03), 1.09% (95% CI 0.73 to 1.64), and 0.64% (95% CI 0.39 to 1.04), respectively. Typical use incidence rates were 0.87 per 100wy for DMPA‐IM (95% CI 0.58 to 1.25), 1.11 for copper IUD (95% CI 0.77 to 1.54), and 0.63 for LNG implants (95% CI 0.39 to 0.96) with 12‐month cumulative probabilities of 0.90% (95% CI 0.59 to 1.36), 1.05% (95% CI 0.71 to 1.57), and 0.64% (95% CI 0.39 to 1.04), respectively. Typical use of copper IUD was associated with statistically significant higher risk of pregnancy compared to LNG implants (aHR 1.74; 95% CI 1.01 to 2.99).

**Conclusions: **In a randomized contraceptive trial of African women, both perfect and typical use of all contraceptive methods resulted in low pregnancy rates. Although we found that women using copper IUD had somewhat higher pregnancy rates than those using LNG implants, our findings provide strong justification to improve access to a range of contraceptive options including LNG implants and copper IUD for African women.

## MOAX0104LB

### No occurrences of neural tube defects among 382 women on dolutegravir at pregnancy conception in Brazil

**G. Pereira^1^, A. Kim^2^, E. Jalil^3^, F. Fernandes Fonseca^4^, B. Shepherd^2^, V.G. Veloso^3^, F.M. Rick^1^, R. Ribeiro^1^, A. Beber^4^, K. Jayathilake^5^, R. Girade^1^, R. Lima^1^, F. Maruri^5^, A. Caruso^1^, F. Perini^1^, C. McGowan^5^, A. Benzaken^4^, J. Castilho^5^, B. Grinsztejn^3^ and The National Cohort Study of Dolutegravir Pregnancy Outcomes in Brazil**

^1^Ministry of Health – Brazil, Department of STI / AIDS / Viral Hepatitis of the Secretariat of Health Surveillance, Brasília, Brazil, ^2^Vanderbilt University Medical Center, Department of Biostatistics, Nashville, United States, ^3^Fiocruz, Instituto Nacional de Infectologia – Evandro Chagas, Rio de Janeiro, Brazil, ^4^Ministry of Health – Brazil, Department of Surveillance, Prevention and Control of STIs, HIV/aids and Viral Hepatitis, Brasília, Brazil, ^5^Vanderbilt University MOAX0104LB States

**Background: **In May 2018, a potential risk of neural tube defects (NTD) in infants born to HIV‐positive women exposed to dolutegravir (DTG) at pregnancy conception was announced. With widespread availability of DTG since 2016, Brazilian public health leaders initiated a national evaluation of birth outcomes of women exposed to DTG at conception.

**Methods: **Women who became pregnant while on ART containing DTG, efavirenz (EFV), or raltegravir (RAL) within ± 8 weeks from estimated date of conception (EDC) between January 2015 and May 2018 were identified using the Brazilian ART database. Every woman with DTG exposure and three women receiving EFV‐based antiretroviral therapy (ART) from similar locations were investigated. Trained personnel systematically collected demographic, medical, obstetric, radiographic, laboratory, and birth outcome data. The primary outcome was the occurrence of NTD; 95% Wilson confidence intervals (CI) were calculated. Characteristics at EDC and during antenatal care were evaluated.

**Results: **Overall, 1468 women were included, of which 382 were DTG‐exposed and 1086 EFV‐ and/or RAL‐exposed. At conception, DTG‐exposed women were slightly younger, more recently diagnosed with HIV, had lower CD4 cell counts, and were less likely to have virological suppression. During pregnancy, 48 and 45% of DTG‐exposed and ‐unexposed women, respectively, received folic acid supplementation. Prenatal syphilis occurred in 11% of DTG‐exposed women (vs. 6% in unexposed, *p *<* *0.01) and gestational hypertension occurred in 7% (vs. 3%, *p *<* *0.01). There were a total of 1493 birth outcomes (Table 1). There were no NTD in either exposure group (0 [95%CI 0, 0.0099] in DTG‐exposed and 0 [95%CI 0, 0.003] in DTG‐unexposed). Twenty‐five (6.5%) and 48 (4.3%) stillbirths/abortions occurred among DTG‐exposed and ‐unexposed fetuses, respectively (*p *=* *0.09).

**Conclusions: **DTG‐exposure was not associated with NTD in our cohort; the incidence of NTD is likely well under 1% among DTG‐exposed HIV‐positive women in Brazil. Ongoing pharmacovigilance of pregnancy outcomes from diverse settings is necessary for clarification of NTD risk associated with DTG.

Abstract MOAX0104LB‐Table 1. Birth outcomes by DTG exposure at conception

**Table:** 

	DTG exposed N = 384	DTG unexposed N = 1109	*p* value
Birth outcome, N (%)			0.01
Live birth	359 (93.5)	1061 (95.7)	
Stillbirth	2 (0.5)	16 (1.4)	
Abortion	23 (6.0)	32 (2.9)	
Neural tube defects	0 (0)	0 (0)	NA
Gestational age in weeks, median (IQR)	39 (38 to 39)	39 (38 to 39)	0.91
Birth weight in grams	3011 (2745 to 3310)	3050 (2741 to 3360)	0.39

## MOAX0105LB

### Neural tube defects by antiretroviral and HIV exposure in the Tsepamo Study, Botswana

**R. Zash^1,2,3^, L. Holmes^4^, M. Diseko^3^, D. Jacobson^5^, S. Brummel^5^, G. Mayondi^3^, A. Isaacson^3^, S. Davey^3,6^, J. Mabuta^3^, M. Mmalane^3^, T. Gaolathe^3,7^, M. Essex^1,3^, S. Lockman^1,3,8^ and R.L. Shapiro^1,3^**

^1^Harvard T.H. Chan School of Public Health, Boston, United States, ^2^Beth Israel Deaconess Medical Center, Boston, United States, ^3^Botswana Harvard AIDS Institute Partnership, Gaborone, Botswana, ^4^MassGeneral Hospital for Children, Boston, United States, ^5^Harvard T.H. Chan School of Public Health, Center for Biostatistics in AIDS Research, Boston, United States, ^6^University of Pennsylvania, Philadephia, United States, ^7^University of Botswana, Gaborone, Botswana, ^8^Brigham and Women's Hospital, Boston, United States

**Background: **We previously reported a preliminary safety signal associating dolutegravir exposure from conception and neural tube defects (NTDs), impacting antiretroviral choices for women of reproductive potential. Planned analysis of NTDs with data collected through March 2019 in the Tsepamo Study is now reported

**Methods: **We conducted birth outcomes surveillance at eight government hospitals throughout Botswana from 2014 to to 2018, expanding to 18 hospitals in 2018 to 2019. Trained midwives performed surface examinations of all live births and stillbirths and described abnormalities. Research assistants photographed major abnormalities after maternal consent, which were reviewed by a birth defects expert blinded to exposures. Prevalence of NTDs and major structural defects detectable by surface exam were determined by maternal HIV and antiretroviral exposure status (95%CI by Wilson method). The primary analysis evaluated prevalence differences by exposure status (95%CI by Newcombe method).

**Results: **From August 2014 through March 2019, 119,477 deliveries were captured in surveillance; 119,033 (99.6%) had an evaluable infant surface exam, with 98 (0.08%, 95%CI 0.07%, 0.10%) NTDs identified (60 with photo, 38 by description only). Among women on dolutegravir from conception, 5/1684 NTDs occurred (0.30%; 95%CI 0.13%, 0.69%): 2 myelomeningoceles, 1 anencephaly, 1 encephalocele, and 1 iniencephaly. In comparison, NTDs occurred in 15/14,792 (0.10%; 95%CI 0.06%, 0.17%) women delivering on any non‐dolutegravir antiretrovirals from conception, 3/7959 (0.04%; 95%CI 0.01%, 0.11%) on efavirenz from conception, 1/3839 (0.03%; 95%CI 0%, 0.15%) on dolutegravir started in pregnancy, 70/89,372 (0.08%; 95%CI 0.06, 0.10%) HIV‐uninfected women. NTD prevalence differed significantly between dolutegravir and any non‐dolutegravir antiretrovirals from conception (0.20% difference; 95%CI 0.01%, 0.59%), and for all other comparisons with dolutegravir (Table 1). Major structural defects were observed in 0.95% (95%CI 0.59, 1.54) of DTG‐conception exposures and 0.68% (95%CI 0.56, 0.83) of non‐dolutegravir exposures from conception (0.27% difference; 95%CI to 0.13, 0.87).

**Conclusions: **NTDs occurred in 3 per 1000 deliveries among women on dolutegravir from conception, a small but significant increase compared with all other antiretroviral exposures. Ongoing NTD surveillance in the Tsepamo Study is planned.

Abstract MOAX0105LB‐Table 1. Differences in Neural Tube Defect Prevalence by HIV‐ and ART‐exposure status

**Table:** 

Exposure group comparisons	% Prevalence difference (95% CI)
DTG at conception vs. Non‐DTG at conception	0.20% (0.01%,0.59%)
DTG at conception vs. EFV at conception	0.26% (0.07%,0.66%)
DTG at conception vs. DTG started in pregnancy	0.27% (0.06%,0.67%)
DTG at conception vs. non‐DTG started in pregnancy	0.25% (0.05%,0.64%)
DTG at conception vs. HIV‐uninfected	0.22% (0.05%,0.61%)

## MOAX0106LB

### Addressing the safety signal with dolutegravir use at conception: Additional surveillance data from Botswana

**M.M. Raesima^1^, S. Forhan^2^, V. Thomas^3^, E. Rabold^2^, M. Ogbuabo^4^, G. Gokatweng^4^, E. Dintwa^1^, C. Petlo^1^, M. Roland^3^, O. Mmunyane^1^, K. Kefitlhile^1^, T. Modise^1^, S. Malima^1^, S. Odunsi^5^, C. Motswere^3^, K. Dare^3^, M. Letebele^3^, S. Tinker^2^, C. Moore^2^, S. Modi^2^ and D. Williamson^2^**

^1^Botswana Ministry of Health and Wellness, Gaborone, Botswana, ^2^Centers for Disease Control and Prevention, Atlanta, United States, ^3^Centers for Disease Control and Prevention, Gaborone, Botswana, ^4^Botswana University of Maryland School of Medicine Health Initiative, Gaborone, Botswana, ^5^Peace Corps Botswana, Gaborone, Botswana

**Background: **In May 2018, the Botswana Tsepamo study reported a higher rate of neural tube defects (NTDs) among infants of women who were using dolutegravir (DTG)‐based antiretroviral treatment (ART) regimens at conception. The Botswana Ministry of Health and Wellness (MoHW) expanded NTD outcome surveillance in health facilities not covered by the Tsepamo study to capture additional pregnancies that were exposed to DTG at the time of conception.

**Methods: **Data on all deliveries (live births and stillbirths) at 22 facilities were collected from October 2018‐March 2019 including maternal HIV status, exposure to ART at conception, and infant birth outcomes. Potential NTDs were identified by midwives before infant discharge from the facility and suspected NTDs were reviewed and classified by a clinical geneticist who was blinded to HIV and ART exposure status.

**Results: **The surveillance system captured of 3076 pregnancies. Of these, 76% (n = 2328) pregnancies were among HIV‐negative women, 24% (n = 742) among HIV‐positive women, and < 1% (n = 6) among women with unknown HIV status. At conception, the majority (73%, n = 544) of HIV‐positive women were on ART; of these 28% (n = 152) were on DTG. (Table 1). Six suspected NTDs were identified. Of these, one NTD was confirmed, two were classified as not NTDs, two as “probably NTDs”, and one as a “possible NTD”. Three NTDs were included in the final analysis (1 confirmed, 2 probable). One of these NTDs occurred among the 152 DTG‐exposed HIV‐positive mothers for a prevalence of 0.66% (95% CI 0.02% to 3.69%; Table 2).The other two NTDs occurred in the 2328 HIV‐negative mothers for a prevalence of 0.09% (95% CI 0.01% to 0.31%).

**Conclusions: **Our findings suggest an increased birth prevalence of NTDs among infants born to HIV‐positive mothers on DTG at the time of conception when compared to infants born to HIV‐negative mothers, which is consistent with previous findings from the Tsepamo study.

The number of observed pregnancies in this study alone is too small to reliably detect differences in NTD prevalences that might exist. As such, these findings should be considered in combination with data from other studies to better assess any differential risks of NTDs associated with DTG.

The findings and conclusions in this report are those of the authors and do not necessarily represent the official position of the Centers for Disease Control and Prevention.

**Abstract MOAX0106LB‐Table 1. HIV status of the study population (n = 3076):**

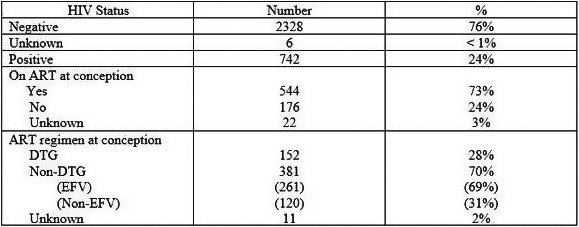

**Abstract MOAX0106LB‐Table 2. Neural tube defect (NTD) prevalence defference by maternal antiretroviral trestment (ART) exposure at conception**

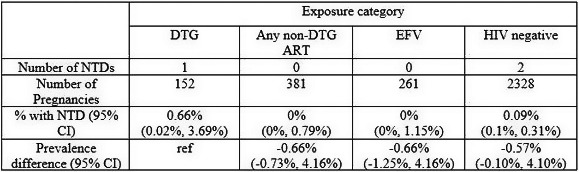

## MOSY0105LB

### Community‐led delivery of HIV self‐testing targeting adolescents and men in rural Malawi: a cluster‐randomised trial

**P.P. Indravudh^1,2^, K. Fielding^1^, M.K. Kumwenda^2^, R. Nzawa^2^, R. Chilongosi^3^, N. Desmond^2,4^, R. Nyirenda^5^, C.C. Johnson^6^, R. Baggaley^6^, K. Hatzold^7^, F. Terris‐Prestholt^1^ and E.L. Corbett^1,2^**

^1^London School of Hygiene & Tropical Medicine, London, United Kingdom, ^2^Malawi‐Liverpool‐Wellcome Trust Clinical Research Programme, Blantyre, Malawi, ^3^Population Services International Malawi, Lilongwe, Malawi, ^4^Liverpool School of Tropical Medicine, Liverpool, United Kingdom, ^5^Ministry of Health, Lilongwe, Malawi, ^6^World Health Organization, Geneva, Switzerland, ^7^Population Services International, Washington D.C., United States

**Background: **Community‐led interventions using participatory methods can ideally provide better outcomes at lower costs than conventional approaches. We conducted a cluster‐randomised trial evaluating community‐led HIV self‐testing (HIVST).

**Methods: **Thirty village‐head catchment areas in rural Mangochi, Malawi were randomised to community‐led HIVST or standard of care (SOC), including periodic community‐based testing. Participatory workshops and trainings supported planning and implementation of 7‐day HIVST campaigns by village health committees (VHC) and community volunteers. Volunteers receiving standardised gratuity (US$10) distributed HIVST kits, provided HIV prevention information and supported linkage to routine services.

The primary outcome was lifetime testing in adolescents (15 to 19 years). Secondary outcomes included recent testing (last three months) in men and older adults (≥40 years), mutual knowledge of status within sexual partners, knowledge of prevention methods, and antiretroviral therapy initiation (ongoing). Analysis compared cluster‐level outcomes by arm measured through post‐intervention surveys.

**Results: **From October 2018‐January 2019, 15 VHCs oversaw distribution by 188 volunteers of 24,347 kits. Post‐intervention surveys showed 74.4% of HIVST arm participants reporting self‐testing, with 2.3% testing positive and 0.39% pressured to self‐test.

Lifetime testing in adolescents was 84.6% versus 67.1% in HIVST and SOC arm (adjusted risk ratio (aRR) 1.25, 95%CI 1.10 to 1.43), with differences greatest for younger ages and males (Table). A higher proportion of males reported recent testing in the HIVST than SOC arm (74.5% versus 33.9%, aRR 2.21, 95%CI 1.92 to 2.55), with similar effects among older adults (74.2% versus 31.6%, aRR 2.37, 95%CI 2.00 to 2.80). Knowledge of status within couples was higher in the HIVST than SOC arm (71.3% versus 56.9%, aRR 1.24, 95%CI 1.08 to 1.42), but prevention knowledge did not differ.

**Conclusions: **Community‐led HIVST following participatory workshops and brief didactic training achieved high HIVST uptake, reaching more adolescents, men, older adults and couples and with minimal harm. Testing coverage was greater than recent community‐based HIVST models, supporting community‐led approaches as highly promising.

Abstract MOSY0105LB‐Table 1. HIV testing coverage by study arm

**Table:** 

	Community‐led HIVST arm % (n/N)	Standard of care arm % (n/N)	Risk difference (95% CI), *p*‐value	Risk ratio (95% CI), *p*‐value	Adjusted risk ratio (95% CI), *p*‐value
Total population surveyed	3974/30371 adults in 15 clusters	3906/25580 adults in 15 clusters			
Primary outcome: Adolescents 15 to 19 years ever tested	84.6% (773/914)	67.1% (579/863)	16.4% (7.8 to 25.0%), <0.001	1.26 (1.11 to 1.43), <0.001	1.25 (1.10 to 1.43), 0.001
Stratified by age: 15 to 17 years	79.8% (320/401)	57.2% (219/383)	22.3% (9.6 to 35.1%), 0.001	1.46 (1.15 to 1.86), 0.003	1.45 (1.14 to 1.85), 0.004
18 to 19 years	88.3% (453/513)	75.0% (360/480)	11.7% (4.5 to 18.9%), 0.002	1.16 (1.06 to 1.27), 0.002	1.16 (1.05 to 1.27), 0.004
Stratified by sex: Males	79.7% (310/389)	57.3% (217/379)	22.6% (12.1 to 33.1%), <0.001	1.42 (1.19 to 1.68), <0.001	1.40 (1.18 to 1.67), <0.001
Females	88.2% (463/525)	74.8% (362/484)	11.9% (2.8 to 21.0%), 0.01	1.18 (1.04 to 1.33), 0.01	1.18 (1.03 to 1.33), 0.01
Secondary outcome: Males tested in last three months	74.5% (1180/1584)	33.9% (504/1488)	40.7% (33.1 to 48.4%), <0.001	2.22 (1.92 to 2.56), <0.001	2.21 (1.92 to 2.55), <0.001
Secondary outcome: Adults ≥40 years tested in last three months	74.2% (871/1174)	31.6% (348/1103)	42.0% (34.5 to 49.5%), <0.001	2.36 (1.99 to 2.80), <0.001	2.37 (2.00 to 2.80), <0.001

Adjusted for sex, age, education level and marital status. *p*‐value for interaction by age group: 0.02. *p*‐value for interaction by sex: 0.01.

## TUAA0205LB

### Contribution of naïve CD4 + T cells to the intact HIV reservoir

**E. Venanzi Rullo^1,2^, L. Cannon^2^, M.R. Pinzone^2^, M. Ceccarelli^1,2^, G. Nunnari^1^ and U. O'Doherty^2^**

^1^University of Messina, Department of Clinical and Experimental Medicine, Messina, Italy, ^2^University of Pennsylvania, Department of Pathology and Laboratory Medicine, Philadelphia, United States

**Background: **Recent findings suggest that naïve CD4 + T cells (TN) may contain a significant amount of replication‐competent HIV DNA. We studied the contribution of different CD4 + T‐cell subsets to the reservoir in two male Caucasian subjects on ART at approximately two and nine years after ART initiation, both virologically suppressed at the time of the donations.

**Methods: **We sorted T CD4 + subsets from CD3 + CD8‐ PBMCs, defined as follows: naïve (CD45RA+, CCR7+, CD27+), central memory (CD45RA−, CCR7+, CD27+), transitional memory (CD45RA−, CCR7−, CD27+), and effector memory (CD45RA−, CCR7−, CD27−). Wemeasured total HIV DNA and sequenced 890 and 513 near full‐length proviruses for Subject 1 and 2, respectively.

**Results: **Among the subsets TN showed the highest percentage of intact proviruses. The contribution of TN to the intact reservoir was stable overtime, representing 38.5 and 34.7% for Subject 1 and 58.7 and 58.3% for Subject 2 at the two timepoints. The intact proviral sequences appeared to be mostly unique in TN while mostly clonal in effector memory cells (Figure 1).

**Conclusions: **TN appear to contribute significantly to the intact reservoir in two subjects followed on ART for nearly one decade.After reducing the clones, even a greater fraction of the reservoir was harbored in TN. Given their long half‐life and lower metabolic activity, the TN reservoir may represent a unique hurdle to HIV eradication. A limitation of our study is that the sorted TN population is a mixture of naïve, stem cell memory and T cells that have divided. In our opinion to sequence two individuals deeply at two time points provided us a better understanding of reservoir dynamics within subsets. Moreover, these individuals represent a spectrum of HIV infection. Subject 1 is a slow progressor with only CCR5‐tropic proviruses while Subject 2 is a rapid progressor with a majority of CXCR4‐tropic proviruses.

## TUAC0205LB

### Sitakhela Likusasa Impact Evaluation: results of a cluster randomized control trial (cRCT) of financial incentives for HIV prevention among adolescent girls and young women (AGYW) in Eswatini

**M. Gorgens^1^, S. Ketende^1^, V. Tsododo^2^, W. Heard^1^, M. Mabuza^3^, A. Longosz^1^, T. Chiperera^2^, L. Shongwe^2^, M. Sacolo^2^, M. Nkambule^3^, G. Maphalala^4^, L. Dlamini^5^, D. Wilson^1^, D. De Walque^1^, K. Mabuza^3^ and Sitakhela Likusasa Impact Evaluation Study Group**

^1^World Bank Group, Washington DC, United States, ^2^Institute for Health Measurement Southern Africa, Mbabane, Eswatini, ^3^National Emergency Response Council on HIV and AIDS (NERCHA), Mbabane, Eswatini, ^4^Ministry of Health (National Blood Bank), Mbabane, Eswatini, ^5^Ministry of Education, Science and Technology, Mbabane, Eswatini

**Background: **Eswatini still has the highest HIV prevalence globally, and very high HIV incidence among AGYW. Cash transfers linked to school attendance were protective against HIV (Baird et. al 2012), but subsequent studies have not shown impact on HIV incidence (Karim et al. 2015; Pettifor et al. 2016).

**Methods: **From Nov2015 to April2016, the Sitakhela Likusasa Impact Evaluation enrolled 4389 HIV‐negative AGYW aged 15 to 22 – 50% of whom were not in education – in a cRCT of periodic financial incentives for HIV prevention, with HIV incidence as the main outcome. Using a 2 × 2 factorial design to create 4 sub‐arms, 50% of participants were eligible for financial incentives conditional on education enrollment and attendance, and 50% were eligible for periodic raffle prizes conditional on periodic random selection into the raffle, on negative tests for syphilis and *Trichomonas vaginalis (TV)* if selected, and on being a periodic raffle winner. Education data were collected throughout. The endline survey, three years later, included behavioral and risk profile data, and HIV, syphilis and TV testing.

**Results: **HIV incidence among participants in the education incentive arm was statistically significantly lower compared to those not eligible for the education incentive, 6.34% vs. 8.08% (*p *=* *0.041); OR: 0.770 [0.599 to 0.989]; aOR: 0.751 [0.579 to 0.974]. HIV incidence in the sub‐arm offering both the education and raffle incentive was significantly lower than incidence in the control arm (participants not eligible for any of the two incentives), OR: 0.634 [0.442 to 0.910]; aOR: 0.618 [0.429 to 0.889].

**Conclusions: **The financial incentives conditional on education participation significantly reduced odds of HIV infection among AGYW in Eswatini. Raffle incentives on their own did not lead to a statistically significant impact, but it amplified the effect: the combination of both incentives statistically significantly further reduced the odds of HIV infection. Financial incentives can be useful for HIV prevention among AGYW in high prevalence settings.

Abstract TUAA0205LB‐Figure 1. Genetic evidence that naïve T cells contribute significantly to the replication competent reservoir. (a) The number of intact proviruses per million CD4 + T cells for both subjects was determined after two years on suppressive ART. Bt sorting PBMCs into naïve (green), central memory (blue), transitional and effector memory (yellow) and other subsets (gray), the number of intact proviruses in all the subsets were determined. Naïve T cells was a major contributor to the intact reservoir and the contribution was stable overtime. TN, Naïve CD4 + T cells; TCM, central memory CD4 + T cells.

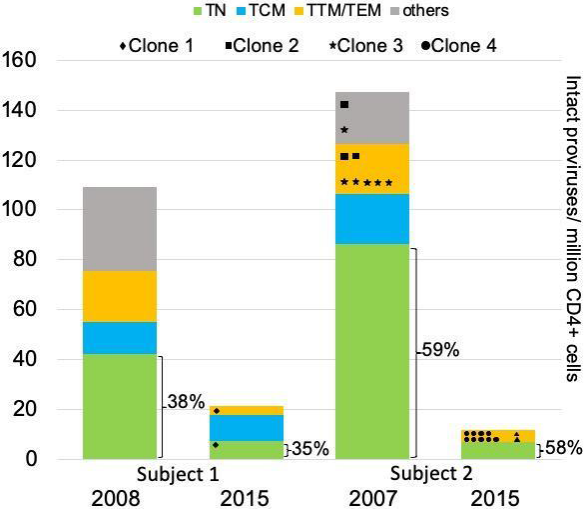

Abstract TUAC0205LB‐Table 1. Sitakhela Likusasa Impact Evaluation in Eswatini: HIV incidence by randomization arms and sub‐arms

**Table:** 

		HIV incidence over study period, % (n/N)	OR [95%CI]	P‐value	aOR1 [95% CI], adjusted for being in the raffle arm or the education arm	P‐value	aOR 2 [95% CI], being in school, rural‐urban residence, region of residence, age, attitude towards risk and assets based social economic status, as well as, in rows 3 and 5 only, for being in the raffle arm or the education arm, as relevant	*p*‐value
Education Incentive arm	*Education Incentive control*	8.08% (153/1894)	1		1		1	
	*Education Incentive treatment*	6.34% (119/1878)	0.770 [0.599 to 0.989]	0.041	0.766 [0.596 to 0.985]	0.037	0.751 [0.579 to 0.974]	0.031
Raffle incentive arm	*Raffle control*	7.85% (145/1851)	1		1		1	
	*Raffle treatment*	6.61% (127/1921)	0.833 [0.648 to 1.070]	0.147	0.827 [0.644 to 1.063]	0.137	0.823 [0.641 to 1.056]	0.126
Randomization sub‐arms	*Control*	8.84% (80/905)	1				1	
	*Raffle only*	7.38% (73/989)	0.822 [0.588 to 1.149]	0.252	n/a		0. 823 [0.589 to 1.149]	0.253
	*Education only*	6.87% (65/946)	0.761 [0.542 to 1.069]	0.115	n/a		0.751 [0.528 to 1.069]	0.112
	*Raffle and Education*	5.79% (54/932)	0.634 [0.442 to 0.910]	0.013	n/a		0.618 [0.429 to 0.889]	0.010
	TOTAL	7.21% (272/3772)						

## TUAC0305LB

### Frequent detection of tenofovir‐diphosphate among young Kenyan women in a real‐world PrEP implementation program

**J. Pintye^1^, J. Kinuthia^1,2^, F. Abuna^2^, K. Mugwanya^1^, H. Lagat^2^, J.C. Dettinger^1^, D. Odinga^2^, J. Sila^2^, J.M. Baeten^1,3^, G. John‐Stewart^1,3^ and PrEP Implementation for Young Women and Adolescents (PrIYA) Program**

^1^University of Washington, Department of Global Health, Seattle, United States, ^2^Kenyatta National Hospital, Nairobi, Kenya, ^3^University of Washington, Department of Medicine, Seattle, United States

**Background: **Programmatic PrEP delivery is scaling‐up in African settings with adolescent girls and young women as a priority population, yet few data exist on real‐world adherence. We evaluated tenofovir‐diphosphate (TFV‐DP) detection in dried blood spots (DBS) collected from young women in Kenya who initiated PrEP within routine maternal child health (MCH) and family planning (FP) clinics.

**Methods: **The PrEP Implementation in Young Women and Adolescent (PrIYA) Program was an implementation program providing PrEP integrated within MCH and FP clinics in Kisumu, Kenya. Between November 2017 and December 2018, women seeking MCH and FP services at 16 facilities were screened for behavioral risk factors and offered PrEP per national guidelines. Follow‐up visits were scheduled at 1‐month and then 3‐monthly post‐PrEP initiation. TFV‐DP levels were measured in DBS from a randomly selected subset of follow‐up visits among women continuing PrEP.

**Results: **Overall, 4376 women initiated PrEP: 90% from MCH and 10% from FP clinics. Median age was 24 years (IQR 21 to 28), 78% were married, and 8% had a known HIV‐positive partner. DBS were tested from 232 randomly selected follow‐up visits (5% of all visits) at a median of eight weeks (IQR 4 to 21) post‐initiation. Overall, 66% had detectable TFV‐DP with a median concentration of 535 fmol/punch (IQR 357 to 719). Almost all (62/65, 95%) samples among women with HIV‐positive partners had detectable TFV‐DP versus 68/109 (62%) for women with partners of unknown HIV status and 23/58 (40%) for women with HIV‐negative partners (*p *<* *0.001). Detectable TFV‐DP was less frequent among visits with pregnant women (52% pregnant vs. 70% non‐pregnant, *p *=* *0.019) and younger women (53% <24 years vs. 76% ≥24 years, *p *<* *0.001). One woman tested HIV‐positive during follow‐up; TFV‐DP was not detected in DBS collected at seroconversion.

**Conclusions: **In this programmatic evaluation of PrEP delivery to Kenyan women, two‐thirds of blood samples had detectable TFV‐DP. Women who had known HIV‐positive partners, were not pregnant, and were ≥24 years were more likely to have TFV‐DP detected, but the majority of women with partners of unknown status and those <24 years also had detectable TFV‐DP. These data suggest that PrEP programs for African women can achieve reasonable PrEP adherence.

## TUAC0401LB

### First‐in‐human trial of MK‐8591‐eluting implants demonstrates concentrations suitable for HIV prophylaxis for at least one year

**R.P. Matthews, S.E. Barrett, M. Patel, W. Zhu, K.L. Fillgrove, L. Haspeslagh, G. Chen, V. Levine, S. Zhang, A. Goodey, S.P. Forster, R. Vargo, J.A. Grobler, S.A. Stoch and M. Iwamoto**

Merck & Co, Kenilworth, United States

**Background: **Preexposure prophylaxis (PrEP) with antiretroviral drugs has demonstrated efficacy in reducing new HIV infections, although efficacy is tightly linked to good adherence, especially in women. HIV infection continues to be a global epidemic, however, with approximately 1.8 million new infections reported in 2018. MK‐8591 is a nucleoside reverse transcriptase translocation inhibitor with high potency, high barrier to resistance, long t1/2, and distribution to sites of HIV sexual transmission at levels comparable to those observed in PBMCs. In addition, MK‐8591 has demonstrated efficacy with weekly dosing in an SIV challenge prophylaxis study. Thus, MK‐8591 appears to have potential as an agent for PrEP.

**Methods: **Drug‐eluting implants were studied in preclinical species to establish general tolerability and pharmacokinetics (PK) of MK‐8591 parent and of the active MK‐8591‐TP (triphosphate). These data, along with data from the SIV challenge study and from previous Phase 1 trials, formed the basis of models for predicting long‐term exposures and for establishing a threshold concentration of 0.05 pmol/10^6^ TP in PBMCs. In a double‐blind placebo‐controlled Phase 1 trial, a single MK‐8591 (54 or 62 mg) or placebo implant was placed in subjects for 12 weeks. Safety and tolerability were assessed throughout the trial, and PK was collected for MK‐8591 parent and MK‐8591‐TP until four weeks after implant removal. Modeling was conducted to extrapolate and predict MK‐8591‐TP concentrations.

**Results: **Implants were generally well tolerated, and PK showed concentrations above target for both implants throughout the study. PK parameter values for MK‐8591‐TP and the projected duration of the implant above the target are depicted in the table.

Abstract TUAC0401LB‐Table 1. PK parameter values for MK‐8591‐TP and projected duration of the implant

**Table:** 

	Panel A	Panel B
MK‐8591 Implant Dose (mg)	54	62
N	6	6
Geometric Mean C85d (%GCV) (pmol/10^6^ cells)	0.135 (27.3)	0.272 (45.2)
Estimated mean C365d (pmol/10^6^ cells)	0.02	0.08
Projected duration range (months)	8 to 10	12 to 16

**Conclusions: **MK‐8591‐eluting implants provide drug release projected to be sufficient for HIV prophylaxis for at least one year. A PrEP implant could provide an attractive option for individuals in whom adherence to a daily PrEP regimen is challenging.

## TUAC0402LB

### ASCENT: Phase 2a, randomized, double‐blind, placebo controlled study evaluating safety and immunogenicity of two HIV‐1 prophylactic vaccine regimens comprising Ad26.Mos4.HIV and either clade C gp140 or bivalent gp140

**D.J. Stieh^1^, F. Tomaka^2^, C.A. Comeaux^1^, S. Nijs^3^, K. Callewaert^3^, J. Hendriks^1^, Z. Euler^1^, G.D. Tomaras^4^, G. Alter^5^, J. Kublin^6^, L. Corey^6^, J. McElrath^6^, E. Swann^7^, M. Robb^8,9^, N. Michael^8,9^, M. Marovich^7^, M.G. Pau^1^, D.H. Barouch^5,10^ and H. Schuitemaker^1^**

^1^Janssen Vaccines & Prevention B.V, Leiden, Netherlands, ^2^Janssen Research & Development, Titusville, United States, ^3^Janssen Research & Development, Beerse, Belgium, ^4^Duke Human Vaccine Institute, Duke University, Durham, United States, ^5^Ragon Institute of MGH, MIT and Harvard, Cambridge, United States, ^6^Vaccine and Infectious Disease Division, Fred Hutchinson Cancer Research Center, Seattle, United States, ^7^Vaccine Research Program, Division of AIDS, National Institutes of Health, Bethesda, United States, ^8^U.S. Military HIV Research Program, Walter Reed Army Institute of Research, Silver Spring, United States, ^9^Henry M. Jackson Foundation for the Advancement of Military Medicine, Bethesda, United States, ^10^Beth Israel Deaconess, Medical Center, Harvard Medical Center, Boston, United States

**Background: **Mosaic HIV‐1 antigens induce broad immune responses and aim to provide protection against diverse HIV‐1 strains. Heterologous vaccination regimens including Ad26 and gp140 have conferred significant protection in NHP, and were safe and immunogenic in humans.

To optimize breadth, and refine the vaccine composition, ASCENT assessed adding Mosaic1 gp140 to clade C gp140 in the regimen.

**Methods: **This study was conducted in adults in Kenya, Rwanda and the USA. Participants were randomized to Ad26.Mos4.HIV at weeks 0 and 12, and Ad26.Mos4.HIV and alum adjuvanted gp140 Env protein (250 µg clade C gp140 or bivalent clade C‐Mosaic1 gp140, each 125 µg) at weeks 24 and 48 or placebo.

Serious adverse events (AEs) were assessed throughout the study, unsolicited AEs until 28 days post‐each vaccination, and solicited AEs until seven days post‐each vaccination.

**Results: **152 healthy adults (18 to 50 years; 59% females) were vaccinated with Ad26.Mos4.HIV and clade C gp140 (n = 26), Ad26.Mos4.HIV and bivalent gp140 (n = 100) or placebo (n = 26). Active regimens were well tolerated (most AEs were mild/moderate; no serious AEs).

HIV Env‐specific binding antibody levels and subclass distribution showed both regimens induced binding and functional antibodies to all antigens tested (Figure 1). Clade C responses were not attenuated by replacing half the clade C dose with Mosaic1 gp140, while clade B responses improved (*p *<* *0.05).

At week 28, similar PTE Env ELISpot responses were observed, with medians of 444 and 452 SFU/10^6^ PBMC in bivalent or clade C groups, respectively. CD4 + (but not CD8 + ) T‐cell ICS responses increased to Mos1 gp120 peptides in the bivalent relative to clade C group (0.147% vs. 0.123% IL‐2 and/or IFNγ+ CD4 T‐cells, 81% vs. 50% response, respectively).

**Conclusions: **Both regimens were well tolerated and immunogenic. ASCENT supports using bivalent clade C‐Mosaic1 gp140 with Ad26.Mos4.HIV for expanded clade coverage in the phase 3 efficacy study trial starting in 2019.

Abstract TUAC0402LB‐Figure 1. *Geometric mean ratio (95% CI) of tetravalent clade c‐Mos/tetravalent clade C groups for Week 52*

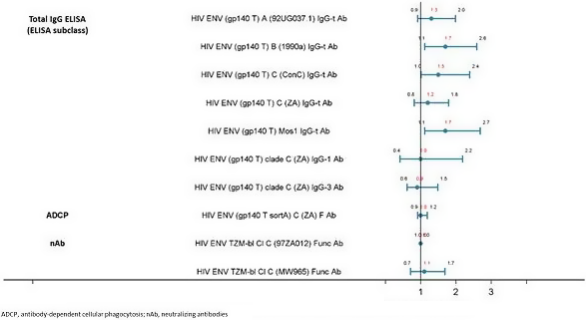

## TUAC0403LB

### DISCOVER study for HIV pre‐exposure prophylaxis (PrEP): F/TAF has a more rapid onset and longer sustained duration of HIV protection compared with F/TDF

**C.D. Spinner^1^, J. Brunetta^2^, P. Shalit^3^, M. Prins^4^, M. Cespedes^5^, D. Brainard^6^, M. Das^6^, S. McCallister^6^, F. Cruickshank^7^, J. Halperin^8^, J.E. Gladstein^9^ and P.W.G. Mallon^10^**

^1^Technische Universität München, Department of Medicine II, Munich, Germany, ^2^Maple Leaf Medial Clinic, Toronto, Canada, ^3^UWSOM, University of Washington, Seattle, United States, ^4^Public Health Service of Amsterdam (GGD), Department of Infectious Diseases Research and Prevention, Amsterdam, Netherlands, ^5^Icahn School of Medicine at Mount Sinai, Division of Infectious Disease, New York, United States, ^6^Gilead Sciences, Foster City, United States, ^7^University of Miami, School of Medicine, Deptartment of Infectious Diseases, Miami, United States, ^8^CrescentCare Health and Wellness Center, New Orleans, United States, ^9^Global Healthcare LA, Los Angeles, United States, ^10^School of Medicine, University College Dublin, Dublin, Ireland

**Background: **In DISCOVER, F/TAF was statistically noninferior to F/TDF. However, there were numerically 53% fewer HIV infections in the F/TAF arm vs. F/TDF. We explored HIV risk, STIs, adherence, and pharmacokinetic (PK) data to evaluate this imbalance.

**Methods: **In all participants (N = 5387), we compared HIV risk from computer‐aided self‐interview, lab‐assessed STIs, and adherence by pill count and self‐report. In a randomized subset, we measured adherence by intracellular TFV‐DP concentrations in peripheral blood mononuclear cells (PBMCs) (Week 4; n = 324) and in dried blood spots (DBS) (every 12 weeks; n = 309). We assessed the relationship between adherence (TFV‐DP in DBS) and efficacy using exact conditional logistic regression in a nested case‐control‐study; every incident HIV case paired with five controls matched by treatment arm, diagnosis date, rectal STI, and geography (cases = 22, controls = 109). We estimated duration of protection using PK data from historic Phase 1 studies to simulate TFV‐DP concentrations for TAF and TDF.

**Results: **There were no differences in HIV risk, STIs, or adherence by pill count or self‐report (N = 5387). Week 4 PBMC TFV‐DP levels were 6.3 fold higher with F/TAF vs. F/TDF; 98% of F/TAF versus 65% of F/TDF participants had TFV‐DP above the protective threshold (*p *<* *0.001). The median duration of protection after last dose at steady state was 60% longer for F/TAF vs. F/TDF. Low DBS TFV‐DP levels (adherence <2 doses/week) were associated with increased risk of HIV for F/TAF: odds ratio [OR] 29.4 and F/TDF: OR 13.2 (*p *<* *0.001 for both) with similar results from sensitivity analyses excluding suspected baseline infections.

**Conclusions: **Low TFV‐DP concentrations were associated with an increased risk of HIV acquisition.There was no difference in HIV risk or adherence between arms, but there were significant differences in TFV‐DP levels. TAF has advantageous PK parameters for HIV prevention compared to TDF including a more rapid, higher, and longer sustained duration of TFV‐DP levels above the protective threshold in PBMCs, which may explain the lower number of HIV infections in the F/TAF vs. F/TDF in DISCOVER.

Abstract TUAC0403LB‐Table 1.Key HIV prevention clinical pharmacology parameters: TFV‐DP with F/TAF and F/TDF

**Table:** 

Drug (Active Moiety)	TAF 25 mg (TFV‐DP)	TDF 300 mg (TFV‐DP)
Median (IQR) PBMC Ctau fmol/10^6^ cells (Ctau [trough] is the concentration at 24 hours post dose)	404 (226, 711)	61 (34, 105)
Protective Threshold in PBMCs (EC90) fmol/10^6^cells	40 (Anderson, 2012)	40 (Anderson, 2012)
Time to Protective Threshold	1 to 2 hours post single dose (historic Phase 1 data from GS‐US‐380‐4017, Schwartz, 2018)	3 to 4 days (Anderson, 2012)
Median Duration of Protection after Last Daily Dose (after steady state achieved)	16 days	10 days

## TUAC0404LB

### Safety, early continuation and adherence of same day PrEP initiation among MSM and TGW in Brazil, Mexico and Peru: the ImPrEP Study

**V.G. Veloso^1^, E.H. Vega‐Ramírez^2,3^, B. Hoagland^1^, K.A. Konda^4^, S. Bautista‐Arredondo^5^, J.V. Guanira^6^, R. Leyva‐Flores^5^, C. Pimenta^7^, M. Benedetti^1^, P.M. Luz^1^, I.C. Leite^1^, R.I. Moreira^1^, B. Grinsztejn^1^, C.F. Cáceres^4^**

^1^National Institute of Infectious Diseases Evandro Chagas (INI‐Fiocruz), Rio de Janeiro, Brazil, ^2^National Institute of Psychiatry Ramon de la Fuente Muñiz, Mexico City, Mexico, ^3^Condesa Specialized Clinic, Mexico City, Mexico, ^4^Centro de Investigación Interdisciplinaria en Sexualidad, Sida y Sociedad, Universidad Peruana Cayetano Heredia, Lima, Peru, ^5^National Institute of Public Health, Mexico City, Mexico, ^6^Investigaciones Medicas en Salud, Lima, Peru, ^7^Departament of Transmissible Diseases of Chronic Conditions and Sexually Transmitted Infections‐MoH, Brasília, Brazil

**Background: **The HIV epidemic in Latin America persists unabated among men who have sex with men (MSM) and transgender women (TGW). Pre‐exposure prophylaxis (PrEP) implementation in the region is still very limited. Same day PrEP initiation can increase PrEP uptake where access to health care services for key populations is scarce. ImPrEP is an ongoing demonstration study that aims to assess safety and feasibility of same day PrEP (daily oral tenofovir+emtricitabine [TDF/FTC]) for MSM and TGW at high risk for HIV infection in Brazil, Peru and Mexico. We herein report results on same day PrEP initiation, safety, early continuation and adherence.

**Methods: **MSM and TWG eligible for recruitment (i.e. HIV uninfected, ≥18 years old, reporting 1 + risk criteria) were screened and enrolled on the same day and received a 30‐day supply of TDF/FTC. Creatinine, hepatitis B, C and STI testing were performed. Main outcomes were PrEP early continuation defined as attendance to the first 2 follow‐up visits within 120 days of PrEP initiation; and PrEP adherence using pharmacy refill data defined as having at least 16 days of PrEP medication filled per 30‐day period (medication possession ratio ≥ 0.53).

**Results: **From February 2018 until April 2019, 4954 individuals were enrolled in Brazil (3205), Peru (1010) and Mexico (739), accumulating 1329.6 person‐years of PrEP use. Median age was 29 years (IQR 24 to 36); 94% (4648/4954) were MSM and 6% (306/4954) TGW; 44 (1.1%) had an eGFR < 60 mL/minute. Baseline active syphilis, rectal chlamydia and rectal gonorrhea prevalence were, respectively, 9.9% (95%CI: 9.0 to 10.8), 11.7% (95%CI: 10.7 to 12.7) and 7.4% (95%CI: 6.6 to 8.2). Overall, early continuation was achieved by 79.8% of participants and PrEP adherence was 96.9%. Early continuation was significantly lower among TGW, 56% (OR = 0.29; CI 95%: 0.21 to 0.40), and young MSM, 71 %(OR = 0.52;CI 95%: 0.40 to 0.67).HIV incidence was 0.8 per 100 person years (CI 95% 0.4 to 1.4).

**Conclusions: **Our study offers evidence that same day PrEP initiation in Latin America is feasible and safe, with overall good levels of early continuation and adherence. TGW and young MSM may require differentiated care to improve PrEP continuation.

## TUAD0305LB

### Achieving low antiretroviral (ARV) costs through competitive national tendering and strategic reference pricing: Results from the Republic of South Africa's recently awarded ARV tender (RT71‐2019)

**Y. Pillay^1^, H. Musariri^2^, R. Borain^2^, J. Quevedo^2^ and A. Pillay^3^**

^1^National Department of Health, HIV/AIDS Programme, Pretoria, South Africa, ^2^Clinton Health Access Initiative (CHAI), Pretoria, South Africa, ^3^National Department of Health, Pretoria, South Africa

**Background: **The Republic of South Africa (RSA) has the largest national HIV program in the world, with over four million patients on treatment and is the most significant single country procurer of ARVs globally. Given its significant HIV burden, national ownership of funding ARVs, and limited funding envelope, RSA relies heavily on efficient national tendering to ensure cost savings through low prices, while ensuring access to optimal ARVs for patients.

**Methods: **In August 2018, RSA advertised RT71‐2019, a three year ARV tender with effective dates from July 1, 2019 through July 31, 2020. RT17‐2019 included 147 million packs of product. For the first time, included within the advertised tender, were ceiling reference prices for ARVs. As an example, RSA employed the innovative pricing agreement for the new, best‐in‐class product “TLD” at $75 per patient per year. To be competitive, suppliers would be required to bid at or below these prices.

**Results: **In February 2019, RSA released the awards for RT71‐2019. For TLD, the focal ARV for optimizing treatment, the weighted‐average price per year for the product was ˜$62, a 17% decrease from the global benchmark. On commodities costs alone, when compared to the previous tender, RSA is expected to save over $530 million, which will allow RSA treat an estimated additional two million patients.

**Conclusions: **As national governments start transitioning away from global donor resources for funding ARVs, it is imperative for them to employ efficient national tendering mechanisms. RSA's approach for tendering, through use of global reference prices and transparent pricing system, could be adopted elsewhere to ensure access to optimal ARVs at affordable prices.

Abstract TUAD0305LB‐Table 1. Total estimated savings due to South Africa's domestic tendering system and ceiling price agreement

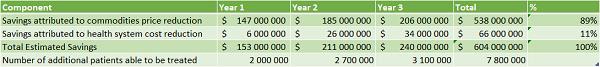

## WEAA0205LB

### Early ART initiation in acute HIV infection generates long‐lived memory HIV‐specific CD8^+^ T cells endowed with efficient proliferative and cytolytic recall response

**H. Takata^1,2^, J. Kakazu^1,2^, E. Kroon^3^, D.J. Colby^3^, C. Sacdalan^3^, S. Buranapraditkun^4^, S. Pinyakorn^1,2^, N. Michael^1,2^, M. Robb^1,2^, J. Intasan^3^, S. Tipsuk^3^, D. Suttichom^3^, P. Prueksakaew^3^, T. Chalermchai^3^, N. Phanuphak^3^, J. Ananworanich^1,2,3^, L. Trautmann^1,2^ and RV254/SEARCH010 and SEARCH011 study groups**

^1^Henry M. Jackson Foundation for the Advancement of Military Medicine, Bethesda, United States, ^2^U.S. Military HIV Research Program, Walter Reed Army Institute of Research, Silver Spring, United States, ^3^SEARCH, The Thai Red Cross AIDS Research Centre, Bangkok, Thailand, Bangkok, Thailand, ^4^Chulalongkorn University, Bangkok, Thailand

**Background: **ART initiation before peak viremia during acute HIV infection (AHI) partially preserves B and T cell responses, and memory potential of HIV‐specific CD8^+^ T cells. However whether these memory responses can be recalled efficiently is unknown. Here, we compared the proliferation and cytolytic capacity of memory HIV‐specific CD8^+^ T cells after ART initiation in AHI or in chronic HIV infection (CHI).

**Methods: **PBMCs from 30 RV254 Thai participants treated in AHI (PTAHI, Fiebig I‐V; median of 2.1 years of ART) and eight participants treated in CHI (PTCHI, median of 1.8 years of ART) were analyzed. Phenotype of HIV‐specific CD8^+^ T cells was analyzed by flow cytometry using tetramer staining. Proliferation was assessed after peptide stimulation at six and twelve days. Cytotoxicity was measured by co‐culturing the PBMCs at day 13 post‐stimulation with autologous peptide‐pulsed CD4^+^ T cells as targets.

**Results:** *Ex vivo* memory HIV‐specific CD8^+^ T cells were at lower frequency in PTAHI (Figure A), but exhibited more stem cell‐like (Tscm) and central memory (CM) phenotype compared with PTCHI (Figure B). After stimulation, their magnitude of expansion was significantly higher in PTAHI than in PTCHI (Figure C). This expansion positively correlated with the *ex vivo* expression of the IL‐7 receptor, transcription factor TCF‐1, and percentages of Tscm and CM. Recalled HIV‐specific CD8^+^ T cells showed significantly higher cytolytic capacity in PTAHI than in PTCHI (Figure D). PD‐1 expression levels were higher over the course of culture in PTCHI compared to PTAHI. The strong cytolytic capacity correlated with the magnitude of expansion and lower expression of PD‐1.

**Conclusions: **Early ART in AHI promotes differentiation of long‐lived memory CD8^+^ T cells that have higher expansion and cytotoxic capacity after recall than those in PTCHI. These data suggest that HIV remission strategies in PTCHI will likely require a combination of reversing T cell exhaustion and boosting the HIV‐specific CD8^+^ T cell response.

Abstract WEAA0205LB‐Figure 1. Ex vivo and recalled memory HIV‐specific CD8 + T cells

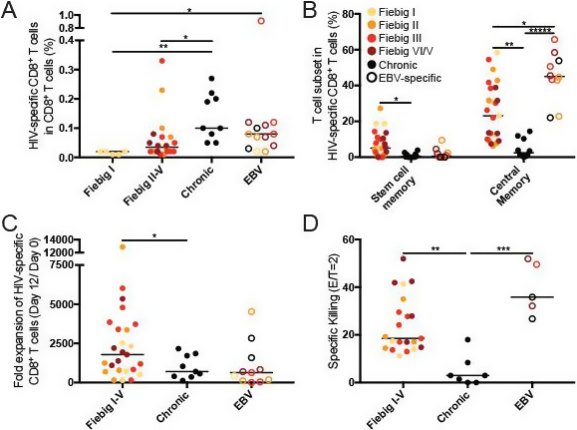

## WEAA0305LB

### Safety and virologic effect of the HIV‐1 broadly neutralizing antibodies, VRC01LS or VRC07‐523LS, administered to HIV‐infected adults in a phase 1 clinical trial

**G. Chen^1^, E. Coates^1^, C. Fichtenbaum^2^, S. Koletar^3^, R. Landovitz^4^, R. Presti^5^, T. Overton^6^, J. Santana^7^, R.S. Rothwel^1^, J. Roa^8^, E. Donaghy^9^, L. Holman^1^, L. Novik^1^, N. Berkowitz^1^, B. Larkin^1^, M. Conan‐Cibotti^1^, R. Tressler^10^, J. Wang^11^, Z. Hu^11^, E. Capparelli^12^, F. Arnold^1^, R. Bailer^1^, A. McDermott^1^, L. Gama^1^, B. Graham^1^, R. Koup^1^, J. Mascola^1^, J. Ledgerwood^1^, P. Tebas^9^ and VRC 607/ACTG A5378 study team**

^1^Vaccine Research Center, NIAID, NIH, Bethesda, United States, ^2^University of Cincinnati, Cincinnati, United States, ^3^Ohio State University CRS, Division of Infectious Diseases, Columbus, United States, ^4^University of California, Los Angeles, Center for Clinical AIDS Research & Education Center CRS, Los Angeles, United States, ^5^Washington University School of Medicine, Division of Infectious Disease, St. Louis, United States, ^6^Alabama CRS, Alabama Vaccine Research Clinic at UAB, Birmingham, United States, ^7^University of Puerto Rico School of Medicine, Proyecto ACTU‐Infectious Disease Section, San Juan, United States, ^8^Social and Scientific Systems, Silver Spring, United States, ^9^Hospital of University of Pennsylvania, Clinical Research Site, Philadelphia, United States, ^10^National Institute of Allergy and Infectious Diseases, National Institutes of Health, HIV Research Branch, Therapeutic Research Program, Division of AIDS, Rockville, United States, ^11^National Institute of Allergy and Infectious Diseases, National Institutes of Health, Biostatistics Research Branch, Division of Clinical Research, Bethesda, United States, ^12^University of California San Diego, School of Medicine and Skaggs School of Pharmacy and Pharmaceutical Sciences, San Diego, United States

**Background: **VRC 607/ACTG 5378 is a two‐part study conducted by the VRC and ACTG investigating the CD4 binding site HIV‐1 broadly neutralizing antibodies (bNAbs) VRC01LS (Part A) and VRC07‐523LS (Part B) in treatment naïve HIV‐infected adults. The study objectives were to evaluate safety and tolerability (primary) and antiviral activity and pharmacokinetic (PK) parameters (secondary).

**Methods: **16 HIV‐infected viremic adults between the ages of 18 to 70 were enrolled. Seven participants in Part A received one intravenous (IV) dose of 40 mg/kg VRC01LS, and nine participants in Part B received one IV dose of 40 mg/kg VRC07‐523LS. Safety was evaluated by collection of local and systemic reactogenicity symptoms for three days and adverse events (AEs) for 56 days post product administration. Blood samples were collected at pre‐specified study timepoints for viral load and PK analysis as well as CD4/8 T‐cell counts and safety labs. The study is ongoing at the time of this report.

**Results: **In Part A, VRC01LS was safe and well tolerated. No local reactogenicity was reported and systemic reactions (n = 1) were mild. There were no AEs related to the study product. Seven days post‐infusion, 3/7 participants had at least a 0.5 log10 decrease in viral load (Figure 1A). In Part B, VRC07‐523LS was safe and well tolerated. Local (n = 3) and systemic (n = 2) reactogenicity were mild. Two AEs were assessed as related to the study product; infusion site paraesthesia and decreased neutrophil count, both which resolved with no residual effects the day of infusion and eight days following onset, respectively. Seven days post‐infusion, 8/9 participants had at least 1.2 log10 decrease in viral load (Figure 1B).

**Conclusions: **Both VRC01LS and VRC07‐523LS were safe and well‐tolerated when administered to viremic HIV‐infected adults. Antiviral activity >0.5 log was observed in 3/7 VRC01LS recipients and >1.2 log for 8/9 VRC07‐523LS recipients.

Abstract WEAA0305LB‐Figure 1. 14‐day post‐infusion HIV viral load (copies/mL) for 40 mg/kg IV VRC01LS (Panel A) and 40 mg/kg IV VRC VRC07‐523LS (Panel B). Red arrow indicates product administration.

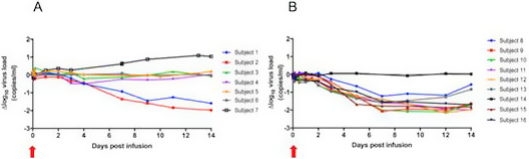

## WEAB0401LB

### Pharmacokinetics of dolutegravir 5 mg dispersible tablets in children weighing 6 to <20 kg dosed using WHO weight bands

**H. Waalewijn^1^, P.D.J. Bollen^1^, C. Moore^2^, A. Kekitiinwa^3^, P. Amuge^3^, H. Mujuru^4^, E. Chidziva^4^, V. Musiime^5^, E. Kaudha^5^, A. Lugemwa^6^, S. Makumbi^6^, A. Violari^7^, E. Variava^8^, S. Ali^2^, C. Giaquinto^9^, P. Rojo^10^, A. Colbers^1^, D. Gibb^2^, D. Ford^2^, A. Turkova^2^, D. Burger^1^ and the ODYSSEY trial team**

^1^Radboud University Medical Center /Radboud Institute for Health Sciences, Pharmacy, Nijmegen, Netherlands, ^2^University College London, Medical Research Council Clinical Trials Unit, London, United Kingdom, ^3^Baylor College of Medicine, Kampala, Uganda, ^4^University of Zimbabwe Clinical Research Centre, Harare, Zimbabwe, ^5^Joint Clinical Research Centre, Kampala, Uganda, ^6^Joint Clinical Research Centre, Mbarara, Uganda, ^7^Klerksdorp‐Tsepong Hospital Complex, Matlosana, South Africa, ^8^Chris Hani Baragwanath Hospital, Perinatal HIV Research Unit (PHRU), Soweto, South Africa, ^9^University of Padova, Padova, Italy, ^10^Hospital 12 de Octubre, Madrid, Spain

**Background: **Dolutegravir (DTG) 5 mg dispersible tablets (DT) are small, child‐friendly and allow easy scaling. We describe a pharmacokinetic (PK) substudy of DT DTG in children weighing 6to <20 kg dosed by WHO weight bands (WB) conducted within ODYSSEY, an ongoing phase‐III trial of DTG (NCT02259127).

**Methods: **Children weighing 6to <10 kg, 10 to<14 kg, 14 to<20 kg received DTG 5 mg DT at 15, 20, and 25 mg QD, respectively. At steady‐state, 24‐hour PK profiles (seven samples) were constructed after DTG intake. DTG plasma concentrations were measured by UPLC‐MS/MS; Phoenix64 was used for non‐compartmental analysis. Results were compared to historical PK parameters from adults taking 50 mg DTG filmcoated tablets (FCT) QD or BID, previous ODYSSEY PK data and published data from IMPAACTP1093.

**Results: **28 children [29 PK curves] from Zimbabwe and Uganda were included in the analysis. PK results (Table 1) from WBs 10 to <14 kg and 14 to <20 kg were similar to PK data from children receiving same DT doses in IMPAACTP1093, and GM Ctrough values were similar to children 20 to <40 kg in ODYSSEY on DTG FCT 50 mg and adults on 50 mg DTG FCT QD. In children 14 to <20 kg, exposures were ˜1.8 to 2‐fold higher on 25 mg DT than 25 mg FCT, similar to relative bioavailability of DT/FCT in adults. Our data from the 6 to <10 kg WB showed lower GM exposure to DTG compared to IMPAACTP1093 with high variability, and 3 of 8 children had observed Ctrough below EC90 (0.32 mg/L).

**Conclusions: **DTG DT in children weighing 10 to <20 kg, dosed QD in WHO WBs achieves similar Ctrough to adults and older children on the adult DTG dose and young children on DT in IMPAACTP1093. Some children in the 6to <10 kg WB had Ctrough levels below EC90, and PK profiles showed high variability in this WB. Further PK data collection in children 3 to <10 kg is ongoing and all children are followed up for safety.

Abstract WEAB0401LB‐Table 1

**Table:** 

	Odyssey Lower weightband PK substudies			Reference adults	
WHO weightband	6 to <10 kg	10 to <14 kg	14 to <20 kg	≥40 kg	≥40 kg/50 mg FCT BID
DTG dose and (N)	15 mg DT (8)	20 mg DT (8)	25 mg DT (13)	50 mg FCT (10)^a^	50 mg FCT BID (12^b^; 24^c^)
Age (years)	1.3 (0.6 to 3.0)	3.0 (1.6 to 4.2)	6.0 (4.9 to 8.5)	34 (22 to 53)	48 (31 to 59)^b^; 47 (33 to 68)^c^
Weight (kg)	7.6 (6.7 to 9.7)	11.5 (10.0 to 12.6)	18.0 (14.9 to 19.9)	‐	‐
Dose/weight (mg/kg)	2.0 (1.5 to 2.2)	1.7 (1.6 to 2.0)	1.4 (1.3 to 1.7)	‐	‐
Ctrough (mg/L); %below EC90	0.43 (207); 37.5%	0.77 (62); 0%	0.85 (67); 0%	0.83 (26)^a^	2.41 (77)^b^; 2.72 (70)^c^
AUC0‐24 h (mg*h/L)	46.3 (90)	76.0 (25)	69.6 (30)	43.4 (20)^a^	92.7 (55)^b^; 93.4 (50)^c^
Cmax (mg/L)	5.3 (58)	8.0 (25)	7.1 (21)	3.3 (16)^a^	5.6 (49)^b^; 5.4 (40)^c^

Pharmacokinetic parameters are expressed as geometric mean with coefficient of variation (%), median (range) for age, weight and dose/weight. Doses represent once daily doses, unless otherwise specified. FCT, film‐coated tablet; DT, dispersible tablet; BID, twice daily. ^a^Fasted HIV‐positive adults. ^b^Fasted healthy HIV‐negative adults. ^c^HIV‐positive treatment experienced adults, fed state not specified.

## WEAB0402LB

### MK‐8591 at doses of 0.25 to 2.25 mg QD, in combination with doravirine establishes and maintains viral suppression through 48 weeks in treatment‐naïve adults with HIV‐1 infection

**J.‐M. Molina^1^, Y. Yazdanpanah^2^, A. Afani Saud^3^, C. Bettacchi^4^, C. Chahin Anania^5^, E. DeJesus^6^, S.O. Klopfer^7^, K. Eves^7^, A. Grandhi^7^, M.N. Robertson^7^, C. Hwang^7^, G. Hanna^7^ and P. Sklar^7^**

^1^St‐Louis Hospital and University, Department of Infectious Disease, Paris, France, ^2^Bichat Hospital, Paris, France, ^3^University of Chile, Santiago, Chile, ^4^North Texas Infectious Diseases Consultants, Dallas, Texas, ^5^Hospital Hernán Henríquez Aravena of Temuco, Temuco, Chile, ^6^Orlando Immunology Center, Orlando, United States, ^7^Merck & Co, Kenilworth, United States

**Background: **MK‐8591 is the first nucleoside reverse transcriptase translocation inhibitor (NRTTI) in development for treatment of HIV‐1 infection. Doravirine (DOR) is a recently approved non‐nucleoside reverse transcriptase inhibitor (NNRTI). We present efficacy and safety data for MK‐8591 with DOR through 48 weeks.

**Methods: **Phase 2B, randomized, double‐blind, comparator‐controlled, dose‐ranging trial to evaluate efficacy and safety of MK‐8591 with DOR. For the first 24 weeks, equal proportions of participants received one of three doses of MK‐8591 (0.25 mg, 0.75 mg, or 2.25 mg) plus DOR (100 mg) and 3TC (300 mg) or DOR/3TC/TDF once daily with placebo. After 24 weeks of treatment, participants taking MK/8591 who achieved HIV‐1 RNA < 50 copies/mL switched to a two‐drug regimen of MK‐8591 and DOR. Efficacy endpoints included the overall proportion of participants at week 48 with HIV‐1 RNA < 50 copies/mL using the FDA snapshot approach. Protocol‐defined virologic failure (PDVF) was defined as rebound with confirmed HIV‐1 RNA ≤ 50 copies/mL after suppression or non‐response with confirmed HIV‐1 RNA ≥ 50 copies/mL by week 48. Safety was assessed by adverse event (AE) reporting.

**Results: **121 participants received drug and were included in analyses (mean age 31 yr, 92.6% male, 76.0% white, 22% HIV‐1 RNA>100,000 copies/ml). At week 48, 89.7% (26/29), 90.0% (27/30), 77.4% (24/31), of participants achieved HIV‐1 RNA<50 copies/mL in the 0.25mg, 0.75mg, 2.25mg dose of MK‐8591 respectively, compared to 83.9% (26/31) with DOR/3TC/TDF. The mean change in CD4^+^ T‐cell count from baseline to week 48 was similar for all groups. The proportion of participants on the 2‐drug regimen for 24 weeks with HIV‐1 RNA < 50 copies/mL was similar across doses (88.9%‐90.0%). Six participants by week 48 met the definition of PDVF, 5/90 (5.6%) in the MK‐8591 groups (4 rebound, 1 non‐response) and 1/31(3.2%) in the DOR/3TC/TDF group (rebound); none had HIV‐1 RNA>200 copies/mL or documented resistance to study drugs. No serious drug‐related AEs were reported by MK‐8591 participants. A higher rate of drug‐related AEs was reported for DOR/3TC/TDF (19.4%) participants compared with any of the doses of MK‐8591 (combined 7.8%).

**Conclusions: **Similar proportion of participants achieved and maintained viral suppression at week 48 across all treatment groups. MK‐8591+DOR was well tolerated regardless of dose.

## WEAB0403LB

### Switching to DTG + 3TC fixed dose combination (FDC) is non‐inferior to continuing a TAF‐based regimen (TBR) in maintaining virologic suppression through 24 weeks (TANGO Study)

**J. van Wyk^1^, F. Ajana^2^, F. Bisshop^3^, S. De Wit^4^, Y. Osiyemi^5^, J. Portilla^6^, J.‐P. Routy^7^, C. Wyen^8^, M. Ait‐Khaled^1^, M. Nascimento^1^, K. Pappa^9^, R. Wang^9^, J. Wright^10^, A.‐R. Tenorio^9^, B. Wynne^9^, M. Aboud^1^, M. Gartland^9^ and K. Smith^9^**

^1^ViiV Healthcare, Brentford, United Kingdom, ^2^Centre Hospitalier de Tourcoing, Tourcoing, France, ^3^Holdsworth House Medical Brisbane, Queensland, Australia, ^4^CHU St‐Pierre, Brussels, Belgium, ^5^Triple O Research Institute PA, West Palm Beach, United States, ^6^Hospital General Universitario de Alicante, Alicante, Spain, ^7^McGill University Health Center, Montreal, Canada, ^8^Praxis am Ebertplatz, Cologne, Germany, ^9^ViiV Healthcare, Research Triangle Park, United States, ^10^GlaxoSmithKline, Stockley Park, United Kingdom

**Background: **DTG + 3TC 2‐drug regimen (2DR) is noninferior to DTG+TDF/FTC 3 drug regimen in HIV‐1 infected ART‐naïve adults. Efficacy and safety of switching to DTG + 3TC in ART‐experienced adults suppressed on 3DRs have been demonstrated in smaller studies.

**Methods: **TANGO, a randomized, open‐label, multicenter, non‐inferiority Phase III study evaluates efficacy and safety of switching to DTG + 3TC once daily in HIV‐1 infected adults on TBR with HIV‐1 RNA <50 c/mL for >6 months, without prior virologic failure, no historical NRTI or INSTI major resistance mutations. Participants were randomized 1:1 (stratified by baseline 3^rd^ agent class: PI, NNRTI, INI) to switch to DTG + 3TC or continue TBR through Wk148. Primary endpoint: proportion of participants with plasma HIV‐1 RNA ≥ 50c/mL at Week 48 (FDA Snapshot algorithm) for Intention To Treat‐Exposed (ITT‐E) population. Planned Wk24 interim analysis assessed non‐inferiority of DTG + 3TC with 4% non‐inferiority (NI)margin. Secondary endpoint: Virologic suppression (HIV‐1 RNA < 50c/mL by FDA Snapshot, ITT‐E) with 8% NI margin.

**Results: **741 randomized/exposed participants (DTG + 3TC: 369; TBR: 372). demonstrated switching to DTG + 3TC was non‐inferior to continuing TBR at Week 24 – Snapshot Virologic Failure: < 1% vs. < 1%; adjusted difference: ‐0.5% (95% CI: ‐1.6%, 0.5%). Proportion with plasma HIV‐1 RNA <50 c/mL was high and similar in both arms and demonstrated non‐inferiority (Table 1). Zero participant on DTG + 3TC and 1 participant (< 1%) on TBR met protocol‐defined virologic failure with no resistance mutations observed at failure. No unexpected AEs were identified for DTG or 3TC.

Abstract WEAB0403LB‐Table 1. Efficacy and key safety results for the ITT‐E and safety population

**Table:** 

Week 24 Study Outcome by Snapshot Analysis (ITT‐E Population)	DTG + 3TC (N = 369) n (%)	TBR (N = 372) n (%)
HIV‐1 RNA ≥ 50 c/mL (Snapshot Virologic Failure)	1 (<1)	3 (<1)
HIV‐1 RNA < 50 c/mL (Snaphot Virologic Success)^a^	350 (95)	358 (96)
No Virologic Data at Week 24 Window	18 (5)	11 (3)
**Key Safety results (Safety Population)**	**(N = 369)**	**(N = 371** b **)**
AEs or death leading to withdrawal^a^	12 (3)	1 (<1)
Drug‐related Grade 2‐5 AEs^a^	15 (4)	3 (<1)
Serious Adverse Events (none related to study treatment)	14 (4)	8 (2)

^a^Snapshot Virologic Success adjusted difference in (DTG + 3TC) ‐ TBR: ‐1.4% (95% CI: ‐4.4%, 1.6%). Estimates and confidence intervals were based on a stratified analysis using Cochran‐Mantel‐Haenszel weights adjusting for baseline 3rd agent class. ^b^One subject was excluded due to receiving a TDF‐based regimen instead of a TAF‐based regimen ^c^One death (homicide) unrelated to treatment occurred in the DTG + 3TC arm.

**Conclusions: **At Wk24, switching to DTG/3TC FDC was non‐inferior to continuing a TAF‐based 3DR in maintaining virologic suppression in HIV‐1 infected ART‐experienced adults. The safety profile of DTG/3TC FDC was consistent with the DTG and 3TC respective labels. DTG/3TC 2DR offers a new robust switch option with reduced ART exposure, without increased risk of virologic failure or resistance. The study is ongoing; conference presentation will include Wk48 results.

## WEAB0404LB

### Durable efficacy of dolutegravir (DTG) plus lamivudine (3TC) in antiretroviral treatment‐naïve adults with HIV‐1 infection – 96‐week results from the GEMINI studies

**P. Cahn^1^, J. Sierra‐Madero^2^, J. Arribas^3^, A. Antinori^4^, R. Ortiz^5^, A. Clarke^6,7^, C.‐C. Hung^8^, J. Rockstroh^9^, P.‐M. Girard^10^, J. Sievers^11^, C. Man^12^, R. Urbaityte^13^, M. Underwood^12^, A.‐R. Tenorio^12^, K. Pappa^12^, B. Wynne^12^, M. Gartland^12^, M. Aboud^11^, J. van Wyk^11^ and K. Smith^12^**

^1^Fundación Huesped, Buenos Aires, Argentina, ^2^Instituto Nacional de Ciencias Médicas y Nutrición Salvador Zubirán, Mexico City, Mexico, ^3^Hospital La Paz, Madrid, Spain, ^4^Istituto Nazionale per le Malattie Infettive Lazzaro Spallanzani, Rome, Italy, ^5^Bliss Healthcare Services, Orlando, United States, ^6^Royal Sussex County Hospital, Brighton, United Kingdom, ^7^Brighton & Sussex Medical School, Brighton, United Kingdom, ^8^National Taiwan University Hospital, Taipei, Taiwan, Province of China, ^9^Rheinische Friedrich‐Wilhelms Universität, Bonn, Germany, ^10^Hôpital Saint Antoine, Paris, France, ^11^ViiV Healthcare, Brentford, United Kingdom, ^12^ViiV Healthcare, Research Triangle Park, United States, ^13^GlaxoSmithKline, Stockley Park, United Kingdom

**Background: **Compared with 3‐drug regimens, two‐drug regimens (2DR) have the potential to reduce cumulative drug exposure during life‐long antiretroviral therapy in HIV‐1 infected patients. In GEMINI‐1 and GEMINI‐2 (ClinicalTrials.gov: NCT02831673/NCT02831764), the efficacy of the 2DR of DTG + 3TC was non‐inferior to DTG+ tenofovir/emtricitabine (TDF/FTC) at week 48 in treatment‐naïve adults.

**Methods: **GEMINI‐1&2 are identical double‐blind, multicentre Phase III studies. Participants with HIV‐1 RNA ≤ 500,000c/mL at screening were randomised 1:1 (stratified by plasma HIV‐1 RNA and CD4 + cell count) to once‐daily treatment with DTG + 3TC or DTG+TDF/FTC. The primary endpoint was the proportion of participants with plasma HIV‐1 RNA < 50c/mL at week 48 (Snapshot algorithm). We present efficacy and safety data from prespecified 96‐week secondary analyses. Estimates and confidence intervals were based on a stratified analysis using Cochran‐Mantel‐Haenszel weights.

**Results: **714 and 719 adults were randomised and treated in GEMINI‐1&2, respectively. At baseline, 20% had HIV‐1 RNA > 100,000c/mL, 8% had CD4 + < 200cells/mm^3^. At week 96, DTG + 3TC was non‐inferior to DTG+TDF/FTC in both GEMINI‐1&2 and in the pooled analysis (using a 10% non‐inferiority margin) [Table 1]. Response rates in participants with baseline HIV‐1 RNA > 100,000c/mL were high and similar between arms. Consistent with week 48 outcomes, response remained lower in DTG + 3TC participants with CD4 + < 200cells/mm^3^. Across both studies, 11 participants on DTG + 3TC and 7 on DTG+TDF/FTC met protocol‐defined virologic withdrawal criteria through week 96; none had treatment‐emergent integrase strand transfer inhibitor or NRTI resistance mutations. Overall rates of AEs were similar, with low rates of withdrawals due to AEs in both arms. Numerically, more drug‐related AEs were reported with DTG+TDF/FTC. Post‐baseline changes in markers of bone and renal function favoured DTG + 3TC through week 96.

Abstract WEAB0404LB‐Table 1. Proportion of participants with plasma HIV‐1 RNA < 50 c/mL at week 96: Snapshot analysis – ITT‐E population

**Table:** 

		GEMINI‐1	GEMINI‐2	Pooled
Snapshot responders	DTG + 3TC	300/356 (84%)	316/360 (88%)	616/716 (86%)
	DTG+TDF/FTC	320/358 (89%)	322/359 (90%)	642/717 (90%)
Adjusted Difference (95% CI)		−4.9 (−9.8, 0.0)	−1.8 (−6.4, 2.7)	−3.4 (−6.7, 0.0)

**Conclusions: **DTG + 3TC remains non‐inferior to DTG+TDF/FTC in treatment‐naïve adults at week 96, with no increased risk of virologic failure and no treatment‐emergent resistance. Both regimens were well tolerated; biomarkers of bone turnover and renal function significantly favoured DTG + 3TC. The results demonstrate durable efficacy and potency of DTG + 3TC, further supporting it as a compelling option for HIV treatment. GEMINI‐1&2 continue until week 148.

## WEAB0405LB

### The ADVANCE trial: Phase 3, randomized comparison of TAF/FTC/DTG, TDF/FTC/DTG or TDF/FTC/EFV for first‐line treatment of HIV‐1 infection

**W.F. Venter^1^, M. Moorhouse^1^, S. Sokhela^1^, L. Fairlee^1^, N. Mashabane^1^, M. Masenya^1^, A. Qavi^2^, P. Clayden^3^, E.J. Abrams^4^, B. Simmons^2^ and A. Hill^5^**

^1^University of Witwatersrand, Health Sciences, Johannesburg, South Africa, ^2^Imperial College, Faculty of Medicine, London, United Kingdom, ^3^HIV i‐Base, London, United Kingdom, ^4^Columbia University, ICAP, Maidman School of Public Health, New York, United States, ^5^University of Liverpool, Translational Medicine, Liverpool, United Kingdom

**Background: **In low‐ and middle‐income countries, most treatment‐naïve people living with HIV (PLWH) take tenofovir disoproxil fumarate (TDF) with FTC (or 3TC) and efavirenz (EFV). Dolutegravir (DTG) and tenofovir alafenamide fumarate (TAF) are recommended in international guidelines, but clinical experience with these ARVs in sub‐Saharan Africa is limited. In South Africa, over 10% of patients have transmitted NNRTI drug resistance.

**Methods: **We conducted a 96‐week, open‐label randomised trial in South Africa, comparing TAF/FTC/DTG, TDF/FTC/DTG and TDF/FTC/EFV. Inclusion criteria included age ≥12 years, no prior ART >30 days, creatinine clearance > 60 mL/min (>80 mL/min if <19 years), and HIV‐1 RNA >500 copies/mL. Pregnancy and tuberculosis (TB) were exclusion criteria. There was no screening for baseline drug resistance, consistent with South African treatment guidelines. The primary treatment failure endpoint was 48‐week HIV‐1 RNA >50 copies/mL, discontinuation or missing data (Intent‐to‐treat population, non‐inferiority margin ‐10%, significance level *p *=* *0.017, adjusted for multiple comparisons). We report 48‐week efficacy and safety data.

**Results: **We randomised 1053 PLWH between February 2017 and May 2018: 99% black, 59% female, mean age 32 years, with mean CD4 336 cells/uL. At week 48, the percentage of participants with HIV RNA <50 copies/mL was 83.8% for TAF/FTC/DTG, 84.9% for TDF/FTC/DTG and 78.6% for TDF/FTC/EFV. In the on‐treatment analysis, 96% of participants on TAF/FTC/DTG, 94% on TDF/FTC/DTG and 95% on TDF/FTC/EFV had HIV RNA <50 copies/mL at Week 48. Both DTG arms demonstrated non‐inferior efficacy versus the EFV arm. Over 70% of participants with HIV RNA >50 copies/mL re‐supressed after adherence counselling and re‐testing. Overall, 136/185 (74%) of treatment failures were from discontinuation. Clinical adverse events and laboratory abnormalities were similar between treatment arms.

**Conclusions: **In the ADVANCE study, TAF/FTC/DTG and TDF/FTC/DTG demonstrated non‐inferior efficacy versus TDF/FTC/EFV, with low rates of virologic failure in all three arms despite country‐level background NRTI/NNRTI resistance. There were more discontinuations for adverse events in the TDF/FTC/EFV arm.

Abstract WEAB0405LB‐Table 1. ADVANCE trial results at Week 48

**Table:** 

Treatment arm	TAF/FTC/DTG	TDF/FTC/DTG	TDF/FTC/EFV
n	n = 351	n = 351	n = 351
Week 48 Efficacy			
HIV RNA < 50 copies/mL	294 (83.8%)	298 (84.9%)	276 (78.6%)
HIV RNA > 50 copies/mL	16 (4.6%)	19 (5.4%)	14 (4.0%)
Discontinuation for adverse events	2 (0.6%)	1 (0.3%)	12 (3.4%)
Discontinuation for other reasons	39 (11.1%)	33 (9.4%)	49 (14.0%)

## WEAB0406LB

### ANRS 170 QUATUOR 4/7 days maintenance strategy in antiretroviral treated adults with HIV‐1 infection: an open randomised parallel non‐inferiority phase III trial

**R. Landman^1,2^, P. De Truchis^3^, L. Assoumou^4^, S. Lambert^5^, K. Amat^2^, J. Bellet^4^, B. Lefebvre^6^, C. Allavena^7^, C. Katlama^8^, Y. Yazdanpanah^1^, J.‐M. Molina^9^, A. Gelley^10^, S. Gibowski^10^, J.‐C. Alvarez^11^, L. Morand‐Joubert^5^, D. Costagliola^4^, P.‐M. Girard^4^ and ANRS 170 QUATUOR study group**

^1^IAME, UMR 1137, INSERM, Université Paris Diderot, Sorbonne Paris Cité, AP‐HP, Hôpital Bichat, AP‐HP, Infectious and tropical diseases, Paris, France, ^2^Institut de Médecine et Epidémiologie Appliquée, Hôpital Bichat, Université Paris 7, Paris, France, ^3^Hôpitaux Universitaires Paris‐Ile de France‐Ouest, Hôpital Raymond Poincaré APHP, Université Versailles‐Saint‐Quentin, France, Infectious diseases department, GARCHES, France, ^4^Sorbonne Universités, INSERM, UPMC Univ Paris 06, Institut Pierre Louis d'Epidémiologie et de Santé Publique (IPLESP UMRS 1136), Paris, France, ^5^Hôpital Saint‐Antoine, Laboratoire de virologie, Paris, France, ^6^Hôpital Saint‐Antoine, Infectious diseases department, Paris, France, ^7^Hôpital Hôtel‐Dieu, Infectious diseases department, NANTES, France, ^8^Hôpital Pitié‐Salpêtrière, Infectious diseases department, Paris, France, ^9^Hôpital Saint‐Louis, Infectious diseases department, Paris, France, ^10^ANRS, France REcherche Nord & sud Sida‐hiv Hépatites, Agence autonome de l'INSERM, Paris, France, ^11^Hôpital R Poincaré APHP, Inserm U‐1173, Université Paris‐Ile de France Ouest, Pharmacology department, GARCHES, France

**Background: **Intermittent treatment could improve the convenience, tolerability and cost of ART. We have previously shown a 96% success rate of a maintenance four days a week (four/seven days) antiretroviral strategy in the ANRS 162 4D pilot study. The current study was designed to demonstrate the non‐inferiority of this strategy versus 7/7 days in patients with controlled viral load (VL) under triple therapy with either PI, NNRTI, or InSTI based regimen.

**Methods: **We conducted an open‐label, randomised, multicentric, non‐inferiority phase III trial evaluating efficacy and safety of a maintenance 4‐days a week therapy (four/seven days) versus current triple ART regimen (CAR). Adults with plasma VL <50 copies/mL for >12 months and no resistance mutations to CAR were randomly assigned (1:1) with stratification by third‐agent class. The primary endpoint was the Kaplan‐Meier estimated proportion of participants with treatment success (VL < 50 copies/mL and no treatment strategy modification) at week 48 among those starting the study strategy. We calculated the Cochran‐Mantel‐Haenszel treatment difference adjusted for the stratification factor, with a 5% non‐inferiority margin. ClinicalTrials.gov: NCT03256422.

**Results: **Participants were screened from Sep 7, 2017, to Jan 22, 2018. Among 647 randomised participants, 636 were included in the modified intent‐to‐treat analysis (318 in each arm). At entry, median age was 49 years (IQR 41 to 55), 85% were male, with VL < 50 copies/mL for 5.8 (3.3 to 9.6) years, median CD4 689 (533 to 884) cells/mL; NRTI: 56.3% TDF/FTC, 16.3% TAF/FTC, 27.4% ABC/3TC; 3rd agent: 6% PI, 46% NNRTI, 48% InSTI. At week 48 (last patient visit was April 4, 2019), the treatment success rate was 95.6% in the 4/7 days arm versus 97.2% with CAR (adjusted difference of ‐1.6%, 95% CI ‐4.5% to 1.3%), demonstrating the non‐inferiority. Six (1.9%) and 4 (1.3%) participants experienced virological failure with selection of resistance mutations in 3 and 1, respectively. No difference in adverse events was observed between the two arms. A moderate improvement of eGFR was observed in the 4/7 days arm, +5.5[−1.2 to +13.6] mL/min vs. * *+* *1.3[‐6.1‐+7.5] mL/min in CAR, P < 0.001.

**Conclusions: **The ANRS 170 QUATUOR randomised trial demonstrates the non‐inferiority of a 4/7 days maintenance strategy versus 7/7 days regimen.

## WESY0802LB

### Qualitative evaluation of PrEP implementation in Brazil – ImPREP stakeholders study

**M.C. Pimenta Oliveira^1,2^, X.P. Bermúdez^3^, A.M. Godoi^4^, M. Benedetti^5^, I. Maksud^6^, A. Martins^3^, B. Lopes^3^, B. Grinsztejn^5^ and V.G. Veloso^5^**

^1^Veiga de Almeida University, School of Nursing, Rio de Janeiro, Brazil, ^2^Ministry of Health – Brazil, STI/HIV and Viral Hepatitis, Brasilia, Brazil, ^3^Universidade de Brasilia, Laboratório de Antropologia da Saúde (LABAS), Brasilia, Brazil, ^4^Universidade de Brasilia, Laboratório de Antropologia da Saúde (LABAS), Brazilia, Brazil, ^5^Fiocruz, Instituto Nacional de Infectologia Evandro Chagas (INI), Rio de Janeiro, Brazil, ^6^Fiocruz, Instituto Fernandes Figueiras (IFF‐Fiocruz), Rio, de Janeiro, Brazil

**Background: **Pre‐exposure prophylaxis (PrEP) became available in Brazil in 2018 to populations at substantial risk of HIV infection as an additional prevention tool under a combination prevention strategy. The Implementation PrEP Project (ImPrEP) aims to generate evidence to support the incorporation of PrEP in Brazil. There is scarce qualitative evidence around PrEP implementation globally. This abstract presents initial findings from a qualitative study conducted with key PrEP stakeholders to gather insight on facilitators and barriers to PrEP implementation within a public health context.

**Methods: **Qualitative study, based on in‐depth structured interviews conducted face‐to‐face with 4 categories of HIV stakeholders in 6 capital cities implementing ImPrEP. Views, perceptions and experiences regarding PrEP and PrEP service incorporation were collected. Interviewees signed consent forms. Interviews were recorded and transcribed, organized and categorized using NVivo 12 Plus software. Discourse was systematized and analyzed based on narrative categories to extract meanings, perceptions and concerns of PrEP policy, its related conceptions, and experiences of key public health HIV program managers, civil society leaders and PrEP users as interlocutors of PrEP policy implementation.

**Results: **A total of 60 interviews have been conducted: 15 service managers; 14 health professionals; 16 PrEP users; 7 MSM and 7 Transgender community leaders, and 1 HIV+ leader. All health service managers, community leaders and PrEP users expressed prior knowledge of PrEP. Stigma and discrimination were cited as the main obstacle for most vulnerable populations to access PrEP services. Service providers and community leaders highlighted that transgender people are the ones who least frequent PrEP services. There is a narrative consensus that combined HIV prevention including PrEP is a step forward in public health policy. An aspect emphasized by health professionals and service managers was that PrEP puts HIV prevention under the individual's control. Structural health service issues standout as a restriction to meet increasing demand such as limited business hours and number of health professionals.

**Conclusions: **Perceptions about PrEP and difficulties of implementation were raised, particularly access barriers of populations at greater risk. Study findings point‐out that PrEP scale‐up within a public health context has culturally specific needs to be addressed.

## Poster Discussion

### MOPDA0104LB

#### Highly sensitive, limiting dilution‐multiplex seminested quantitative PCR‐based assay reveals a large active HIV‐1 reservoir in infected ART‐suppressed individuals and elite suppressors

**K. Groen^1^, M. Feuchert^1^, Y. Verschoor^1^, M.R. Pinzone^2^, L. DeMaster^2^, U. O'Doherty^2^, B. Berkhout^1^ and A. Pasternak^1^**

^1^Academic Medical Center of the University of Amsterdam, Medical Microbiology, Amsterdam, Netherlands, ^2^University of Pennsylvania, Pathology and Laboratory Medicine, Philadelphia, United States

**Background: **Improved assays are necessary to better characterize the HIV‐1 reservoir and to reliably monitor curative interventions. Here we describe a novel highly sensitive assay to measure the size of the active HIV‐1 reservoir in infected individuals.

**Methods: **We developed a new limiting dilution‐multiplex seminested quantitative PCR‐based assay that allows measuring frequencies of cells transcribing HIV‐1 cell‐associated unspliced (US, *gag*) and multiply spliced (MS, *tat/rev/nef*) RNA, bypassing the need to partition the samples for measuring different HIV‐1 RNA species. This approach results in improved assay sensitivity with the theoretical detection limit of one HIV‐1 RNA+ cell in 7.6 million cells, based on the typical experimental setup.

**Results: **Levels of total and integrated HIV‐1 DNA, as well as frequencies of HIV‐1 US RNA+ and MS RNA+ cells, were measured in PBMC from three HIV‐infected viremic therapy‐naïve individuals, five ART‐treated individuals with undetectable plasma viremia, and two elite suppressors, in the absence of *ex vivo* stimulation. All HIV‐1 forms were detectable in all individuals, with the highest levels of HIV‐1 DNA and frequencies of HIV‐1 US and MS RNA+ cells measured in therapy‐naïve, intermediate in ART‐treated, and the lowest in elite suppressors (averages of 1136‐340‐14 US RNA+ cells and 66‐7‐0.3 MS RNA+ cells per million PBMC, respectively). Surprisingly, in ART‐treated individuals and elite suppressors, the majority (averages of 57.6% and 68.1%, respectively) of HIV‐1 integrated DNA‐positive cells transcribed US RNA, but only 3.0% and 2.3% of these US RNA+ cells, respectively, transcribed MS RNA. However, in therapy‐naïve individuals, only 25.6% of HIV‐1 integrated DNA‐positive cells transcribed US RNA, but of the latter, 8.9% transcribed MS RNA.

**Conclusions: **This sensitive assay revealed a large, previously underestimated, HIV‐1 US RNA+ reservoir in ART‐treated individuals and elite suppressors. However, only a small percentage of US RNA+ cells transcribe MS RNA, suggesting post‐transcriptional latency blocks or genetic defects. The relatively smaller active reservoir in therapy‐naïve individuals might be explained by a higher percentage of intact proviruses coupled to more robust immune responses that eliminate HIV RNA+ cells. This assay can serve as a robust tool for the measurement of HIV‐1 active reservoir, even without *ex vivo* stimulation.

### MOPDA0105LB

#### CD8 + lymphocyte mediated suppression of HIV expression in CD4 + T cells

**L. Franchitti, A. Willemse, J. Yoon, E. White, B. Cox, G. Silvestri and D. Kulpa**

Emory University School of Medicine, Atlanta, United States

**Background: **The persistence of HIV infection under ART is due to a reservoir of latently infected cells that remain indefinitely despite suppression of virus replication.Defining the mechanisms responsible for the establishment and maintenance of the HIV reservoir under ART has been the focus of HIV cure strategies. Recent studies have suggested that CD8 + T cells inhibit virus production in ART‐treated SIV‐infected rhesus macaques. However, the mechanisms responsible for this antiviral effect remain poorly understood.

**Methods: **To test the hypothesis that CD8 + T cells suppress virus production through silencing of HIV transcription, we used an innovative *in vitro*HIV latency model to examine the effect of CD8 + T cells in the establishment and reversal of HIV latency. To first examine HIV latency establishment, CD4 + T cells from HIV naïve donors were infected *in vitro*and then co‐cultured with activated CD8 + T lymphocytes at 1:1 or 1:5 ratios. CD4 + cell monoculture was included as a control. After 3 days of co‐culture, we assessed Gag expression by flow cytometry, and quantified the frequency of integrated HIV DNA by qPCR. To examine CD8 + T mediated suppression of HIV latency reversal, we stimulated latently infected CD4 + T cells and examined the frequency of Gag+ cells in the presence or absence of CD8 + T cells (1:1 or 1:5 ratio).

**Results: **During HIV latency establishment, Gag expression in CD4 + T cells was reduced when co‐cultured with CD8 + T cells an average of 9‐fold (*p *<* *0.0001) and 18‐fold (*p *<* *0.0001) at 1:1 or 1:5 target: effector ratios respectively, without significantly reducing the frequency of HIV‐infected cells (n = 21). Notably, significant suppression of HIV latency reversal was observed upon TCR activation of latently infected CD4 + T cells in the presence of CD8 + lymphocytes, with a 6‐fold decrease at 1:1 target: effector ratio (*p *=* *0.0156) and 14‐fold decrease at 1:5 ratio (*p *=* *0.0156).

**Conclusions: **Our studies demonstrate that CD8 + T cells suppress HIV expression in CD4 + T cells in a way that promotes the establishment and maintenance of latency. Further understanding of the mechanisms by which CD8 + lymphocytes suppress virus transcription may support the design of new approaches for HIV eradication.

### MOPDA0106LB

#### The inexorability of latent reservoir formation: HIV induces quiescence in effector CD4^+^ T cells

**L. Plasek, L. Gunawardane, C. Dobrowolski, J. Karn and S. Valadkhan**

Case Western Reserve University, Molecular Biology and Microbiology, Cleveland, United States

**Background: **HIV preferentially infects activated effector cells but is unable to infect quiescent memory cells. It is therefore generally believed that the latent reservoir forms when a few rare effector cells enter quiescence and generate a memory cell population. Using polarized primary cell models, we have recently shown that virtually all productively infected cells become latently infected when they are forced into quiescence.

**Methods: **To identify the molecular pathways regulating T‐cell entry into quiescence, we performed RNA‐seq analyses of uninfected and HIV‐infected primary human CD4 + T cells during proliferation, upon induction of quiescence using a defined cocktail of cytokines, and following reactivation through TCR stimulation.

**Results: **In uninfected primary CD4^+^ T cells, entry into quiescence is associated with a dramatic downregulation of cellular pathways involved in the positive regulation of transcription, translation, cellular metabolism and the cell cycle. To our surprise, HIV infection strongly downregulated each of the cellular pathways involved in quiescence, leading to an overall gene expression pattern that closely resembled quiescent cells, whereas mock infected cells retained a proliferative phenotype. Re‐analysis of published RNA‐seq studies confirmed these transcriptomic signatures, demonstrating the reproducibility of these results in CD4 + T cells from different donors and after infection with different HIV strains. The shift to a quiescent phenotype is due to the upregulation of a number of a few key regulators, including the transcription factor KLF2 and a number of long non‐coding RNAs. Flow cytometry studies indicated that HIV‐infected cells have a reduced growth rate, which can be reversed by knockdown of KLF2 and other regulatory factors.

**Conclusions: **Our results demonstrate that rather than being a rare and random event, HIV entry into latency, and the consequent seeding of the HIV reservoir, is a direct result of the HIV‐induced shutdown of effector cell proliferation. This implies that intensification of antiviral treatment and early initiation of treatment will never be able to block the establishment of latency, and that interventions to reactivate the reservoir and enhance immune surveillance will be needed for the eradication of the HIV reservoir and develop a functional cure for HIV infections.

### MOPDD0106LB

#### Tuberculosis preventive therapy in select PEPFAR supported countries

**C. Godfrey, M. Bochnowitz, S. Ally, W. Paul, J. Cavanaugh, T. Al‐Samarrai**

PEPFAR, OGAC, Washington, United States

**Background: **Tuberculosis preventive therapy (TPT) reduces mortality in people living with HIV (PLH) and is a critical element in strategies to eliminate tuberculosis. Programmatic uptake has been limited even in countries where TPT is routinely recommended. In November 2018 PEPFAR committed to rapid and complete scale‐up of Tuberculosis Preventive Treatment (TPT) over two years. All eligible individuals who are on ART are expected to complete treatment by the end of 2020. In April 2019 each country was given a target for 2019 that represented approximately half of all eligible individuals receiving ART from PEPFAR supported facilities. The 2020 targets require that the remaining 50% of individuals receive TPT by the following year. This report evaluates baseline rates of TPT initiation and completion in 2018 prior to scale up of TPT throughout PEPFAR in 2019.

**Methods: **We conducted a descriptive analysis including sex differences of the number of PLH who were initiated and completed a course of isoniazid as preventive therapy in 2018. We restricted our analysis to PEPFAR supported countries in which TPT was initiated in more than 5% of PLH. We then tabulated these with the targets and designated budgets that were communicated to the country teams.

**Results: **Few PLH on ART were offered TPT in the target population. Completion rates were variable but did not exceed 75%. We did not observe sex differences in either individuals offered TPT or in completion rates (data not shown). The country targets for TPT completion and budget available for this activity is presented.

Abstract MOPDD0106LB‐Table 1. TPT Initiation, Completion and Targets 2018 to 2019

**Table:** 

Country	PLH on ART 2018	PLH who initiated TPT	TPT completion 2018 (completed 6 months INH/# scheduled)	Country Target 2019	Country Budget (USD)
Democratic Republic of the Congo	72143	20%	57%	45083	252464
Haiti	959697	9%	59%	48587	272087
Kenya	1066579	9%	75%	138951	778125
Lesotho	190569	2%	13%	132566	742370
Mozambique	1077726	9%	75%	138951	2604532
Nigeria	799718	8%	75%	382500	2142000
Eswatini	169272	6%	75%	43678	244597

**Conclusions: **Uptake of TPT is currently low in PEPFAR supported countries, but mandated targets and budgeting activities are expected ameliorate this since PEPFAR funding is dependent on reaching targets. Strategies to improve completion rates, including the use short‐course regimens are required.

### TUPDA0107LB

#### Contraceptive‐induced changes in genital tract HIV‐1 cellular targets and microbiota among women enrolled in the ECHO Trial

**H. Jaspan^1,2,3^, R. Bunjun^3^, B. Brown^1^, R. Tanko^3^, M. Onono^4^, G. Nair^5^, T. Palanee‐Phillips^6^, C. Scoville^2^, K. Heller^2^, S. Jaumdally^3^, S. Dabee^1,3^, H. Gamieldien^3^, J.M. Baeten^2^, S. Bosinger^7,8^, A.D. Burgener^9,10^, J.‐A.S. Passmore^3,11^ and R. Heffron^2^**

^1^Seattle Children's Research Institute, Seattle, United States, ^2^University of Washington, Seattle, United States, ^3^University of Cape Town, Cape Town, South Africa, ^4^Kenya Medical Research Institute, Kisumu, Kenya, ^5^Desmond Tutu HIV Centre, Cape Town, South Africa, ^6^Wits Reproductive Health & HIV Institute (Wits RHI), Johannesburg, South Africa, ^7^Emory University, Atlanta, United States, ^8^Yerkes National Primate Research Center, Atlanta, United States, ^9^Public health agency of canada, Winnipeg, Canada, ^10^University of Manitoba, Winnipeg, Canada, ^11^National Health Laboratory Service, Cape Town, South Africa

**Background: **Changes in vaginal microbiota, inflammation and mucosal HIV‐1 cellular targets may underlie the increased HIV‐1 risk observed in some studies of women using intramuscular depot medroxyprogesterone acetate (DMPA‐IM).

**Methods: **Within ECHO, designed to compare the relative HIV‐1 incidence among women randomized to DMPA‐IM, copper intrauterine device (Cu‐IUD) or levonorgesterol implant (LNG‐implant), this nested three‐site substudy aimed to evaluate the impact of these contraceptives on genital tract cytokines, T cell activation and microbiota. Women were randomly selected from among the 430 in the substudy for analyses of samples collected at enrollment (pre‐contraceptive initiation) and 1‐month post‐contraceptive initiation. From 72 women in Cape Town only, multiparameter flow cytometry was used to measure activation (CD38) and frequencies of cervical cytobrush‐derived Th17‐like cells (CD4 + CCR6 + CCR10‐) *ex vivo*. From 201 women in Cape Town, Johannesburg, and Kisumu, the 16S rRNA gene was amplified and sequenced from fluid collected via lateral vaginal wall swabs and 27 cytokine concentrations were measured via multiplex bead array on menstrual cup cervicovaginal secretions.

**Results: **Women randomized to DMPA‐IM, but not Cu‐IUD nor LNG‐implant, had significant increases in genital CD4 + Th17 cell frequencies relative to their baseline (median 44.9% to 57.9%, Wilcoxon *p *=* *0.012). Th17 cell frequencies 1‐month post‐initiation were also significantly higher compared to women randomized to Cu‐IUD (*p *<* *0.001) and LNG‐Implant (*p *=* *0.038). Activated CD38 + Th17 cells significantly increased between pre‐ and post‐contraceptive initiation among women in the DMPA‐IM arm (*p *=* *0.040) but not in the other arms. There were no changes in cervicovaginal cytokine concentrations after contraceptive initiation in any arm. Vaginal bacterial diversity increased significantly among women assigned to Cu‐IUD (mean Shannon index 1.22 versus 1.54, paired t‐test *p *=* *0.025) between baseline and post‐contraceptive initiation but not for women assigned to LNG‐implant or DMPA‐IM. Beta diversity significantly differed between arms post‐contraceptive initiation (PERMANOVA *p *=* *0.021), with women assigned to Cu‐IUD transitioning to more diverse bacterial communities.

**Conclusions: **These are the first data comparing markers of HIV‐1 risk among women randomized to effective contraceptives. Our observation that DMPA‐IM elicits increases in the frequency and activation status of Th17 cells, critical target cells for HIV, provides a plausible mechanism by which DMPA‐IM may influence HIV transmission.

### TUPDD0206LB

#### Quantifying the contribution of different aged men and women to onwards transmission of HIV‐1 in generalised epidemics in sub‐Saharan Africa: A modelling and phylogenetics approach from the HPTN071 (PopART) trial

**W. Probert^1^, M. Hall^1^, X. Xi^2^, R. Sauter^1^, T. Golubchik^1^, D. Bonsall^1^, L. Abeler‐Dörner^1^, M. Pickles^1^, A. Cori^2^, J. Bwalya^3^, S. Floyd^4^, N. Mandla^5^, K. Shanaube^3^, B. Yang^5^, H. Ayles^3,4^, P. Bock^5^, D. Donnell^6^, K. Grabowski^7^, D. Pillay^8^, A. Rambaut^9^, O. Ratmann^2^, S. Fidler^2^, R. Hayes^4^, C. Fraser^1^, PANGEA‐HIV consortium and the HPTN 071 (PopART) study team**

^1^University of Oxford, Oxford, United Kingdom, ^2^Imperial College, London, United Kingdom, ^3^Zambart, Lusaka, Zambia, ^4^London School of Hygiene and Tropical Medicine, London, United Kingdom, ^5^Desmond Tutu TB Centre, Stellenbosch University, Cape Town, South Africa, ^6^Fred Hutchinson Cancer Research Center, Seattle, United States, ^7^Bloomberg School of Public Health, Johns Hopkins University, Baltimore, United States, ^8^African Health Research Institute, Durban, South Africa, ^9^Edinburgh University, Edinburgh, United Kingdom

**Background: **Understanding the spread of HIV during HIV prevention trials has high potential to inform future intervention programming. Here, we provide a first characterisation of the residual age‐ and gender‐specific transmission dynamics during the HPTN 071 (PopART) trial using orthogonal methods, and investigate the potential impact of suppressing transmissions from inferred source populations.

**Methods: **First, epidemic predictions were made using an individual‐based model (IBM) calibrated on trial data. Model parameters of the sexual network were derived from surveys on sexual behaviour. Second, model predictions were tested against phylogenetic estimates obtained with *phyloscanner* from viral short‐read next‐generation sequencing (NGS) data from three trial communities in Zambia.

**Results: **Phylogenetic analysis identified 180 probable transmission pairs and the direction of transmission between them. 62% of these transmissions were from men to women, the same as was predicted by the IBM. The phylogenetic analysis predicted men to be 5.4 years than women in male‐to‐female transmission (IBM predicted 5.5 years), and 3.9 years older in female‐to‐male transmissions (the IBM predicted 2.9 years). The age distribution of transmitters agreed with modelling predictions, more closely after adjusting for sampling bias (Figure 1). According to both the model and phylogenetics analysis, onwards transmissions peaked in 25 to 29 year old (y.o.) men and 20 to 24 y.o. women. Modelling the prevention of all transmissions from 25 to 29 y.o. men and 20 to 24 y.o. women reduced cumulative incidence over the trial period (mid‐2014 to 2018) by 20% and 19% respectively.

**Conclusions: **Our results validate predictions of a mathematical model using phylogenetic data. These results support observations that there is a significant contribution of young people to HIV transmission in sub‐Saharan Africa, especially 25 to 29 y.o. men. These results highlight that if universal testing‐and‐treatment (UTT) does not reach young people, and 25 to 29 y.o. men in particular, then the effect of UTT on reducing incidence may be limited.

Abstract TUPDD0206LB‐Figure 1.107